# Gene expression profiling of *Hfe*^-/- ^liver and duodenum in mouse strains with differing susceptibilities to iron loading: identification of transcriptional regulatory targets of Hfe and potential hemochromatosis modifiers

**DOI:** 10.1186/gb-2007-8-10-r221

**Published:** 2007-10-18

**Authors:** Hélène Coppin, Valérie Darnaud, Léon Kautz, Delphine Meynard, Marc Aubry, Jean Mosser, Maria Martinez, Marie-Paule Roth

**Affiliations:** 1 INSERM, U563, Centre de Physiopathologie de Toulouse Purpan, Toulouse, F-31300 France; 2 Université Toulouse III Paul-Sabatier, IFR 30, Toulouse, F-31400 France; 3 CNRS, UMR6061, Génétique et Développement, Rennes, F-35000 France; 4 Université de Rennes 1, IFR 140, Rennes, F-35000 France

## Abstract

**Background:**

*Hfe *disruption in mouse leads to experimental hemochromatosis by a mechanism that remains elusive. Affymetrix GeneChip^® ^Mouse Genome 430 2.0 microarrays and bioinformatics tools were used to characterize patterns of gene expression in the liver and the duodenum of wild-type and *Hfe*-deficient B6 and D2 mice (two inbred mouse strains with divergent iron loading severity in response to *Hfe *disruption), to clarify the mechanisms of Hfe action, and to identify potential modifier genes.

**Results:**

We identified 1,343 transcripts that were upregulated or downregulated in liver and 370 in duodenum of *Hfe*^-/- ^mice, as compared to wild-type mice of the same genetic background. In liver, *Hfe *disruption upregulated genes involved in antioxidant defense, reflecting mechanisms of hepatoprotection activated by iron overload. *Hfe *disruption also downregulated the expression of genes involved in fatty acid β-oxidation and cholesterol catabolism, and of genes participating in mitochondrial iron traffic, suggesting a link between Hfe and the mitochondrion in regulation of iron homeostasis. These latter alterations may contribute to the inappropriate iron deficiency signal sensed by the duodenal enterocytes of these mice, and the subsequent upregulation of the genes encoding the ferrireductase Dcytb and several iron transporters or facilitators of iron transport in the duodenum. In addition, for several genes differentially expressed between B6 and D2 mice, expression was regulated by loci overlapping with previously mapped *Hfe*-modifier loci.

**Conclusion:**

The expression patterns identified in this study contribute novel insights into the mechanisms of Hfe action and potential candidate genes for iron loading severity.

## Background

Hereditary hemochromatosis (HH) accounts for most of the iron overload disorders that occur in individuals of European descent. It is an autosomal-recessive condition that is characterized by increased absorption of iron from the gastrointestinal tract and progressive accumulation of catalytically active iron in parenchymal organs. This iron excess can cause tissue damage and result in serious medical complications, including cirrhosis, primary liver cancer, diabetes, cardiomyopathy, endocrine dysfunction, and arthritis [1]. In Northern Europe, most patients with HH are homozygous for a single mutation (C282Y) in the *HFE *gene (which encodes the hereditary hemochromatosis [HFE] protein) [2]. Although the C282Y mutation disrupts a disulfide bond required for proper folding of the HFE molecule, the exact mechanisms by which HFE regulates iron homeostasis remain elusive. HFE expression can result in either the accumulation or the depletion of intracellular iron stores, depending on the cell type, suggesting that HFE interacts with other proteins that are involved in either the import or the export of iron [3,4]. The challenge remains to identify these proteins.

Despite its high prevalence (approximately 5/1,000 individuals of Northern European descent), C282Y homozygosity is characterized by a low penetrance [5], and family studies have shown that genetic factors contribute to this reduced penetrance [6]. Polymorphisms of modifier genes may have profound effects on the dominance of the *HFE *gene defect itself and explain individual variations in excess iron absorption and their pathologic consequences among carriers of the HH-predisposing genotype. However, the exact nature of these modifier genes in HH remains unknown, which currently precludes accurate prediction of who, among C282Y homozygotes, is likely to develop clinically significant iron-storage disease.

Murine models of iron loading, such as *Hfe *knockout mice (*Hfe*^-/-^), provide a useful alternative to humans in which to elucidate the physiologic pathways that are involved in the HH disease process and identifying modifier loci [7,8]. We previously reported that, compared with the inbred mouse strain C57BL/6 (B6), the strain DBA/2 (D2) was particularly susceptible to iron loading in response to *Hfe *disruption [9], suggesting the existence of genes other than *HFE *that modify the severity of iron accumulation. We therefore took advantage of the marked phenotypic differences between these two strains to localize five chromosomal intervals that control hepatic iron loading [10]. Analysis of recombinant inbred strains and exploration of strain-specific gene expression changes that result from *Hfe *disruption should facilitate the identification of the *Hfe *modifiers that account for variable disease expression in these intervals.

Thus far, investigations of regulatory circuits in response to *Hfe *disruption haves not addressed possible strain differences and have been limited to IronChip cDNA microarrays customized to analyze a selection of 300 genes encoding proteins that are directly involved in iron metabolism or in linked pathways [11]. Of note, expression of genes that may still have unsuspected importance in iron metabolism cannot be explored using these customized microarrays. Our goal in the present study was to identify functional classes of genes and individual candidates that are involved in the perturbation of mechanisms of iron homeostasis that results from *Hfe *disruption, and to identify differences in gene expression profiles between the inbred mouse strains B6 and D2 that could explain their difference in iron accumulation. To achieve this goal, we used Affymetrix GeneChip^® ^Mouse Genome 430 2.0 arrays containing 45,101 probe sets for over 39,000 transcripts, including 34,000 well characterized mouse genes, and bioinformatics tools to characterize expression networks in the duodenum and the liver of wild-type control and *Hfe*^-/- ^B6 and D2 mice.

## Results

### Differential gene expression between *Hfe*-deficient and wild-type mice

**Figure 1:**
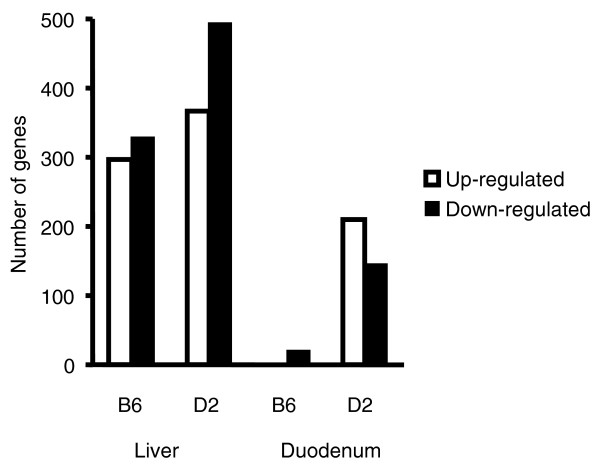
Number of genes regulated by *Hfe *disruption by mouse strain and organ studied. Genes regulated by *Hfe *disruption identified by statistical analysis of microarrays (SAM) were filtered to summarize the number of upregulated or downregulated genes in liver and duodenum. Genes were included if the mean S-score across three independent comparisons was ≥2 or ≤-2.

Microarray studies of liver and duodenum from *Hfe*^-/- ^mice identified 1,343 transcripts that were upregulated or downregulated in liver of either B6 or D2 *Hfe*^-/- ^mice, as compared with wild-type mice of the same genetic backgrounds. Much fewer genes, namely 370, were upregulated or downregulated in the duodenum of these mice. A list of the transcripts differentially regulated between *Hfe*-deficient and wild-type mice is provided in Additional data files 1 (liver) and 2 (duodenum). As shown in Figure 1, more transcripts were regulated in *Hfe*-deficient D2 mice than in B6 mice, and this difference was particularly striking in duodenum.

**Figure 2:**
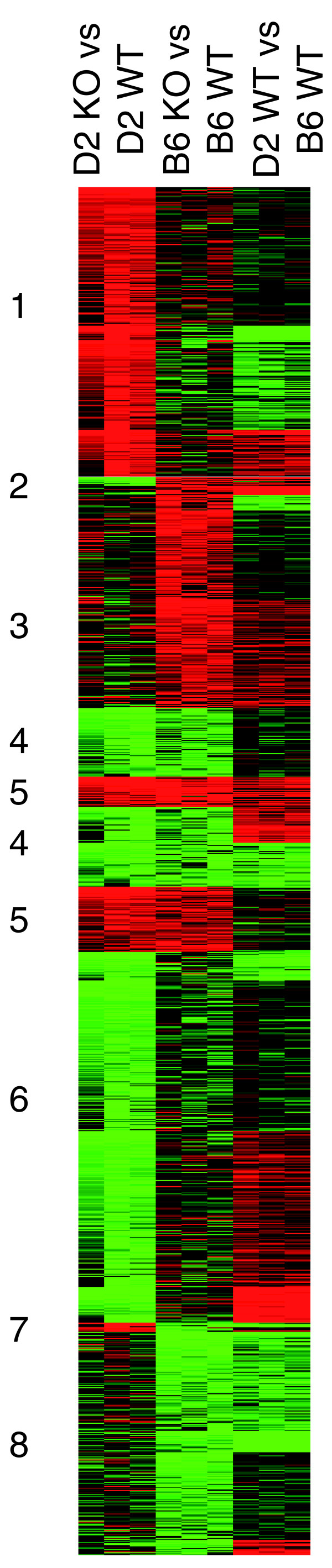
Genes regulated by *Hfe *deficiency in D2 and B6 liver. A tree view image of k-means clustering for 1,343 genes regulated by *Hfe *disruption in liver of D2 or B6 mice is shown. Genes were selected by statistical filtering of knockout (KO) versus wild-type (WT) S-scores, as described in Materials and methods. Corresponding values for wild-type D2 versus B6 S-scores are also shown. Red indicates upregulation by *Hfe *deficiency or more highly expressed in D2 mice; green indicates downregulation by *Hfe *deficiency or more highly expressed in B6 mice; and black indicates no difference.

In liver, clustering analysis detected groups of transcripts that were similarly regulated in response to *Hfe *disruption in B6 and D2 mice (specifically, they were either downregulated [Figure 2, cluster 4] or upregulated [cluster 5] in both strains). However, most of the transcripts modulated after *Hfe *disruption had expression patterns that were strain specific (regulated only in D2 mice [clusters 1 and 6] or only in B6 mice [clusters 3 and 8]).

In duodenum of B6 mice, the expression of fewer than 20 genes was significantly modified by *Hfe *deficiency (Figure 1). Consequently, clustering analysis was essentially based on expression changes in D2 mice. Two main clusters were therefore identified in duodenum, one with genes upregulated (cluster 1, Additional data file 2) and the other with genes downregulated (cluster 3, Additional file 2) in response to *Hfe *disruption in D2 mice.

### Enriched functional categories in the liver of *Hfe*-deficient mice

The Database for Annotation, Visualization, and Integrated Discovery (DAVID) annotation tool was used to search for over-representation of functional categories within the different gene clusters from Figure 2. Categories found to be enriched within the clusters of genes similarly regulated in the liver of *Hfe*^-/- ^compared with wild-type mice are summarized in Table 1. As detailed below, they mainly concern detoxification mechanisms in response to oxidative stress, fatty acid β-oxidation, cholesterol catabolism, and circadian rhythm.

**Table 1:** Functional categories over-represented in clusters of genes similarly regulated by *Hfe*-disruption in the liver

Category	Term	*n*	EASE score
Cluster 1 (284 Affy IDs [248 genes])			
GOTERM_BP	Steroid metabolism	11	1.4 × 10^-5^
GOTERM_MF	Mono-oxygenase activity	10	3.7 × 10^-5^
GOTERM_MF	UDP glucuronosyltransferase activity	5	2.9 × 10^-2^
Cluster 3 (218 Affy IDs [196 genes])			
	No functional category overrepresented		
Cluster 4 (145 Affy IDs [139 genes])			
GOTERM_BP	Rhythmic process	6	6.5 × 10^-5^
KEGG_PATHWAY	Fatty acid metabolism	7	3.2 × 10^-6^
GOTERM_BP	Defense response	14	4.7 × 10^-3^
GOTERM_BP	Nitrogen compound metabolism	9	5.6 × 10^-3^
Cluster 5 (94 Affy IDs [84 genes])			
KEGG_PATHWAY	Glutathione metabolism	8	5.8 × 10^-8^
GOTERM_MF	Iron ion binding	8	2.7 × 10^-4^
Cluster 6 (364 Affy IDs [315 genes])			
SP_PIR_KEYWORDS	Fatty acid metabolism	15	2.5 × 10^-14^
SP_PIR_KEYWORDS	Oxidoreductase	31	1.1 × 10^-8^
GOTERM_MF	Iron ion binding	19	1.6 × 10^-6^
KEGG_PATHWAY	Bile acid biosynthesis	6	1.1 × 10^-3^
GOTERM_BP	Cholesterol metabolism	6	3.2 × 10^-3^
Cluster 8 (219 Affy IDs [209 genes])			
	No functional category overrepresented		

Affymetrix probesets in the different k-means clusters shown in Figure 2 were compared with Affymetrix MG-430 2.0 probe sets for over-representation of gene categories, using the DAVID (Database for Annotation, Visualization, and Integrated Discovery) functional annotation tool. The Category column shows the original database/resource from which the terms originate. The Term column indicates enriched terms associated with the gene list. The *n *column indicates the number of genes involved in the term. The expression analysis systematic explorer (EASE) score is a modified Fisher exact *P *value [51]. BP, biological processes; GO, Gene Ontology; KEGG, Kyoto Encyclopedia of Genes and Genomes; MF, molecular functions; PIR, Protein Information Resource; UDP, Uridine 5'-diphospho.

#### Detoxification mechanisms in response to oxidative stress

The 84 genes from cluster 5 (Figure 2) and the 248 genes from cluster 1 that were induced by *Hfe*-deficiency in the liver were particularly enriched for functional categories associated with response to oxidative stress and iron ion binding (Table 2). Excess iron is known to generate reactive oxygen species that promote cell damage and fibrosis, and may be responsible for the induction of the aldehyde oxidase and NADPH (nicotinamide adenine dinucleotide phosphate) oxidase genes observed in these mice. This appears to be counterbalanced by upregulation of genes involved in the glutathione metabolism pathway, in particular genes encoding enzymes that are responsible for glutathione synthesis (*Gclc*, *Gclm*, and *Gss*) and glutathione *S*-transferases, which catalyze the conjugation of reduced glutathione to electrophilic centers on a wide variety of substrates; the latter activity is useful in the detoxification of endogenous compounds such as peroxidized lipids. Excess iron also appears to be counterbalanced, particularly in *Hfe*^-/- ^D2 mice, by upregulation of genes encoding uridine 5'-diphospho (UDP)-glucuronosyltransferases, which catalyze the glucuronidation reaction (the addition of sugars to lipids), which is an important step in the body's elimination of endogenous toxins. In addition, there was an enrichment, most notably in *Hfe*^-/- ^D2 mice, of genes with mono-oxygenase activity, particularly genes encoding several cytochrome P450 isoforms and flavin-containing mono-oxygenase-5, which are considered to be xenobiotic detoxication catalysts and believed to protect mammals from lipophilic nucleophilic chemicals [12]. The iron ion binding category, also enriched in the liver of both strains, includes the genes for ferroportin, ferritin light chain, and heme oxygenase, which catalyzes the degradation of heme into carbon monoxide and biliverdin. Of note, although expression of *vanin1 *was downregulated in mice lacking *Hfe *in both strains (cluster 4), this regulation is worth noting because mice deficient in vanin-1 exhibit a glutathione-mediated tissue resistance to oxidative stress [13].

**Table 2:** Main genes regulated by *Hfe *deficiency in liver and pertaining to enriched functional categories related to response to oxidative stress

Gene	Protein	S-score		
		
		D2 KO versus WT	B6 KO versus WT	D2 WT versus B6 WT
Glutathione metabolism pathway				
*Gclc*	Glutamate-cysteine ligase, catalytic subunit	3.68	4.74	3.24
*Gclm*	Glutamate-cysteine ligase, modifier subunit	NS	4.11	NS
*Gss*	Glutathione synthetase	NS	2.15	NS
*Gsta2*	Glutathione S-transferase alpha2	8.83	9.10	NS
*Gsta3*	Glutathione S-transferase alpha3	2.44	2.24	NS
*Gsta4*	Glutathione S-transferase alpha4	4.64	5.99	4.35
*Gstm1*	Glutathione S-transferase mu1	NS	2.65	NS
*Gstm3*	Glutathione S-transferase mu3	3.04	4.04	2.56
*Gstm6*	Glutathione S-transferase mu6	3.36	2.26	NS
UDP glucuronosyltransferase activity				
*Ugt2b1*	UDP glucuronosyltransferase 2B1	3.72	NS	NS
*Ugt2b5*	UDP glucuronosyltransferase 2B5	2.56	3.91	NS
*Ugt2b34*	UDP glucuronosyltransferase 2B34	NS	2.25	NS
*Ugt2b35*	UDP glucuronosyltransferase 2B35	2.56	3.91	NS
*Ugt2b36*	UDP glucuronosyltransferase 2B36	4.66	NS	-4.89
Mono-oxygenase activity				
*Cyp1a2*	Cytochrome P450 1A2	3.14	2.60	3.49
*Cyp2c29*	Cytochrome P450 2C29	NS	2.79	4.84
*Cup2c44*	Cytochrome P450 2C44	3.07	NS	-5.89
*Cyp2c55*	Cytochrome P450 2C55	3.94	6.22	4.43
*Cyp2c70*	Cytochrome P450 2C70	5.55	4.70	4.17
*Cyp2j6*	Cytochrome P450 2J6	2.70	NS	NS
*Cyp2j9*	Cytochrome P450 2J9	NSD	2.91	2.14
*Cyp2u1*	Cytochrome P450 2U1	3.50	NS	NS
*Fmo5*	Flavin mono-oxygenase 5	2.12	NS	NS
Iron ion binding				
*Ftl1*	Ferritin light chain 1	1.70	2.24	NS
*Slc40a1*	Ferroportin	3.18	4.89	3.94
*Hmox1*	Heme oxygenase 1	5.27	2.40	-3.66
*Blvrb*	Biliverdin reductase (for information)	2.50	3.06	NS
*Vnn1*	Vanin 1 (for information)	-4.27	-2.73	2.69

S-scores were obtained as described in Materials and methods and are proportional to fold changes. Positive S-scores indicate that the genes are more highly expressed in knockout (KO) than in wild-type (WT) mice, or in WT D2 than in WT B6 mice. NS, not significant; UDP, Uridine 5'-diphospho.

#### Fatty acid β-oxidation and cholesterol catabolism

The 139 genes from cluster 4 (Figure 2) and the 315 genes from cluster 6, which were repressed in liver by *Hfe *deficiency, were particularly enriched for functional categories associated with lipid metabolism (Table 3). In particular, genes encoding the rate-limiting enzyme for β-oxidation of long-chain fatty acids (*Cpt*) and the transcripts for enzymes involved in the three steps of β-oxidation were all significantly downregulated. The expression of the *Cyp4a10 *and *Cyp4a14 *genes was also repressed in *Hfe*^-/- ^mice of both strains, which could be a physiologic response in the context of the reduced fatty acid β-oxidation. With a decrease in acetyl-coenzyme A generated by decreased β-oxidation, a decrease in citrate (the first intermediate generated in the tricarboxylic acid [TCA] cycle) would be expected in the mitochondria of *Hfe*^-/- ^mice, with a subsequent slowing of the TCA cycle. Indeed, a downregulation of mitochondrial aconitase and isocitrate dehydrogenase suggests that the flux through the TCA cycle is maintained at a low level in order to adapt to the downregulated β-oxidation in these *Hfe*-deficient mice. Interestingly, the cholesterol metabolism category is also enriched among genes downregulated by *Hfe *deficiency in D2 mice, and this mainly affects genes that are involved in the catabolism of cholesterol into bile acids (*Cyp7a1 *and *Cyp39a1*).

**Table 3:** Main genes regulated by *Hfe *deficiency in liver and pertaining to the enriched functional categories fatty acid β-oxidation and cholesterol metabolism

Gene	Protein	S-score		
		D2 KO versus WT	B6 KO versus WT	D2 WT versus B6 WT
Fatty acid β-oxidation				
*Cpt1a*	Carnitine palmitoyl transferase 1a	-2.95	-1.94	NS
*Cpt2*	Carnitine palmitoyl transferase 2	-2.59	NS	NS
*Acadm*	Acyl-CoA dehydrogenase, medium chain	-2.97	-1.80	NS
*Acadl*	Acyl-CoA dehydrogenase, long chain	-3.00	NS	NS
*Acadvl*	Acyl-CaA dehydrogenase, very long chain	-2.40	NS	NS
*Ehhadh*	Enoyl-CoA hydratase/3-hydroxyacyl-CoA dehydrogenase	-2.30	NS	NS
*Hadha*	Tripartite protein, alpha subunit	-2.08	-1.58	NS
*Hadhb*	Tripartite protein, beta subunit	-2.96	-1.90	NS
*Hadh2*	Hydroxyacyl-CoA dehydrogenase type II	NS	-4.05	-4.61
*Acox1*	Acyl-CoA oxydase 1, palmitoyl (peroxisomal)	-2.10	-1.63	-4.27
*Cyp4a10*	Cytochrome P450 4A10	-6.76	-2.46	NS
*Cpy4a14*	Cytochrome P450 4A14	-9.21	-3.09	NS
TCA cycle				
*Aco2*	Aconitase 2, mitochondrial	-2.14	-1.82	NS
*Idh2*	Isocitrate dehydrogenase 2, mitochondrial	-2.09	NS	NS
Cholesterol catabolism				
*Cyp7a1*	Cholesterol 7α-hydroxylase	-3.15	NS	NS
*Cyp39a1*	Oxysterol 7α-hydroxylase	-2.60	NS	NS

S-scores were obtained as described in Material and methods and are proportional to fold changes. Negative S-scores indicate that the genes are more highly expressed in wild-type (WT) than in knockout (KO) mice, or in WT B6 than in WT D2 mice. Variations in the expression of genes involved in the tricarboxylic acid (TCA) cycle are provided for information. CoA, coenzyme A; NS, not significant.

#### Circadian rhythm

*Hfe*^-/- ^mice of both strains exhibit reduced expression of genes encoding Period (*Per2 *and *Per3*), D site albumin promoter binding protein (*Dpb*), and the nuclear receptor subfamily 1 (*Nr1d1*). Although surprising, this can be related to the recent observation that the circadian clock and heme biosynthesis are reciprocally regulated in mammals [14] and may be correlated with the upregulation of δ-aminolevulinate synthase (*Alas2*) in the liver of these mice.

#### Other variations of potential interest

*Hfe*^-/- ^D2 mice exhibit increased expression of the gene encoding 3β-hydroxysteroid dehydrogenase (*Hsd3b5*), which is thought to be involved in the inactivation of steroid hormones, for example dihydrotestosterone [15]. They also exhibit induction of the dopachrome tautomerase gene (*Dpt*), which affects pigmentation [16]. It would be interesting to investigate whether these variations in gene expression are related to the deficit in testosterone and melanodermia observed in patients with severe hemochromatosis.

### Enriched functional categories in the duodenum of *Hfe*-deficient mice

As shown in Table 4, there was no clearly enriched functional categories among the 177 genes (cluster 1) that were induced in #*Hfe*^-/- ^D2 mice. Conversely, there was significant enrichment of genes involved in the immune defense among the 131 genes that were repressed in the same mice (cluster 3), particularly for genes involved in apoptosis (*Casp4*, *Cdca7l*, *Ifit1 *and *Ifit2*, *Oasl2*, and *Scotin*), innate antiviral or antimicrobial activity (*Defcr4*, *Ddx58*, and *Lzp-s*), and B and T cell mediated immune response (*Mpa2l*, *Psme1*, *Trfrsf13b*, and *Tnfrsf17*). This suggests a link between the control of iron metabolism and the immune system that should be explored.

**Table 4:** Functional categories over-represented in clusters of genes similarly regulated by *Hfe *disruption in duodenum

Category	Term	*n*	EASE score
Cluster 1 (209 Affy IDs [177 genes])			
	No functional category overrepresented		
Cluster 3 (141 Affy IDs [131 genes])			
GOTERM_BP	Defense response	21	1.6 × 10^-7^
GOTERM_BP	Induction of apoptosis	6	1.7 × 10^-3^

Affymetrix probesets in the different k-means clusters shown in Additional data file 2 were compared with Affymetrix MG-430 2.0 probe sets for over-representation of gene categories, using the DAVID (Database for Annotation, Visualization, and Integrated Discovery) functional annotation tool. The Category column shows the original database/resource from which the terms originate. The Term column indicates enriched terms associated with the gene list. The *n *column indicates the number of genes involved in the term. The expression analysis systematic explorer (EASE) score is a modified Fisher exact *P *value [51]. BP, biological process; GO, Gene Ontology.

Although mRNAs for duodenal iron transporters were not found to be significantly upregulated, expression levels of other metal ion transporters were increased in duodenum of *Hfe*^-/- ^D2 mice, most notably the zinc transporters *Slc39a4 *and *Slc39a14*. The copper transporter *Slc31a1 *and, more anecdotally, the sodium-dependent vitamin C transporter *Slc23a2 *(previously observed to be increased in response to dietary iron deprivation [17]) were also induced in D2 mice lacking *Hfe*. In addition, *Hfe*^-/- ^D2 mice had increased expression of the mucin (*Muc3*) and spermin synthase (*Sms*) genes, which encode proteins that both may modulate iron uptake [18,19].

### Changes in expression of genes encoding proteins of iron metabolism

The Affymetrix GeneChip^® ^Mouse Genome 430 2.0 arrays contain probe sets for the transcripts of all the genes directly or indirectly involved in iron metabolism [20]. Significant alterations in their expression in liver or duodenum of *Hfe*^-/- ^mice and gene expression differences between wild-type strains are summarized in Table 5. Specifically in the D2 strain, *Hfe *disruption induces expression of the *Cybrd1 *gene in duodenum; this gene encodes Dcytb, which converts dietary ferric iron into its ferrous form for transport. In the liver, *Hfe*-deficient mice of both strains exhibit upregulated expression of the gene encoding the ferritin light chain, which is responsible for cytosolic iron storage, and of the ferroportin gene, which is consistent with the notion that this protein plays a protective role by facilitating the release of excess iron [21]. Somewhat unexpectedly, we observed significant downregulation of the sideroflexin gene (*Sfxn2*) and upregulation of the mitoferrin gene (*Slc25a37*) and the Bcrp gene (*Abcg2*), which encode three molecules that are involved in the mitochondrial import/traffic of iron and heme export. Also worthy of mention are several strain-specific modifications of the messengers of some regulators of iron metabolism in *Hfe*-deficient mice. First, we confirmed that wild-type B6 and D2 diverge in terms of the amounts of the two hepcidin messengers, namely *Hamp1 *and *Hamp2 *[22], and we observed a downregulation of the two genes in *Hfe*^-/- ^D2 mice. Conversely, we observed significant upregulation of the gene encoding the upstream transcription factor *Usf2*, which was recently found to be involved in the control of hepcidin expression [23], in the B6 strain. Finally, and worthy of note within the context of modifiers of iron loading severity, wild-type D2 mice have significantly lower expression of the Smad4 transcription factor, also involved in the control of hepcidin expression, than wild-type B6 mice.

**Table 5:** Changes in expression of genes involved in iron metabolism

Gene	Protein	Major biochemical activity	Role	Organ	S-score		
					
					D2 KO versus D2 WT	B6 KO versus B6 WT	D2 WT versus B6 WT
Iron storage							
*Ftl1*	Ferritin L chain	Fe mineralization	Cytosolic storage	Liver	+1.70	+2.24	NS
Iron transport							
*Slc40a1*	Ferroportin	Membrane transporter	Cellular export	Liver	+3.18	+4.89	+3.94
*Abcg2*	Bcrp	Membrane transporter	Possible mitochondrial heme export	Liver	+2.97	NS	NS
*Sfxn2*	Sideroflexin2	Membrane transporter	Mitochondrial traffic	Liver	-2.38	-2.16	NS
*Slc25a37*	Mitoferrin	Membrane transporter	Mitochondrial traffic	Liver	NS	+2.28	NS
*Lcn2*	Lipocalin2	Siderophore iron binding	Traffic of siderophore-bound iron	Liver	-2.91	NS	NS
Receptors							
*Tfrc*	Transferrin receptor1	Transferrin binding	Transferrin iron uptake	Duodenum	-2.07	NS	NS
*Lrp1*	LRP/CD91	Hemoplexin receptor	Hemoplexin uptake	Liver	NS	-2.03	NS
Regulators							
*Ireb2*	IRP2	RNA binding	Control of cellular iron	Duodenum	+2.26	NS	-2.64
*Hamp1*	Hepcidin 1	Ferroportin binding	Control of systemic iron	Liver	-6.27	NS	-3.57
*Hamp2*	Hepcidin 2	?	?	Liver	-3.40	NS	+3.36
*Hfe*	HFE	TfR1 binding	?	Liver	-7.96	-8.96	-3.16
				Duodenum	-5.70	-7.32	NS
*Hfe2*	HJV	Neogenin binding	Control of hepcidin expression	Liver	NS	-2.01	NS
*Fxn*	Frataxin	Iron binding	Chaperon for Fe-S synthesis	Liver	NS	NS	-3.09
*Smad4*	Smad4	Transcription factor	Control of hepcidin expression	Liver	NS	NS	-3.59
				Duodenum	NS	NS	-5.05
*Usf2*	Usf2	Transcription factor	Control of hepcidin expression	Liver	NS	+2.08	NS
Oxidoreductases							
*Cybrd1*	Dcytb	Fe(III) reduction	Facilitates duodenal transport by DMT1	Duodenum	+2.97	NS	NS

Iron metabolism genes are cited in this table where significant expression variations in *Hfe*^-/- ^mice (knockout [KO]) or expression differences between wild-type (WT) strains were detected. S-scores were obtained as described in Material and Methods and are proportional to fold changes. Positive S-scores indicate that the genes are more highly expressed in KO than in WT mice, or in WT D2 than in WT B6 mice. NS, not significant.

### Confirmation of differential gene expression by quantitative PCR

Quantitative real-time PCR was performed on 21 genes expressed in the liver and four genes expressed in the duodenum. The selection of these genes was based on different criteria. The first group included genes of an enriched functional category identified using the DAVID annotation tool (*Aox1*, *Ftl1*, *Fpn1*, *Hmox1*, *Vnn1*, *Por*, *Cpt1a*, *Aco2*, *Cyp7a1*, and *Hsd3b5*). The second group of genes encode proteins of iron or heme metabolism, and their expression was either induced or repressed in *Hfe*^-/- ^mice (*Hfe2*, *Hamp1*, *Hamp2*, *Usf2*, *Lcn2*, *Sfxn2*, *Alas2*, *Slc25a37*, and *Abcg2*). The third group encode proteins that might modulate iron absorption in the duodenum (*Dcytb*, *Slc39a4*, and *Muc3*). The fourth group includes genes that, although their involvement in iron metabolism regulation cannot be assumed, were highly regulated in liver (*Lcn13 *and *Fmo3*) or duodenum (*Clca4*) of *Hfe*^-/- ^mice. A further 20 mice that were not analyzed using Affymetrix arrays (five per genotype/strain combination) were included in the analysis to test the validity of the results.

Concordant results were obtained for 24 out of 25 genes selected. Downregulation of the hemojuvelin gene (*Hfe2*) in *Hfe*^-/- ^B6 mice was not confirmed. Downregulation of *Lcn2*, *Hamp1*, and *Hamp2 *in *Hfe*^-/- ^D2 mice was confirmed in the samples used for Affymetrix array hybridizations but not in the additional samples used for validation, although a trend toward downregulation was observed in the validation set for *Hamp1 *and *Hamp2*. The upregulation of *Usf2 and Slc25a37*, originally found only in the liver of *Hfe*^-/- ^B6 mice, was observed by quantitative PCR in both strains. Interestingly, *Lcn13 *and *Fmo3 *- which had highly significant S-scores of 11.06 and -6.66, respectively, in the liver of *Hfe*^-/- ^D2 mice - were confirmed to be regulated by *Hfe *deficiency in both datasets. Because neither of these two genes is regulated by dietary iron content in wild-type mice (data not shown), these variations appear specific to *Hfe *disruption and warrant further investigation.

### Correlation of expression profiling with studies on *Hfe *modifiers

Differences in liver or duodenal expression of specific genes between B6 and D2 wild-type mice could contribute to the divergent phenotypes induced by *Hfe *disruption in the two strains. We therefore established a list of the 1,538 transcripts with differential expression between wild-type D2 and B6 mice (Additional data file 3). In order to relate genomic results to severity of hemochromatosis, we first identified 210 genes exhibiting differences in basal expression between strains or with expression regulation in response to *Hfe *disruption, which reside within the five *Hfe*-modifier regions that we previously mapped on chromosomes 3, 7, 8, 11, and 12 [10]. To identify those that could be potential candidates for disease severity, we used the WebQTL interface to map the loci that regulate the expression of these genes. The information necessary to map these regulatory loci was available for a subset of 139 of these 210 genes.

We found that two genes on chromosome 3, four on chromosome 7, six on chromosome 8, 17 on chromosome 11, and one on chromosome 12 exhibited highly significant evidence for *cis *regulation (for regulation by a polymorphic variant between B6 and D2 mice located in the region of the gene itself; Table 6). None of them, except for *Hamp*, has yet been implicated in iron metabolism.

**Table 6:** Genes differentially expressed between wild-type strains or regulated by *Hfe deficiency*, located within the chromosomal regions containing *Hfe*-modifiers, and with evidence for *cis *regulation

Gene name	Chromosome	Position (Mb)	Type	Position of linkage peak (Mb) for *cis *regulator	Max LRS for *cis *regulator
*Clca2*	3	144.73	D	144.70 to 144.94	46.7
*Lphn2*	3	148.87	S	149.36 to 151.27	15.5
*Uble1a*	7	15.49	S	15.19 to 15.53	51.53
*Ckap1*	7	29.93	S	29.49 to 30.12	53.8
*Hamp1/Hamp2*	7	30.63	L, S	30.43 to 34.11	18.9
*Fxyd5*	7	30.74	D	34.41 to 34.62	15.2
*Gpsn2*	8	86.46	S	83.77 to 85.83	15.2
*Ddx39*	8	86.61	L	86.07 to 88.74	12.7
*2410018C20Rik*	8	87.14	S	86.07 to 88.74	20.6
*Ier2*	8	87.55	L	86.07 to 88.74	15.9
*Gadd45gip1*	8	87.72	S	86.07 to 88.74	116.4
*Prdx2*	8	87.86	S	83.77 to 85.83	36.1
*Pttg1*	11	43.26	S	42.87 to 44.25	68.9
*5730409G07Rik*	11	45.79	S	42.21 to 46.06	16.4
*2900006B13Rik*	11	51.43	L, S	50.95 to 53.90	38.9
*Tnip1*	11	54.75	S	50.95 to 53.90	60.6
*Sparc*	11	55.24	S	55.24 to 55.92	26.1
*Guk1*	11	59.00	S	58.93 to 59.04	50.8
*Sat2*	11	69.44	S	69.42 to 70.27	133.2
*Mpdu1*	11	69.47	S	72.49 to 72.98	43.3
*Asgr2*	11	69.91	S	69.42 to 70.27	69.9
*Rabep1*	11	70.66	L	73.93 to 75.08	18.3
*Txnl5*	11	72.02	S	67.96 to 68.74	11.1
*Pafah1b1*	11	74.49	L, S	75.29 to 76.41	14.5
*Crk*	11	75.50	L	76.76 to 76.83	10.5
*Ccl9*	11	83.39	L, S	88.48 to 89.36	36.9
*Bcas3*	11	85.17	S	89.57 to 89.92	12.2
*Dhx40*	11	86.59	S	83.52 to 88.25	36.8
*Scpep1*	11	88.74	L, S	83.52 to 88.25	39.2
*9030617O03Rik*	12	101.18	S	100.97 to 102.71	51.5

The Chromosome and Position columns indicate, respectively, the chromosome number and position (in megabases [Mb]) within one of the five *Hfe*-modifier intervals of the gene with expression variation. In the Type column, S indicates that expression differed between wild-type strains, D that expression was modulated by *Hfe *deficiency in duodenum, and L that expression was modulated by *Hfe *deficiency in liver. Max LRS indicates the maximum likelihood ratio statistic in favor of the *cis *regulator. Position of linkage peak for *cis *regulator and maximum LRS were retrieved from the WebQTL interface.

## Discussion

Recent advances in the field of iron metabolism have elucidated basic processes of iron absorption and distribution in mammals [24]. However, many aspects of iron metabolism remain obscure, in particular the mechanisms by which HFE regulates iron absorption. In this study we investigated the expression patterns of 34,000 well characterized mouse genes in liver and duodenum of wild-type and *Hfe*^-/- ^mice of two inbred strains with different susceptibilities to iron accumulation.

Variations in duodenal gene expression in *Hfe*-deficient mice, as compared with wild-type mice, are consistent with our previously reported hypothesis [9] that hyperabsorption of iron in these mice reflects an inappropriate iron deficiency signal that is sensed by duodenal enterocytes. Indeed, expression of the *Cybrd1 *gene (encoding Dcytb, which converts dietary ferric iron to its ferrous form for transport by the divalent metal iron transporter Dmt1 to the duodenum) and the expression levels of several metal ion transporters, most notably the zinc transporters Zip4 (*Slc39a4*) and Zip14 (*Slc39a14*), were increased in the duodenum of *Hfe*^-/- ^D2 mice. Although *Hfe *knockout was previously shown to increase *Cybrd1 *expression [11] and mucosal reductase activity near the villus tips [25], the increase in expression of the two zinc transporters has not yet been observed and is interesting within the context of recent reports indicating that Zip4 is a minor intestinal iron importer [26] and that Zip14 mediates non-transferrin-bound iron uptake into cells [27]. Of note, *Hfe*^-/- ^D2 mice also have increased duodenal expression of mucin and spermine synthase. Increased binding of Dmt1 to mucin in vesicles near the intestinal surface was observed in iron-deficient animals, which is believed to facilitate iron internalization [19], and recent studies have suggested that polyamines such as spermine modulate iron uptake [18].

**Figure 3:**
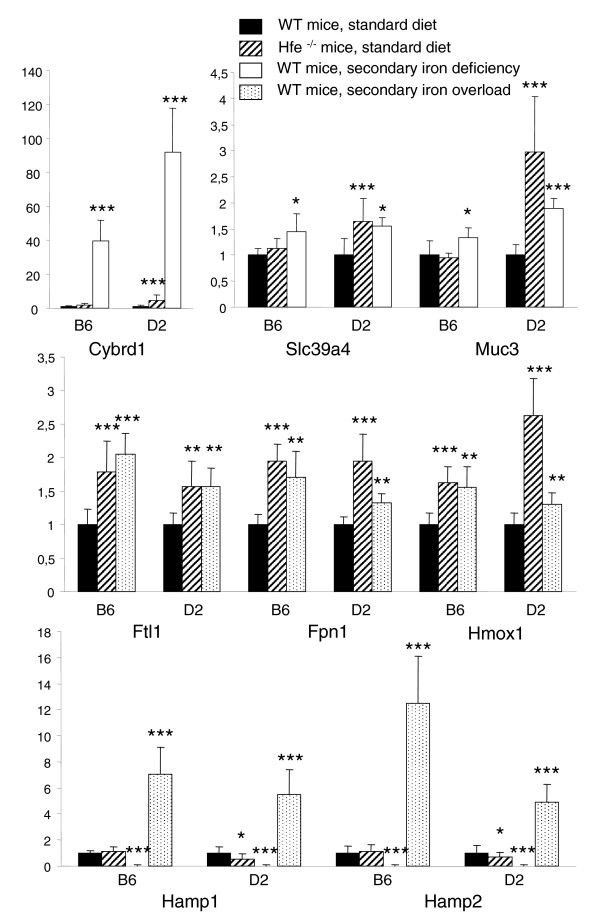
mRNA expression changes: *Hfe *disruption versus secondary iron deficiency or iron overload. shown is a comparison of mRNA expression changes induced by *Hfe *disruption with changes induced by secondary iron deficiency or iron overload within the B6 and D2 strains. Quantification of duodenal (*Cybrd1*, *Slc39a4*, and *Muc3*) or liver (*Ftl1*, *Fpn1*, *Hmox1*, *Hamp1*, and *Hamp2*) mRNAs was performed by quantitative real-time PCR on 7-week-old mice fed a diet containing 280 mg Fe/kg (wild-type [WT] controls and *Hfe*^-/- ^mice), an iron-deficient, or an iron-supplemented diet [40] for 3 weeks before they were killed. Expression values for each mouse were calculated as described in Materials and methods, and divided by the mean expression in control WT mice of the same genetic background. Error bars denote standard deviations. *P < 0.05, **P < 0.01, and ***P < 0.001.

Although it cannot be excluded that a slight upregulation of the *Cybrd1*, *Slc39a4*, and *Muc3 *messengers also exists in *Hfe*^-/- ^B6 mice but does not reach a level detectable by microarray or RT-PCR analysis, the differential expression of these genes between *Hfe*^-/- ^D2 and B6 mice does not appear to be related to the individual capacity of the two strains to respond to an iron-deficiency signal. Indeed, as shown in Figure 3, wild-type mice of both B6 and D2 genetic backgrounds fed an iron-deficient diet have induced duodenal expression of *Cybrd1*, *Slc39a4*, and *Muc3*, as compared with wild-type mice of the same genetic backgrounds fed a standard diet. Rather, the differences between *Hfe*^-/- ^D2 and B6 mice appear to be related to their varying capacity to perceive the iron-deficiency signal when Hfe is not functional. This probably explains the differences in extent of liver iron accumulation between the two strains.

As a result of *Hfe *deficiency, both strains accumulate iron, although the extent of iron overload is more severe in the D2 strain. This leads, in liver, to variations in expression of genes encoding glutathione synthetases, glutathione *S*-transferases, UDP-glucuronosyltransferases, vanin, ferroportin, the ferritin light chain, and heme oxygenase. These variations are encountered at a significant level more often in the liver of *Hfe*^-/- ^D2 mice than in that of B6 mice, which is consistent with the observation that *Hfe*^-/- ^D2 mice are more heavily iron loaded than *Hfe*^-/- ^B6 mice. Global expression profiling of *Hfe *wild-type mice of both strains fed an iron-supplemented diet for 3 weeks showed that they also had significant induction of several genes that are involved in the glutathione metabolism pathway or with UDP-glucuronosyltransferase activity (data not shown). In addition, these mice fed an iron-supplemented diet exhibited significant induction of *Ftl1*, *Fpn1*, and *Hmox1 *genes, as shown in Figure 3, which reinforces the hypothesis that these modifications are the consequence of iron overload and lipid peroxidation, and contribute to hepatoprotection [28].

Finally, as shown in Figure 3, only slight downregulation in levels of *Hamp1 *and *Hamp2 *was observed in *Hfe*^-/- ^D2 mice, and no significant variation was observed in *Hfe*^-/- ^B6 mice. These observations run counter to the marked induction of *Hamp1 *and *Hamp2 *expression by secondary iron overload, and virtually complete repression by secondary iron deficiency in wild-type mice of both B6 and D2 genetic backgrounds. In contrast to previous hypotheses regarding hepcidin regulation by Hfe, we speculate that hepcidin expression in *Hfe*-deficient mice might be subject to the counter-regulatory and conflicting influences of an inappropriate iron deficiency signal (which tends to downregulate hepcidin transcripts) and iron overload (which tends to upregulate them). This probably explains why, globally, the hepcidin transcripts are not largely altered by *Hfe *disruption, despite the excess iron accumulated by *Hfe*-deficient mice. This could also explain why young, 4-week-old *Hfe*^-/- ^mice exhibit reduced hepcidin expression, as compared with wild-type mice of the same genetic background [29], whereas this downregulation disappears in more severely iron loaded 8-week-old mice.

Notably, we observed enrichment of functional gene categories associated with lipid metabolism among genes that were downregulated in liver of *Hfe*^-/- ^mice. First, we noted an important downregulation of transcripts encoding key enzymes in the conversion of cholesterol to bile acids in *Hfe*^-/- ^D2 mice. Dietary iron overload in rats [30] was previously shown to affect the activity of key intracellular enzymes in cholesterol metabolism, in particular cholesterol 7α-hydroxylase (*Cyp7a1*), and was attributed to a marked membrane lipid peroxidation. The strain specificity of the downregulation of these transcripts may therefore be related to the variable iron accumulation observed in mice of the two genetic backgrounds. Cyp7a1 controls the main pathway whereby cholesterol is removed from the body in mammals. Thus, a decrease in cholesterol catabolism could lead to accumulation of plasma cholesterol and explain our previous observation that *Hfe*^-/- ^mice of the D2 genetic background have slightly higher plasma cholesterol levels than D2 wild-type mice (Table 7). Second, we observed striking and coordinated downregulation of multiple genes that regulate mitochondrial fatty acid β-oxidation in the *Hfe*^-/- ^mice of both strains, as well as variations in gene expression levels, suggesting that the flux through the TCA cycle is maintained at a low level to adapt to the downregulated β-oxidation in these *Hfe*^-/- ^mice. This suggests altered mitochondrial functioning induced by lack of Hfe, which warrants further investigation. Interestingly, the observed variations in the expression of genes encoding proteins involved in the mitochondrial iron or heme traffic, such as *Sfxn2*, *Slc25a37*, and *Abcg2*, are also compatible with the hypothesis that mitochondrial iron homeostasis is affected in *Hfe*^-/- ^mice.

**Table 7:** Effect of *Hfe *disruption on plasma lipid profiles

	C57BL/6 strain			DBA/2 strain		
	
	*Hfe*^-/-^	*Hfe*^+/+^	*P*	*Hfe*^-/-^	*Hfe*^+/+^	*P*
Total cholesterol (mg/ml)	0.93 ± 0.24	1.07 ± 0.05	0.28	1.46 ± 0.25	1.17 ± 0.13	0.04
HDL-cholesterol (mg/ml)	0.71 ± 0.18	0.86 ± 0.05	0.16	1.14 ± 0.11	0.97 ± 0.08	0.02

*Hfe*^-/- ^and *Hfe*^+/+ ^mice (five males per group) were killed at age 7 weeks. Blood was removed and plasma lipid levels were determined by chromatography. Results are expressed as mean ± standard deviation in each group. *P *values for comparisons of plasma lipid levels between Hfe^-/- ^and Hfe^+/+ ^mice of each strain were obtained by Student's *t*-test. HDL, high-density lipoprotein.

The reasons why *Hfe*-deficient mice incorrectly perceive the body's iron needs are still unknown, and one of our goals in this study was to identify gene expression changes that could help to elucidate why lack of functional Hfe leads to an inappropriate iron deficiency signal. Interestingly, we observed that the expression levels of several genes that participate in mitochondrial iron traffic and heme biosynthesis were altered in *Hfe*-deficient mice; in particular, the mRNA level of hepatic sideroflexin *Sfxn2 *was downregulated in both strains. Because of sequence and structural similarity to sideroflexin 1, sideroflexin 2 was suggested to be in the mitochondrion [31], and in a proteomic study [32] it was proved to be located in the mitochondrial inner membrane. Whether, like sideroflexin 1, sideroflexin 2 facilitates transport of pyridoxine or another Alas co-factor into the mitochondrion remains to be demonstrated. However, if this were the case, then *Hfe*^-/- ^mice with lower expression of *Sfxn2 *than wild-type mice would have reduced levels of Alas co-factor in the mitochondrion and have lower efficacy of heme biosynthesis, thus leading to the inappropriate iron-deficiency signal and the consequent upregulation of intestinal iron absorption. This would also be compatible with the paradoxic upregulation of δ-aminolevulinic acid synthase(*Alas2*), mitoferrin (*Slc25a37*; a mitochondrial iron importer essential for heme biosynthesis), and Bcrp (*Abcg2*; a possible mitochondrial heme exporter [24,33]) observed in these mice. Although this possible mechanism is still speculative, it would establish a link between Hfe and the mitochondrion in regulation of iron homeostasis. It is also consistent with recent studies suggesting that intermediates in heme metabolism, in particular levels of hepatic 5-amino-levulinate, regulate intestinal iron absorption [34-36].

Our expression studies also identified a large number of genes exhibiting differences in basal expression between strains or with regulation in response to *Hfe *disruption, and which reside within one of the five chromosomal regions harboring *Hfe*-modifier genes [10]. In order to relate these genomics findings to severity of hemochromatosis, we used the information available from WebQTL and found that several of these genes exhibited highly significant evidence for *cis *regulation. For example, expression profiling identified four genes residing in the critical region on chromosome 7, which were differentially expressed between B6 and D2 mice, and whose basal expression was linked to a chromosomal position coinciding with the gene itself. Among those, *Hamp *was also regulated by *Hfe *disruption in the liver of D2 mice. Previous studies have implicated *Hamp *in the severity of hemochromatosis [37,38], thus supporting recent suggestions that expression profiling can accelerate identification of genes that control complex traits [39]. Although none of the other *cis*-regulated genes has yet been implicated in iron metabolism, these genes are attractive candidate modifiers for phenotypic expression of hemochromatosis and warrant further investigation. Additional work is also needed to identify possible *trans *regulators in the chromosomal regions that harboring *Hfe*-modifier genes, because those could be candidate modifiers as well.

## Conclusion

In this study we investigated *Hfe *deficiency induced gene expression profiles in the liver and the duodenum of B6 and D2 mice, which are two inbred mouse strains with divergent iron loading severity in response to *Hfe *disruption. We identified organ-specific patterns of gene expression that contribute novel insight into the mechanisms of *Hfe *action in liver and duodenum. We also identified multiple genes with differential expression between wild-type or between *Hfe*-deficient strains, which had expression-regulating loci overlapping with disease modifier loci. Superimposing expression data and genetic data has thus yielded a testable set of hypotheses regarding genes related to iron loading severity and signaling events evoked by *Hfe *deficiency, with potential functional relevance to human hemochromatosis.

## Materials and methods

### Mice and tissue collection

Male *Hfe*^-/- ^(knockout) mice of the C57BL/6 (B6) and DBA/2 (D2) backgrounds were produced in the Institut Fédératif de Recherche (IFR) 30 animal facility [9]. Wild-type *Hfe*^+/+ ^controls (wild-type) of the same sex and genetic backgrounds were purchased from the Centre d'Elevage Robert Janvier (Le Genest St-Isle, France). The studied population consisted of 16 wild-type mice (eight B6 and eight D2) and 16 knockout mice (eight B6 and eight D2). Three mice in each of the four genotype/strain groups were used for genome-wide expression profiling, and five for validation of microarray results. Wild-type and knockout mice were housed in the IFR30 animal facility and had free access to water and R03 diet (UAR, Epinay-sur-Orge, France) containing 280 mg Fe/kg. All mice were analyzed at 7 weeks of age and fasted for 14 hours before they were killed. Experimental protocols were approved by the Midi-Pyrénées Animal Ethics Committee. Liver and duodenum were dissected for RNA isolation, rapidly frozen, and stored in liquid nitrogen. Nonheme iron was quantified as described previously [10]. Mean ± standard deviation iron concentrations were 304 ± 50, 456 ± 68, 946 ± 110, and 2,937 ± 282 μg/g dry weight in liver of B6 wild-type, D2 wild-type, B6 knockout, and D2 knockout mice, respectively. Mice fed an iron-deficient or an iron-supplemented diet were obtained as described previously [40]. Liver and duodenum samples were used to compare gene expression variations resulting from lack of functional *Hfe *with those induced by secondary iron deficiency or iron overload.

### RNA isolation, preparation of labeled cRNA, and microarray hybridization

Total RNA was extracted and purified using the RNeasy Lipid Tissue kit (Qiagen, Courtaboeuf, France). RNA quality was checked on RNA 6000 Nano chips using a Bioanalyzer 2100 (Agilent Technologies, Palo Alto, CA, USA). RNA samples used for chip experiments all had RNA Integrity Numbers [41] ranging from 9 to 10. Double-stranded cDNA and biotin-labeled cRNA were synthesized using the Affymetrix cDNA synthesis and IVT Labeling kits. Fragmented cRNAs (15 μg) were hybridized to 24 GeneChip^® ^Mouse Genome 430 2.0 arrays (Affymetrix, Santa Clara, CA, USA), in accordance with the standard protocol of the manufacturer. The arrays were scanned with a GeneChip^® ^Scanner 3000 (Affymetrix) and raw image files were converted to probe set data (*.CEL files), using the Affymetrix GeneChip^® ^Operating Software. Expression microarray data have been submitted to the National Center for Biotechnology Information's Gene Expression Omnibus repository (accession number Genbank: GSE7357).

### Microarray data analysis

All the analyses were performed using Bioconductor, an open source software for the analysis of genomic data rooted in the statistical computing environment R [42]. Arrays were normalized to have the same target mean intensity of 100. Quality control metrics were first obtained using the simpleaffy Bioconductor package [43]. Average background and the number of genes called present (42% to 48% in liver and 50% to 55% in duodenum) were similar across all chips. All arrays had a scale factor lower than 1.4-fold away from the average scale factor for all samples, a GAPDH (glyceraldehyde 3-phosphate dehydrogenase) 3':5' ratio at around 1 and a β-actin 3':5' ratio of under 2.2. Furthermore, plots of mean intensity per probe position averaged over all probe sets had very similar slopes for the different arrays, permitting valid comparisons within genes across arrays. Genes that were not reliably detected in at least three liver samples or three duodenum samples, in accordance with the Affymetrix detection call algorithm, were excluded from further analysis [44]. Of the 45,101 probe sets represented on the GeneChip^® ^Mouse Genome 430 2.0 arrays, 28,031 were retained for assessing changes in gene expression between groups of mice.

The S-score algorithm, available in the Bioconductor Sscore package [45], was applied to compare hybridization signals between two arrays. It uses the statistical power of all oligonucleotide pairs for a given gene and is thus particularly useful for studies having limited numbers of Affymetrix microarrays [46]. S-scores have a normal distribution with mean of 0 and standard deviation of 1, and are correlated with the fold change. Three types of comparisons were made: S-scores were calculated for D2 wild-type versus B6 wild-type samples within each organ to examine basal strain expression differences between D2 and B6 mice; S-scores were calculated for knockout versus wild-type samples within each organ and mouse strain to study responses to *Hfe *disruption; and control S-scores were calculated between biologic replicates within the different groups. To reduce the contribution of biologic and technical noise, S-scores were divided by the greater of 1 or the standard deviation of control S-scores within each organ. This general approach has been applied previously to microarrays [47] and reduces variance across experimental replicates [48]. Statistical analysis of microarrays (SAM) [49], a rank-based permutation method, was carried out to identify genes with S-scores significantly different from 0, using the R samr package. Genes regulated by *Hfe *deficiency were identified for each strain/organ combination by performing one-class SAM on knockout versus wild-type scores, using a false discovery rate of ≤10% to avoid eliminating genes that may be biologically important and increase our ability to populate functional networks of genes in subsequent bioinformatics studies. *Hfe*-regulated transcripts identified by SAM were filtered to count transcripts with an average S-score over three observations of ≥2 or ≤-2. Genes that exhibited both significant and reproducible changes were further analyzed for correlated gene expression patterns by application of k-means clustering, as described by Eisen and coworkers [50]. Genes differentially expressed between mice strains were identified by one-class SAM on wild-type D2 versus wild-type B6 S-scores, using a false discovery rate of ≤1%. This gene list was further filtered for an average S-score of ≥2.6 or ≤-2.6 over three observations.

### Bioinformatics analysis of gene expression patterns

DAVID (2007), a functional annotation tool [51,52], was used to identify enriched biologic themes and to discover function-related enriched gene groups among clusters, compared with all genes present on the Mouse Genome 430 2.0 array. The following annotation groupings were analyzed for overrepresentation in gene lists: the Protein Information Resource keywords, Kyoto Encyclopedia of Genes and Genomes and BioCarta pathways, and Gene Ontology biological processes and molecular functions. Results were filtered to remove categories with EASE (expression analysis systematic explorer) scores, based on a Fisher exact test, greater than 0.05. Redundant categories with the same gene members were removed to yield a single representative category. The chromosomal location of all genes exhibiting differential basal expression between strains or regulation by *Hfe *deficiency was superimposed on support intervals for hepatic iron loading modifiers on mouse chromosomes 3, 7, 8, 11, and 12 [10], and a list of differentially expressed genes mapping to these intervals was obtained. The WebQTL resource [53,54], which includes measures of mRNA expression in livers of 35 adult BXD recombinant inbred male mice obtained with Agilent G4121A microarrays, was used to link expression of the genes in this list to genetic markers and identify potential *cis*-regulators.

### Validation of microarray results by real-time PCR

All primers were designed using the Primer Express 2.0 software (Applied Biosystems, Foster City, CA, USA). Quantitative real-time PCR reactions were prepared with M-MLV reverse transcriptase (Promega, Charbonnières-les-Bains, France) and qPCR MasterMix Plus for SYBR^® ^Green (Eurogentec, Seraing, Belgium), as described previously [9], and run in duplicate. GenBank accession numbers, forward (F) and reverse (R) primers, and measured PCR efficiencies for the genes to be validated are given in Table 8. For each mouse, an expression measure was calculated as E_GoI_^Ct GoI^/E_HPRT_^Ct HPRT^, where GoI is the gene of interest; HPRT is a transcript with stable level between strains and genotypes, quantified to control for variation in cDNA amounts; E is the PCR reaction efficiency associated with either the gene of interest (E_GoI_) or the reference gene (E_HPRT_); and Ct is the cycle number at which fluorescence reaches a given threshold. Data were analyzed by one-factor (iron-deficient, standard, or iron-supplemented diet) or two-factor (chip/validation experiment and wild-type/knockout genotype) analysis of variance followed by Scheffe post-hoc tests using SAS software (version 9.1.3; SAS Institute Inc., Cary, NC, USA).

**Table 8:** Sequences of the primers used for validation of microarray results by real-time PCR

Gene	GeneBank accession	Forward primer	Reverse primer	Amplification efficiency
*Hprt*	NM_013556	5'-CTG GTT AAG CAG TAC AGC CCC AA-3'	5'-CAG GAG GTC CTT TTC ACC AGC-3'	1.99
*Aox1*	NM_009676	5'-CAC CCT GTA TTC ATC TAA GGG CCT-3'	5'-CAC TGC ATC ATG GAT GGC AA-3'	1.92
*Ftl1*	NM_01024	5'-GGA GAA GAA CCT GAA TCA GGC C-3'	5'-GGT TGC CCA TCT TCT TGA TGA G-3'	2.00
*Fpn1*	NM_016917	5'-CAT TGC TGC TAG AAT CGG TCT T-3'	5'-GCA ACT GTG TCA CCG TCA AAT-3'	1.97
*Hmox1*	NM_010442	5'-CAG ATG GCG TCA CTT CGT CA-3'	5'-CTC TGC AGG GGC AGT ATC TTG-3'	2.00
*Vanin1*	NM_011704	5'-GGC TGC ACA CCG TGG AAG-3'	5'-GGT AAA AGC CGT GTC CAC TGA A-3'	1.90
*Por*	NM_008898	5'-GCC TCG TCG TCT AAG GTC CA-3'	5'-GAC TTC GCT TCA TAC TCC ACA GC-3'	1.99
*Cpt1a*	NM_013495	5'-GAC CCC ACA ACA ACG GCA G-3'	5'-ATG GCG AGG CGG TAC AGG T-3'	2.00
*Aco2*	NM_080633	5'-GAC CAT TCA AGG CCT GAA GG-3'	5'-ACG CAC TTC AGA GGC TTT CC-3'	2.00
*Cyp7a1*	NM_007824	5'-GCT CTG GAG GGA ATG CCA T-3'	5'-CCG CAG AGC CTC CTT GAT G-3'	2.00
*Hsd3b5*	NM_008295	5'-AGA GGA ATT GTC CAA GCT GCA-3'	5'-TGT GGA TGA CAG CAG ACA TGC-3'	1.99
*Hfe2*	NM_027126	5'-ACC ACC ATC CGG AAG ATC ACT-3'	5'-AAG GCT GCA GGA AGA TTG TCC-3'	2.00
*Hamp1*	AF_503444	5'-AAG CAG GGC AGA CAT TGC GAT-3'	5'-CAG GAT GTG GCT CTA GGC TAT GT-3'	1.98
*Hamp2*	AY_232841	5'-AAG CAG GGC AGA CAT TGC GAT-3'	5'-GGA TGT GGC TCT AGG CTC TCT ATT-3'	2.00
*Usf2*	NM_011680	5'-ATG GAA CCA GAA CTC CTC GAG A-3'	5'-CCG TTC CAC TTC ATT GTG CTG-3'	1.93
*Lcn2*	NM_008491	5'-TCT GTC CCC ACC GAC CAA T-3'	5'-CCA GTC AGC CAC ACT CAC CAC-3'	1.99
*Sfxn2*	NM_053196	5'-CGC ACA AGT GGT TAT CTC TCG G-3'	5'-CCA TGA TGA CAG GCA ACA GGA-3'	1.99
*Alas2*	NM_009653	5'-TGG AAC TCT TGG CAA GGC C-3'	5'-CAA GTC CCG AGT GCT GGC T-3'	1.99
*Slc25a37*	NM_026331	5'-GAG CAC TCC ATC ATG TAC CCG-3'	5'-TGG ATT CAA ACT CTG CAT CCG-3'	2.00
*Abcg2*	NM_011920	5'-TTG GCT GTC CTG GCT TCA GTA C-3'	5'-CAA AGC TGT GAA GCC ATA TCG A-3'	1.99
*Cybrd1*	AF_354666	5'-GCA GCG GGC TCG AGT TTA-3'	5'-TTC CAG GTC CAT GGC AGT CT-3'	1.98
*Slc39a4*	NM_028064	5'-GCG ACT GAG AGC AGA GCT GA-3'	5'-GTT GTG TAC CGC GTC GCC-3'	2.00
*Mucin3*	NM_355711	5'-TCG TGT TCT CCA TCC GCT TC-3'	5'-GAC ACT CTG GAC CGT TGC CT-3'	1.99
*Lcn13*	NM_153558	5'-TGT TTG TGC CAG AGA TCG GAG-3'	5'-GCT GGC TCA GCT GTT GCA G-3'	1.95
*Fmo3*	NM_008030	5'-GGA ACT TGC ACT TTG CCT TCT G-3'	5'-GGT GGT GCT ATT GCC ATA CCA-3'	1.96
*Clca4*	NM_139148	5'-GCC GTC ATA GAA GCT GAG AGT GG-3'	5'-AGC ACC TGC CCC GTT GTC-3'	2.00

*Hfe*^-/- ^and *Hfe*^+/+ ^mice (five males per group) were killed at age 7 weeks. Blood was removed and plasma lipid levels were determined by chromatography. Results are expressed as mean ± standard deviation in each group. *P *values for comparisons of plasma lipid levels between Hfe^-/- ^and Hfe^+/+ ^mice of each strain were obtained by Student's *t*-test. HDL, high-density lipoprotein.

## Abbreviations

DAVID, Database for Annotation, Visualization, and Integrated Discovery; HFE, hereditary hemochromatosis protein; HH, hereditary hemochromatosis; RT-PCR, reverse transcription polymerase chain reaction; SAM, statistical analysis of microarrays; TCA, tricarboxylic acid.

## Authors' contributions

HC and MPR designed the experiments, participated in their execution, analyzed the data, and wrote the manuscript. VD, LK, and DM assisted with the execution of the experiments. MA and JM provided conceptual expertise for functional annotation, and MM for statistical analysis. All authors read and approved the final version of the manuscript.

## Additional data files

The following additional data are available with the online version of this paper. Additional data file 1 lists genes significantly regulated by *Hfe *disruption in the liver of D2 or B6 mice, according to microarray analysis. Additional data file 2 lists genes significantly regulated by *Hfe *disruption in duodenum of D2 or B6 mice. Additional data file 3 lists genes differentially expressed in the liver or the duodenum of wild-type D2 and B6 mice.

## Supplementary Material

Additional File 1 Presented is a table listing genes significantly regulated by *Hfe *disruption in the liver of D2 or B6 mice according to microarray analysis. Click here for file

Additional File 2 Presented is a table listing genes significantly regulated by *Hfe *disruption in the duodenum of D2 or B6 mice. Click here for file

Additional File 3 Presented is a table listing genes differentially expressed in the liver and/or the duodenum of wild-type D2 and B6 mice. Click here for file
