# Supplementary material for: Gene expression profiling of Hfe-/- liver and duodenum in mouse strains with differing susceptibilities to iron loading: identification of transcriptional regulatory targets of Hfe and potential hemochromatosis modifiers
Source: Genome Biol. 2007 Oct 18;8(10):R221. doi: 10.1186/gb-2007-8-10-r221 (PMC2246295; doi:10.1186/gb-2007-8-10-r221)
Supplement: Additional File 1 — Presented is a table listing genes significantly regulated by Hfe disruption in the liver of D2 or B6 mice according to microarray analysis. [file gb-2007-8-10-r221-S1.pdf]

Additional data file 1. Genes regulated by Hfe disruption in D2 and/or B6 liver.

Cluster		ProbesetID	Genbank	Gene symbol	Description	D2KOvsD2WT	B6KOvsB6WT	D2WTvsB6WT	chromosome	position (kb)
1	A	1417764_at	BG077348	Ssr1	signal sequence receptor, alpha	8,22	1,01	-0,18	13	37 985 475
1	A	1449848_at	NM_008137	Gna14	guanine nucleotide binding protein, alpha 14	4,64	0,31	-0,08	19	16 502 780
1	A	1449816_at	NM_020564	Sult5a1	sulfotransferase family 5A, member 1	4,05	0,58	-0,40	8	126 028 936
1	A	1453582_at	BB129366	Chka	choline kinase alpha	4,17	0,80	-1,21	19	3 851 772
1	A	1427512_a_at	X84014	Lama3	laminin, alpha 3	4,09	-0,13	-0,37	18	
1	A	1460593_at	BF455403	Susd4	sushi domain containing 4	4,03	1,73	-0,33	1	184 601 582
1	A	1442277_at	BB546429	Chka	choline kinase alpha	3,43	0,36	-0,98	19	3 851 772
1	A	1428509_at	AK018649	Myo1e	myosin IE	2,80	0,08	-0,65	9	70 006 513
1	A	1424934_at	BC027200	Ugt2b1	UDP glucuronosyltransferase 2 family, polypeptide B1	3,72	0,69	-0,19	5	87 991 199
1	A	1425898_x_at	AF442824	Olfm3	olfactomedin 3	3,52	-0,03	0,24	3	115 072 968
1	A	1417580_s_at	NM_019414	Selenbp2	selenium binding protein 2	4,83	0,67	-1,15	3	94 778 976
1	A	1434135_at	BB107552	B3galnt2	UDP-GalNAc:betaGlcNAc beta 1,3-galactosaminyltransferase, polypeptide 2	2,43	0,24	-0,19	13	13 746 799
1	A	1436454_x_at	BB393998	Fen1	flap structure specific endonuclease 1	2,43	0,36	0,41	19	10 266 183
1	A	1426406_at	BB787289	Setd8	SET domain containing (lysine methyltransferase) 8	2,59	1,16	0,93	5	124 700 547
1	A	1440691_at	AV373767	Cyp2j6	cytochrome P450, family 2, subfamily j, polypeptide 6	2,70	-0,31	0,27	4	96 009 259
1	A	1451114_at	BC027248	Cmtm6	CKLF-like MARVEL transmembrane domain containing 6	2,20	1,09	0,65	9	114 579 900
1	A	1454885_at	BM211194	BC063263	cDNA sequence BC063263	2,21	0,77	-1,12	7	42 246 539
1	A	1423117_at	BB837171	Pum1	pumilio 1 (Drosophila)	2,40	0,79	0,38	4	129 935 464
1	A	1454858_x_at	AV171622	Mettl7a	methyltransferase like 7A	3,26	1,46	-0,74	15	100 132 850
1	A	1459314_at	BB229373	Cdkal1	CDK5 regulatory subunit associated protein 1-like 1	2,95	-0,16	-0,22	13	29 332 772
1	A	1424905_a_at	BC019647	Slc39a11	solute carrier family 39 (metal ion transporter), member 11	2,76	1,22	0,90	11	113 060 944
1	A	1429549_at	BB044654	Col27a1	procollagen, type XXVII, alpha 1	3,32	0,81	0,47	4	62 702 186
1	A	1433733_a_at	BG069864	Cry1	cryptochrome 1 (photolyase-like)	3,17	1,63	-0,97	10	84 561 499
1	A	1434230_at	BG094331	Polb	polymerase (DNA directed), beta	2,84	0,02	0,85	8	24 093 810
1	A	1434151_at	AV171622	Mettl7a	methyltransferase like 7A	2,93	0,85	-0,84	15	100 132 850
1	A	1428935_at	AI988026	Canx	calnexin	2,76	-0,70	-0,36	11	50 137 890
1	A	1450985_a_at	NM_011597	Tjp2	tight junction protein 2	2,72	-0,16	-0,81	19	24 161 607
1	A	1416698_a_at	NM_016904	Cks1b	CDC28 protein kinase 1b	2,35	1,37	0,17	3	89 501 400
1	A	1425127_at	BC026757	Hsd3b2	hydroxy-delta-5-steroid dehydrogenase, 3 beta- and steroid delta-isomerase 2	2,41	0,37	0,45	3	98 838 322
1	A	1427248_at	BG075255	Whsc2	Wolf-Hirschhorn syndrome candidate 2 (human)	2,33	-0,10	-0,24	5	34 215 038
1	A	1444960_at	BB228423	Cyp2u1	cytochrome P450, family 2, subfamily u, polypeptide 1	3,26	1,06	0,70	3	131 283 564
1	A	1438790_x_at	AA589282	Tmem41b	transmembrane protein 41B	2,78	1,00	-0,56	7	109 763 363
1	A	1442258_at	AV229709	1300002A08Rik	RIKEN cDNA 1300002A08 gene	2,46	0,52	0,33	8	89 514 211
1	A	1451300_a_at	BC024115	Chmp7	CHMP family, member 7	2,43	1,53	0,38	14	68 452 080
1	A	1417763_at	BG077348	Ssr1	signal sequence receptor, alpha	2,62	1,20	0,09	13	37 985 475
1	A	1440846_at	BB430553	A930041I02Rik	RIKEN cDNA A930041I02 gene	2,06	-0,41	-0,34	2	38 888 223
1	A	1438218_at	BM232143	Zbtb24	zinc finger and BTB domain containing 24	2,16	0,72	-0,08	10	41 138 810
1	A	1428669_at	AK012810	Bmyc	brain expressed myelocytomatosis oncogene	2,96	-0,32	0,10	2	25 528 887
1	A	1422053_at	NM_008380	Inhba	inhibin beta-A	3,38	0,08	0,85	13	15 805 361
1	A	1422902_s_at	NM_023799	Mgea5	meningioma expressed antigen 5 (hyaluronidase)	2,23	1,06	-0,29	19	45 803 571
1	A	1442340_x_at	BB533736	Cyr61	cysteine rich protein 61	2,46	-1,10	0,58	3	145 584 362
1	A	1436181_at	AV077160	Itgb1bp1	integrin beta 1 binding protein 1	3,93	0,73	-1,02	12	21 516 295
1	A	1421258_a_at	NM_013631	Pklr	pyruvate kinase liver and red blood cell	3,56	1,03	0,83	3	89 222 068
1	A	1453500_at	AK018458	Cyp2u1	cytochrome P450, family 2, subfamily u, polypeptide 1	3,50	1,29	0,35	3	131 283 564
1	A	1417850_at	NM_009029	Rb1	retinoblastoma 1	2,88	1,08	-0,51	14	71 929 656
1	A	1452090_a_at	BB667895	Olfm3	olfactomedin 3	2,18	0,09	0,30	3	115 072 968
1	A	1455915_at	BF583094	Galnt4	UDP-N-acetyl-alpha-D-galactosamine:polypeptide N-acetylgalactosaminyltransferase 4	2,40	0,18	1,03	10	98 537 825
1	A	1456190_a_at	BB745805	Acsm2	acyl-CoA synthetase medium-chain family member 2	2,39	0,31	-0,23	7	119 352 859
1	A	1423648_at	BC006865	Pdia6	protein disulfide isomerase associated 6	2,48	0,00	-0,67	12	17 292 074
1	A	1426614_at	BB523906	Prkcbp1	protein kinase C binding protein 1	2,63	0,82	-0,97	2	165 475 359
1	A	1418668_at	NM_054094	Acsm1	acyl-CoA synthetase medium-chain family member 1	3,15	1,07	-1,29	7	119 408 976
1	A	1445824_at	AV276001	Zfp458	zinc finger protein 458	2,25	-0,28	-0,27	13	67 754 015
1	A	1448100_at	NM_133797	4833439L19Rik	RIKEN cDNA 4833439L19 gene	2,04	1,25	-1,07	13	54 560 846
1	A	1427923_at	BM233793	Zmpste24	zinc metallopeptidase, STE24 homolog (S. cerevisiae)	2,33	0,68	0,24	4	120 556 872
1	A	1450911_at	BI525209	Ppib	peptidylprolyl isomerase B	3,10	1,34	-0,33	9	65 858 174

1	A	1434229_a_at	BG094331	Polb	polymerase (DNA directed), beta	2,60	0,41	-0,03	8	24 093 810
1	A	1417773_at	BI151886	Nans	N-acetylneuraminic acid synthase (sialic acid synthase)	2,85	1,02	0,14	4	46 510 428
1	A	1453746_at	AI606033	Fnbp1	formin binding protein 1	2,38	0,74	-1,19	2	30 848 215
1	A	1438418_at	BB536931	4932432K03Rik	RIKEN cDNA 4932432K03 gene	2,61	0,54	0,42	14	47 988 499
1	A	1456029_a_at	AW543761	Ttc7b	tetratricopeptide repeat domain 7B	2,38	0,76	-0,05	12	100 701 820
1	A	1437345_a_at	BB223872	Bscl2	Bernardinelli-Seip congenital lipodystrophy 2 homolog (human)	2,68	1,36	-1,20	19	8 906 547
1	A	1418923_at	NM_134069	Slc17a3	solute carrier family 17 (sodium phosphate), member 3	2,20	1,02	0,28	13	23 847 093
1	A	1445377_at	BB443911	Als2cr19	amyotrophic lateral sclerosis 2 (juvenile) chromosome region, candidate 19 (human)	2,30	-0,24	-0,44	1	
1	A	1423333_at	AK003239	1200007D18Rik	RIKEN cDNA 1200007D18 gene	2,48	0,78	-0,77	17	26 289 111
1	A	1424854_at	BC019757	Hist1h4i	histone 1, H4i	2,60	1,27	0,69	13	22 048 280
1	A	1419161_a_at	AB041034	Nox4	NADPH oxidase 4	2,24	0,67	-0,96	7	87 122 555
1	A	1438561_x_at	BB400326	4930538D17Rik	RIKEN cDNA 4930538D17 gene	2,35	-0,29	-0,29	19	46 410 245
1	A	1448685_at	NM_026063	2900010M23Rik	RIKEN cDNA 2900010M23 gene	2,45	1,28	-0,81	17	26 850 253
1	A	1428445_at	AK020444	Yif1b	Yip1 interacting factor homolog B (S. cerevisiae)	3,86	0,76	-0,25	7	28 947 112
1	A	1423695_at	BC008268	Edem2	ER degradation enhancer, mannosidase alpha-like 2	3,18	1,34	-1,06	2	155 393 468
1	A	1457619_at	BB743970	Ces6	carboxylesterase 6	2,20	1,16	-0,88	8	107 623 136
1	A	1438038_at	C78926	Psmd11	proteasome (prosome, macropain) 26S subunit, non-ATPase, 11	2,32	0,74	0,14	11	80 244 822
1	A	1425077_at	AI606403	Dnajc18	DnaJ (Hsp40) homolog, subfamily C, member 18	2,10	0,37	-0,61	18	35 799 800
1	A	1449351_s_at	NM_019971	Pdgfc	platelet-derived growth factor, C polypeptide	2,28	-0,44	0,96	3	81 122 342
1	A	1450332_s_at	NM_010232	Fmo5	flavin containing monooxygenase 5	2,12	0,83	-0,70	3	97 714 279
1	A	1419622_at	NM_009467	Ugt2b5	UDP glucuronosyltransferase 2 family, polypeptide B5	2,17	0,97	-0,98	5	88 199 520
1	A	1418013_at	NM_023160	Cml1	camello-like 1	3,06	0,70	0,29	6	85 875 790
1	A	1437667_a_at	AW553304	Bach2	BTB and CNC homology 2	2,60	0,49	0,82	4	32 567 562
1	A	1437908_a_at	BB095626	1200007D18Rik	RIKEN cDNA 1200007D18 gene	2,64	1,35	-0,37	17	26 289 111
1	A	1417070_at	NM_133969	Cyp4v3	cytochrome P450, family 4, subfamily v, polypeptide 3	2,04	0,02	-0,74	8	46 804 618
1	A	1428671_at	AK008617	2200002D01Rik	RIKEN cDNA 2200002D01 gene	2,91	0,26	-0,18	7	28 956 043
1	A	1447563_at	AV169767	Rps3	ribosomal protein S3	2,16	-0,32	-0,34	7	99 353 009
1	A	1418963_at	NM_024185	2310047O13Rik	RIKEN cDNA 2310047O13 gene	2,49	0,87	-0,94	2	12 265 116
1	A	1415827_a_at	BF658806	D3Ucla1	DNA segment, Chr 3, University of California at Los Angeles 1	2,25	1,26	-0,69	3	58 609 906
1	A	1452274_at	BG976649	Zfand3	zinc finger, AN1-type domain 3	2,01	0,93	0,51	17	29 732 098
1	A	1437649_x_at	AV051525	Snx22	sorting nexin 22	2,72	1,25	-1,09	9	65 863 183
1	A	1449459_s_at	AF403041	Asb13	ankyrin repeat and SOCS box-containing protein 13	2,26	0,88	0,94	13	3 633 323
1	A	1421259_at	NM_013631	Pklr	pyruvate kinase liver and red blood cell	2,76	-0,57	-0,12	3	89 222 068
1	A	1416712_at	NM_008820	Pepd	peptidase D	2,02	0,49	0,99	7	34 621 166
1	A	1432492_a_at	AK002295	Haao	3-hydroxyanthranilate 3,4-dioxygenase	2,06	0,72	0,04	17	
1	A	1434087_at	BG069750	Mthfr	5,10-methylenetetrahydrofolate reductase	2,52	1,76	-1,02	4	146 885 021
1	A	1422973_a_at	NM_009381	Thrsp	thyroid hormone responsive SPOT14 homolog (Rattus)	2,78	0,77	-0,75	7	97 288 150
1	A	1417962_s_at	NM_010284	Ghr	growth hormone receptor	2,22	0,34	0,10	15	3 267 776
1	A	1445677_x_at	BB401264	Slc35f2	solute carrier family 35, member F2	2,06	0,96	1,03	9	53 569 674
1	A	1439030_at	BI410722	Gmppb	GDP-mannose pyrophosphorylase B	2,18	0,63	-0,57	9	107 907 390
1	A	1427302_at	AV224446	Enpp3	ectonucleotide pyrophosphatase/phosphodiesterase 3	2,32	0,76	-0,94	10	24 463 318
1	A	1453685_at	AK008824	Nudt7	nudix (nucleoside diphosphate linked moiety X)-type motif 7	2,49	1,03	0,21	8	117 019 591
1	A	1448852_at	NM_009060	Rgn	regucalcin	2,21	1,09	0,15	X	19 706 776
1	A	1421321_a_at	NM_019671	Net1	neuroepithelial cell transforming gene 1	2,48	0,67	-0,38	13	3 882 581
1	A	1424743_at	BC010800	2610003J06Rik	RIKEN cDNA 2610003J06 gene	2,62	0,14	-0,92	17	25 556 646
1	A	1418932_at	AY061760	Nfil3	nuclear factor, interleukin 3, regulated	2,14	0,54	-0,35	13	52 979 641
1	A	1437119_at	BG075179	Ern1	endoplasmic reticulum (ER) to nucleus signalling 1	2,01	1,24	0,20	11	106 213 709
1	A	1425107_a_at	D17444	Lifr	leukemia inhibitory factor receptor	2,49	0,62	-0,36	15	7 087 755
1	A	1452353_at	BB762731	Gpr155	G protein-coupled receptor 155	2,21	0,47	-0,21	2	
1	A	1420385_at	NM_008137	Gna14	guanine nucleotide binding protein, alpha 14	2,83	0,54	0,70	19	16 502 780
1	A	1450264_a_at	NM_013490	Chka	choline kinase alpha	2,02	0,40	0,58	19	3 851 772
1	A	1419954_s_at	AW539211	Zfand3	zinc finger, AN1-type domain 3	2,03	0,59	0,48	17	29 732 098
1	A	1431354_a_at	AK020164	Fars2	phenylalanine-tRNA synthetase 2 (mitochondrial)	2,01	-0,23	0,03	13	36 124 875
1	A	1455987_at	BF148622	Sec61a1	Sec61 alpha 1 subunit (S. cerevisiae)	2,17	1,10	-0,32	6	88 469 247
1	A	1450725_s_at	NM_011797	Car14	carbonic anhydrase 14	2,33	1,06	0,03	3	95 983 205
1	A	1448844_at	NM_025558	Cyb5b	cytochrome b5 type B	2,03	1,51	0,68	8	110 039 789
1	A	1434330_at	BB540721	Lrrc35	leucine rich repeat containing 35	2,33	1,62	0,13	9	42 163 313
1	A	1434267_at	BG069735	Nek1	NIMA (never in mitosis gene a)-related expressed kinase 1	2,30	0,49	-0,54	8	64 022 716
1	A	1416130_at	BE630020	Prnp	prion protein	2,35	-0,43	0,36	2	131 601 397
1	A	1425129_a_at	BC004754	Taldo1	transaldolase 1	2,29	0,57	-1,00	7	141 243 536

1	A	1417067_s_at	AK014605	Cabc1	chaperone, ABC1 activity of bc1 complex like (S. pombe)	2,45	0,30	-0,88	1	182 001 814
1	A	1448948_at	NM_009057	Rag1ap1	recombination activating gene 1 activating protein 1	2,08	-0,97	-0,10	3	89 354 172
1	A	1453732_at	BF533688	6230416A05Rik	RIKEN cDNA 6230416A05 gene	2,14	1,18	0,43	13	
1	A	1416479_a_at	NM_025387	Tmem14c	transmembrane protein 14C	2,05	1,16	0,40	13	41 027 265
1	A	1426812_a_at	BF166604	9130404D14Rik	RIKEN cDNA 9130404D14 gene	2,14	-0,19	0,34	2	32 698 232
1	A	1451788_at	AF356627	F11	coagulation factor XI	2,08	-0,98	0,13	8	46 739 986
1	A	1456181_at	BG069289	9530020G05Rik	RIKEN cDNA 9530020G05 gene	2,09	0,55	0,06	6	34 810 578
1	A	1416403_at	AV382118	Abcb10	ATP-binding cassette, sub-family B (MDR/TAP), member 10	2,45	0,28	-0,57	8	126 838 550
1	A	1434923_at	AV354536	2810437L13Rik	RIKEN cDNA 2810437L13 gene	2,06	1,47	0,80	5	139 591 463
1	A	1459242_at	AW556311	Elovl5	ELOVL family member 5, elongation of long chain fatty acids (yeast)	2,84	-1,37	0,44	9	77 703 051
1	A	1434520_at	AU067703	Sc5d	sterol-C5-desaturase (fungal ERG3, delta-5-desaturase) homolog (S. cerevisae)	2,96	-0,37	1,00	9	42 005 171
1	A	1424026_s_at	BC013529	BC013529	cDNA sequence BC013529	2,42	0,03	0,30	10	7 458 143
1	A	1439846_at	BM249597	Klf12	Kruppel-like factor 12	2,18	-0,72	-0,29	14	98 753 776
1	A	1438320_s_at	BB464359	Mcm7	minichromosome maintenance deficient 7 (S. cerevisiae)	2,13	1,27	0,21	5	138 394 376
1	A	1453706_at	AV152654	Wwox	WW domain-containing oxidoreductase	2,08	-0,24	-0,33	8	117 325 672
1	A	1422643_at	NM_021509	Moxd1	monooxygenase, DBH-like 1	5,49	-0,16	-0,40	10	23 912 932
1	A	1417203_at	BC010592	Ethe1	ethylmalonic encephalopathy 1	2,56	1,97	1,13	7	24 296 302
1	A	1425094_a_at	AB031040	Lhx6	LIM homeobox protein 6	2,10	0,66	0,36	2	35 905 550
1	A	1434238_at	BQ176372	Taf2	TAF2 RNA polymerase II, TATA box binding protein (TBP)-associated factor	2,07	1,34	-0,58	15	
1	A	1451297_at	BC019856	Gulo	gulonolactone (L-) oxidase	2,45	-0,02	-0,29	14	64 940 896
1	A	1422103_a_at	BC024319	Stat5b	signal transducer and activator of transcription 5B	2,04	-0,67	0,19	11	100 596 822
1	B	1428851_at	AK005003	1300014I06Rik	RIKEN cDNA 1300014I06 gene	7,72	0,03	-5,50	13	34 635 304
1	B	1421092_at	AK014346	Serpina12	serine (or cysteine) peptidase inhibitor, clade A (alpha-1 antiproteinase, antitrypsin), member 12	6,91	-1,32	-5,35	12	104 429 818
1	B	1438294_at	BG067317	Atxn1	ataxin 1	7,51	-1,06	-7,11	13	45 565 864
1	B	1437892_at	BQ084812	Zfp306	zinc finger protein 306	5,74	-0,57	-4,70	13	21 394 560
1	B	1423397_at	AI118428	Ugt2b36	UDP glucuronosyltransferase 2 family, polypeptide B36	4,66	-1,34	-4,89	5	88 140 487
1	B	1449844_at	AB031813	Slco1a1	solute carrier organic anion transporter family, member 1a1	5,32	-1,72	-3,94	6	141 870 258
1	B	1418858_at	NM_023617	Aox3	aldehyde oxidase 3	5,31	0,68	-7,09	1	58 057 681
1	B	1424576_s_at	BC025819	Cyp2c44	cytochrome P450, family 2, subfamily c, polypeptide 44	3,07	0,16	-5,69	19	44 058 336
1	B	1419407_at	NM_010406	Hc	hemolytic complement	2,45	0,65	-7,59	2	34 805 339
1	B	1454777_at	BB553107	Slco2b1	solute carrier organic anion transporter family, member 2b1	2,68	-1,51	-4,87	7	99 531 886
1	B	1448261_at	NM_009864	Cdh1	cadherin 1	4,53	0,15	-3,95	8	109 492 496
1	B	1424886_at	BC025145	Ptprd	protein tyrosine phosphatase, receptor type, D	2,37	0,06	-3,90	4	75 412 469
1	B	1433933_s_at	BB553107	Slco2b1	solute carrier organic anion transporter family, member 2b1	2,58	-1,46	-5,11	7	99 531 886
1	B	1449419_at	NM_028785	Dock8	dedicator of cytokinesis 8	2,20	-1,68	-4,69	19	25 066 625
1	C	1424221_at	BC021842	Susd4	sushi domain containing 4	6,74	1,19	-2,19	1	184 601 582
1	C	1424245_at	BC015290	Ces2	carboxylesterase 2	3,98	1,37	-2,94	8	107 736 214
1	C	1450352_at	NM_008639	Mtnr1a	melatonin receptor 1A	4,23	-1,70	-0,82	8	46 568 026
1	C	1451460_a_at	BC026598	Slc22a7	solute carrier family 22 (organic anion transporter), member 7	4,03	-0,93	-0,29	17	45 895 373
1	C	1420531_at	NM_008295	Hsd3b5	hydroxy-delta-5-steroid dehydrogenase, 3 beta- and steroid delta-isomerase 5	2,78	0,32	-1,31	3	98 747 696
1	C	1421074_at	NM_007825	Cyp7b1	cytochrome P450, family 7, subfamily b, polypeptide 1	2,95	-0,46	-2,41	3	18 264 100
1	C	1421075_s_at	NM_007825	Cyp7b1	cytochrome P450, family 7, subfamily b, polypeptide 1	2,89	-0,45	-2,21	3	18 264 100
1	C	1443138_at	BB541054	Sult5a1	sulfotransferase family 5A, member 1	3,69	-1,64	-0,30	8	126 028 936
1	C	1448769_at	NM_016752	Slc35b1	solute carrier family 35, member B1	3,14	0,33	-1,82	11	95 201 011
1	C	1423090_x_at	AV216331	Slc37a2	solute carrier family 37 (glycerol-3-phosphate transporter), member 2	4,99	1,51	-2,29	9	36 978 819
1	C	1420420_at	NM_010403	Hao1	hydroxyacid oxidase 1, liver	3,59	0,65	-1,45	2	134 188 802
1	C	1443263_at	AV365508	Bach2	BTB and CNC homology 2	3,14	-1,17	-0,89	4	32 567 562
1	C	1431406_at	BC022644	Agxt2l1	alanine-glyoxylate aminotransferase 2-like 1	3,07	0,59	-2,50	3	130 606 729
1	C	1450699_at	NM_009150	Selenbp1	selenium binding protein 1	6,78	0,13	-1,15	3	95 018 486
1	C	1422906_at	NM_011920	Abcg2	ATP-binding cassette, sub-family G (WHITE), member 2	2,97	0,35	-1,36	6	58 526 249
1	C	1426223_at	BC020021	2810439F02Rik	RIKEN cDNA 2810439F02 gene	4,04	-1,69	-2,87	18	12 786 968
1	C	1451499_at	AF000969	Cadps2	Ca2+-dependent activator protein for secretion 2	2,76	0,56	-2,04	6	23 212 838
1	C	1423151_at	AK010861	Dnajb11	DnaJ (Hsp40) homolog, subfamily B, member 11	3,17	-0,59	-1,38	16	22 773 199
1	C	1424259_at	BC020104	2400010G15Rik	RIKEN cDNA 2400010G15 gene	2,90	-0,50	-1,57	17	25 306 788
1	C	1456170_x_at	AW544960	Calr	calreticulin	2,12	-1,18	-0,72	8	87 731 954
1	C	1421184_a_at	NM_027334	Mettl7a	methyltransferase like 7A	2,25	0,32	-1,22	15	100 132 850
1	C	1442537_at	BB771206			2,99	-0,09	-2,21	3	131 277 598
1	C	1455697_at	BQ175938	LOC629059		2,69	-0,23	-2,18	14	
1	C	1452540_a_at	M25487	Hist1h2bp	histone 1, H2bp	2,79	-1,90	-1,92	13	21 794 979

1	C	1417606_a_at	NM_007591	Calr	calreticulin	2,19	-0,87	-0,93	8	87 731 954
1	C	1449308_at	NM_016704	C6	complement component 6	2,52	-0,21	-1,21	15	4 677 211
1	C	1416488_at	U95826	Ccng2	cyclin G2	2,03	-0,43	-2,55	5	94 342 460
1	C	1417083_at	NM_024171	Sec61b	Sec61 beta subunit	4,08	1,09	-1,66	4	47 495 760
1	C	1435101_at	BB389641	Derl2	Der1-like domain family, member 2	2,71	1,43	-3,00	11	70 823 639
1	C	1441380_at	BB044461	2810439F02Rik	RIKEN cDNA 2810439F02 gene	2,55	-1,68	-1,53	18	12 786 968
1	C	1417365_a_at	AU079514	Calm1	calmodulin 1	2,22	-0,87	-1,40	12	100 600 590
1	C	1460742_at	BI144423	Alg3	asparagine-linked glycosylation 3 homolog (yeast, alpha-1,3-mannosyltransferase)	2,43	0,17	-1,45	16	20 519 015
1	C	1419520_at	NM_023455	Cml4	camello-like 4	2,55	0,82	-2,27	6	85 796 045
1	C	1429720_at	AK021042	Mak10	MAK10 homolog, amino-acid N-acetyltransferase subunit, (S. cerevisiae)	2,98	-0,79	-2,95	13	59 595 033
1	C	1450011_at	AK012103	Hsd17b12	hydroxysteroid (17-beta) dehydrogenase 12	2,26	-0,04	-1,94	2	93 833 537
1	C	1459301_at	AW546127	Mrg1	myeloid ecotropic viral integration site-related gene 1	2,37	-0,49	-0,77	2	115 554 509
1	C	1460232_s_at	NM_013821	Hsd3b6	hydroxy-delta-5-steroid dehydrogenase, 3 beta- and steroid delta-isomerase 6	2,80	-0,44	-1,06	3	98 934 565
1	C	1438699_at	AA530749	Srd5a1	steroid 5 alpha-reductase 1	2,45	-1,97	-1,50	13	70 040 821
1	C	1460174_at	NM_021428	Dexi	dexamethasone-induced transcript	3,41	-0,88	-0,89	16	10 443 785
1	C	1420379_at	AB031813	Slco1a1	solute carrier organic anion transporter family, member 1a1	2,63	-0,65	-1,68	6	141 870 258
1	C	1421214_at	NM_007717	Cmah	cytidine monophospho-N-acetylneuraminic acid hydroxylase	2,33	-0,92	-2,53	13	24 422 586
1	C	1451131_at	AF133669	Arl6ip1	ADP-ribosylation factor-like 6 interacting protein 1	2,34	-0,03	-1,68	7	117 910 048
1	C	1416381_a_at	BC008174	Prdx5	peroxiredoxin 5	2,64	-0,17	-3,11	19	6 973 862
1	C	1432282_a_at	AK008522	2010305C02Rik	RIKEN cDNA 2010305C02 gene	2,98	-1,14	-1,59	11	75 284 273
1	C	1423732_at	BC012401	Tram1	translocating chain-associating membrane protein 1	2,38	0,62	-2,11	1	13 549 915
1	C	1425305_at	BC027135	Zfp295	zinc finger protein 295	2,03	-0,23	-1,22	16	98 103 855
1	C	1455293_at	BG065311	Leo1	Leo1, Paf1/RNA polymerase II complex component, homolog (S. cerevisiae)	2,00	0,40	-2,02	9	75 227 432
1	C	1439257_x_at	BB519934	Rpn1	ribophorin I	2,36	0,22	-1,31	6	88 050 156
1	C	1423278_at	AI893646	Ptprk	protein tyrosine phosphatase, receptor type, K	2,37	-0,98	-1,11	10	27 764 235
1	C	1434277_a_at	BG069663	6430570G24		2,88	-0,63	-2,71	11	86 752 622
1	C	1418519_at	BC012637	Aadat	aminoadipate aminotransferase	2,50	-1,24	-2,26	8	63 398 265
1	C	1423235_at	BM222025	Ap3b1	adaptor-related protein complex 3, beta 1 subunit	2,32	-1,30	-1,46	13	95 459 729
1	C	1436039_at	BM245957			2,52	-1,37	-2,96	13	24 484 196
1	C	1448438_at	NM_033562	Derl2	Der1-like domain family, member 2	2,43	0,53	-1,66	11	70 823 639
1	C	1433570_s_at	BG083730	Mak10	MAK10 homolog, amino-acid N-acetyltransferase subunit, (S. cerevisiae)	2,15	-0,45	-2,15	13	59 595 033
1	C	1437070_at	BB224362	Cdc14b	CDC14 cell division cycle 14 homolog B (S. cerevisiae)	2,33	-0,81	-0,50	13	64 204 579
1	C	1448353_x_at	NM_133933	Rpn1	ribophorin I	3,15	0,02	-1,52	6	88 050 156
1	C	1448903_at	NM_053102	Sep15	selenoprotein	2,09	0,34	-1,10	3	144 507 812
1	C	1417843_s_at	NM_133191	Eps8l2	EPS8-like 2	2,80	-1,42	-0,41	7	141 190 321
1	C	1439468_at	BM239035	Bach2	BTB and CNC homology 2	2,11	-1,17	-0,74	4	32 567 562
1	C	1456438_x_at	BB145729	Rpn1	ribophorin I	2,79	0,32	-1,53	6	88 050 156
1	C	1423006_at	NM_008842	Pim1	proviral integration site 1	2,23	-1,99	-2,19	17	29 217 823
1	C	1427377_x_at	M77015	Hsd3b3	hydroxy-delta-5-steroid dehydrogenase, 3 beta- and steroid delta-isomerase 3	2,08	-0,72	-1,50	3	98 870 584
1	C	1434511_at	BM233125	Phkb	phosphorylase kinase beta	2,03	-0,86	-1,65	8	88 731 106
1	C	1418206_at	NM_022324	Sdf2l1	stromal cell-derived factor 2-like 1	2,17	-0,14	-0,93	16	17 043 702
1	C	1435084_at	BB200607	C730049O14Rik	RIKEN cDNA C730049O14 gene	2,46	-0,62	-3,14	18	
1	C	1425540_at	BC024893	Otc	ornithine transcarbamylase	2,05	-1,86	-0,78	X	9 409 317
1	C	1419287_at	NM_025486	Hspc171	HSPC171 protein	2,27	0,45	-1,35	8	108 215 511
1	C	1452384_at	AV224446	Enpp3	ectonucleotide pyrophosphatase/phosphodiesterase 3	2,49	-0,39	-2,31	10	24 463 318
1	C	1453263_at	AK021042	Mak10	MAK10 homolog, amino-acid N-acetyltransferase subunit, (S. cerevisiae)	2,24	-0,18	-2,46	13	59 595 033
1	C	1446769_at	AV028075	2810439F02Rik	RIKEN cDNA 2810439F02 gene	2,11	-1,23	-0,98	18	12 786 968
1	C	1427450_x_at	BI080370	Myo1b	myosin IB	2,26	-0,55	-2,41	1	51 694 312
1	C	1416470_a_at	NM_133933	Rpn1	ribophorin I	2,57	0,64	-1,19	6	88 050 156
1	C	1426263_at	AY059394	Igsf4c	immunoglobulin superfamily, member 4C	2,25	-0,21	-2,14	7	24 190 782
1	C	1460544_at	BG083730	Mak10	MAK10 homolog, amino-acid N-acetyltransferase subunit, (S. cerevisiae)	2,51	-0,63	-2,20	13	59 595 033
1	C	1444883_at	AW551464	Tmem19	transmembrane protein 19	2,04	-0,61	-1,41	10	114 744 850
1	C	1448160_at	NM_008879	Lcp1	lymphocyte cytosolic protein 1	2,24	1,38	-2,51	14	73 870 579
1	C	1427472_a_at	BC022129	C8b	complement component 8, beta subunit	2,46	-0,67	-1,22	4	104 264 248
1	C	1455839_at	BM211361	Ugcgl1	UDP-glucose ceramide glucosyltransferase-like 1	2,16	-0,19	-1,29	1	36 088 034
1	C	1418837_at	AI195046	Qprt	quinolinate phosphoribosyltransferase	2,18	-1,06	-0,35	7	126 898 918
1	C	1448524_s_at	NM_009279	Ssr4	signal sequence receptor, delta	2,16	0,75	-1,56	X	70 039 781
1	C	1448989_a_at	AI255256	Myo1b	myosin IB	2,67	-1,21	-2,90	1	51 694 312
1	C	1420525_a_at	NM_008769	Otc	ornithine transcarbamylase	2,06	-0,16	-1,17	X	9 409 317
1	C	1451600_s_at	BC019147	LOC13909		2,02	-0,48	-1,02	8	107 972 898

1	C	1452130_at	BI790903	Txndc14	thioredoxin domain containing 14	2,11	-0,51	-1,31	2	84 472 157
1	C	1455388_at	BB549335	Pcmtd1	protein-L-isoaspartate (D-aspartate) O-methyltransferase domain containing 1	2,00	-0,55	-0,99	1	7 079 230
1	C	1437380_x_at	BB558114	Pgd	phosphogluconate dehydrogenase	2,09	0,02	-1,49	4	
1	C	1453286_at	BB085537	Plxna2	plexin A2	2,17	0,10	-2,04	1	196 320 644
1	D	1428022_at	BC027556	Lcn13	lipocalin 13	11,06	0,40	2,65	2	25 522 082
1	D	1456074_at	BB143568	Sdro	orphan short chain dehydrogenase/reductase	3,68	1,30	4,55	10	127 301 483
1	D	1418028_at	NM_010024	Dct	dopachrome tautomerase	4,73	1,27	3,27	14	116 895 152
1	D	1430785_at	BB150587	Sdro	orphan short chain dehydrogenase/reductase	3,33	1,19	3,30	10	127 301 483
1	D	1417828_at	NM_007474	Aqp8	aquaporin 8	5,17	1,10	5,44	7	123 253 459
1	D	1457823_at	BB533736	Cyr61	cysteine rich protein 61	3,37	-1,32	1,26	3	145 584 362
1	D	1453191_at	BB044654	Col27a1	procollagen, type XXVII, alpha 1	2,99	1,49	1,56	4	62 702 186
1	D	1434422_at	AV027153	1700066M21Rik	RIKEN cDNA 1700066M21 gene	2,98	1,40	1,58	1	
1	D	1436070_at	BM933153	Glo1	glyoxalase 1	3,11	1,23	3,09	17	30 319 872
1	D	1455468_at	BB768758			3,46	1,32	1,81	7	35 394 258
1	D	1425314_at	AF435926	Gpr98	G protein-coupled receptor 98	2,67	-0,14	2,18	13	81 558 503
1	D	1438751_at	BB736474	Slc30a10	solute carrier family 30, member 10	3,20	0,78	2,15	1	187 155 635
1	D	1455446_x_at	BF228057	Acadsb	acyl-Coenzyme A dehydrogenase, short/branched chain	2,52	0,92	1,78	7	131 201 850
1	D	1415710_at	BM123013	Cox18	COX18 cytochrome c oxidase assembly homolog (S. cerevisiae)	2,24	1,23	2,87	5	91 289 926
1	D	1423693_at	BC011218	Ela1	elastase 1, pancreatic	3,43	-0,42	2,30	15	100 502 458
1	D	1424273_at	BC025822	Cyp2c70	cytochrome P450, family 2, subfamily c, polypeptide 70	3,45	1,96	0,96	19	40 206 672
1	D	1448233_at	BE630020	Prnp	prion protein	3,33	-0,64	1,27	2	131 601 397
1	D	1422061_at	NM_054080	Akr1c20	aldo-keto reductase family 1, member C20	3,29	1,56	1,72	13	4 506 404
1	D	1424352_at	BC025936	Cyp4a12	cytochrome P450, family 4, subfamily a, polypeptide 12	2,74	0,31	2,81	4	114 796 977
1	D	1438711_at	BB667651	Pklr	pyruvate kinase liver and red blood cell	3,64	0,50	1,14	3	89 222 068
1	D	1449817_at	NM_021022	Abcb11	ATP-binding cassette, sub-family B (MDR/TAP), member 11	2,93	1,56	1,48	2	69 039 124
1	D	1434329_s_at	BG074607	Adipor2	adiponectin receptor 2	2,16	1,51	1,94	6	119 318 770
1	D	1431302_a_at	AK011172	Nudt7	nudix (nucleoside diphosphate linked moiety X)-type motif 7	2,35	-0,10	2,97	8	117 019 591
1	D	1449460_at	AF403041	Asb13	ankyrin repeat and SOCS box-containing protein 13	2,91	1,27	1,50	13	3 633 323
1	D	1424953_at	BC021614	BC021614	cDNA sequence BC021614	2,47	0,26	1,59	19	4 057 486
1	D	1427513_at	BI144810	BC024137	cDNA sequence BC024137	2,32	0,18	1,69	8	117 039 280
1	D	1443856_at	BE951220	Rabep1	rabaptin, RAB GTPase binding effector protein 1	2,32	-0,07	1,53	11	70 661 119
1	D	1450387_s_at	NM_009647	Ak3l1	adenylate kinase 3 alpha-like 1	2,03	1,17	2,16	4	100 917 690
1	D	1418209_a_at	NM_019410	Pfn2	profilin 2	2,52	0,52	5,73	3	57 929 824
1	D	1421662_a_at	NM_030254	Tusc3	tumor suppressor candidate 3	2,25	0,40	1,26	8	40 474 408
1	D	1418457_at	AF252873	Cxcl14	chemokine (C-X-C motif) ligand 14	4,23	-0,05	1,29	13	56 298 264
1	D	1419134_at	NM_021375	Rhbg	Rhesus blood group-associated B glycoprotein	2,46	-1,17	2,59	3	88 328 800
1	D	1450064_at	BM228488	Fmn2	formin 2	2,36	-0,79	1,97	1	176 338 520
1	D	1424726_at	BC014685	Tmem150	transmembrane protein 150	3,35	0,28	1,64	6	72 285 001
1	D	1425329_a_at	AF332060	Cyb5r3	cytochrome b5 reductase 3	2,42	1,30	3,11	15	82 981 269
1	D	1433645_at	BM207133	2210409B22Rik	RIKEN cDNA 2210409B22 gene	2,62	0,89	2,41	4	
1	D	1422186_s_at	NM_029787	Cyb5r3	cytochrome b5 reductase 3	2,42	1,49	1,83	15	82 981 269
1	D	1435193_at	BB085904	A230050P20Rik	RIKEN cDNA A230050P20 gene	2,46	0,22	3,16	9	20 619 097
1	D	1430125_s_at	AK009256	Pqlc1	PQ loop repeat containing 1	2,08	-0,09	1,09	18	80 418 365
1	D	1434181_at	BG070082	Plekhc1	pleckstrin homology domain containing, family C (with FERM domain) member 1	2,86	-0,51	1,04	14	44 380 668
1	D	1434180_at	BG070082	Plekhc1	pleckstrin homology domain containing, family C (with FERM domain) member 1	2,33	-0,59	1,42	14	44 380 668
1	D	1455918_at	BB224790	Adrb3	adrenergic receptor, beta 3	2,02	0,47	4,70	8	28 691 711
1	D	1421912_at	AA276202	Slc23a1	solute carrier family 23 (nucleobase transporters), member 1	2,10	-0,87	1,55	18	35 740 579
1	D	1446412_at	AW538350	Wwox	WW domain-containing oxidoreductase	2,97	0,13	3,19	8	117 325 672
1	D	1451003_at	BM213179	Map3k7ip2	mitogen-activated protein kinase kinase kinase 7 interacting protein 2	2,05	0,82	1,48	10	7 596 330
1	D	1416402_at	AV382118	Abcb10	ATP-binding cassette, sub-family B (MDR/TAP), member 10	2,04	0,56	1,06	8	126 838 550
2	_	1443128_at	BB522781	Ints6	integrator complex subunit 6	-2,81	5,43	5,03	14	61 630 437
2	_	1448506_at	NM_007618	Serpina6	serine (or cysteine) peptidase inhibitor, clade A, member 6	-3,78	2,33	6,97	12	104 047 682
2	_	1422230_s_at	NM_007812	Cyp2a5	cytochrome P450, family 2, subfamily a, polypeptide 5	-4,14	3,10	1,03	7	26 544 100
2	_	1434442_at	BB667844	D5Ertd593e	DNA segment, Chr 5, ERATO Doi 593, expressed	-4,39	2,28	3,72	5	93 678 254
2	_	1455454_at	BG073853	Akr1c19	aldo-keto reductase family 1, member C19	-2,50	2,10	-0,81	13	4 232 985
2	_	1450971_at	AK010420	Gadd45b	growth arrest and DNA-damage-inducible 45 beta	-2,64	2,95	1,68	10	80 333 219
2	_	1437821_at	BB408240	Diap1	diaphanous homolog 1 (Drosophila)	-2,13	2,27	2,45	18	37 970 800
2	_	1426875_s_at	BM210600	Srxn1	sulfiredoxin 1 homolog (S. cerevisiae)	-3,03	2,24	2,01	2	151 797 170
2	_	1448667_x_at	AV174616	Tob2	transducer of ERBB2, 2	-2,62	2,55	1,01	15	81 675 524

3	A	1448253_at	NM_008133	Glud1	glutamate dehydrogenase 1	0,39	3,01	3,93	14	33 139 842
3	A	1417651_at	NM_007815	Cyp2c29	cytochrome P450, family 2, subfamily c, polypeptide 29	-0,26	2,79	4,84	19	39 340 413
3	A	1448300_at	NM_025569	Mgst3	microsomal glutathione S-transferase 3	-0,47	2,48	7,66	1	169 209 058
3	A	1424108_at	BC024663	Glo1	glyoxalase 1	1,51	2,35	6,40	17	30 319 872
3	A	1422001_at	U95962	Inhbc	inhibin beta-C	0,22	2,46	3,76	10	126 759 270
3	A	1418701_at	NM_007744	Comt	catechol-O-methyltransferase	0,62	2,47	8,86	16	18 321 105
3	A	1420919_at	BB768208	Sgk3	serum/glucocorticoid regulated kinase 3	-1,34	2,19	3,58	1	9 833 494
3	A	1418507_s_at	NM_007706	Socs2	suppressor of cytokine signaling 2	-2,01	2,34	3,48	10	94 841 729
3	A	1452439_s_at	AF250135	Sfrs2	splicing factor, arginine/serine-rich 2 (SC-35)	-1,06	2,19	5,00	11	116 665 996
3	B	1449523_at	NM_009746	Bcl7c	B-cell CLL/lymphoma 7C	-0,12	3,07	-4,80	7	127 496 127
3	B	1450410_a_at	BC022913	4930570C03Rik	RIKEN cDNA 4930570C03 gene	-0,43	2,78	-1,98	15	97 612 398
3	B	1455439_a_at	AI642438	Lgals1	lectin, galactose binding, soluble 1	1,24	3,52	-2,26	15	78 753 979
3	B	1460151_at	BG070910			0,57	3,02	-2,25	?	
3	B	1416866_at	NM_009748	Bet1	blocked early in transport 1 homolog (S. cerevisiae)	2,09	2,43	-2,37	6	4 026 903
3	B	1457324_at	BM115786	Oprs1	opioid receptor, sigma 1	0,21	2,55	-5,95	4	41 927 006
3	B	1458092_at	BM232899	Ap3m1	adaptor-related protein complex 3, mu 1 subunit	-0,08	2,47	-3,54	14	19 823 361
3	B	1419573_a_at	NM_008495	Lgals1	lectin, galactose binding, soluble 1	1,14	2,91	-2,53	15	78 753 979
3	B	1416632_at	BC011081	Mod1	malic enzyme, supernatant	0,79	2,96	-1,45	9	86 378 093
3	B	1419426_s_at	NM_011335	Ccl21a	chemokine (C-C motif) ligand 21a	0,52	2,10	-1,75	4	42 633 918
3	B	1424811_at	BC024605	Cml5	camello-like 5	0,17	2,51	-4,40	6	85 782 877
3	B	1445980_at	BG076366	Aldh1a1	aldehyde dehydrogenase family 1, subfamily A1	-1,03	2,60	-2,69	19	20 669 078
3	B	1434437_x_at	AV301324	Rrm2	ribonucleotide reductase M2	-0,03	2,10	-2,50	12	25 297 361
3	B	1444057_at	BB253461	Ubxd2	UBX domain containing 2	-0,14	2,12	-2,65	1	130 071 726
3	B	1426516_a_at	AK014526	Lpin1	lipin 1	0,44	2,21	-5,46	12	16 562 115
3	C	1450893_a_at	AV132698	Ubap1	ubiquitin-associated protein 1	0,68	3,34	-0,13	4	41 537 723
3	C	1435452_at	BM121082	Tmem20	transmembrane protein 20	0,14	3,13	0,12	19	38 461 123
3	C	1433443_a_at	BB705380	Hmgcs1	3-hydroxy-3-methylglutaryl-Coenzyme A synthase 1	-0,89	2,89	-0,33	13	114 526
3	C	1443673_x_at	BB710847			1,89	2,92	-1,26	?	
3	C	1433599_at	BM239162	Baz1a	bromodomain adjacent to zinc finger domain 1A	-0,49	2,88	-1,22	12	55 811 449
3	C	1439443_x_at	AV103412	Tkt	transketolase	1,56	3,28	0,57	14	29 378 157
3	C	1454785_at	BE951717	Dusp11	dual specificity phosphatase 11 (RNA/RNP complex 1-interacting)	-0,11	2,72	-0,13	6	85 907 904
3	C	1428294_at	AK016551	Zfp259	zinc finger protein 259	-0,05	3,22	-0,27	9	46 024 160
3	C	1420836_at	BB032012	Slc25a30	solute carrier family 25, member 30	1,92	3,33	0,74	14	74 496 153
3	C	1438001_x_at	AV054377	Reep5	receptor accessory protein 5	1,17	2,75	-1,33	18	34 469 688
3	C	1433445_x_at	BB705380	Hmgcs1	3-hydroxy-3-methylglutaryl-Coenzyme A synthase 1	-0,42	2,63	-0,84	13	114 526
3	C	1425627_x_at	J03952	Gstm1	glutathione S-transferase, mu 1	0,30	2,65	0,61	3	108 140 313
3	C	1451814_a_at	AF061972	Htatip2	HIV-1 tat interactive protein 2, homolog (human)	-1,35	2,68	-0,16	7	49 627 173
3	C	1447898_s_at	BB054483	Sfrs6	splicing factor, arginine/serine-rich 6	0,96	2,70	0,42	2	162 622 985
3	C	1449375_at	NM_133960	Ces6	carboxylesterase 6	1,29	2,63	-0,13	8	107 623 136
3	C	1422301_at	NM_008049	Ftl2	ferritin light chain 2	1,48	2,83	0,55	4	124 612 982
3	C	1429001_at	AK009757	Pir	pirin	0,21	3,14	-1,61	X	159 613 541
3	C	1438953_at	BB359521	Figf	c-fos induced growth factor	0,76	2,82	-0,93	X	159 717 652
3	C	1425626_at	J03952	Gstm1	glutathione S-transferase, mu 1	1,01	2,65	0,22	3	108 140 313
3	C	1421209_s_at	NM_010547	Ikbkg	inhibitor of kappaB kinase gamma	1,23	2,71	0,40	X	70 685 524
3	C	1442881_at	BF462648			1,45	3,36	0,06	?	
3	C	1450018_s_at	BB032012	Slc25a30	solute carrier family 25, member 30	1,90	3,10	0,49	14	74 496 153
3	C	1428593_at	BG095162	1700029F09Rik	RIKEN cDNA 1700029F09 gene	1,45	2,61	0,14	1	
3	C	1451415_at	BC016562	1810011O10Rik	RIKEN cDNA 1810011O10 gene	0,42	2,47	-0,93	8	25 903 160
3	C	1416867_at	NM_009748	Bet1	blocked early in transport 1 homolog (S. cerevisiae)	1,22	2,59	-0,35	6	4 026 903
3	C	1427202_at	AV002340	4833442J19Rik	RIKEN cDNA 4833442J19 gene	-0,97	2,44	-1,03	6	149 098 766
3	C	1426870_at	BM939903	Fbxo33	F-box only protein 33	0,66	2,53	0,32	12	60 118 669
3	C	1416998_at	NM_021511	Rrs1	RRS1 ribosome biogenesis regulator homolog (S. cerevisiae)	0,58	2,43	-0,87	1	9 530 655
3	C	1454227_at	AK015389	Htatip2	HIV-1 tat interactive protein 2, homolog (human)	0,51	2,24	-0,11	7	49 627 173
3	C	1456865_x_at	AV304745	Rrs1	RRS1 ribosome biogenesis regulator homolog (S. cerevisiae)	0,24	2,56	-0,22	1	9 530 655
3	C	1449037_at	AI467599	Crem	cAMP responsive element modulator	0,92	2,75	-0,40	18	3 266 353
3	C	1424860_at	BM200153	Zbtb24	zinc finger and BTB domain containing 24	0,13	2,17	-0,57	10	41 138 810
3	C	1428010_at	BC028435	Timm9	translocase of inner mitochondrial membrane 9 homolog (yeast)	0,15	2,76	0,45	12	72 041 777
3	C	1424861_at	BM200153	Zbtb24	zinc finger and BTB domain containing 24	1,34	2,60	-1,48	10	41 138 810
3	C	1433627_at	AW546839	Sec23ip	Sec23 interacting protein	0,92	2,09	0,09	7	128 536 128
3	C	1428490_at	AK008884	C1galt1	core 1 UDP-galactose:N-acetylgalactosamine-alpha-R beta 1,3-galactosyltransferase	-0,01	2,36	0,00	6	7 795 332

3	C	1418241_at	AW537576	Usf2	upstream transcription factor 2	0,06	2,08	0,68	7	30 654 755
3	C	1415948_at	BC027426	Creg1	cellular repressor of E1A-stimulated genes 1	-0,30	2,36	-0,11	1	167 600 454
3	C	1418322_at	AI467599	Crem	cAMP responsive element modulator	1,52	2,62	-0,67	18	3 266 353
3	C	1423722_at	BC004013	Tmem49	transmembrane protein 49	1,11	2,28	0,44	11	86 400 059
3	C	1449886_a_at	NM_013896	Timm9	translocase of inner mitochondrial membrane 9 homolog (yeast)	0,11	2,53	0,20	12	72 041 777
3	C	1433444_at	BB705380	Hmgcs1	3-hydroxy-3-methylglutaryl-Coenzyme A synthase 1	-0,51	2,02	-0,59	13	114 526
3	C	1444217_at	BM125546	Mrpl38	mitochondrial ribosomal protein L38	0,06	2,62	0,68	11	115 947 911
3	C	1460624_at	BB047007	6330564D18Rik	RIKEN cDNA 6330564D18 gene	0,52	2,18	-0,14	1	
3	C	1424223_at	BC019205	1700020C11Rik	RIKEN cDNA 1700020C11 gene	-0,53	2,26	0,56	11	3 991 483
3	C	1455310_at	AW681705	Rbm16	RNA binding motif protein 16	0,84	2,15	0,69	17	
3	C	1426426_at	AV111031	Rbm13	RNA binding motif protein 13	1,23	2,30	0,22	8	32 625 402
3	C	1433446_at	BB705380	Hmgcs1	3-hydroxy-3-methylglutaryl-Coenzyme A synthase 1	-1,32	2,07	-1,33	13	114 526
3	C	1438954_x_at	BB359521	Figf	c-fos induced growth factor	0,32	2,33	-0,33	X	159 717 652
3	C	1427960_at	BC028826	Ugt2b34	UDP glucuronosyltransferase 2 family, polypeptide B34	-0,11	2,25	-1,00	5	87 964 331
3	C	1455165_at	BE335227	Rora	RAR-related orphan receptor alpha	0,37	2,50	-0,12	9	68 452 965
3	C	1429758_at	BB046208	1700017B05Rik	RIKEN cDNA 1700017B05 gene	1,11	2,15	0,24	9	57 050 459
3	C	1423963_at	BC020044	Wdr26	WD repeat domain 26	0,08	2,27	0,58	1	183 014 367
3	C	1451765_a_at	AF136571	Entpd5	ectonucleoside triphosphate diphosphohydrolase 5	0,27	2,35	-0,15	12	85 263 604
3	C	1460249_at	NM_080795	Lnx2	ligand of numb-protein X 2	1,22	2,15	0,07	5	147 327 452
3	C	1431026_at	BF168504	Nat12	N-acetyltransferase 12	0,69	2,01	-0,56	14	
3	C	1423185_a_at	AV132698	Ubap1	ubiquitin-associated protein 1	1,43	2,07	-0,16	4	41 537 723
3	C	1436590_at	BG071940	Ppp1r3b	protein phosphatase 1, regulatory (inhibitor) subunit 3B	-0,40	2,06	-0,15	8	36 844 257
3	C	1457674_at	AV255519	Vps13a	vacuolar protein sorting 13A (yeast)	-0,37	2,20	0,35	19	16 683 692
3	C	1440575_at	BB541289	Hspa4	heat shock protein 4	0,07	2,34	0,50	11	53 103 237
3	C	1430007_a_at	BF319512	Cabyr	calcium-binding tyrosine-(Y)-phosphorylation regulated (fibrousheathin 2)	0,79	2,69	0,51	18	12 884 523
3	C	1433789_at	BI082172	Snhg3	small nucleolar RNA host gene (non-protein coding) 3	0,42	2,09	-0,77	4	
3	C	1419461_at	BI455861	Rpp14	ribonuclease P 14 subunit (human)	0,51	2,26	-0,45	14	6 872 245
3	C	1455951_at	BM211104	Mars	methionine-tRNA synthetase	1,35	2,01	0,46	10	126 699 176
3	C	1454839_a_at	AV285979	Ccdc84	coiled-coil domain containing 84	0,83	2,07	0,04	9	44 161 160
3	C	1435733_x_at	AI646809	1500026D16Rik	RIKEN cDNA 1500026D16 gene	0,89	2,09	0,23	19	5 601 872
3	C	1424019_at	BC007151	Nol1	nucleolar protein 1	0,12	2,13	0,70	6	125 097 557
3	C	1420113_s_at	AA409325	2410022L05Rik	RIKEN cDNA 2410022L05 gene	1,22	2,01	0,55	8	13 884 771
3	C	1417441_at	NM_013888	Dnajc12	DnaJ (Hsp40) homolog, subfamily C, member 12	-0,82	2,23	-0,01	10	62 781 973
3	C	1434307_at	AW489972	Tmem64	transmembrane protein 64	0,74	2,11	0,83	4	15 192 977
3	C	1419281_a_at	BC021397	Zfp259	zinc finger protein 259	-0,21	2,09	-0,01	9	46 024 160
3	C	1417384_at	NM_007647	Entpd5	ectonucleoside triphosphate diphosphohydrolase 5	-0,33	2,38	0,50	12	85 263 604
3	C	1433581_at	AV309085	1190002N15Rik	RIKEN cDNA 1190002N15 gene	0,64	2,21	0,06	9	94 328 874
3	C	1429392_at	BI558553	Wdr40a	WD repeat domain 40A	0,90	2,00	-0,27	4	41 479 974
3	C	1425776_a_at	BC025632	C87436	expressed sequence C87436	0,09	2,07	-0,34	6	86 404 031
3	C	1425948_a_at	BC022676	Slc25a30	solute carrier family 25, member 30	2,13	2,29	0,01	14	74 496 153
3	C	1417383_at	NM_007647	Entpd5	ectonucleoside triphosphate diphosphohydrolase 5	-0,39	2,02	0,68	12	85 263 604
3	C	1455991_at	BG094881	Ccbl2	cysteine conjugate-beta lyase 2	-0,69	2,02	0,08	3	142 638 465
3	C	1455121_at	BB553784	Lcor	ligand dependent nuclear receptor corepressor	0,56	2,01	0,60	19	41 602 949
3	C	1435376_at	BB770542	Ppapdc1	phosphatidic acid phosphatase type 2 domain containing 1	0,36	2,12	-0,10	8	27 190 891
3	C	1415947_at	BC027426	Creg1	cellular repressor of E1A-stimulated genes 1	0,66	2,07	-0,90	1	167 600 454
3	C	1422488_at	NM_019761	Nxt1	NTF2-related export protein 1	0,28	2,01	0,34	2	148 364 082
3	C	1454364_at	BB110590	9530025L08Rik	RIKEN cDNA 9530025L08 gene	0,46	2,33	-0,06	19	
3	C	1445391_at	AW554652	Diap1	diaphanous homolog 1 (Drosophila)	-0,82	2,05	0,61	18	37 970 800
3	C	1437046_x_at	AI639566	4930504E06Rik	RIKEN cDNA 4930504E06 gene	-1,33	2,03	-0,09	3	95 368 347
3	C	1439128_at	AI595815	Zbtb20	zinc finger and BTB domain containing 20	1,74	2,11	-0,19	16	43 429 639
3	C	1455580_at	BE851876	Usp6nl	USP6 N-terminal like	-0,08	2,04	-0,44	2	6 270 004
3	D	1423642_at	BC005547	Tubb2c	tubulin, beta 2c	1,01	4,31	0,85	2	25 044 170
3	D	1448484_at	NM_009665	Amd1	S-adenosylmethionine decarboxylase 1	0,48	3,76	1,80	10	39 976 874
3	D	1433898_at	AV000840	AV025504	expressed sequence AV025504	1,90	4,29	1,69	14	
3	D	1437073_x_at	BB115446	AV025504	expressed sequence AV025504	2,46	4,21	1,39	14	
3	D	1454709_at	BG075363	Tmem64	transmembrane protein 64	0,39	3,35	1,28	4	15 192 977
3	D	1456405_at	BG063067	Dido1	death inducer-obliterator 1	-0,74	3,39	2,24	2	180 610 466
3	D	1456059_at	BI677127	Psmd11	proteasome (prosome, macropain) 26S subunit, non-ATPase, 11	0,46	3,12	1,52	11	80 244 822
3	D	1430536_a_at	BB071632	Erh	enhancer of rudimentary homolog (Drosophila)	-0,78	3,33	2,32	12	81 552 884
3	D	1448330_at	NM_010358	Gstm1	glutathione S-transferase, mu 1	0,74	3,41	0,81	3	108 140 313
3	D	1430135_at	AK018651	Dnase2a	deoxyribonuclease II alpha	0,92	3,22	2,31	8	87 798 728

3	D	1417374_at	AW491660	Tuba4	tubulin, alpha 4	0,57	3,12	0,71	1	75 098 121
3	D	1424118_a_at	BC027121	Spbc25	spindle pole body component 25 homolog (S. cerevisiae)	-0,22	2,92	1,99	2	68 994 734
3	D	1447411_at	AI506883	Ugdh	UDP-glucose dehydrogenase	0,88	3,42	0,99	5	65 692 358
3	D	1424677_at	AF336850	Cyp2j9	cytochrome P450, family 2, subfamily j, polypeptide 9	-1,11	2,91	2,14	4	96 060 448
3	D	1448641_at	NM_134012	Mbtd1	mbt domain containing 1	-0,60	2,74	2,18	11	93 728 498
3	D	1420835_at	BB032012	Slc25a30	solute carrier family 25, member 30	2,88	3,65	1,05	14	74 496 153
3	D	1455940_x_at	BB453609	Wdr6	WD repeat domain 6	2,04	3,37	1,72	9	108 430 415
3	D	1426404_a_at	BI150320	Rnf11	ring finger protein 11	0,36	2,97	1,13	4	108 950 789
3	D	1422608_at	BE648432	Arpp19	cAMP-regulated phosphoprotein 19	-0,22	2,67	1,83	9	74 824 336
3	D	1416755_at	AK002290	Dnajb1	DnaJ (Hsp40) homolog, subfamily B, member 1	-0,68	2,76	1,35	8	86 498 297
3	D	1445144_at	BG072125	Sntb1	syntrophin, basic 1	-1,68	2,90	2,68	15	55 469 236
3	D	1438816_at	BM247201	Ahctf1	AT hook containing transcription factor 1	1,32	3,24	1,02	1	181 582 766
3	D	1424876_s_at	BB040507	Spg20	spastic paraplegia 20, spartin (Troyer syndrome) homolog (human)	0,80	2,82	1,15	3	55 200 068
3	D	1429169_at	AK011224	Rbm3	RNA binding motif protein 3	0,32	2,59	1,06	X	7 297 025
3	D	1448306_at	NM_010907	Nfkbia	nuclear factor of kappa light chain gene enhancer in B-cells inhibitor, alpha	-1,17	2,60	1,33	12	56 407 990
3	D	1416416_x_at	NM_010358	Gstm1	glutathione S-transferase, mu 1	0,87	2,95	0,71	3	108 140 313
3	D	1451509_at	BC019453	Taf9	TAF9 RNA polymerase II, TATA box binding protein (TBP)-associated factor	1,09	3,11	0,73	13	101 751 863
3	D	1424081_at	BC016195	Pcgf6	polycomb group ring finger 6	1,52	2,53	2,35	19	47 086 929
3	D	1440230_at	BB548458	Lrrc54	leucine rich repeat containing 54	0,49	2,83	1,54	7	98 226 059
3	D	1434879_at	BI794243	Cdc34	cell division cycle 34 homolog (S. cerevisiae)	0,15	2,72	1,27	10	79 085 323
3	D	1416721_s_at	AW539046	Sfrs6	splicing factor, arginine/serine-rich 6	0,89	2,64	0,77	2	162 622 985
3	D	1416171_at	NM_133714	2310037I24Rik	RIKEN cDNA 2310037I24 gene	-0,15	2,97	1,16	15	98 346 845
3	D	1417373_a_at	AW491660	Tuba4	tubulin, alpha 4	1,47	2,88	0,67	1	75 098 121
3	D	1455534_s_at	BM220135	Osbpl11	oxysterol binding protein-like 11	0,73	2,70	1,43	16	33 104 817
3	D	1416835_s_at	NM_009665	Amd1	S-adenosylmethionine decarboxylase 1	1,08	3,01	1,23	10	39 976 874
3	D	1429539_at	AK012404	Bcl2l13	BCL2-like 13 (apoptosis facilitator)	-0,27	2,70	1,43	6	120 801 848
3	D	1439061_at	BB503633	Ccdc84	coiled-coil domain containing 84	-0,11	2,46	0,75	9	44 161 160
3	D	1426381_at	BM199989	Pprc1	peroxisome proliferative activated receptor, gamma, coactivator-related 1	0,69	3,05	1,52	19	
3	D	1436027_at	BM220135	Osbpl11	oxysterol binding protein-like 11	0,38	2,27	0,82	16	33 104 817
3	D	1416566_at	NM_011499	Strap	serine/threonine kinase receptor associated protein	-0,19	2,36	2,61	6	137 699 321
3	D	1434075_at	AV374294	BC030336	cDNA sequence BC030336	0,47	2,39	0,94	7	120 524 193
3	D	1428223_at	AK006096	Mfsd2	major facilitator superfamily domain containing 2	-2,50	2,90	0,74	4	122 449 157
3	D	1423275_at	BB381966	E130113E03Rik	RIKEN cDNA E130113E03 gene	0,56	2,65	1,84	14	
3	D	1447930_at	AV357135	Baz1a	bromodomain adjacent to zinc finger domain 1A	-0,29	2,60	1,38	12	55 852 987
3	D	1422433_s_at	NM_010497	Idh1	isocitrate dehydrogenase 1 (NADP+), soluble	1,22	2,38	1,68	1	65 092 904
3	D	1416256_a_at	NM_011655	Tubb5	tubulin, beta 5	1,18	2,54	2,70	17	35 441 972
3	D	1415892_at	NM_009163	Sgpl1	sphingosine phosphate lyase 1	0,67	2,38	1,88	10	60 494 000
3	D	1423828_at	AF127033	Fasn	fatty acid synthase	2,09	2,30	0,94	11	120 622 047
3	D	1455855_x_at	BB168316	Fancl	Fanconi anemia, complementation group L	0,51	2,32	0,77	11	26 287 083
3	D	1417750_a_at	NM_026331	Slc25a37	solute carrier family 25, member 37	0,87	2,28	1,08	14	68 195 180
3	D	1426727_s_at	BC004771	Ppp1r10	protein phosphatase 1, regulatory subunit 10	0,36	2,52	1,46	17	35 525 250
3	D	1437869_at	BF140684	3222402P14Rik	RIKEN cDNA 3222402P14 gene	-0,41	2,48	2,32	9	
3	D	1416201_at	BF020879	Crk	v-crk sarcoma virus CT10 oncogene homolog (avian)	0,50	2,40	1,20	11	75 495 504
3	D	1433595_at	BB409668	Slc35d1	solute carrier family 35 (UDP-glucuronic acid/UDP-N-acetylgalactosamine dual transporter), member D1	0,57	2,55	1,65	4	102 669 649
3	D	1451166_a_at	BC026784	Ccdc101	coiled-coil domain containing 101	-0,49	2,31	1,44	7	126 440 518
3	D	1429787_x_at	AK008144	Zwint	ZW10 interactor	-1,57	2,32	1,35	10	72 050 194
3	D	1434514_at	AV277021	Rbm15	RNA binding motif protein 15	1,09	2,20	1,12	3	107 453 041
3	D	1427479_at	AA413531	LOC544881		0,88	2,21	1,40	12	88 507 970
3	D	1421262_at	BC020991	Lipg	lipase, endothelial	-0,69	2,11	2,18	18	75 064 746
3	D	1427333_s_at	BF226494	Sfrs15	splicing factor, arginine/serine-rich 15	0,46	2,19	1,56	16	90 118 004
3	D	1431011_at	AK019713	Dlst	dihydrolipoamide S-succinyltransferase (E2 component of 2-oxo-glutarate complex)	-0,99	2,31	1,16	12	85 999 998
3	D	1454311_at	BB029361			0,41	2,19	0,80	2	130 702 852
3	D	1435734_x_at	BB396413	Dus1l	dihydrouridine synthase 1-like (S. cerevisiae)	0,81	2,31	0,66	11	120 605 291
3	D	1452047_at	AK013924	Cacybp	calcyclin binding protein	-1,11	2,21	0,99	1	162 039 601
3	D	1417636_at	NM_008135	Slc6a9	solute carrier family 6 (neurotransmitter transporter, glycine), member 9	-1,48	2,57	2,68	4	117 333 189
3	D	1426726_at	BC004771	Ppp1r10	protein phosphatase 1, regulatory subunit 10	0,09	2,33	1,16	17	35 525 250
3	D	1452914_at	AK010587	2410024N18Rik	RIKEN cDNA 2410024N18 gene	-0,38	2,67	1,31	5	
3	D	1446258_at	BB118716	Sntb1	syntrophin, basic 1	-0,01	2,23	2,07	15	55 469 236
3	D	1448273_at	NM_008180	Gss	glutathione synthetase	0,86	2,15	1,06	2	155 254 623

3	D	1448707_at	NM_025444	Taf13	TAF13 RNA polymerase II, TATA box binding protein (TBP)-associated factor	0,77	2,42	0,90	3	108 699 754
3	D	1455679_at	BE457727	5830411E10Rik	RIKEN cDNA 5830411E10 gene	-0,05	2,20	1,16	1	51 413 234
3	D	1456617_a_at	BM210677	Eif2s2	eukaryotic translation initiation factor 2, subunit 2 (beta)	-0,42	2,25	1,17	2	154 562 858
3	D	1439816_at	BM120676	LOC433270		-1,35	3,08	1,73	1	
3	D	1443940_at	BB278297	Lrrc22	leucine rich repeat containing 22	0,71	2,24	0,52	14	35 979 289
3	D	1447122_at	BM218697			-1,37	2,06	1,25	1	87 908 800
3	D	1447961_s_at	BI135190	Mrpl38	mitochondrial ribosomal protein L38	0,96	2,14	2,10	11	115 947 911
3	D	1455131_at	BB475273	Opa3	optic atrophy 3 (human)	0,44	2,12	3,01	7	18 386 910
3	D	1426500_at	BF462080	Icmt	isoprenylcysteine carboxyl methyltransferase	-0,32	2,01	0,87	4	151 141 150
3	D	1456597_at	AV324623	Heatr3	HEAT repeat containing 3	0,00	2,19	1,42	8	91 027 989
3	D	1418746_at	NM_019999	Pnkd	paroxysmal nonkinesiogenic dyskinesia	1,42	2,39	1,49	1	74 218 240
3	D	1443750_s_at	R75260	Rpp40	ribonuclease P 40 subunit (human)	0,61	2,10	1,61	13	35 900 936
3	D	1427254_at	BI729900	Zfp445	zinc finger protein 445	-0,47	2,08	0,87	9	122 699 720
3	D	1441682_s_at	C79645	Xpot	exportin, tRNA (nuclear export receptor for tRNAs)	-1,04	2,01	1,93	10	
3	D	1459976_s_at	AV261043	Sod1	superoxide dismutase 1, soluble	-0,41	2,22	2,04	16	90 109 600
3	D	1417010_at	NM_013915	Zfp238	zinc finger protein 238	0,37	2,15	1,69	1	179 281 335
3	D	1429384_at	BM195380	Csnk1g3	casein kinase 1, gamma 3	1,25	2,25	0,90	18	54 032 011
3	D	1431199_at	BG271102	0610031G08Rik	RIKEN cDNA 0610031G08 gene	0,64	2,08	2,36	17	
3	D	1423790_at	BC024876	Dap	death-associated protein	1,47	2,04	2,08	15	31 169 021
3	D	1435364_at	BB323901	Cyhr1	cysteine and histidine rich 1	0,08	2,10	1,47	15	76 473 022
3	D	1451624_a_at	BC025612	Phospho2	phosphatase, orphan 2	0,16	2,27	0,72	2	69 590 575
3	D	1442071_at	BG063303	Abce1	ATP-binding cassette, sub-family E (OABP), member 1	-0,52	2,14	1,26	8	82 579 716
3	D	1415914_at	NM_010448	Hnrpab	heterogeneous nuclear ribonucleoprotein A/B	0,01	2,00	1,23	11	51 443 522
3	D	1428248_at	AK005038	Nfx1	nuclear transcription factor, X-box binding 1	0,74	2,08	2,11	4	41 159 613
3	D	1440675_at	AW556148	Grlf1	glucocorticoid receptor DNA binding factor 1	0,83	2,01	0,96	7	15 653 560
3	D	1448673_at	NM_021495	Pvrl3	poliovirus receptor-related 3	-1,24	2,08	3,04	16	46 367 420
3	D	1452434_s_at	AF021031	Dgcr6	DiGeorge syndrome critical region gene 6	0,40	2,05	1,23	16	17 966 422
3	D	1418601_at	NM_011921	Aldh1a7	aldehyde dehydrogenase family 1, subfamily A7	1,44	2,15	1,51	19	20 760 049
3	D	1441110_at	BB280837	Lrrc21	leucine rich repeat containing 21	0,90	2,11	1,06	14	35 966 075
3	D	1420617_at	NM_026252	Cpeb4	cytoplasmic polyadenylation element binding protein 4	1,42	2,07	0,93	11	31 772 213
3	D	1447167_at	BB713397	AL033314	expressed sequence AL033314	0,89	2,25	1,89	6	28 430 357
3	D	1448358_s_at	NM_026506	Snrpg	small nuclear ribonucleoprotein polypeptide G	-0,05	2,06	2,61	6	86 337 173
3	D	1423418_at	BI247584	Fdps	farnesyl diphosphate synthetase	1,41	2,04	2,54	3	89 179 517
3	D	1439780_at	BG071958	Rpl7l1	ribosomal protein L7-like 1	-0,25	2,17	1,68	17	46 237 097
3	D	1449677_s_at	C77858	Tmem38b	transmembrane protein 38B	-1,40	2,03	2,99	4	53 847 161
3	D	1446007_at	BM231135	Bmp1	bone morphogenetic protein 1	-1,42	2,20	2,35	14	69 209 639
3	D	1448950_at	NM_008362	Il1r1	interleukin 1 receptor, type I	0,65	2,08	0,98	1	40 169 625
3	D	1448666_s_at	AV174616	Tob2	transducer of ERBB2, 2	-1,69	2,06	1,13	15	81 675 524
4	A	1424759_at	BC017528	Arrdc4	arrestin domain containing 4	-3,53	-2,88	-0,64	7	68 610 507
4	A	1424175_at	BC017689	Tef	thyrotroph embryonic factor	-3,87	-1,99	0,45	15	81 638 727
4	A	1423147_at	AW551830	Mat1a	methionine adenosyltransferase I, alpha	-3,66	-1,82	-1,03	14	40 014 093
4	A	1442151_at	BM238456	D230040A04Rik	RIKEN cDNA D230040A04 gene	-3,88	-4,06	-0,10	13	13 860 001
4	A	1419737_a_at	NM_010699	Ldha	lactate dehydrogenase A	-2,76	-1,80	-0,57	7	46 715 254
4	A	1459363_at	AV318787	Atxn2	ataxin 2	-2,34	-3,20	0,59	5	121 972 225
4	A	1440134_at	AI647584	Cyp4a10	cytochrome P450, family 4, subfamily a, polypeptide 10	-4,30	-2,65	-0,80	4	115 016 232
4	A	1435017_at	AV000569	Mel13	melanoma nuclear protein 13	-3,23	-2,28	0,06	11	97 501 677
4	A	1423257_at	AI327006	Cyp4a14	cytochrome P450, family 4, subfamily a, polypeptide 14	-9,21	-3,09	0,15	4	114 984 077
4	A	1451190_a_at	BC025837	Sbk1	SH3-binding kinase 1	-3,59	-2,39	-1,10	7	126 063 766
4	A	1456541_x_at	BB357220	Atad3a	ATPase family, AAA domain containing 3A	-3,36	-1,81	0,06	4	154 584 440
4	A	1448398_s_at	NM_009079	Rpl22	ribosomal protein L22	-2,08	-1,54	0,30	4	151 169 677
4	A	1441445_at	AW553065	Per3	period homolog 3 (Drosophila)	-2,43	-2,80	0,61	4	149 848 511
4	A	1416444_at	NM_019423	Elovl2	elongation of very long chain fatty acids (FEN1/Elo2, SUR4/Elo3, yeast)-like 2	-3,06	-1,89	-0,11	13	41 193 472
4	A	1453817_at	AK018242	Abca6	ATP-binding cassette, sub-family A (ABC1), member 6	-2,17	-1,65	-1,21	11	109 992 913
4	A	1424853_s_at	BC010747	Cyp4a10	cytochrome P450, family 4, subfamily a, polypeptide 10	-6,76	-2,45	-0,31	4	115 016 232
4	A	1427345_a_at	AK002700	Sult1a1	sulfotransferase family 1A, phenol-preferring, member 1	-2,98	-3,19	-0,59	7	126 464 018
4	A	1458176_at	BB021263	Per3	period homolog 3 (Drosophila)	-2,30	-2,46	-0,48	4	149 848 511
4	A	1420541_at	NM_009040	Rdh16	retinol dehydrogenase 16	-4,37	-2,35	0,72	10	127 204 102
4	A	1424969_s_at	BC027189	Upp2	uridine phosphorylase 2	-2,72	-1,58	-0,26	2	58 570 570
4	A	1453303_at	BI076733	4833417J20Rik	RIKEN cDNA 4833417J20 gene	-4,18	-1,82	0,23	19	
4	A	1436098_at	BB667452	Bche	butyrylcholinesterase	-3,19	-2,40	-0,35	3	73 721 733
4	A	1434613_at	BM230552	1810013L24Rik	RIKEN cDNA 1810013L24 gene	-2,72	-2,12	-0,45	16	

4	A	1436504_x_at	AV027367	Apoa4	apolipoprotein A-IV	-2,05	-3,09	-0,96	9	45 991 838
4	A	1435985_at	BM232304	Farp2	FERM, RhoGEF and pleckstrin domain protein 2	-2,03	-1,73	0,35	1	95 342 530
4	A	1452173_at	AW107842	Hadha	hydroxyacyl-Coenzyme A dehydrogenase/3-ketoacyl-Coenzyme A thiolase/enoyl-Coenzyme A hydratase (trifunctional protein), alpha subunit	-2,08	-1,58	0,82	5	30 449 090
4	A	1419367_at	NM_026172	Decr1	2,4-dienoyl CoA reductase 1, mitochondrial	-2,30	-1,86	0,24	4	15 844 387
4	A	1452648_at	AK010925	Tbrg1	transforming growth factor beta regulated gene 1	-2,44	-1,75	-1,11	9	37 398 852
4	A	1426522_at	BG866501	Hadhb	hydroxyacyl-Coenzyme A dehydrogenase/3-ketoacyl-Coenzyme A thiolase/enoyl-Coenzyme A hydratase (trifunctional protein), beta subunit	-3,19	-1,90	0,22	5	30 486 108
4	A	1452124_at	BB628935	Ank3	ankyrin 3, epithelial	-2,01	-2,25	0,54	10	69 320 888
4	A	1423495_at	BE952632	Decr2	2-4-dienoyl-Coenzyme A reductase 2, peroxisomal	-2,46	-2,37	-1,48	17	25 808 810
4	A	1448900_at	NM_138583	D16H22S680E	DNA segment, Chr 16, human D22S680E, expressed	-2,43	-1,67	0,11	16	18 214 389
4	A	1438629_x_at	AV166504	Grn	granulin	-3,02	-1,81	-0,75	11	102 246 596
4	A	1430614_at	BG962576	4632415K11Rik	RIKEN cDNA 4632415K11 gene	-2,30	-1,70	-0,11	8	122 646 563
4	A	1435031_at	AV069499	2010310D06Rik	RIKEN cDNA 2010310D06 gene	-3,18	-1,76	-0,82	5	136 020 116
4	A	1415984_at	NM_007382	Acadm	acyl-Coenzyme A dehydrogenase, medium chain	-2,97	-1,80	0,46	3	153 859 744
4	A	1451322_at	BC024580	2310016A09Rik	RIKEN cDNA 2310016A09 gene	-2,22	-2,51	-0,29	15	31 513 496
4	A	1437398_a_at	BB703752	Aldh9a1	aldehyde dehydrogenase 9, subfamily A1	-3,22	-1,80	-0,26	1	169 186 887
4	A	1452973_at	AK013741	Ppm1k	protein phosphatase 1K (PP2C domain containing)	-2,48	-2,16	-0,34	6	57 436 079
4	A	1428976_at	AK017463	Tmpo	thymopoietin	-2,14	-2,07	0,31	10	90 591 556
4	A	1441823_at	BB164673	Rai17	retinoic acid induced 17	-2,25	-2,63	-0,71	14	24 292 475
4	A	1438156_x_at	BB119196	Cpt1a	carnitine palmitoyltransferase 1a, liver	-2,95	-1,94	-0,69	19	3 323 347
4	A	1419031_at	NM_019699	Fads2	fatty acid desaturase 2	-3,62	-2,16	0,34	19	10 131 209
4	A	1426065_a_at	BC012955	Trib3	tribbles homolog 3 (Drosophila)	-2,23	-1,85	1,04	2	152 028 865
4	A	1436934_s_at	AU019938	Aco2	aconitase 2, mitochondrial	-2,14	-1,82	-1,33	15	81 699 717
4	A	1452264_at	BI408679	Tenc1	tensin like C1 domain-containing phosphatase	-2,07	-2,09	0,84	15	101 930 263
4	A	1453081_at	AA204410	2410022M11Rik	RIKEN cDNA 2410022M11 gene	-2,15	-1,70	0,23	14	
4	A	1431213_a_at	BG297038	1300007C21Rik	RIKEN cDNA 1300007C21 gene	-2,24	-1,72	0,99	18	
4	A	1450714_at	BE626090	Azin1	antizyme inhibitor 1	-2,09	-1,71	-0,37	15	38 432 018
4	A	1455869_at	BG862223	Camk2b	calcium/calmodulin-dependent protein kinase II, beta	-2,21	-2,55	1,28	11	5 869 674
4	A	1434817_s_at	BM206427	4930535B03Rik	RIKEN cDNA 4930535B03 gene	-2,44	-1,65	-1,04	3	95 857 648
4	A	1456225_x_at	BB508622	Trib3	tribbles homolog 3 (Drosophila)	-2,07	-1,96	0,73	2	152 028 865
4	A	1460603_at	BB145092	Samd9l	sterile alpha motif domain containing 9-like	-2,65	-3,46	0,02	6	
4	A	1435691_at	BB209207	C630028N24Rik	RIKEN cDNA C630028N24 gene	-2,16	-2,22	-0,67	9	54 715 419
4	A	1458177_at	BB048420	Ebpl	emopamil binding protein-like	-2,05	-2,90	1,20	14	60 294 870
4	A	1418116_at	NM_022329	Ifrg15	interferon alpha responsive gene	-2,17	-1,91	0,05	1	157 803 134
4	A	1450504_a_at	NM_053014	Agpat3	1-acylglycerol-3-phosphate O-acyltransferase 3	-1,93	-2,09	0,08	10	77 674 691
4	A	1453255_at	AK011417	Slc43a1	solute carrier family 43, member 1	-1,90	-2,66	0,00	2	
4	A	1417761_at	BC010769	Apoa4	apolipoprotein A-IV	-1,68	-2,33	-0,65	9	45 991 838
4	A	1451628_a_at	BC021657	Ank3	ankyrin 3, epithelial	-1,89	-2,36	-0,17	10	69 320 888
4	A	1443516_at	BE953583	Atxn2	ataxin 2	-1,67	-2,21	0,54	5	121 972 225
4	A	1430029_a_at	AK012853	Tspan31	tetraspanin 31	-1,66	-2,54	-1,29	10	126 470 240
4	A	1416383_a_at	NM_008797	Pcx	pyruvate carboxylase	-1,53	-2,49	-0,34	19	4 594 357
4	A	1423157_at	AK008566	Gnpnat1	glucosamine-phosphate N-acetyltransferase 1	-1,88	-2,94	-0,67	14	44 298 297
4	A	1446626_at	BM233202	D16H22S680E	DNA segment, Chr 16, human D22S680E, expressed	-1,68	-2,26	-0,85	16	18 214 389
4	A	1426464_at	W13191	Nr1d1	nuclear receptor subfamily 1, group D, member 1	-1,53	-3,06	-0,24	11	98 584 024
4	A	1436841_at	AV229336	B230380D07Rik	RIKEN cDNA B230380D07 gene	-1,63	-2,35	-1,32	9	70 403 759
5	A	1421041_s_at	NM_008182	Gsta2	glutathione S-transferase, alpha 2 (Yc2)	8,83	9,10	1,32	9	78 116 705
5	A	1451204_at	BC016096	Scara5	scavenger receptor class A, member 5 (putative)	5,47	3,38	2,78	14	64 620 536
5	A	1444296_a_at	BF383739	Serpina4ps1	serine (or cysteine) peptidase inhibitor, clade A, member 4, pseudogene 1	5,66	3,51	1,58	12	104 487 078
5	A	1416368_at	NM_010357	Gsta4	glutathione S-transferase, alpha 4	4,64	5,99	4,35	9	77 977 715
5	A	1436162_at	BB667865	C730048C13Rik	RIKEN cDNA C730048C13 gene	4,03	2,38	2,15	19	8 402 667
5	A	1448092_x_at	AA267743	Serpina4ps1	serine (or cysteine) peptidase inhibitor, clade A, member 4, pseudogene 1	5,76	5,59	2,08	12	104 486 879
5	A	1448566_at	AF226613	Slc40a1	solute carrier family 40 (iron-regulated transporter), member 1	3,18	4,89	3,94	1	45 852 629
5	A	1444297_at	BF383739	Serpina4ps1	serine (or cysteine) peptidase inhibitor, clade A, member 4, pseudogene 1	3,86	3,21	1,26	12	104 487 078
5	A	1424296_at	BC019374	Gclc	glutamate-cysteine ligase, catalytic subunit	3,68	4,74	3,24	9	77 540 493
5	A	1416833_at	NM_029550	Keg1	kidney expressed gene 1	3,62	2,78	2,63	19	12 762 811
5	A	1427474_s_at	J03953	Gstm3	glutathione S-transferase, mu 3	3,04	4,04	2,56	3	108 091 752
5	A	1417766_at	NM_025558	Cyb5b	cytochrome b5 type B	3,50	4,24	1,54	8	110 039 789
5	A	1454690_at	BB147462	Ikbkg	inhibitor of kappaB kinase gamma	2,97	4,46	0,48	X	70 685 524
5	A	1440327_at	AA985897	Cyp2c70	cytochrome P450, family 2, subfamily c, polypeptide 70	5,55	4,70	4,17	19	40 206 672
5	A	1439624_at	AA572504	Ugt2b35	UDP glucuronosyltransferase 2 family, polypeptide B35	2,56	3,91	0,78	5	88 075 420

5	A	1442005_at	BB018051	AW987390	expressed sequence AW987390	2,79	3,36	4,40	4	
5	A	1423846_x_at	BC008117	Tuba2	tubulin, alpha 2	2,33	2,91	2,18	15	98 759 463
5	A	1437654_at	BE630700	3110001K24Rik	RIKEN cDNA 3110001K24 gene	2,18	5,16	2,98	14	33 066 132
5	A	1427473_at	J03953	Gstm3	glutathione S-transferase, mu 3	2,29	4,15	2,22	3	108 091 752
5	A	1450715_at	NM_009993	Cyp1a2	cytochrome P450, family 1, subfamily a, polypeptide 2	3,14	2,60	3,49	9	57 475 073
5	A	1428805_at	AI854658	Slc35e3	solute carrier family 35, member E3	3,02	4,07	0,59	10	117 136 671
5	A	1417767_at	NM_025558	Cyb5b	cytochrome b5 type B	2,48	3,86	0,89	8	110 039 789
5	A	1419582_at	NM_028089	Cyp2c55	cytochrome P450, family 2, subfamily c, polypeptide 55	3,94	6,22	4,43	19	39 072 062
5	A	1430307_a_at	AK006387	Mod1	malic enzyme, supernatant	2,34	4,10	1,07	9	86 378 093
5	A	1460256_at	NM_007606	Car3	carbonic anhydrase 3	2,41	1,76	2,56	3	14 840 281
5	A	1455959_s_at	AW825835	Gclc	glutamate-cysteine ligase, catalytic subunit	2,06	3,56	1,58	9	77 540 493
5	A	1423462_at	BM213179	Map3k7ip2	mitogen-activated protein kinase kinase kinase 7 interacting protein 2	2,17	2,25	2,33	10	7 596 330
5	A	1419703_at	NM_016919	Col5a3	procollagen, type V, alpha 3	2,16	1,97	2,66	9	20 520 451
5	A	1417061_at	AF226613	Slc40a1	solute carrier family 40 (iron-regulated transporter), member 1	1,81	3,85	1,79	1	45 852 629
5	A	1422438_at	NM_010145	Ephx1	epoxide hydrolase 1, microsomal	1,60	4,01	0,81	1	182 826 232
4	B	1421422_at	NM_138654	AF397014	cDNA sequence AF397014	-8,61	-7,41	2,11	13	16 642 615
4	B	1451798_at	M57525	Il1rn	interleukin 1 receptor antagonist	-5,02	-3,09	1,57	2	24 167 426
4	B	1455316_x_at	AI987693	Kcnh6	potassium voltage-gated channel, subfamily H (eag-related), member 6	-4,39	-2,73	2,62	11	105 824 292
4	B	1448715_x_at	NM_010490	Ccrn4l	CCR4 carbon catabolite repression 4-like (S. cerevisiae)	-3,19	-1,72	1,91	3	51 312 375
4	B	1418174_at	BC018323	Dbp	D site albumin promoter binding protein	-3,09	-2,07	1,72	7	45 573 273
4	B	1438211_s_at	BB550183	Dbp	D site albumin promoter binding protein	-2,82	-2,03	1,66	7	45 573 273
4	B	1449025_at	NM_010501	Ifit3	interferon-induced protein with tetratricopeptide repeats 3	-3,07	-2,62	3,27	19	34 649 550
4	B	1417883_at	BC012707	Gstt2	glutathione S-transferase, theta 2	-2,95	-1,56	1,99	10	75 275 601
4	B	1426278_at	AY090098	Ifi27	interferon, alpha-inducible protein 27	-3,28	-3,59	1,54	12	103 843 218
4	B	1450672_a_at	NM_011637	Trex1	three prime repair exonuclease 1	-2,63	-2,30	2,58	9	108 915 337
4	B	1418645_at	L07645	Hal	histidine ammonia lyase	-2,29	-2,76	1,96	10	92 918 566
4	B	1448898_at	AF128196	Ccl9	chemokine (C-C motif) ligand 9	-2,12	-1,86	5,52	11	83 389 111
4	B	1417602_at	AF035830	Per2	period homolog 2 (Drosophila)	-2,37	-1,70	1,47	1	93 246 389
4	B	1417244_a_at	NM_016850	Irf7	interferon regulatory factor 7	-2,80	-2,80	3,19	7	141 114 508
4	B	1453939_x_at	AK019325	LOC677168		-2,35	-1,84	1,60	17	
4	B	1418191_at	NM_011909	Usp18	ubiquitin specific peptidase 18	-4,61	-4,55	4,74	6	121 211 575
4	B	1421917_at	AW537708	Pdgfra	platelet derived growth factor receptor, alpha polypeptide	-2,31	-2,19	1,57	5	75 434 040
4	B	1457644_s_at	BB554288	Cxcl1	chemokine (C-X-C motif) ligand 1	-2,38	-2,40	2,04	5	91 966 509
4	B	1420915_at	AW214029	Stat1	signal transducer and activator of transcription 1	-2,41	-1,98	3,26	1	52 064 001
4	B	1450783_at	NM_008331	Ifit1	interferon-induced protein with tetratricopeptide repeats 1	-3,42	-2,29	3,67	19	34 706 885
4	B	1425156_at	BC010229	Gbp7	guanylate binding protein 7	-3,13	-1,74	3,65	3	142 467 727
4	B	1447845_s_at	AV360029	Vnn1	vanin 1	-4,27	-2,73	2,69	10	23 584 275
4	B	1445597_s_at	BB404920	Hrasls3	HRAS like suppressor 3	-2,07	-1,80	3,16	19	7 624 502
4	B	1424339_at	AB067533	Oasl1	2'-5' oligoadenylate synthetase-like 1	-2,20	-2,55	2,55	5	115 184 238
4	B	1437503_a_at	BB533076	Scotin	scotin gene	-2,36	-2,10	2,54	9	108 896 032
4	B	1418486_at	NM_011704	Vnn1	vanin 1	-3,63	-2,06	2,42	10	23 584 275
4	B	1418580_at	BC024872	Rtp4	receptor transporter protein 4	-3,43	-3,19	6,75	16	23 525 275
4	B	1417292_at	NM_008330	Ifi47	interferon gamma inducible protein 47	-2,16	-2,07	2,99	11	48 930 477
4	B	1423986_a_at	BC010238	Scotin	scotin gene	-2,09	-2,20	1,58	9	108 896 032
4	B	1431591_s_at	AK019325	LOC677168		-2,42	-1,65	3,14	17	
4	B	1451426_at	AF316999	D11Lgp2e	DNA segment, Chr 11, Lothar Hennighausen 2, expressed	-2,17	-3,04	2,16	11	100 510 974
4	B	1455500_at	AW556558	D11Ertd759e	DNA segment, Chr 11, ERATO Doi 759, expressed	-2,86	-2,11	3,86	11	119 296 890
4	B	1460318_at	NM_013808	Csrp3	cysteine and glycine-rich protein 3	-1,60	-3,00	2,25	7	48 698 418
4	B	1438676_at	BM241485	Mpa2l	macrophage activation 2 like	-1,96	-2,31	5,59	5	105 455 208
4	B	1451564_at	BC021340	Parp14	poly (ADP-ribose) polymerase family, member 14	-1,83	-2,49	4,00	16	35 752 626
4	C	1422645_at	AJ306425	Hfe	hemochromatosis	-7,96	-8,96	-2,34	13	23 711 307
4	C	1450970_at	AA792094	Got1	glutamate oxaloacetate transaminase 1, soluble	-4,72	-1,88	-3,26	19	43 553 065
4	C	1424715_at	BB775176	Retsat	retinol saturase (all trans retinol 13,14 reductase)	-4,87	-1,60	-3,12	6	72 528 136
4	C	1456611_at	BB482650	D430015B01Rik	RIKEN cDNA D430015B01 gene	-3,68	-3,11	-1,35	6	58 863 114
4	C	1421653_a_at	NM_134051	IghVJ558	immunoglobulin heavy chain (J558 family)	-3,73	-2,62	-1,44	12	113 706 525
4	C	1418300_a_at	NM_021462	Mknk2	MAP kinase-interacting serine/threonine kinase 2	-4,65	-2,10	-1,84	10	80 068 460
4	C	1435902_at	BM120193	Nudt18	nudix (nucleoside diphosphate linked moiety X)-type motif 18	-2,56	-2,11	-1,82	14	69 312 929
4	C	1448350_at	NM_133768	Asl	argininosuccinate lyase	-3,00	-2,41	-4,40	5	130 295 966
4	C	1419857_at	AA254866			-2,68	-2,23	-1,57	?	
4	C	1430319_at	BB667652	4833411C07Rik	RIKEN cDNA 4833411C07 gene	-2,55	-3,08	-1,73	8	10 899 866
4	C	1439293_at	BB369212	BC031353	cDNA sequence BC031353	-2,07	-2,50	-1,76	9	74 762 714

4	C	1424943_at	BC013476	BC013476	cDNA sequence BC013476	-4,97	-2,91	-2,18	4	115 016 247
4	C	1417077_at	NM_007530	Bcap29	B-cell receptor-associated protein 29	-2,89	-3,25	-2,43	12	32 181 119
4	C	1416153_at	NM_011899	Srp54	signal recognition particle 54	-3,27	-2,57	-3,67	12	55 998 584
4	C	1427370_at	AK005066	Amdhd1	amidohydrolase domain containing 1	-2,51	-1,99	-4,81	10	
4	C	1420984_at	AF114437	Pctp	phosphatidylcholine transfer protein	-3,94	-3,29	-1,55	11	89 800 681
4	C	1416250_at	NM_007570	Btg2	B-cell translocation gene 2, anti-proliferative	-3,44	-4,88	-4,52	1	135 891 275
4	C	1429144_at	AV291259	Prei4	preimplantation protein 4	-3,58	-2,45	-3,74	2	132 220 524
4	C	1424815_at	BC021322	Gys2	glycogen synthase 2	-2,23	-3,07	-1,65	6	142 379 835
4	C	1448111_at	NM_018737	Ctps2	cytidine 5'-triphosphate synthase 2	-2,48	-1,78	-2,30	X	158 245 709
4	C	1431980_a_at	AK009814	As3mt	arsenic (+3 oxidation state) methyltransferase	-2,97	-2,63	-3,50	19	46 760 783
4	C	1447380_at	BB797326	Amdhd1	amidohydrolase domain containing 1	-2,06	-2,18	-2,05	10	
4	C	1424745_at	BC024461	2900006B13Rik	RIKEN cDNA 2900006B13 gene	-2,04	-1,69	-1,56	11	51 428 225
4	C	1438169_a_at	BB009122	Frmd4b	FERM domain containing 4B	-2,04	-2,23	-2,05	6	97 252 330
4	C	1449853_at	NM_053196	Sfxn2	sideroflexin 2	-2,07	-2,16	-1,51	19	46 626 675
4	C	1420983_at	AF114437	Pctp	phosphatidylcholine transfer protein	-3,02	-3,77	-5,10	11	89 800 681
4	C	1425665_a_at	BC005543	Srp54	signal recognition particle 54	-2,61	-1,62	-2,78	12	55 998 584
4	C	1456295_at	BB304874	B230114P17Rik	RIKEN cDNA B230114P17 gene	-4,06	-4,26	-2,56	10	
4	C	1418915_at	NM_025962	Mmachc	methylmalonic aciduria cblC type, with homocystinuria	-2,13	-2,30	-1,71	4	116 200 366
4	C	1423523_at	BF687395	Aass	aminoadipate-semialdehyde synthase	-2,58	-1,54	-1,78	6	23 022 170
4	C	1436689_a_at	AV028069	Aldh9a1	aldehyde dehydrogenase 9, subfamily A1	-2,56	-1,56	-3,00	1	169 186 887
4	C	1416101_a_at	NM_015786	Hist1h1c	histone 1, H1c	-2,25	-6,84	-2,78	13	23 746 271
4	C	1419477_at	NM_053109	Clec2d	C-type lectin domain family 2, member d	-2,10	-2,38	-1,78	6	129 146 220
4	C	1416939_at	NM_026438	Ppa1	pyrophosphatase (inorganic) 1	-1,94	-2,36	-2,78	10	61 043 977
4	C	1418249_at	NM_007761	Crcp	calcitonin gene-related peptide-receptor component protein	-1,75	-2,02	-3,31	5	130 314 008
4	C	1430802_at	AK013097	H2Q8	histocompatibility 2, Q region locus 8	-1,80	-2,61	-2,71	17	35 002 151
4	C	1438385_s_at	BB068040	Gpt2	glutamic pyruvate transaminase (alanine aminotransferase) 2	-1,91	-2,55	-4,13	8	88 382 721
4	C	1442214_at	BG173293	Nfib	nuclear factor I/B	-1,94	-2,50	-1,67	4	81 766 961
4	C	1428083_at	AK018202	2310043N10Rik	RIKEN cDNA 2310043N10 gene	-1,73	-3,57	-2,15	19	
4	C	1437040_at	AV280878	Etnk2	ethanolamine kinase 2	-1,67	-4,45	-7,21	1	135 191 334
4	C	1426243_at	BC019483	Cth	cystathionase (cystathionine gamma-lyase)	-1,88	-2,12	-2,50	3	157 829 540
4	C	1452123_s_at	BG067753	Frmd4b	FERM domain containing 4B	-1,51	-2,84	-1,87	6	97 252 330
4	C	1424683_at	BC019494	1810015C04Rik	RIKEN cDNA 1810015C04 gene	-1,73	-2,33	-2,67	15	25 787 988
5	B	1457403_at	AV378018	9130409I23Rik	RIKEN cDNA 9130409I23 gene	6,14	7,42	-2,28	1	182 887 911
5	B	1430681_at	AI482548	Cryl1	crystallin, lamda 1	4,14	1,85	0,64	14	56 229 144
5	B	1419435_at	NM_009676	Aox1	aldehyde oxidase 1	4,05	3,73	-0,65	1	57 974 514
5	B	1426215_at	AF071068	Ddc	dopa decarboxylase	4,76	2,07	-1,87	11	11 714 105
5	B	1420722_at	BC016468	Elovl3	elongation of very long chain fatty acids (FEN1/Elo2, SUR4/Elo3, yeast)-like 3	3,47	1,99	-0,18	19	46 185 209
5	B	1421040_a_at	NM_008182	Gsta2	glutathione S-transferase, alpha 2 (Yc2)	3,97	3,79	0,28	9	78 116 705
5	B	1426382_at	AJ271833	Ppm1b	protein phosphatase 1B, magnesium dependent, beta isoform	3,04	2,96	-0,57	17	84 866 340
5	B	1447112_s_at	C85932	Cryl1	crystallin, lamda 1	3,70	3,36	-0,26	14	56 229 144
5	B	1453011_at	AK007603	Bdh2	3-hydroxybutyrate dehydrogenase, type 2	4,59	1,78	-0,72	3	135 218 678
5	B	1418547_at	NM_009364	Tfpi2	tissue factor pathway inhibitor 2	3,16	2,42	0,05	6	3 912 595
5	B	1453155_at	AK014282	Tmem50a	transmembrane protein 50A	2,79	1,66	1,00	4	134 169 925
5	B	1451675_a_at	M63244	Alas2	aminolevulinic acid synthase 2, erythroid	3,26	1,77	1,39	X	145 888 160
5	B	1435259_s_at	AW551717	Tmem141	transmembrane protein 141	2,18	1,68	0,32	2	25 442 112
5	B	1422072_a_at	NM_008184	Gstm6	glutathione S-transferase, mu 6	3,36	2,26	0,35	3	108 066 903
5	B	1455618_x_at	BI440178	Tspan33	tetraspanin 33	2,86	1,74	0,12	6	29 644 265
5	B	1454930_at	BB540721	Lrrc35	leucine rich repeat containing 35	2,62	2,54	0,66	9	42 163 313
5	B	1448230_at	NM_009462	Usp10	ubiquitin specific peptidase 10	2,07	2,35	-0,28	8	122 796 828
5	B	1423399_a_at	BG143658	Yaf2	YY1 associated factor 2	2,02	2,90	0,76	15	93 112 077
5	B	1416541_at	NM_009191	Clpb	ClpB caseinolytic peptidase B homolog (E. coli)	2,06	1,93	-0,20	7	101 537 588
5	B	1431183_at	AA109251	1700066M21Rik	RIKEN cDNA 1700066M21 gene	2,74	1,98	1,31	1	
5	B	1446892_at	AU041374	Lrrc16	leucine rich repeat containing 16	2,02	1,91	-1,08	13	24 020 125
5	B	1436164_at	BE685959	Slc30a1	solute carrier family 30 (zinc transporter), member 1	2,42	2,37	0,91	1	193 607 395
5	B	1438992_x_at	AV314773	Atf4	activating transcription factor 4	2,42	2,36	1,38	15	80 082 755
5	B	1418979_at	NM_134072	Akr1c14	aldo-keto reductase family 1, member C14	2,64	2,65	0,88	13	4 058 836
5	B	1425141_at	BE691552	Lactb2	lactamase, beta 2	2,36	1,82	-0,63	1	13 611 114
5	B	1451035_a_at	BG296124	Akr1a4	aldo-keto reductase family 1, member A4 (aldehyde reductase)	2,57	2,21	0,29	4	116 134 442
5	B	1460323_at	AK010256	Tars	threonyl-tRNA synthetase	2,46	2,26	0,28	15	11 328 387
5	B	1440576_at	AV375940	Cpn1	carboxypeptidase N, polypeptide 1	2,40	1,75	0,53	19	44 009 620
5	B	1457756_at	BB483373	Zfp192	zinc finger protein 192	2,78	1,84	-0,08	13	21 520 686

5	B	1423437_at	AI172943	Gsta3	glutathione S-transferase, alpha 3	2,44	2,24	-0,15	1	21 234 901
5	B	1424110_a_at	BC005629	Nme1	expressed in non-metastatic cells 1, protein	2,65	1,98	-1,05	11	93 775 016
5	B	1435917_at	BM937735			2,68	1,66	1,98	5	73 601 333
5	B	1417058_a_at	NM_025327	Krtcap2	keratinocyte associated protein 2	2,08	1,97	0,38	3	89 332 364
5	B	1435646_at	BB821318	Ikbkg	inhibitor of kappaB kinase gamma	2,01	3,75	0,65	X	70 685 524
5	B	1422183_a_at	NM_007416	Adra1b	adrenergic receptor, alpha 1b	2,29	1,55	0,85	11	43 618 028
5	B	1441214_at	BQ176550	Exph5	exophilin 5	2,61	1,88	-1,26	9	53 064 064
5	B	1448239_at	NM_010442	Hmox1	heme oxygenase (decycling) 1	5,27	2,40	-3,66	8	77 989 693
5	B	1439089_at	BM220854	Zbtb41	zinc finger and BTB domain containing 41 homolog	2,03	2,11	-0,27	1	141 238 795
5	B	1420654_a_at	NM_028803	Gbe1	glucan (1,4-alpha-), branching enzyme 1	2,34	1,88	0,25	16	70 196 775
5	B	1417741_at	NM_133198	Pygl	liver glycogen phosphorylase	2,29	1,90	-0,89	12	71 109 416
5	B	1423436_at	AI172943	Gsta3	glutathione S-transferase, alpha 3	2,32	3,25	0,68	1	21 234 901
5	B	1451386_at	BC027279	Blvrb	biliverdin reductase B (flavin reductase (NADPH))	2,50	3,06	-0,75	7	27 156 737
5	B	1439934_at	BB540543	Slc30a10	solute carrier family 30, member 10	2,08	1,59	1,48	1	187 155 635
5	B	1454964_at	BM210917	BC021395	cDNA sequence BC021395	2,43	1,69	0,00	18	24 627 971
5	B	1415983_at	NM_008879	Lcp1	lymphocyte cytosolic protein 1	1,90	2,49	-1,79	14	73 870 579
5	B	1428447_at	AK017734	Tmem14a	transmembrane protein 14A	1,91	2,01	0,06	1	21 203 781
5	B	1439050_at	BB258019	AI649393	expressed sequence AI649393	1,72	2,26	-1,45	3	122 259 918
5	B	1418627_at	NM_008129	Gclm	glutamate-cysteine ligase , modifier subunit	1,65	4,11	-1,05	3	122 237 613
5	B	1452919_a_at	W59405	1700012G19Rik	RIKEN cDNA 1700012G19 gene	1,97	2,39	0,45	17	24 198 072
5	B	1416537_at	NM_133930	Creld1	cysteine-rich with EGF-like domains 1	1,97	2,55	-0,12	6	113 449 345
5	B	1435258_at	AW551717	Tmem141	transmembrane protein 141	1,61	2,12	0,94	2	25 442 112
5	B	1416837_at	BC018228	Bax	Bcl2-associated X protein	1,95	2,28	1,14	7	45 329 743
5	B	1428779_at	BB526541	Zbtb41	zinc finger and BTB domain containing 41 homolog	1,64	2,08	0,18	1	141 238 795
5	B	1426123_a_at	AF273691	Rrbp1	ribosome binding protein 1	1,86	2,10	-1,31	2	
5	B	1459773_x_at	BB097174	Snip1	Smad nuclear interacting protein 1	1,82	2,55	1,08	4	124 568 996
5	B	1418334_at	NM_013726	Dbf4	DBF4 homolog (S. cerevisiae)	1,72	2,46	0,76	5	8 403 017
5	B	1418364_a_at	NM_010240	Ftl1	ferritin light chain 1	1,70	2,24	1,03	7	45 325 989
5	B	1439453_x_at	AV259141	1500026D16Rik	RIKEN cDNA 1500026D16 gene	1,58	3,36	0,56	19	5 601 872
5	B	1427347_s_at	BC003475	Tubb2a	tubulin, beta 2a	1,99	3,20	1,17	13	34 081 746
5	B	1451015_at	AI314476	Tkt	transketolase	1,94	3,75	0,32	14	29 378 157
5	B	1434332_at	AV168216	Zzz3	zinc finger, ZZ domain containing 3	1,62	2,70	0,85	3	152 334 079
5	B	1416308_at	NM_009466	Ugdh	UDP-glucose dehydrogenase	1,59	2,28	0,05	5	65 692 358
5	B	1435878_at	BB476811	Stk38l	serine/threonine kinase 38 like	1,56	2,89	-1,64	6	146 682 177
5	B	1449221_a_at	NM_133626	Rrbp1	ribosome binding protein 1	1,73	2,16	-1,64	2	
6	A	1424744_at	BC021950	Sds	serine dehydratase	-4,82	-1,34	-2,14	5	120 737 163
6	A	1419197_x_at	NM_032541	Hamp1	hepcidin antimicrobial peptide 1	-7,11	0,47	-2,11	7	30 651 130
6	A	1420405_at	NM_030687	Slco1a4	solute carrier organic anion transporter family, member 1a4	-3,75	-0,91	-2,06	6	141 767 845
6	A	1419196_at	NM_032541	Hamp1	hepcidin antimicrobial peptide 1	-6,27	0,28	-3,57	7	30 651 130
6	A	1436555_at	AV244175	Slc7a2	solute carrier family 7 (cationic amino acid transporter, y+ system), member 2	-3,87	-1,38	-2,62	8	42 361 228
6	A	1434216_a_at	BG070689	Nudt19	nudix (nucleoside diphosphate linked moiety X)-type motif 19	-3,47	0,49	-2,56	7	35 255 946
6	A	1424716_at	BB775176	Retsat	retinol saturase (all trans retinol 13,14 reductase)	-4,63	-1,56	-3,15	6	72 528 136
6	A	1449410_a_at	NM_013525	Gas5	growth arrest specific 5	-2,58	-0,63	-4,59	1	
6	A	1419291_x_at	NM_013525	Gas5	growth arrest specific 5	-2,65	-0,03	-2,91	1	
6	A	1434510_at	BF780807	Papss2	3'-phosphoadenosine 5'-phosphosulfate synthase 2	-3,17	-1,15	-3,89	19	32 686 001
6	A	1436506_a_at	AW988981	1110008H02Rik	RIKEN cDNA 1110008H02 gene	-3,19	1,51	-2,70	1	
6	A	1421921_at	BC011158	Serpina3m	serine (or cysteine) peptidase inhibitor, clade A, member 3M	-3,63	-0,73	-3,26	12	104 788 241
6	A	1419523_at	NM_007819	Cyp3a13	cytochrome P450, family 3, subfamily a, polypeptide 13	-3,14	-1,41	-8,17	5	138 122 720
6	A	1426921_at	AV309591	Abcf1	ATP-binding cassette, sub-family F (GCN20), member 1	-2,30	-0,81	-3,55	17	35 564 872
6	A	1417355_at	AB003040	Peg3	paternally expressed 3	-4,15	-0,90	-1,96	7	6 310 045
6	A	1455029_at	BB342219	Kif21a	kinesin family member 21A	-2,39	-1,37	-1,50	15	90 762 149
6	A	1418780_at	NM_018887	Cyp39a1	cytochrome P450, family 39, subfamily a, polypeptide 1	-2,60	-0,33	-2,68	17	43 130 479
6	A	1418490_at	NM_133902	Sdsl	serine dehydratase-like	-2,25	-1,49	-1,51	5	120 718 818
6	A	1426622_a_at	BB150720	Qpct	glutaminyl-peptide cyclotransferase (glutaminyl cyclase)	-2,23	-0,79	-2,91	17	78 956 963
6	A	1435896_at	BG066686	Sfxn2	sideroflexin 2	-2,38	-1,40	-2,30	19	46 626 675
6	A	1420810_at	NM_019769	1500003O03Rik	RIKEN cDNA 1500003O03 gene	-3,62	-0,85	-3,21	2	119 239 147
6	A	1456036_x_at	AV003026	Gsto1	glutathione S-transferase omega 1	-2,49	0,94	-3,40	19	47 908 299
6	A	1416452_at	BC008119	Oat	ornithine aminotransferase	-2,37	-1,09	-1,95	7	132 395 821
6	A	1424574_at	BC020076	Tmed5	transmembrane emp24 protein transport domain containing 5	-3,81	-0,96	-1,82	5	108 361 948
6	A	1420624_a_at	NM_016794	Vamp8	vesicle-associated membrane protein 8	-2,31	-1,10	-4,96	6	72 314 732
6	A	1460241_a_at	BB829192	St3gal5	ST3 beta-galactoside alpha-2,3-sialyltransferase 5	-2,37	0,17	-3,16	6	72 027 121

6	A	1450738_at	NM_016705	Kif21a	kinesin family member 21A	-2,61	-1,18	-3,78	15	90 762 149
6	A	1416408_at	AB034914	Acox1	acyl-Coenzyme A oxidase 1, palmitoyl	-2,10	-1,63	-4,27	11	115 987 978
6	B	1416933_at	NM_008898	Por	P450 (cytochrome) oxidoreductase	-6,37	-0,42	-0,82	5	135 973 840
6	B	1442513_at	AU041573	BC016423	cDNA sequence BC016423	-3,80	-0,01	0,12	13	
6	B	1433924_at	BM200248	Peg3	paternally expressed 3	-4,57	-1,09	-1,19	7	6 310 045
6	B	1415943_at	BI788645	Sdc1	syndecan 1	-3,43	-0,49	0,03	12	8 797 403
6	B	1460196_at	NM_007620	Cbr1	carbonyl reductase 1	-3,29	0,77	-0,86	16	93 496 378
6	B	1437279_x_at	BB533095	Sdc1	syndecan 1	-2,82	-1,30	-1,04	12	8 797 403
6	B	1417348_at	NM_025966	2310039H08Rik	RIKEN cDNA 2310039H08 gene	-3,38	-1,07	-0,02	17	46 235 825
6	B	1453587_at	BB503754	Ggt6	gamma-glutamyltransferase 6	-2,87	-0,65	-0,53	11	72 251 720
6	B	1438178_x_at	BB368056	Atad3a	ATPase family, AAA domain containing 3A	-2,85	-1,25	0,57	4	154 584 440
6	B	1456424_s_at	AI591480	Pltp	phospholipid transfer protein	-3,10	-1,15	0,76	2	164 530 722
6	B	1415944_at	BI788645	Sdc1	syndecan 1	-3,63	-0,70	0,27	12	8 797 403
6	B	1451681_at	BC018263	BC089597	cDNA sequence BC089597	-2,85	-0,17	0,44	10	127 269 436
6	B	1458309_at	BM122422	Dip2c	DIP2 disco-interacting protein 2 homolog C (Drosophila)	-3,31	-1,14	-0,23	13	
6	B	1419094_at	NM_010001	Cyp2c37	cytochrome P450, family 2. subfamily c, polypeptide 37	-3,26	0,08	0,29	19	40 045 750
6	B	1452649_at	AK003859	Rtn4	reticulon 4	-5,96	-1,63	-0,19	11	29 618 569
6	B	1436739_at	AI551199	Agtr1a	angiotensin II receptor, type 1a	-3,62	-1,08	0,14	13	30 343 921
6	B	1448987_at	BB728073	Acadl	acyl-Coenzyme A dehydrogenase, long-chain	-3,00	-1,39	0,59	1	66 764 060
6	B	1449457_at	AB078618	Acot12	acyl-CoA thioesterase 12	-2,90	-1,31	-0,47	13	92 215 759
6	B	1415867_at	NM_009837	Cct4	chaperonin subunit 4 (delta)	-2,68	-0,04	0,29	11	22 890 592
6	B	1429786_a_at	AK008144	Zwint	ZW10 interactor	-3,03	-0,41	-0,46	10	72 050 194
6	B	1448385_at	NM_133895	Slc15a4	solute carrier family 15, member 4	-2,11	-1,12	-0,24	5	127 884 869
6	B	1450420_at	BQ174473	Stag1	stromal antigen 1	-2,29	-1,42	-0,31	9	100 452 974
6	B	1450866_a_at	BQ179556	Mrpl17	mitochondrial ribosomal protein L17	-2,06	-0,27	0,60	7	105 677 602
6	B	1426288_at	AF247637	Lrp4	low density lipoprotein receptor-related protein 4	-2,20	0,26	0,07	2	91 258 539
6	B	1435207_at	BB758432	Dixdc1	DIX domain containing 1	-3,97	-1,11	-1,00	9	50 414 984
6	B	1447644_at	BB269821	Wnt5b	wingless-related MMTV integration site 5B	-2,18	-1,39	-0,84	6	119 398 152
6	B	1448491_at	NM_016772	Ech1	enoyl coenzyme A hydratase 1, peroxisomal	-2,63	-1,49	0,60	7	28 534 097
6	B	1450968_at	AK003966	Uqcrfs1	ubiquinol-cytochrome c reductase, Rieske iron-sulfur polypeptide 1	-2,62	-0,43	-0,33	13	30 547 776
6	B	1425616_a_at	BC002274	Ccdc23	coiled-coil domain containing 23	-2,18	0,03	-0,50	4	118 693 470
6	B	1424184_at	BC026559	Acadvl	acyl-Coenzyme A dehydrogenase, very long chain	-2,40	-0,36	0,07	11	69 826 388
6	B	1424359_at	BC025120	Oplah	5-oxoprolinase (ATP-hydrolysing)	-4,48	-0,74	0,84	15	76 123 858
6	B	1436884_x_at	BB699868	Ewsr1	Ewing sarcoma breakpoint region 1	-2,53	-0,80	0,63	11	4 969 690
6	B	1449325_at	NM_019699	Fads2	fatty acid desaturase 2	-3,55	-1,59	0,18	19	10 131 209
6	B	1458658_at	AW986533			-2,40	-0,01	0,03	16	13 584 899
6	B	1425921_a_at	BC019471	1810055G02Rik	RIKEN cDNA 1810055G02 gene	-4,06	-0,03	0,14	19	3 708 332
6	B	1419395_at	AB078618	Acot12	acyl-CoA thioesterase 12	-2,19	-0,80	-0,33	13	92 215 759
6	B	1426942_at	BM233292	Aim1	absent in melanoma 1	-2,97	-1,31	0,48	10	
6	B	1457248_x_at	BB554029	Hsd17b7	hydroxysteroid (17-beta) dehydrogenase 7	-3,57	-0,66	0,63	1	171 786 211
6	B	1452889_at	AK009207	2310007H09Rik	RIKEN cDNA 2310007H09 gene	-2,11	-0,92	-1,22	7	132 448 985
6	B	1428512_at	AK012577	Bhlhb9	basic helix-loop-helix domain containing, class B9	-2,38	-1,74	-1,60	X	131 235 265
6	B	1435679_at	BB770843	Optn	optineurin	-3,14	-0,86	0,25	2	4 937 915
6	B	1418321_at	NM_010023	Dci	dodecenoyl-Coenzyme A delta isomerase (3,2 trans-enoyl-Coenyme A isomerase)	-2,90	-1,24	0,44	17	24 154 282
6	B	1428636_at	AK015015	Steap2	six transmembrane epithelial antigen of prostate 2	-2,05	0,50	-0,14	5	
6	B	1415930_a_at	AU080586	Map1lc3b	microtubule-associated protein 1 light chain 3 beta	-2,24	-0,50	0,44	8	124 476 527
6	B	1433461_at	AV240527	Sf3b2	splicing factor 3b, subunit 2	-2,24	-0,54	-0,04	19	5 273 932
6	B	1430418_at	AK020354	Tmem57	transmembrane protein 57	-2,21	-0,79	0,67	4	134 074 838
6	B	1451738_at	AF363030	Ogt	O-linked N-acetylglucosamine (GlcNAc) transferase (UDP-N-acetylglucosamine:polypeptide-N-acetylglucosaminyl transferase)	-2,36	-0,97	-1,07	X	97 842 782
6	B	1419428_a_at	NM_008064	Gaa	glucosidase, alpha, acid	-2,21	-0,42	-1,13	11	119 084 133
6	B	1449945_at	NM_133249	Ppargc1b	peroxisome proliferative activated receptor, gamma, coactivator 1 beta	-3,54	-1,03	0,10	18	61 423 505
6	B	1417419_at	NM_007631	Ccnd1	cyclin D1	-2,10	0,01	0,31	7	144 739 320
6	B	1422100_at	NM_007824	Cyp7a1	cytochrome P450, family 7, subfamily a, polypeptide 1	-2,16	-0,87	0,79	4	6 192 758
6	B	1421987_at	BF786072	Papss2	3'-phosphoadenosine 5'-phosphosulfate synthase 2	-2,43	-1,11	-0,53	19	32 686 001
6	B	1435594_at	AV334690	Arl6ip2	ADP-ribosylation factor-like 6 interacting protein 2	-2,16	-1,59	-0,81	17	79 758 502
6	B	1417963_at	NM_011125	Pltp	phospholipid transfer protein	-2,36	-1,12	0,82	2	164 530 722
6	B	1434839_s_at	BG071620	Tbl1xr1	transducin (beta)-like 1X-linked receptor 1	-2,08	-1,10	0,15	3	22 267 856
6	B	1448915_at	NM_025324	Zfp524	zinc finger protein 524	-2,02	-0,49	-0,16	7	4 618 594
6	B	1425392_a_at	AF009328	Nr1i3	nuclear receptor subfamily 1, group I, member 3	-3,15	-1,02	0,45	1	173 050 644

6	B	1417598_a_at	NM_008053	Fxr1h	fragile X mental retardation gene 1, autosomal homolog	-2,47	-0,72	-0,21	3	34 211 656
6	B	1452046_a_at	BG071790	Ppp1cc	protein phosphatase 1, catalytic subunit, gamma isoform	-2,30	-1,32	-0,71	5	122 418 905
6	B	1417212_at	NM_026633	9530058B02Rik	RIKEN cDNA 9530058B02 gene	-2,77	-0,30	0,43	17	25 591 299
6	B	1451290_at	BC010596	Map1lc3a	microtubule-associated protein 1 light chain 3 alpha	-2,31	-0,11	-0,17	2	154 967 904
6	B	1423568_at	BG297088	Psma7	proteasome (prosome, macropain) subunit, alpha type 7	-2,85	0,00	0,32	2	179 965 783
6	B	1436016_x_at	BF660710	Gdi2	guanosine diphosphate (GDP) dissociation inhibitor 2	-2,14	-0,34	-0,27	13	3 537 392
6	B	1446950_at	BM124834	Tox	thymocyte selection-associated HMG box gene	-3,36	0,33	-0,19	4	6 614 604
6	B	1451084_at	BC012522	Etfdh	electron transferring flavoprotein, dehydrogenase	-2,48	-0,95	0,36	3	79 689 738
6	B	1455814_x_at	AV111502	Ddx39	DEAD (Asp-Glu-Ala-Asp) box polypeptide 39	-2,15	-0,66	-0,30	8	86 605 303
6	B	1426310_at	AV241525	Zdhhc5	zinc finger, DHHC domain containing 5	-2,01	0,18	-0,75	2	84 488 810
6	B	1460409_at	AI987925	Cpt1a	carnitine palmitoyltransferase 1a, liver	-2,94	-1,50	-0,26	19	3 323 347
6	B	1448188_at	BC012697	Ucp2	uncoupling protein 2 (mitochondrial, proton carrier)	-2,19	-0,83	0,91	7	100 367 445
6	B	1424951_at	BC015459	Baiap2l1	BAI1-associated protein 2-like 1	-2,49	-0,26	-0,36	5	144 518 051
6	B	1437180_at	BE824567	6530403A03Rik	RIKEN cDNA 6530403A03 gene	-2,18	-1,03	0,44	13	38 212 404
6	B	1423122_at	BI649826	Avpi1	arginine vasopressin-induced 1	-2,37	-0,91	-0,92	19	42 176 591
6	B	1416248_at	AW538068	Nadk	NAD kinase	-2,45	-1,30	-0,78	4	154 406 203
6	B	1453651_a_at	AV223468	Brp44l	brain protein 44-like	-2,05	0,05	0,03	17	8 121 986
6	B	1416738_at	NM_028227	Brap	BRCA1 associated protein	-2,34	-0,77	-0,82	5	121 921 202
6	B	1416980_at	NM_027853	Mettl7b	methyltransferase like 7B	-2,45	-1,20	-0,35	10	128 361 225
6	B	1428111_at	AK003626	Slc38a4	solute carrier family 38, member 4	-2,29	-0,88	-0,51	15	96 822 856
6	B	1455435_s_at	BG072100	Chdh	choline dehydrogenase	-2,11	0,21	-0,08	14	28 850 311
6	B	1428440_at	AK019150	Slc25a12	solute carrier family 25 (mitochondrial carrier, Aralar), member 12	-2,13	-1,26	-0,75	2	71 075 133
6	B	1436204_at	BM730637	1110059G02Rik	RIKEN cDNA 1110059G02 gene	-2,39	-0,18	0,32	15	
6	B	1456668_at	BB554044	Zcchc11	zinc finger, CCHC domain containing 11	-2,20	-1,07	0,44	4	107 957 357
6	B	1451548_at	BC027189	Upp2	uridine phosphorylase 2	-2,14	-1,00	0,01	2	58 570 570
6	B	1433666_s_at	AW536822	Vps41	vacuolar protein sorting 41 (yeast)	-2,63	-0,78	0,81	13	18 724 763
6	B	1449130_at	NM_007639	Cd1d1	CD1d1 antigen	-2,12	-0,83	-0,91	3	87 081 764
6	B	1423724_at	BC027100	Zwint	ZW10 interactor	-2,22	0,00	-0,26	10	72 050 194
6	B	1425057_at	BC019997	0610038K03Rik	RIKEN cDNA 0610038K03 gene	-2,05	-1,01	0,46	10	62 456 973
6	B	1417168_a_at	AI553394	Usp2	ubiquitin specific peptidase 2	-2,25	0,00	0,41	9	43 836 313
6	B	1427945_at	BC028831	Dpyd	dihydropyrimidine dehydrogenase	-2,51	-1,31	-0,54	3	118 554 190
6	B	1426999_at	BM198642	Zc3h14	zinc finger CCCH type containing 14	-2,09	0,07	-0,13	12	99 148 017
6	B	1415776_at	NM_007437	Aldh3a2	aldehyde dehydrogenase family 3, subfamily A2	-3,18	-1,08	0,17	11	61 060 949
6	B	1425484_at	BB547854	Tox	thymocyte selection-associated HMG box gene	-2,31	-0,75	-0,17	4	6 614 604
6	B	1416793_at	NM_019717	Arl6ip2	ADP-ribosylation factor-like 6 interacting protein 2	-2,82	-0,29	-1,16	17	79 758 502
6	B	1460575_at	BB667209	Eif2a	eukaryotic translation initiation factor 2a	-2,04	0,23	0,29	3	58 613 944
6	B	1437144_x_at	AV100134	Psma6	proteasome (prosome, macropain) subunit, alpha type 6	-2,03	-0,25	-0,08	12	56 317 026
6	B	1455204_at	BB305507	Pitpnc1	phosphatidylinositol transfer protein, cytoplasmic 1	-2,32	-1,11	0,78	11	107 023 981
6	B	1417560_at	BB478992	Sfxn1	sideroflexin 1	-2,05	-0,38	0,30	13	54 081 498
6	B	1437172_x_at	AV150884	Hadhb	hydroxyacyl-Coenzyme A dehydrogenase/3-ketoacyl-Coenzyme A thiolase/enoyl-Coenzyme A hydratase (trifunctional protein), beta subunit	-2,96	-0,96	0,91	5	30 486 108
6	B	1454698_at	BG075943	Ptplad1	protein tyrosine phosphatase-like A domain containing 1	-2,36	-0,23	-0,47	9	64 784 990
6	B	1421116_a_at	NM_024226	Rtn4	reticulon 4	-3,06	-0,63	-0,35	11	29 618 569
6	B	1420961_a_at	NM_028582	Ivns1abp	influenza virus NS1A binding protein	-2,26	-1,04	-0,02	1	153 106 715
6	B	1423017_a_at	NM_031167	Il1rn	interleukin 1 receptor antagonist	-2,09	-1,39	0,39	2	24 167 426
6	B	1417356_at	AB003040	Peg3	paternally expressed 3	-2,11	-0,89	-0,54	7	6 310 045
6	B	1448148_at	M86736	Grn	granulin	-2,88	-1,07	-0,21	11	102 246 596
6	B	1456812_at	AW456685	Abcd2	ATP-binding cassette, sub-family D (ALD), member 2	-3,08	0,33	-0,57	15	90 973 638
6	B	1435987_x_at	BM570681	1110059G02Rik	RIKEN cDNA 1110059G02 gene	-2,20	-0,53	0,38	15	
6	B	1450391_a_at	NM_011844	Mgll	monoglyceride lipase	-2,98	-1,20	0,78	6	88 690 468
6	B	1431789_s_at	AK014065	Tmed5	transmembrane emp24 protein transport domain containing 5	-2,12	-0,36	-1,04	5	108 361 948
6	B	1448865_at	NM_010476	Hsd17b7	hydroxysteroid (17-beta) dehydrogenase 7	-2,41	-1,05	-0,33	1	171 786 211
6	B	1443838_x_at	BB229957	Fads2	fatty acid desaturase 2	-2,81	-1,10	0,78	19	10 131 209
6	B	1418183_a_at	AB013464	Pscd1	pleckstrin homology, Sec7 and coiled-coil domains 1	-2,02	0,04	-0,38	11	117 980 265
6	B	1431940_at	AK016900	LOC74457		-2,09	-1,03	-0,39	5	
6	B	1435327_at	BG071867	AW112037	expressed sequence AW112037	-3,77	-1,08	0,11	1	
6	B	1460362_at	BC004803	2410001C21Rik	RIKEN cDNA 2410001C21 gene	-3,32	-0,81	0,64	2	172 131 782
6	B	1435901_at	AV226197	Usp40	ubiquitin specific peptidase 40	-2,16	-0,01	0,01	1	89 776 293
6	B	1422997_s_at	NM_134188	Acot2	acyl-CoA thioesterase 2	-2,23	-1,14	0,81	12	84 877 637
6	B	1428143_a_at	AK003207	Pnpla2	patatin-like phospholipase domain containing 2	-2,21	-0,53	-0,97	7	141 306 526
6	B	1421852_at	AF319542	Kcnk5	potassium channel, subfamily K, member 5	-2,17	-1,07	-0,98	14	18 930 753

6	B	1420809_a_at	NM_019769	1500003O03Rik	RIKEN cDNA 1500003O03 gene	-2,11	0,21	-0,42	2	119 239 147
6	B	1432332_a_at	AK008430	Nudt19	nudix (nucleoside diphosphate linked moiety X)-type motif 19	-2,02	0,09	-1,15	7	35 255 946
6	B	1431012_a_at	AK009478	Peci	peroxisomal delta3, delta2-enoyl-Coenzyme A isomerase	-2,67	-0,44	0,59	13	34 985 214
6	B	1452071_at	BE655147	Slc4a4	solute carrier family 4 (anion exchanger), member 4	-2,15	-0,57	0,22	5	89 961 857
6	B	1434866_x_at	BB021753	Cpt1a	carnitine palmitoyltransferase 1a, liver	-2,20	-1,56	-0,63	19	3 323 347
6	B	1426698_a_at	AK011521	Hnrpm	heterogeneous nuclear ribonucleoprotein M	-2,08	0,21	-0,33	17	33 252 926
6	B	1455789_x_at	BB453676	Hspa8	heat shock protein 8	-3,24	-0,70	-0,31	9	40 552 341
6	B	1416555_at	NM_007915	Ei24	etoposide induced 2.4 mRNA	-2,36	-0,85	-0,23	9	36 528 829
6	B	1448332_at	NM_023041	Pex19	peroxisome biogenesis factor 19	-2,20	-0,78	-0,79	1	173 963 446
6	B	1437449_at	BB818348	Rsad1	radical S-adenosyl methionine domain containing 1	-2,08	-0,27	0,18	11	94 355 888
6	B	1425351_at	BC011325	Srxn1	sulfiredoxin 1 homolog (S. cerevisiae)	-2,25	0,21	0,23	2	151 797 170
6	B	1423833_a_at	BC018324	Brp44	brain protein 44	-2,03	-0,37	-0,48	1	167 297 882
6	B	1424573_at	BC020076	Tmed5	transmembrane emp24 protein transport domain containing 5	-2,22	0,92	-1,10	5	108 361 948
6	B	1426785_s_at	BI411560	Mgll	monoglyceride lipase	-2,18	-0,80	0,85	6	88 690 468
6	B	1423358_at	AK007407	1810009K13Rik	RIKEN cDNA 1810009K13 gene	-2,17	-1,29	0,77	16	20 525 143
6	B	1441915_s_at	BB717485	2310076L09Rik	RIKEN cDNA 2310076L09 gene	-2,13	0,23	0,07	17	55 704 894
6	B	1450776_at	NM_018743	Agpat6	1-acylglycerol-3-phosphate O-acyltransferase 6 (lysophosphatidic acid acyltransferase, zeta)	-2,85	0,73	-0,41	8	24 638 490
6	B	1424826_s_at	BC024131	Mtss1	metastasis suppressor 1	-3,09	-0,60	0,69	15	58 773 330
6	B	1448382_at	NM_023737	Ehhadh	enoyl-Coenzyme A, hydratase/3-hydroxyacyl Coenzyme A dehydrogenase	-2,30	-0,74	0,16	16	21 675 275
6	B	1423396_at	AK018763	Agt	angiotensinogen (serpin peptidase inhibitor, clade A, member 8)	-2,14	-1,01	0,10	8	127 442 677
6	B	1456567_x_at	BB000455	Grn	granulin	-2,22	-1,13	-0,04	11	102 246 596
6	B	1429115_at	AK008077	2010003O02Rik	RIKEN cDNA 2010003O02 gene	-2,26	-1,46	0,42	4	
6	B	1423108_at	AV008091	Slc25a20	solute carrier family 25 (mitochondrial carnitine/acylcarnitine translocase), member 20	-2,20	-0,27	0,32	9	108 519 501
6	B	1425859_a_at	AB029146	Psmd4	proteasome (prosome, macropain) 26S subunit, non-ATPase, 4	-2,09	0,04	-0,91	3	95 118 108
6	B	1419553_a_at	NM_011231	Rabggtb	RAB geranylgeranyl transferase, b subunit	-2,03	-0,64	0,13	3	153 844 839
6	B	1423109_s_at	AV008091	Slc25a20	solute carrier family 25 (mitochondrial carnitine/acylcarnitine translocase), member 20	-2,09	-0,39	-0,52	9	108 519 501
6	B	1417449_at	NM_133240	Acot8	acyl-CoA thioesterase 8	-2,45	-1,12	0,23	2	164 483 973
6	B	1458383_at	BB026943	Idh2	isocitrate dehydrogenase 2 (NADP+), mitochondrial	-2,09	-1,24	-0,35	7	79 968 361
6	B	1425518_at	AK004874	Rapgef4	Rap guanine nucleotide exchange factor (GEF) 4	-3,16	1,21	-1,34	2	71 782 125
6	B	1454159_a_at	AK011784	Igfbp2	insulin-like growth factor binding protein 2	-2,73	-0,18	-1,21	1	72 757 709
6	B	1452837_at	AK021389	Lpin2	lipin 2	-2,16	1,66	-1,30	17	71 109 532
6	C	1419748_at	NM_011994	Abcd2	ATP-binding cassette, sub-family D (ALD), member 2	-6,17	-0,77	1,89	15	90 973 638
6	C	1438431_at	BB197269	Abcd2	ATP-binding cassette, sub-family D (ALD), member 2	-4,87	-0,18	1,73	15	90 973 638
6	C	1422660_at	AY052560	LOC671237		-3,32	-0,60	0,96	Un	
6	C	1424599_at	BC021946	Fgl1	fibrinogen-like protein 1	-5,64	-1,46	1,38	8	42 690 251
6	C	1420723_at	NM_011979	Vnn3	vanin 3	-3,67	-1,40	2,06	10	23 540 887
6	C	1432416_a_at	AK005498	Npm1	nucleophosmin 1	-3,04	0,28	1,64	11	33 052 510
6	C	1448540_a_at	BC021536	0610012G03Rik	RIKEN cDNA 0610012G03 gene	-3,40	-0,62	1,48	16	31 866 797
6	C	1441359_at	AV381417	9230115F04Rik	RIKEN cDNA 9230115F04 gene	-4,02	-1,27	1,45	4	
6	C	1420000_s_at	C81413	Igbp1	immunoglobulin (CD79A) binding protein 1	-3,01	-0,07	0,59	X	96 697 063
6	C	1435493_at	AV297961	Dsp	desmoplakin	-3,12	0,46	1,39	13	
6	C	1455457_at	AI256046	Cyp2c54		-3,02	-0,65	0,84	19	40 045 750
6	C	1438743_at	BB667338	Cyp7a1	cytochrome P450, family 7, subfamily a, polypeptide 1	-3,15	-0,64	2,03	4	6 192 758
6	C	1420776_a_at	NM_016709	Auh	AU RNA binding protein/enoyl-coenzyme A hydratase	-2,85	-1,20	1,02	13	52 847 553
6	C	1424251_a_at	BC021374	Hnrpdl	heterogeneous nuclear ribonucleoprotein D-like	-2,58	-0,11	0,84	5	100 275 022
6	C	1421430_at	NM_009014	Rad51l1	RAD51-like 1 (S. cerevisiae)	-2,66	-0,23	1,57	12	80 216 121
6	C	1417691_at	NM_021328	Bin3	bridging integrator 3	-2,34	0,58	1,46	14	68 835 224
6	C	1424740_at	BG070002	Creb3	cAMP responsive element binding protein 3	-2,55	0,05	0,68	4	43 583 733
6	C	1455010_at	BE859736	1500012F01Rik	RIKEN cDNA 1500012F01 gene	-3,52	0,30	0,81	2	
6	C	1429178_at	BB472509	Odz3	odd Oz/ten-m homolog 3 (Drosophila)	-2,35	-0,46	2,96	8	49 726 507
6	C	1447936_at	AA939619	2410006H16Rik	RIKEN cDNA 2410006H16 gene	-2,79	-0,17	1,06	11	62 419 091
6	C	1451680_at	BC011325	Srxn1	sulfiredoxin 1 homolog (S. cerevisiae)	-2,84	0,65	0,49	2	151 797 170
6	C	1423803_s_at	BC017637	Gltscr2	glioma tumor suppressor candidate region gene 2	-2,72	-0,36	1,20	7	15 096 356
6	C	1417568_at	BG071381	Ncald	neurocalcin delta	-2,16	-0,54	1,25	15	37 310 763
6	C	1429809_at	AK018506	Tmtc2	transmembrane and tetratricopeptide repeat containing 2	-3,76	-0,20	1,67	10	104 591 773
6	C	1459238_at	AV319481	Pln	phospholamban	-2,93	1,57	2,67	10	53 026 121
6	C	1442916_at	BB202300	Psd3	pleckstrin and Sec7 domain containing 3	-2,56	-0,20	2,67	8	70 625 871
6	C	1425645_s_at	AF128849	Cyp2b10	cytochrome P450, family 2, subfamily b, polypeptide 10	-2,39	1,13	1,52	7	25 606 435

6	C	1417043_at	NM_008490	Lcat	lecithin cholesterol acyltransferase	-2,16	-0,37	1,71	8	108 828 681
6	C	1425079_at	BC024498	Tm6sf2	transmembrane 6 superfamily member 2	-2,64	0,07	1,66	8	73 001 920
6	C	1438024_at	AW554392	6230416A05Rik	RIKEN cDNA 6230416A05 gene	-2,58	0,18	0,58	13	
6	C	1425752_at	AJ132857	BC014805	cDNA sequence BC014805	-3,18	0,33	2,77	19	7 849 028
6	C	1417008_at	BC006668	Crat	carnitine acetyltransferase	-3,24	0,13	1,79	2	30 222 484
6	C	1452725_a_at	AK010292	Rnaseh2a	ribonuclease H2, large subunit	-2,02	1,37	0,01	8	87 846 714
6	C	1418081_at	NM_025362	Wbscr18	Williams-Beuren syndrome chromosome region 18 homolog (human)	-2,70	-0,91	0,97	5	135 348 828
6	C	1423567_a_at	BG297088	Psma7	proteasome (prosome, macropain) subunit, alpha type 7	-2,54	0,44	1,06	2	179 965 783
6	C	1430271_x_at	AA672926	Josd3	Josephin domain containing 3	-2,40	-0,10	0,96	9	15 056 655
6	C	1451626_x_at	U58494			-2,26	-0,20	1,93	X	
6	C	1451787_at	AF128849	Cyp2b10	cytochrome P450, family 2, subfamily b, polypeptide 10	-2,20	1,10	1,55	7	25 606 435
6	C	1455572_x_at	AI415136	Rps18	ribosomal protein S18	-2,27	0,63	1,59	17	33 562 452
6	C	1417900_a_at	NM_013703	Vldlr	very low density lipoprotein receptor	-3,04	-0,19	2,67	19	27 284 528
6	C	1443889_at	AI789751	9030619P08Rik	RIKEN cDNA 9030619P08 gene	-3,31	-0,97	2,73	15	75 254 861
6	C	1419547_at	BC026949	Fahd1	fumarylacetoacetate hydrolase domain containing 1	-2,07	-0,04	1,56	17	24 576 498
6	C	1452939_a_at	AI595920	Pitpnc1	phosphatidylinositol transfer protein, cytoplasmic 1	-2,08	-0,17	0,92	11	107 023 981
6	C	1459740_s_at	AW108044	Ucp2	uncoupling protein 2 (mitochondrial, proton carrier)	-2,19	-0,23	1,83	7	100 367 445
6	C	1454786_at	AV327520	5031439G07Rik	RIKEN cDNA 5031439G07 gene	-2,27	-0,37	1,98	15	84 773 485
6	C	1421382_at	NM_008932	Prlr	prolactin receptor	-2,72	-0,04	2,20	15	10 122 379
6	C	1431166_at	BF319155	Chd1	chromodomain helicase DNA binding protein 1	-2,48	0,08	2,09	17	15 409 979
6	C	1438258_at	BE647363	Vldlr	very low density lipoprotein receptor	-2,03	0,22	1,14	19	27 284 528
6	C	1416855_at	BB550400	Gas1	growth arrest specific 1	-3,16	-1,04	1,61	13	60 184 027
6	C	1449009_at	NM_011579	Tgtp	T-cell specific GTPase	-2,45	-0,71	2,57	11	48 828 752
6	C	1428988_at	AK006128	Abcc3	ATP-binding cassette, sub-family C (CFTR/MRP), member 3	-2,88	0,46	0,40	11	94 159 384
6	C	1427259_at	BB611004	Trim24	tripartite motif protein 24	-2,64	-0,42	0,90	6	37 800 418
6	C	1426378_at	AW741459	Eif4b	eukaryotic translation initiation factor 4B	-2,21	-0,02	1,04	15	101 910 019
6	C	1455713_x_at	BB791424	Phb2	prohibitin 2	-2,12	0,02	0,80	6	124 677 954
6	C	1417420_at	NM_007631	Ccnd1	cyclin D1	-3,74	0,35	2,98	7	144 739 320
6	C	1434601_at	AV315087	Amigo2	adhesion molecule with Ig like domain 2	-2,72	0,39	2,33	15	97 072 159
6	C	1451371_at	AY079153	Mrap	melanocortin 2 receptor accessory protein	-2,44	0,75	1,19	16	90 627 182
6	C	1456204_at	AV069567	2010107H07Rik	RIKEN cDNA 2010107H07 gene	-3,29	0,06	0,74	14	
6	C	1424092_at	BC017137	Epb4.1	erythrocyte protein band 4.1	-2,94	0,28	1,05	4	131 196 252
6	C	1425824_a_at	D01093	Pcsk4	proprotein convertase subtilisin/kexin type 4	-2,35	0,38	2,22	10	79 724 430
6	C	1422076_at	AA571017	Acot4	acyl-CoA thioesterase 4	-2,79	-0,58	1,57	12	84 928 126
6	C	1449304_at	NM_133677	2310061J03Rik	RIKEN cDNA 2310061J03 gene	-2,45	-0,85	0,92	16	55 898 227
6	C	1421087_at	NM_011067	Per3	period homolog 3 (Drosophila)	-2,48	-0,56	1,81	4	149 848 511
6	C	1415696_at	BC005549	Sar1a	SAR1 gene homolog A (S. cerevisiae)	-2,21	0,58	1,59	10	61 075 687
6	C	1432543_a_at	AK002926	Klf13	Kruppel-like factor 13	-2,49	0,43	1,18	7	63 770 316
6	C	1434971_x_at	AV216669			-2,01	-0,43	1,28	1	4 766 458
6	C	1450724_at	NM_053090	Drctnnb1a	down-regulated by Ctnnb1, a	-2,12	-0,62	1,58	5	23 471 094
6	C	1425142_a_at	BC011172	Hnrpd	heterogeneous nuclear ribonucleoprotein D	-2,75	0,24	1,00	5	100 201 308
6	C	1448450_at	NM_016895	Ak2	adenylate kinase 2	-2,43	-0,21	2,42	4	128 495 568
6	C	1418715_at	BC023496	Pank1	pantothenate kinase 1	-2,16	-0,09	1,09	19	34 876 891
6	C	1449556_at	NM_010398	H2T23	histocompatibility 2, T region locus 23	-2,05	-1,01	1,25	17	35 638 031
6	C	1430295_at	BG094302	Gna13	guanine nucleotide binding protein, alpha 13	-2,41	-0,10	1,64	11	109 178 883
6	C	1427258_at	BB611004	Trim24	tripartite motif protein 24	-2,29	-0,29	0,82	6	37 800 418
6	C	1438143_s_at	BB705334	Atxn2	ataxin 2	-2,23	-0,42	1,75	5	121 972 225
6	C	1456808_at	BI076717	4933426M11Rik	RIKEN cDNA 4933426M11 gene	-2,60	0,36	1,17	12	81 709 406
6	C	1448287_at	NM_009087	Rpo13	RNA polymerase 1-3	-2,04	0,74	0,78	5	147 387 889
6	C	1418123_at	BC001990	Unc119	unc-119 homolog (C. elegans)	-2,06	0,24	1,12	11	78 159 716
6	C	1425853_s_at	M22958	Prlr	prolactin receptor	-2,10	-0,24	2,15	15	10 122 379
6	C	1434088_at	BM119467	Zfp496	zinc finger protein 496	-2,07	-1,02	1,13	11	59 301 757
6	C	1451121_a_at	BC017637	Gltscr2	glioma tumor suppressor candidate region gene 2	-2,18	-0,40	1,09	7	15 096 356
6	C	1435803_a_at	BM120823	Eif4e2	eukaryotic translation initiation factor 4E member 2	-2,27	0,24	0,57	1	89 045 109
6	C	1426676_s_at	BB225670	Tomm70a	translocase of outer mitochondrial membrane 70 homolog A (yeast)	-2,65	0,82	1,61	16	57 043 072
6	C	1415839_a_at	NM_008722	Npm1	nucleophosmin 1	-2,45	0,46	1,19	11	33 052 510
6	C	1430404_at	BM239430	Akap13	A kinase (PRKA) anchor protein 13	-2,58	1,42	1,58	7	75 574 377
6	C	1433129_at	AK013628	2900040J22Rik	RIKEN cDNA 2900040J22 gene	-2,53	0,08	0,67	18	
6	C	1441328_at	BB222968	Picalm	phosphatidylinositol binding clathrin assembly protein	-2,01	1,50	-0,10	7	90 005 423
6	C	1420623_x_at	BC006722	Hspa8	heat shock protein 8	-4,06	-0,62	0,93	9	40 552 341
6	C	1423891_at	BC003903	Gstt3	glutathione S-transferase, theta 3	-2,74	1,06	1,30	10	75 217 838

6	C	1416332_at	NM_007705	Cirbp	cold inducible RNA binding protein	-2,15	-0,19	0,99	10	79 570 969
6	C	1423080_at	AK002902	Tomm20	translocase of outer mitochondrial membrane 20 homolog (yeast)	-2,25	0,67	0,61	8	129 820 641
6	C	1419276_at	AF339910	Enpp1	ectonucleotide pyrophosphatase/phosphodiesterase 1	-2,02	-0,12	2,22	10	24 330 826
6	C	1460228_at	AW537576	Usf2	upstream transcription factor 2	-2,45	1,63	0,26	7	30 654 755
6	C	1420033_s_at	AU019644	Ppp2r2d	protein phosphatase 2, regulatory subunit B, delta isoform	-2,12	1,69	1,83	7	138 684 728
6	C	1420622_a_at	BC006722	Hspa8	heat shock protein 8	-4,75	-0,69	1,38	9	40 552 341
6	C	1415716_a_at	AA208652	Rps27	ribosomal protein S27	-2,26	0,17	2,32	3	90 298 597
6	C	1423131_at	BE308547	5730427N09Rik	RIKEN cDNA 5730427N09 gene	-2,06	0,01	0,68	13	98 231 079
6	C	1439268_x_at	BB032885	Eif3s6	eukaryotic translation initiation factor 3, subunit 6	-2,32	0,16	0,76	15	43 080 114
6	C	1448494_at	BB550400	Gas1	growth arrest specific 1	-2,46	-0,12	1,24	13	60 184 027
6	C	1438288_x_at	BB766817	1110059G02Rik	RIKEN cDNA 1110059G02 gene	-2,78	0,46	1,04	15	
6	C	1455282_x_at	AI255644	Alas1	aminolevulinic acid synthase 1	-3,32	1,36	1,09	9	106 092 030
6	C	1427747_a_at	X14607	Lcn2	lipocalin 2	-2,91	-0,19	2,28	2	32 206 645
6	C	1449065_at	NM_012006	Acot1	acyl-CoA thioesterase 1	-2,63	-1,06	1,14	12	84 899 252
6	C	1415773_at	BF118393	Ncl	nucleolin	-2,08	1,09	0,59	1	88 175 889
6	C	1428128_at	BG975608	4921506J03Rik	RIKEN cDNA 4921506J03 gene	-2,10	0,05	1,20	10	112 332 610
6	C	1436404_at	BM119088	Tlcd1	TLC domain containing 1	-2,06	-0,07	0,55	11	77 994 960
6	C	1428926_at	AK003388	1110003O08Rik	RIKEN cDNA 1110003O08 gene	-2,46	0,55	0,97	8	124 435 415
6	C	1435800_a_at	BB779100	Csda	cold shock domain protein A	-2,30	0,40	1,35	6	131 330 550
6	C	1435326_at	BG071867	AW112037	expressed sequence AW112037	-3,09	-0,65	1,44	1	
6	C	1415792_at	NM_019705	Rbck1	RanBP-type and C3HC4-type zinc finger containing 1	-2,23	-0,68	1,15	2	152 007 774
6	C	1453317_a_at	AK014353	Khdrbs3	KH domain containing, RNA binding, signal transduction associated 3	-2,11	-0,90	0,95	15	68 758 088
6	C	1447842_x_at	AV143687	Tcn2	transcobalamin 2	-2,69	-0,20	2,21	11	3 817 195
6	C	1427091_at	BC025488	AI481105	expressed sequence AI481105	-2,54	-1,14	1,86	2	
6	C	1416772_at	NM_009949	Cpt2	carnitine palmitoyltransferase 2	-2,59	0,25	0,71	4	107 401 914
6	C	1434096_at	BB283443	Slc4a4	solute carrier family 4 (anion exchanger), member 4	-2,27	-0,57	0,95	5	89 961 857
6	C	1424126_at	BC022110	Alas1	aminolevulinic acid synthase 1	-3,35	1,37	1,91	9	106 092 030
6	C	1418472_at	BC024934	Aspa	aspartoacylase (aminoacylase) 2	-2,11	-0,68	1,18	11	73 121 182
6	C	1416958_at	NM_011584	Nr1d2	nuclear receptor subfamily 1, group D, member 2	-2,02	0,43	2,00	14	16 997 183
6	C	1454906_at	BB266455	Rarb	retinoic acid receptor, beta	-2,29	-0,39	2,42	14	15 224 108
6	C	1418240_at	NM_010260	Gbp2	guanylate nucleotide binding protein 2	-2,12	-0,66	1,65	3	142 558 048
6	C	1424191_a_at	BC019770	Tmem41a	transmembrane protein 41a	-2,25	-0,15	2,15	16	21 848 309
6	C	1448550_at	NM_008489	Lbp	lipopolysaccharide binding protein	-2,80	-1,12	1,46	2	157 997 985
6	C	1415965_at	NM_009127	Scd1	stearoyl-Coenzyme A desaturase 1	-2,09	-0,07	0,97	19	44 447 765
6	C	1441150_x_at	BB829165			-2,22	0,39	0,75	?	
6	C	1416125_at	U16959	Fkbp5	FK506 binding protein 5	-2,50	1,28	1,37	17	28 126 685
6	C	1437395_at	BE370775	Zcchc11	zinc finger, CCHC domain containing 11	-2,51	-0,12	1,13	4	107 957 357
6	C	1422732_at	NM_026389	Poldip2	polymerase (DNA-directed), delta interacting protein 2	-2,22	-0,14	0,86	11	78 328 490
6	C	1418825_at	NM_008326	Irgm	immunity-related GTPase family, M	-2,35	-0,70	2,86	11	48 708 671
6	C	1416846_a_at	NM_018884	Pdzrn3	PDZ domain containing RING finger 3	-2,50	-0,13	2,41	6	101 115 712
6	C	1455112_at	BB772185	Amid	apoptosis-inducing factor (AIF)-like mitochondrion-associated inducer of death	-2,44	-0,35	0,97	10	61 110 644
6	C	1457896_at	BM120183	Esrrg	estrogen-related receptor gamma	-2,01	-0,92	1,78	1	189 698 646
6	C	1423079_a_at	AK002902	Tomm20	translocase of outer mitochondrial membrane 20 homolog (yeast)	-2,12	1,15	0,67	8	129 820 641
6	C	1450395_at	NM_011396	Slc22a5	solute carrier family 22 (organic cation transporter), member 5	-3,21	0,14	0,76	11	53 707 968
6	C	1426857_a_at	BM200015	Hsdl2	hydroxysteroid dehydrogenase like 2	-2,81	0,02	0,71	4	59 675 743
6	C	1416946_a_at	NM_130864	Acaa1a	acetyl-Coenzyme A acyltransferase 1A	-2,54	-1,01	1,47	9	119 189 991
6	C	1417192_at	NM_138599	Tomm70a	translocase of outer mitochondrial membrane 70 homolog A (yeast)	-2,16	0,57	1,18	16	57 043 072
6	C	1442992_at	BB361398	BC033915	cDNA sequence BC033915	-2,06	-0,26	0,80	9	45 763 872
6	C	1418392_a_at	NM_018734	Gbp4	guanylate nucleotide binding protein 4	-2,95	-0,95	2,35	3	142 497 536
6	C	1423735_a_at	BC021497	Wdr36	WD repeat domain 36	-2,58	0,90	0,85	18	32 980 262
6	C	1448231_at	U16959	Fkbp5	FK506 binding protein 5	-2,12	0,54	0,70	17	28 126 685
6	C	1426856_at	BM200015	Hsdl2	hydroxysteroid dehydrogenase like 2	-3,22	0,24	1,31	4	59 675 743
6	C	1444032_at	AI746421	Keg1	kidney expressed gene 1	-2,03	1,21	2,75	19	12 762 811
6	C	1450643_s_at	BI413218	Acsl1	acyl-CoA synthetase long-chain family member 1	-2,06	-0,83	1,86	8	47 969 858
6	C	1438454_at	BB030030	Pten	phosphatase and tensin homolog	-2,00	0,22	0,57	19	32 823 573
6	C	1436736_x_at	BB369191	D0H4S114	DNA segment, human D4S114	-2,99	-1,38	2,12	18	33 561 831
6	C	1448607_at	AW989410	Pbef1	pre-B-cell colony-enhancing factor 1	-2,26	0,70	1,72	12	33 405 721
6	C	1434036_at	AV024771	Mtss1	metastasis suppressor 1	-3,48	-0,47	1,05	15	58 773 330
6	C	1416234_at	AW476171	Lrrc59	leucine rich repeat containing 59	-2,15	1,43	1,55	11	94 445 913
6	C	1460316_at	BI413218	Acsl1	acyl-CoA synthetase long-chain family member 1	-2,66	-0,50	2,43	8	47 969 858

6	C	1455520_at	AV327200	Ppp2r5c	protein phosphatase 2, regulatory subunit B (B56), gamma isoform	-2,01	-0,52	2,35	12	110 933 695
6	C	1456743_x_at	BB068032	Morf4l2	mortality factor 4 like 2	-2,34	0,18	0,85	X	132 079 316
6	C	1447602_x_at	AU020235	Sulf2	sulfatase 2	-2,01	-1,60	1,39	2	165 764 293
6	C	1420091_s_at	AI452146	Morc3	microrchidia 3	-2,24	0,31	0,83	16	
6	C	1449442_at	NM_011068	Pex11a	peroxisomal biogenesis factor 11a	-2,57	2,28	2,08	7	79 610 776
6	D	1449525_at	NM_008030	Fmo3	flavin containing monooxygenase 3	-6,66	-0,72	6,13	1	164 790 474
6	D	1451593_at	BC018402	H2K1	histocompatibility 2, K1, K region	-4,30	0,39	7,72	17	
6	D	1446085_at	BB022048	Socs2	suppressor of cytokine signaling 2	-6,46	-0,11	4,44	10	94 841 729
6	D	1434465_x_at	AV333363	Vldlr	very low density lipoprotein receptor	-5,32	-0,62	3,95	19	27 284 528
6	D	1421363_at	NM_010003	Cyp2c39	cytochrome P450, family 2, subfamily c, polypeptide 39	-4,31	-0,28	4,05	19	39 564 181
6	D	1452501_at	AF047725	Cyp2c38	cytochrome P450, family 2, subfamily c, polypeptide 38	-4,33	-0,76	3,07	19	39 442 866
6	D	1428547_at	AV273591	Nt5e	5' nucleotidase, ecto	-3,51	-1,01	3,62	9	88 125 532
6	D	1422974_at	NM_011851	Nt5e	5' nucleotidase, ecto	-3,23	-0,53	3,50	9	88 125 532
6	D	1435196_at	BB795585	Ntrk2	neurotrophic tyrosine kinase, receptor, type 2	-3,12	-0,31	4,53	13	58 817 455
6	D	1450226_at	NM_008932	Prlr	prolactin receptor	-4,24	-0,42	3,55	15	10 122 379
6	D	1420342_at	NM_010268	Gdap10	ganglioside-induced differentiation-associated-protein 10	-4,09	1,61	4,27	12	33 407 119
6	D	1449315_at	NM_011857	Odz3	odd Oz/ten-m homolog 3 (Drosophila)	-3,88	0,25	4,16	8	49 726 507
6	D	1436643_x_at	AV051678	Hamp2	hepcidin antimicrobial peptide 2	-3,40	1,39	3,36	7	30 631 156
6	D	1426959_at	BF322712	Bdh1	3-hydroxybutyrate dehydrogenase, type 1	-3,15	-0,09	6,88	16	31 342 040
6	D	1460662_at	NM_011067	Per3	period homolog 3 (Drosophila)	-3,27	0,29	3,22	4	149 848 511
6	D	1417280_at	NM_009198	Slc17a1	solute carrier family 17 (sodium phosphate), member 1	-2,46	0,74	3,10	13	23 877 796
6	D	1460684_at	BC014769	Tm7sf2	transmembrane 7 superfamily member 2	-2,29	-0,11	3,53	19	6 062 821
6	D	1438617_at	BB222737	Serpina7	serine (or cysteine) peptidase inhibitor, clade A (alpha-1 antiproteinase, antitrypsin), member 7	-2,50	0,68	3,69	X	134 426 521
6	D	1422077_at	AA571017	Acot4	acyl-CoA thioesterase 4	-3,98	0,25	3,05	12	84 928 126
6	D	1431095_a_at	AI639807	Herc5	hect domain and RLD 5	-2,51	-0,68	3,08	6	
6	D	1423680_at	BC026831	Fads1	fatty acid desaturase 1	-2,08	1,83	4,14	19	10 250 017
6	D	1428692_at	AW259452	Hddc3	HD domain containing 3	-2,43	-0,40	6,47	7	80 216 649
6	D	1429831_at	BI684288	Pik3ap1	phosphoinositide-3-kinase adaptor protein 1	-2,41	-0,67	6,74	19	41 325 683
6	D	1436194_at	BE985366	C330008K14Rik	RIKEN cDNA C330008K14 gene	-2,20	0,08	8,90	18	
6	D	1420838_at	AK018789	Ntrk2	neurotrophic tyrosine kinase, receptor, type 2	-2,41	0,87	5,45	13	58 817 455
6	D	1455908_a_at	AV102733	Scpep1	serine carboxypeptidase 1	-2,19	0,64	3,93	11	88 740 111
6	D	1451229_at	BC016208	Hdac11	histone deacetylase 11	-2,13	0,04	3,34	6	91 122 318
6	D	1419017_at	NM_016869	Corin	corin	-3,01	-1,29	7,71	5	72 579 160
6	D	1438037_at	AW208668	Ids	iduronate 2-sulfatase	-2,56	-1,48	3,15	X	66 603 736
6	D	1415802_at	NM_009196	Slc16a1	solute carrier family 16 (monocarboxylic acid transporters), member 1	-2,90	1,65	3,30	3	104 767 728
6	D	1443621_at	BG092359	1700010H23Rik	RIKEN cDNA 1700010H23 gene	-2,82	-0,47	8,29	11 ?	
6	D	1455079_at	BQ176904	Dcun1d4	DCN1, defective in cullin neddylation 1, domain containing 4 (S. cerevisiae)	-2,19	0,31	3,09	5	73 770 216
6	D	1455265_a_at	BB100249	Rgs16	regulator of G-protein signaling 16	-3,01	-1,79	3,94	1	155 502 590
6	D	1429947_a_at	AK008179	Zbp1	Z-DNA binding protein 1	-3,24	-0,63	3,71	2	172 849 553
6	D	1423555_a_at	BB329808	Ifi44	interferon-induced protein 44	-2,40	-0,94	4,88	3	151 668 310
7	_	1422804_at	NM_011454	Serpinb6b	serine (or cysteine) peptidase inhibitor, clade B, member 6b	4,11	-2,17	-2,71	13	32 972 977
7	_	1418804_at	NM_032400	Sucnr1	succinate receptor 1	4,67	-2,15	-1,41	3	60 169 797
7	_	1435458_at	AI323550	Pim1	proviral integration site 1	2,71	-3,92	-4,36	17	29 217 823
7	_	1429399_at	BB667823	Rnf125	ring finger protein 125	3,09	-2,01	-3,64	18	21 087 634
7	_	1430274_a_at	AK018331	Stard3nl	STARD3 N-terminal like	2,05	-3,11	-4,17	13	19 365 142
7	_	1430893_at	AK011413	Mup1	major urinary protein 1	4,60	-2,92	-0,25	4	60 312 676
7	_	1438403_s_at	BF537798	Ramp2	receptor (calcitonin) activity modifying protein 2	2,69	-2,48	-0,33	11	101 062 423
7	_	1418188_a_at	AF146523	Ramp2	receptor (calcitonin) activity modifying protein 2	2,56	-2,50	-0,13	11	101 062 423
7	_	1450063_at	BM228488	Fmn2	formin 2	3,97	-2,09	3,69	1	176 338 520
7	_	1430584_s_at	BB213876	Car3	carbonic anhydrase 3	2,63	-2,34	1,12	3	14 840 281
8	A	1441115_at	BB205273	Rnf125	ring finger protein 125	-1,10	-4,55	-2,58	18	21 087 634
8	A	1450702_at	AJ306425	Hfe	hemochromatosis	-0,26	-3,56	-3,16	13	23 711 307
8	A	1437953_at	BM246706	Prei4	preimplantation protein 4	-0,92	-3,57	-3,32	2	132 220 524
8	A	1417930_at	NM_008668	Nab2	Ngfi-A binding protein 2	-0,51	-3,65	-2,63	10	127 063 867
8	A	1442700_at	BG793493	Pde4b	phosphodiesterase 4B, cAMP specific	0,64	-3,42	-2,16	4	101 752 870
8	A	1436202_at	AI853644	Malat1	metastasis associated lung adenocarcinoma transcript 1 (non-coding RNA)	-0,33	-3,59	-1,88	19	
8	A	1458829_at	BB496845			1,56	-3,83	-1,42	13	62 893 557
8	A	1455197_at	BE852181	Rnd1	Rho family GTPase 1	-1,20	-3,84	-2,39	15	98 497 238
8	A	1449317_at	NM_009805	Cflar	CASP8 and FADD-like apoptosis regulator	-1,43	-3,23	-2,34	1	58 657 839
8	A	1434606_at	BF140685	Erbb3	v-erb-b2 erythroblastic leukemia viral oncogene homolog 3 (avian)	0,05	-3,20	-1,24	10	127 972 317

8	A	1456072_at	AU067663	Ppp1r9a	protein phosphatase 1, regulatory (inhibitor) subunit 9A	0,01	-3,28	-3,39	6	4 853 319
8	A	1427590_at	BC011182	Zfp39	zinc finger protein 39	-0,88	-3,04	-3,16	11	58 704 347
8	A	1418745_at	NM_012050	Omd	osteomodulin	1,82	-3,19	-1,98	13	49 594 683
8	A	1442222_at	BB206335	Ifnar1	interferon (alpha and beta) receptor 1	-0,56	-3,24	-1,33	16	91 374 107
8	A	1417086_at	BE688382	Pafah1b1	platelet-activating factor acetylhydrolase, isoform 1b, beta1 subunit	-0,24	-2,79	-1,61	11	74 490 149
8	A	1450878_at	AK008404	Sri	sorcin	-0,91	-3,15	-1,43	5	8 052 083
8	A	1457486_at	BM120811	D9Ucla1	DNA segment, Chr 9, University of California at Los Angeles 1	0,21	-3,26	-3,51	9	42 972 274
8	A	1436054_at	BM220028	9130227C08Rik	RIKEN cDNA 9130227C08Rik gene	-0,10	-2,99	-1,66	14	54 839 039
8	A	1444746_at	BM195033	Ptbp2	polypyrimidine tract binding protein 2	0,05	-3,43	-1,40	3	119 710 998
8	A	1447019_at	BB207504	Cmah	cytidine monophospho-N-acetylneuraminic acid hydroxylase	1,07	-3,13	-1,78	13	24 422 586
8	A	1436842_at	AV229336	B230380D07Rik	RIKEN cDNA B230380D07 gene	-0,20	-2,76	-2,62	9	70 403 759
8	A	1419310_s_at	L43164	Rfxank	regulatory factor X-associated ankyrin-containing protein	-0,22	-2,76	-1,24	8	73 059 794
8	A	1448629_at	NM_138646	Hps4	Hermansky-Pudlak syndrome 4 homolog (human)	1,67	-2,94	-1,05	5	112 585 082
8	A	1443547_at	AI327318			-1,04	-2,74	-2,09	12	81 872 949
8	A	1416442_at	NM_010499	Ier2	immediate early response 2	0,59	-3,10	-1,48	8	87 549 655
8	A	1452604_at	BB667840	Stard13	serologically defined colon cancer antigen 13	1,27	-2,65	-1,85	5	151 306 009
8	A	1434717_at	BM198837	Cul3	cullin 3	-0,22	-2,57	-1,80	1	80 145 841
8	A	1423321_at	BI078799	Myadm	myeloid-associated differentiation marker	-0,63	-2,54	-2,00	7	159 030
8	A	1427885_at	AK010477	Pold4	polymerase (DNA-directed), delta 4	-0,95	-2,60	-1,26	19	4 231 936
8	A	1439244_a_at	BB822587	Tnrc6a	trinucleotide repeat containing 6a	-0,71	-2,45	-1,70	7	122 984 423
8	A	1435580_at	AW553275	C230081A13Rik	RIKEN cDNA C230081A13 gene	0,26	-2,44	-1,90	9	56 025 845
8	A	1428615_at	AK008952	P2ry5	purinergic receptor P2Y, G-protein coupled, 5	0,32	-2,66	-1,78	14	71 972 049
8	A	1455128_x_at	BG342407	Tnrc6a	trinucleotide repeat containing 6a	-0,42	-2,48	-1,99	7	122 983 291
8	A	1422601_at	NM_009256	Serpinb9	serine (or cysteine) peptidase inhibitor, clade B, member 9	1,33	-2,63	-2,18	13	33 012 005
8	A	1435930_at	BQ175997	Zfp291	zinc finger protein 291	-0,48	-2,49	-1,31	9	
8	A	1423939_a_at	BC011117	Yif1a	Yip1 interacting factor homolog A (S. cerevisiae)	-0,85	-2,39	-1,45	19	5 088 552
8	A	1453023_at	AK003441	Ankhd1	ankyrin repeat and KH domain containing 1	-1,06	-2,57	-3,32	18	
8	A	1436767_at	BB475271	Luc7l2	LUC7-like 2 (S. cerevisiae)	0,26	-2,61	-1,78	6	38 481 126
8	A	1433618_at	BB397899	C330006A16Rik	RIKEN cDNA C330006A16 gene	-0,08	-2,32	-1,49	2	
8	A	1415899_at	NM_008416	Junb	Jun-B oncogene	-1,04	-2,55	-1,15	8	87 867 037
8	A	1443670_at	AI451392	2010001J22Rik	RIKEN cDNA 2010001J22 gene	0,10	-2,59	-2,80	15	89 205 215
8	A	1429639_at	AK009137	Prei4	preimplantation protein 4	-1,07	-2,44	-2,29	2	132 220 524
8	A	1436994_a_at	BB533903	Hist1h1c	histone 1, H1c	-1,16	-2,51	-1,35	13	23 746 271
8	A	1449403_at	NM_008804	Pde9a	phosphodiesterase 9A	0,09	-2,39	-1,77	17	31 114 960
8	A	1449303_at	NM_030261	Sesn3	sestrin 3	0,90	-2,46	-2,33	9	14 026 697
8	A	1442593_at	AW553880	LOC668833		-0,75	-2,31	-1,62	12	41 629 177
8	A	1433468_at	BB770958	6430527G18Rik	RIKEN cDNA 6430527G18 gene	0,36	-2,56	-2,10	12	87 769 805
8	A	1450028_a_at	NM_133737	Lancl2	LanC (bacterial lantibiotic synthetase component C)-like 2	0,47	-2,23	-2,08	6	57 632 213
8	A	1441946_at	AV239969	Itih5	inter-alpha (globulin) inhibitor H5	1,28	-2,31	-3,49	2	10 071 468
8	A	1455508_at	BF318246	A530082C11Rik	RIKEN cDNA A530082C11 gene	-0,59	-2,56	-3,29	4	154 445 215
8	A	1437235_x_at	BB218844	Morf4l1	mortality factor 4 like 1	-1,16	-2,45	-1,70	9	89 889 563
8	A	1444691_at	BB667397	Prkcn	protein kinase C, nu	-0,78	-2,30	-1,92	17	78 855 612
8	A	1422811_at	NM_011977	Slc27a1	solute carrier family 27 (fatty acid transporter), member 1	-0,69	-2,38	-2,65	8	74 497 915
8	A	1434890_at	BG072813	Opa1	optic atrophy 1 homolog (human)	-0,66	-2,49	-1,45	16	29 499 088
8	A	1434116_at	BI693188	Cbx2	chromobox homolog 2 (Drosophila Pc class)	-1,25	-2,36	-1,45	11	118 839 118
8	A	1439038_at	BF455240	9130227C08Rik	RIKEN cDNA 9130227C08Rik gene	0,18	-2,28	-2,40	14	54 839 039
8	A	1423155_at	AK008404	Sri	sorcin	0,06	-2,37	-1,47	5	8 052 083
8	A	1428228_at	AK013402	Pgm3	phosphoglucomutase 3	-0,98	-2,23	-3,06	9	86 349 193
8	A	1435222_at	BM220880	Foxp1	forkhead box P1	0,18	-2,41	-2,37	6	98 895 553
8	A	1443671_x_at	AI451392	2010001J22Rik	RIKEN cDNA 2010001J22 gene	0,22	-2,02	-1,99	15	89 205 215
8	A	1424402_at	AW494299	Rufy3	RUN and FYVE domain containing 3	0,94	-2,60	-1,47	5	89 658 134
8	A	1441331_at	BB153043	A230061C15Rik	RIKEN cDNA A230061C15 gene	-0,86	-2,17	-3,12	10	
8	A	1424904_at	BC019143	1300010F03Rik	RIKEN cDNA 1300010F03 gene	-1,14	-2,68	-2,35	14	
8	A	1419329_at	NM_011366	Sorbs3	sorbin and SH3 domain containing 3	-1,38	-2,38	-1,88	14	68 915 548
8	A	1433857_at	AV088463	Fath	fat tumor suppressor homolog (Drosophila)	1,94	-2,27	-2,71	8	46 535 639
8	A	1440226_at	BB088782	Zfp760	zinc finger protein 760	0,42	-2,35	-1,44	17	21 412 440
8	A	1421918_at	AF022957	Anp32a	acidic (leucine-rich) nuclear phosphoprotein 32 family, member A	-1,33	-2,41	-1,23	9	62 139 433
8	A	1448825_at	NM_133667	Pdk2	pyruvate dehydrogenase kinase, isoenzyme 2	-0,24	-2,26	-1,20	11	94 842 348
8	A	1447621_s_at	AV300716	2610307O08Rik	RIKEN cDNA 2610307O08 gene	0,19	-2,09	-1,53	18	
8	A	1448227_at	NM_010346	Grb7	growth factor receptor bound protein 7	-0,92	-2,03	-1,95	11	98 262 980
8	A	1436902_x_at	BB096368	Tmsb10	thymosin, beta 10	0,49	-2,11	-1,42	6	72 886 861

8	A	1430857_s_at	AK007848	Pex11c	peroxisomal biogenesis factor 11c	1,00	-2,26	-1,69	8	3 458 816
8	A	1417952_at	NM_010008	Cyp2j6	cytochrome P450, family 2, subfamily j, polypeptide 6	1,34	-2,01	-1,34	4	96 009 259
8	A	1439554_at	AV323498	Scmh1	sex comb on midleg homolog 1	0,92	-2,04	-1,49	4	119 903 043
8	A	1444430_at	BB481523	Armc8	armadillo repeat containing 8	-0,05	-2,06	-1,50	9	99 289 446
8	A	1426818_at	BC025091	Arrdc4	arrestin domain containing 4	-1,49	-2,38	-1,88	7	68 610 507
8	A	1431220_at	AI119761	Tbl1xr1	transducin (beta)-like 1X-linked receptor 1	-1,31	-2,10	-1,53	3	22 267 856
8	A	1420515_a_at	NM_021319	Pglyrp2	peptidoglycan recognition protein 2	-0,33	-2,23	-1,21	17, Un	32 143 639
8	A	1448426_at	BI217574	Sardh	sarcosine dehydrogenase	-0,40	-2,11	-3,08	2	27 011 391
8	A	1417271_a_at	NM_007932	Eng	endoglin	0,32	-2,28	-1,51	2	32 468 603
8	A	1459695_at	AU040352			-0,27	-2,37	-1,35	16	42 952 401
8	A	1455899_x_at	BB241535	Socs3	suppressor of cytokine signaling 3	-1,25	-2,92	-2,29	11	117 782 178
8	A	1455663_at	BB055163	Olfml1	olfactomedin-like 1	1,22	-2,20	-2,07	7	107 358 622
8	A	1417749_a_at	NM_009386	Tjp1	tight junction protein 1	0,28	-2,04	-1,56	7	65 175 117
8	A	1451970_at	BC016105	E330036I19Rik	RIKEN cDNA E330036I19 gene	1,13	-2,03	-2,03	5	143 728 562
8	A	1434366_x_at	AW227993	C1qb	complement component 1, q subcomponent, beta polypeptide	-1,22	-2,24	-2,07	4	136 152 223
8	A	1439531_at	BB754259	E130311K13Rik	RIKEN cDNA E130311K13 gene	1,16	-2,03	-1,35	3	64 002 624
8	A	1437484_at	AI267092	Zbtb5	zinc finger and BTB domain containing 5	1,74	-2,02	-1,91	4	45 012 341
8	A	1454609_x_at	BB770958	6430527G18Rik	RIKEN cDNA 6430527G18 gene	1,70	-2,12	-3,03	12	87 769 805
8	A	1419583_at	NM_007625	Cbx4	chromobox homolog 4 (Drosophila Pc class)	-1,36	-2,08	-2,41	11	118 896 246
8	A	1419248_at	AF215668	Rgs2	regulator of G-protein signaling 2	-0,13	-2,00	-1,53	1	145 762 214
8	A	1418443_at	BC025628	Xpo1	exportin 1, CRM1 homolog (yeast)	-0,81	-2,17	-1,62	11	23 156 040
8	A	1418073_at	NM_019736	Acot9	acyl-CoA thioesterase 9	-0,37	-2,04	-2,40	X	150 603 225
8	A	1451339_at	BC027197	Suox	sulfite oxidase	-0,52	-2,11	-1,56	10	128 072 843
8	A	1448272_at	NM_007570	Btg2	B-cell translocation gene 2, anti-proliferative	-1,22	-2,09	-2,34	1	135 891 275
8	A	1456212_x_at	BB831725	Socs3	suppressor of cytokine signaling 3	-1,29	-2,03	-2,00	11	117 782 178
8	A	1442849_at	AV345706	Lrp1	low density lipoprotein receptor-related protein 1	-0,93	-2,03	-1,18	10	126 941 142
8	B	1435872_at	BE631223	Pim1	proviral integration site 1	0,89	-5,67	-8,45	17	29 217 823
8	B	1418250_at	NM_025404	Arfl4	ADP-ribosylation factor 4-like	-1,42	-4,21	-5,72	11	101 481 650
8	B	1438391_x_at	AV078914	Hadh2	hydroxyacyl-Coenzyme A dehydrogenase type II	-1,60	-4,05	-4,61	X	147 342 611
8	B	1448034_at	AA215276	AI842396	expressed sequence AI842396	-0,63	-3,23	-4,28	11	70 504 555
8	B	1448286_at	NM_016763	Hadh2	hydroxyacyl-Coenzyme A dehydrogenase type II	-1,21	-3,71	-4,04	X	147 342 611
8	B	1443027_at	BB667435	4930523C07Rik	RIKEN cDNA 4930523C07 gene	0,13	-3,00	-6,79	1	161 881 063
8	B	1429159_at	AK018605	4631408O11Rik	RIKEN cDNA 4631408O11 gene	1,43	-2,75	-4,19	2	
8	B	1438081_at	BB794635	Mcc	mutated in colorectal cancers	-0,63	-3,13	-4,07	18	44 550 391
8	B	1427213_at	X98848	Pfkfb1	6-phosphofructo-2-kinase/fructose-2,6-biphosphatase 1	-0,74	-2,75	-3,97	X	145 930 636
8	B	1457141_at	BB229969	Aqp11	aquaporin 11	-1,08	-2,35	-4,66	7	97 601 570
8	B	1452349_x_at	AI481797	Ifi205	interferon activated gene 205	-1,08	-2,18	-5,62	1	175 848 673
8	B	1454973_at	BF456242	Atf7ip	activating transcription factor 7 interacting protein	0,78	-2,12	-4,32	6	136 483 046
8	B	1441423_at	BB344763	Eif4g3	eukaryotic translation initiation factor 4 gamma, 3	-0,31	-2,27	-6,03	4	137 265 545
8	B	1451602_at	BC025911	Snx6	sorting nexin 6	-0,36	-2,29	-7,00	12	55 667 982
8	B	1439631_at	BG060248	Zcchc11	zinc finger, CCHC domain containing 11	-0,95	-2,19	-4,13	4	107 957 357
8	B	1424673_at	AF350410	Clec2h	C-type lectin domain family 2, member h	-0,19	-2,34	-4,28	6	128 628 004
8	B	1429254_at	BF472491	Aqp11	aquaporin 11	-1,07	-2,14	-4,72	7	97 601 570
8	B	1433758_at	BB025231	Nisch	nischarin	0,34	-2,07	-6,14	14	29 999 938
8	B	1449836_x_at	NM_007546	Bik	Bcl2-interacting killer	-0,05	-2,07	-5,76	15	83 354 637
8	B	1452231_x_at	M74124	Ifi205	interferon activated gene 205	-1,13	-2,07	-7,78	1	175 848 673
8	B	1424857_a_at	AF220142	Trim34	tripartite motif protein 34	-0,15	-2,08	-5,49	7	104 118 384
8	C	1429152_at	AI509716	Zkscan1	zinc finger with KRAB and SCAN domains 1	0,30	-3,87	-0,44	5	138 314 871
8	C	1436199_at	AU042532			0,10	-4,27	-0,34	4	46 526 113
8	C	1450947_at	AK012175	2610528J11Rik	RIKEN cDNA 2610528J11 gene	0,07	-3,93	-0,46	4	118 025 213
8	C	1424522_at	BC019693	Heatr1	HEAT repeat containing 1	-0,97	-3,42	-0,73	13	12 449 707
8	C	1426332_a_at	AF087821	Cldn3	claudin 3	-0,57	-3,48	0,06	5	135 270 840
8	C	1426276_at	AY075132	Ifih1	interferon induced with helicase C domain 1	-1,92	-3,49	0,17	2	62 398 288
8	C	1434651_a_at	AW611462	Cldn3	claudin 3	-0,29	-2,99	0,53	5	135 270 840
8	C	1434449_at	BB193413	Aqp4	aquaporin 4	1,97	-2,78	-0,27	18	15 535 849
8	C	1451701_x_at	BC012650	Cldn3	claudin 3	-0,18	-2,98	0,32	5	135 270 840
8	C	1447643_x_at	BB040443	Snai2	snail homolog 2 (Drosophila)	-0,68	-3,51	-0,25	16	14 619 436
8	C	1425746_at	BC021771	D730039F16Rik	RIKEN cDNA D730039F16 gene	0,75	-2,65	0,46	2	34 696 444
8	C	1458399_at	AI413999	Lrrc3	leucine rich repeat containing 3	-0,61	-2,69	-0,06	10	77 302 975
8	C	1460569_x_at	AW611462	Cldn3	claudin 3	-1,25	-2,63	1,01	5	135 270 840
8	C	1436187_at	BB513634	1110054M08Rik	RIKEN cDNA 1110054M08 gene	-1,48	-3,05	-1,08	16	

8	C	1442056_at	BE200446	Zfp608	zinc finger protein 608	-1,15	-2,59	0,10	18	
8	C	1445729_at	BB742199	Sirt3	sirtuin 3 (silent mating type information regulation 2, homolog) 3 (S. cerevisiae)	-1,42	-2,43	-0,82	7	140 714 992
8	C	1457177_at	BB050663	Rora	RAR-related orphan receptor alpha	-0,33	-2,43	0,22	9	68 452 965
8	C	1423050_s_at	BF228203	Hnrpu	heterogeneous nuclear ribonucleoprotein U	-1,24	-2,59	-1,20	1	180 165 993
8	C	1423348_at	AV345166	Fzd8	frizzled homolog 8 (Drosophila)	-0,90	-2,32	-0,26	18	9 212 917
8	C	1452207_at	Y15163	Cited2	Cbp/p300-interacting transactivator, with Glu/Asp-rich carboxy-terminal domain, 2	0,65	-2,57	-0,63	10	17 412 661
8	C	1456303_at	BI465935	Phf14	PHD finger protein 14	-0,41	-2,39	-0,84	6	11 875 880
8	C	1442017_at	AI662441	Nfs1	nitrogen fixation gene 1 (S. cerevisiae)	-0,90	-2,56	-0,30	2	155 815 090
8	C	1441988_at	AI482429	Spag5	sperm associated antigen 5	-1,06	-2,46	-0,62	11	78 117 833
8	C	1452070_at	AK010701	Dedd2	death effector domain-containing DNA binding protein 2	-1,41	-2,29	-0,88	7	24 911 597
8	C	1457110_at	BB440150	Pank1	pantothenate kinase 1	-0,41	-2,59	-0,42	19	34 876 891
8	C	1415850_at	NM_009025	Rasa3	RAS p21 protein activator 3	-0,31	-2,34	-0,02	8	13 567 194
8	C	1451601_a_at	BC025823	BC011467	cDNA sequence BC011467	0,30	-2,43	-0,85	11	72 267 837
8	C	1422286_a_at	NM_009372	Tgif	TG interacting factor	0,51	-2,11	-1,04	17	70 749 075
8	C	1418913_at	NM_022884	Bhmt2	betaine-homocysteine methyltransferase 2	-0,60	-2,21	-0,14	13	94 757 012
8	C	1418438_at	NM_007980	Fabp2	fatty acid binding protein 2, intestinal	-1,39	-2,38	0,93	3	122 887 413
8	C	1433429_at	BB309245	Pigs	phosphatidylinositol glycan anchor biosynthesis, class S	-0,46	-2,29	-0,69	11	78 144 616
8	C	1450869_at	AI649186	Fgf1	fibroblast growth factor 1	-1,00	-2,49	-0,73	18	38 965 153
8	C	1458208_s_at	BB770903	8430422M14Rik	RIKEN cDNA 8430422M14 gene	-0,04	-2,17	-0,54	3	36 150 860
8	C	1440417_at	BG918834	D19Ertd409e	DNA segment, Chr 19, ERATO Doi 409, expressed	1,29	-2,29	-0,96	19	
8	C	1422662_at	AI987967	Lgals8	lectin, galactose binding, soluble 8	-0,08	-2,37	-0,19	13	12 493 756
8	C	1418189_s_at	AF146523	Ramp2	receptor (calcitonin) activity modifying protein 2	1,49	-2,35	0,45	11	101 062 423
8	C	1444184_at	AI042789	Alkbh3	alkB, alkylation repair homolog 3 (E. coli)	0,71	-2,35	-0,96	2	93 781 473
8	C	1453588_at	BB213876	Car3	carbonic anhydrase 3	2,19	-3,19	0,57	3	14 840 281
8	C	1448873_at	NM_008756	Ocln	occludin	-0,40	-2,09	0,36	13	101 597 623
8	C	1436892_at	BB133520	Spred2	sprouty-related, EVH1 domain containing 2	-0,09	-2,11	-0,90	11	19 824 444
8	C	1447310_at	AI426506	C630028N24Rik	RIKEN cDNA C630028N24 gene	-0,77	-2,35	-0,17	9	54 715 419
8	C	1445640_at	AW610715	Hlcs	holocarboxylase synthetase (biotin- [propriony-Coenzyme A-carboxylase (ATP-hydrolysing)] ligase)	-0,43	-2,17	0,61	16	94 239 206
8	C	1445591_at	AI314018	Sardh	sarcosine dehydrogenase	-0,17	-2,08	-1,25	2	27 011 391
8	C	1444466_at	BB433324	Ncald	neurocalcin delta	-0,71	-2,09	-0,78	15	37 310 763
8	C	1433505_a_at	BB315861	Lrrc8d	leucine rich repeat containing 8D	-0,04	-2,23	0,10	5	105 941 135
8	C	1423076_at	BB703513	Snx9	sorting nexin 9	-0,31	-2,05	-0,34	17	5 797 845
8	C	1439175_at	BB131938	Ptprd	protein tyrosine phosphatase, receptor type, D	-0,56	-2,05	-1,12	4	75 412 469
8	C	1444092_at	BM228262	9430025M13Rik	RIKEN cDNA 9430025M13 gene	-0,70	-2,18	-0,41	7	39 248 490
8	C	1455725_a_at	BI903743	H3f3b	H3 histone, family 3B	-1,12	-2,18	-0,85	11	115 838 382
8	C	1424418_at	BC010801	BC010801	cDNA sequence BC010801	-1,09	-2,06	-0,79	9	119 959 096
8	C	1452256_at	AK003325	1110002N22Rik	RIKEN cDNA 1110002N22 gene	0,29	-2,06	-0,85	11	79 952 876
8	C	1418442_at	BC025628	Xpo1	exportin 1, CRM1 homolog (yeast)	-0,32	-2,10	-0,89	11	23 156 040
8	C	1459202_at	BB366565	Cecr2	cat eye syndrome chromosome region, candidate 2 homolog (human)	-1,40	-2,21	0,33	6	
8	C	1425673_at	BC005613	Lpp	LIM domain containing preferred translocation partner in lipoma	-1,39	-2,02	-0,65	16	24 308 838
8	C	1422661_at	AI987967	Lgals8	lectin, galactose binding, soluble 8	-0,42	-2,10	-0,15	13	12 493 756
8	C	1428853_at	BG071079	Ptch1	patched homolog 1	-0,81	-2,03	-0,75	13	63 520 754
8	C	1457161_at	BB111383	9530029O12Rik	RIKEN cDNA 9530029O12 gene	-0,78	-2,01	0,15	11	45 706 340
8	C	1416639_at	NM_019741	Slc2a5	solute carrier family 2 (facilitated glucose transporter), member 5	0,68	-2,29	0,55	4	148 963 143
8	C	1448747_at	AF441120	Fbxo32	F-box only protein 32	-0,73	-2,02	-1,22	15	58 010 161
8	C	1429550_at	AK008590	Entpd8	ectonucleoside triphosphate diphosphohydrolase 8	0,00	-2,06	-0,58	2	
8	C	1459008_at	BM942252			0,67	-2,10	-1,22	?	
8	C	1456253_s_at	BB448454	Klhl17	kelch-like 17 (Drosophila)	-0,51	-2,05	0,97	4	
8	C	1458125_at	BM199710	C80913	expressed sequence C80913	-0,86	-2,02	0,15	7	37 668 761
8	C	1460314_s_at	NM_019469	Hist2h3c1	histone 2, H3c1	-0,44	-2,14	-1,03	3	96 354 098
8	C	1420543_at	BC005604	ORF28	open reading frame 28	-0,67	-2,01	-0,28	16	91 504 589
8	C	1415973_at	AW546141	Marcks	myristoylated alanine rich protein kinase C substrate	0,25	-2,02	-0,46	10	36 824 236
8	C	1437873_at	BB044772	6030490I01Rik	RIKEN cDNA 6030490I01 gene	-0,52	-2,12	-1,08	17	32 554 572
8	C	1450857_a_at	BF227507	Col1a2	procollagen, type I, alpha 2	-1,49	-2,14	0,01	6	4 455 696
8	C	1446342_at	BB137728	2310001H17Rik	RIKEN cDNA 2310001H17 gene	1,27	-2,03	-0,35	6	
8	C	1419483_at	NM_009779	C3ar1	complement component 3a receptor 1	-0,46	-2,15	-0,81	6	122 813 592
8	C	1444790_at	AV048499	1810005K13Rik	RIKEN cDNA 1810005K13 gene	0,02	-2,07	-0,43	18	
8	C	1451291_at	BC026942	Obfc2b	oligonucleotide/oligosaccharide-binding fold containing 2B	0,09	-2,12	-0,29	10	127 804 344

8	C	1454137_s_at	AK009636	Hfe2	hemochromatosis type 2 (juvenile) (human homolog)	1,27	-2,01	-0,28	3	96 610 589
8	C	1423704_at	BC019373	Lypla3	lysophospholipase 3	0,27	-2,13	-0,65	8	109 039 527
8	C	1451321_a_at	BC003333	0610033I05Rik	RIKEN cDNA 0610033I05 gene	-1,08	-2,12	-0,62	2	51 746 465
8	C	1439722_at	BB165972	BC020002	cDNA sequence BC020002	-1,29	-2,04	-0,74	6	8 159 228
8	C	1450014_at	NM_016674	Cldn1	claudin 1	1,79	-2,03	-0,02	16	26 271 999
8	C	1437347_at	BF100813	Ednrb	endothelin receptor type B	-1,23	-2,27	-1,16	14	102 700 304
8	C	1432355_at	AK008780	2210039B01Rik	RIKEN cDNA 2210039B01 gene	0,12	-2,00	0,97	12	74 467 316
8	C	1423889_at	BC011344	MGC5739		-0,83	-2,12	-0,01	9	
8	C	1431916_at	AK018558	9030618K22Rik	RIKEN cDNA 9030618K22 gene	0,35	-2,32	-0,83	3	98 889 683
8	C	1435602_at	BE200310	Sephs2	selenophosphate synthetase 2	-0,28	-2,05	-0,86	7	127 063 030
8	C	1437211_x_at	BB254141	Elovl5	ELOVL family member 5, elongation of long chain fatty acids (yeast)	-1,49	-2,12	0,01	9	77 703 051
8	C	1444518_at	BM240237	Acox1	acyl-Coenzyme A oxidase 1, palmitoyl	-0,64	-2,04	-0,76	11	115 987 978
8	C	1450624_at	NM_016668	Bhmt	betaine-homocysteine methyltransferase	0,31	-2,25	-0,69	13	94 717 645
8	C	1435454_a_at	BE853170	BC006779	cDNA sequence BC006779	-1,30	-2,02	0,23	2	
8	D	1450696_at	NM_013585	Psmb9	proteosome (prosome, macropain) subunit, beta type 9 (large multifunctional peptidase 2)	-0,90	-3,94	3,24	17	33 792 386
8	D	1457027_at	BB667395	Dhtkd1	dehydrogenase E1 and transketolase domain containing 1	-1,17	-3,65	5,15	2	
8	D	1433507_a_at	BE553881	Hmgn2	high mobility group nucleosomal binding domain 2	-1,26	-3,65	1,76	4	133 236 816
8	D	1436562_at	BG063981	Ddx58	DEAD (Asp-Glu-Ala-Asp) box polypeptide 58	-1,98	-2,88	2,03	4	40 394 460
8	D	1434380_at	BM241271	Gbp7	guanylate binding protein 7	-2,92	-3,30	4,08	3	142 467 727
8	D	1443858_at	BI653857	Trim34	tripartite motif protein 34	-0,85	-2,20	2,68	7	104 118 384
8	D	1453547_at	AK007796	1810046K07Rik	RIKEN cDNA 1810046K07 gene	-0,51	-2,27	1,35	9	
8	D	1441242_at	BB229546	Dpp4	dipeptidylpeptidase 4	0,28	-2,19	1,33	2	62 132 578
8	D	1442535_at	BB533591	Zfp609	zinc finger protein 609	-0,65	-2,29	1,38	9	65 493 469
8	D	1429535_at	AK004793	Armc8	armadillo repeat containing 8	-0,91	-2,02	2,46	9	99 289 446
8	D	1423216_a_at	AV109006	2510049I19Rik	RIKEN cDNA 2510049I19 gene	-0,60	-2,25	3,10	8	75 148 718
8	D	1427797_s_at	BF580235	Ctse	cathepsin E	1,80	-2,06	2,92	1	133 465 859
8	D	1426037_a_at	U94828	Rgs16	regulator of G-protein signaling 16	-2,60	-2,39	2,73	1	155 502 590
8	D	1460674_at	BC022922	Paqr7	progestin and adipoQ receptor family member VII	0,18	-2,02	4,44	4	133 769 053
8	D	1419100_at	NM_009252	Serpina3n	serine (or cysteine) peptidase inhibitor, clade A, member 3N	-1,30	-2,18	3,98	12	104 807 808

Genes were selected as described in Materials and Methods. Values are the mean of 3 S-scores comparing D2KO versus D2WT expression, B6KO versus B6WT expression, and D2WT versus B6WT expression.
