# Supplementary material for: Gene expression profiling of Hfe-/- liver and duodenum in mouse strains with differing susceptibilities to iron loading: identification of transcriptional regulatory targets of Hfe and potential hemochromatosis modifiers
Source: Genome Biol. 2007 Oct 18;8(10):R221. doi: 10.1186/gb-2007-8-10-r221 (PMC2246295; doi:10.1186/gb-2007-8-10-r221)
Supplement: Additional File 2 — Presented is a table listing genes significantly regulated by Hfe disruption in the duodenum of D2 or B6 mice. [file gb-2007-8-10-r221-S2.pdf]

Additional data file 2. Genes regulated by Hfe disruption in D2 and/or B6 duodenum.

Cluster	ProbesetID	Genbank	Gene symbol	Description	D2KOvsD2WT	B6KOvsB6WT	D2WTvsB6WT	Chromosome	Position (kb)
1 A	1437286_x_at	BB277742	1110020G09Rik	RIKEN cDNA 1110020G09 gene	4,04	-1,10	-0,56	15	8 998 562
1 A	1439678_at	BM239869	Gls	glutaminase	4,80	-0,40	-0,53	1	
1 A	1437285_at	BB277742	1110020G09Rik	RIKEN cDNA 1110020G09 gene	3,63	-0,65	-0,35	15	8 998 562
1 A	1428236_at	AK005001	Acbd5	acyl-Coenzyme A binding domain containing 5	3,20	-0,98	-0,61	2	22 891 457
1 A	1435494_s_at	AV297961	Dsp	desmoplakin	3,41	0,16	-0,43	13	
1 A	1440005_at	BB667396	C730009D12		3,02	-1,48	-1,15	18	
1 A	1455232_at	BB745660	Cml2	camello-like 2	3,42	-0,27	-0,32	6	85 831 056
1 A	1452942_at	BF383782	Tmem65	transmembrane protein 65	3,53	-0,34	-1,09	15	58 612 352
1 A	1434657_at	BE983473	AI314027	expressed sequence AI314027	2,80	-0,60	-0,71	1	
1 A	1445870_at	BB367621	Cdc42ep2	CDC42 effector protein (Rho GTPase binding) 2	2,97	-0,59	0,21	19	5 917 560
1 A	1429108_at	BB745314	Msl2l1	male-specific lethal 2-like 1 (Drosophila)	3,19	-0,40	0,10	9	
1 A	1437287_at	BB277742	1110020G09Rik	RIKEN cDNA 1110020G09 gene	3,30	-0,47	-0,52	15	8 998 562
1 A	1444980_at	BE996518	C730009D12		2,81	-1,48	-0,89	18	
1 A	1422450_at	NM_007615	Ctnnd1	catenin (cadherin associated protein), delta 1	2,79	0,02	-0,94	2	84 401 621
1 A	1445824_at	AV276001	Zfp458	zinc finger protein 458	2,57	-0,34	0,20	13	67 754 015
1 A	1434329_s_at	BG074607	Adipor2	adiponectin receptor 2	2,46	-1,18	0,51	6	119 318 770
1 A	1452679_at	AA986082	Tubb2b	tubulin, beta 2b	2,66	-1,34	0,33	13	34 134 472
1 A	1449459_s_at	AF403041	Asb13	ankyrin repeat and SOCS box-containing protein 13	2,65	-0,35	-0,51	13	3 633 323
1 A	1425314_at	AF435926	Gpr98	G protein-coupled receptor 98	2,41	-0,65	0,85	13	81 558 503
1 A	1439568_at	AV373997	Greb1	gene regulated by estrogen in breast cancer protein	2,64	-0,69	-1,06	12	16 697 104
1 A	1437333_x_at	BB833010	Aldh18a1	aldehyde dehydrogenase 18 family, member A1	2,42	-0,98	-0,28	19	40 603 567
1 A	1415941_s_at	NM_133349	Zfand2a	zinc finger, AN1-type domain 2A	2,55	-0,56	-1,16	5	139 724 858
1 A	1439999_at	BB432092			2,68	-0,19	-1,45	10	98 456 397
1 A	1416980_at	NM_027853	Mettl7b	methyltransferase like 7B	2,46	-0,62	-1,14	10	128 361 225
1 A	1434061_at	BB431808	Rp2h	retinitis pigmentosa 2 homolog (human)	2,44	-0,58	-0,84	X	19 521 566
1 A	1418429_at	BI328541	Kif5b	kinesin family member 5B	2,56	-0,81	-1,72	18	6 202 229
1 A	1437325_x_at	BB251523	Aldh18a1	aldehyde dehydrogenase 18 family, member A1	2,49	-1,34	-0,29	19	40 603 567
1 A	1415836_at	NM_019698	Aldh18a1	aldehyde dehydrogenase 18 family, member A1	2,58	-0,63	0,25	19	40 603 567
1 A	1448470_at	NM_019395	Fbp1	fructose bisphosphatase 1	2,36	-1,67	-1,54	13	62 874 376
1 A	1441930_x_at	BB089991	Vat1	vesicle amine transport protein 1 homolog (T californica)	2,57	-0,84	-1,06	11	101 274 837
1 A	1460370_at	AF362952	Top1mt	DNA topoisomerase 1, mitochondrial	2,40	-0,65	-0,09	15	75 484 288
1 A	1445128_at	BQ176646			2,54	-1,95	-0,01	9	96 877 535
1 A	1460444_at	AK004614	Arrb1	arrestin, beta 1	2,78	-0,87	-0,17	7	99 409 568
1 A	1457731_at	BB247595	Psip1	PC4 and SFRS1 interacting protein 1	2,31	-0,14	-0,98	4	82 926 910
1 A	1418505_at	NM_027722	Nudt4	nudix (nucleoside diphosphate linked moiety X)-type motif 4	2,71	0,38	-1,23	10	94 976 976
1 A	1429347_at	AK016670	Bcl2l14	Bcl2-like 14 (apoptosis facilitator)	2,19	-0,01	-1,13	6	134 362 021
1 A	1433831_at	BM213879	4833418A01Rik	RIKEN cDNA 4833418A01 gene	2,38	-0,45	-0,11	2	70 856 690
1 A	1459860_x_at	BB466780	Trim2	tripartite motif protein 2	2,36	-1,52	-1,73	3	84 246 371
1 A	1448415_a_at	NM_009153	Sema3b	sema domain, immunoglobulin domain (Ig), short basic domain, secreted, (semaphorin) 3B	2,71	0,19	-1,00	9	107 456 215
1 A	1420821_at	NM_030750	Sgpp1	sphingosine-1-phosphate phosphatase 1	2,41	-2,08	-1,78	12	76 633 106
1 A	1417319_at	NM_021495	Pvrl3	poliovirus receptor-related 3	2,35	-0,85	-1,32	16	46 367 420
1 A	1451271_a_at	BG070487	Acat1	acetyl-Coenzyme A acetyltransferase 1	2,52	-0,74	-0,70	9	53 342 916
1 A	1434193_at	AV122561	Zmym6	zinc finger, MYM-type 6	2,09	-0,30	-1,18	4	126 579 686
1 A	1440051_at	BB467915	Ppp3ca	protein phosphatase 3, catalytic subunit, alpha isoform	2,27	-0,70	-0,02	3	136 608 156
1 A	1436737_a_at	BB737680	Sorbs1	sorbin and SH3 domain containing 1	2,29	-0,83	-0,24	19	40 345 351
1 A	1434853_x_at	AA717142	Mkrn1	makorin, ring finger protein, 1	2,22	-0,58	-0,95	6	39 327 426
1 A	1436994_a_at	BB533903	Hist1h1c	histone 1, H1c	2,35	-1,90	-1,05	13	23 746 271
1 A	1426675_at	BB225670	Tomm70a	translocase of outer mitochondrial membrane 70 homolog A (yeast)	2,39	-1,21	-0,12	16	57 043 072
1 A	1419401_at	AF403041	Asb13	ankyrin repeat and SOCS box-containing protein 13	2,21	-0,29	-0,42	13	3 633 323
1 A	1438442_at	AI450236	AI450236	expressed sequence AI450236	2,38	-0,68	0,24	3	103 136 225
1 A	1432946_at	AK017228	5230400M06Rik	RIKEN cDNA 5230400M06 gene	2,05	0,23	-1,07	14	
1 A	1436915_x_at	AU024771	Laptm4b	lysosomal-associated protein transmembrane 4B	2,20	-1,03	-1,27	15	34 182 616
1 A	1455285_at	BB771765	Slc31a1	solute carrier family 31, member 1	2,18	0,31	-1,25	4	61 847 109
1 A	1439406_x_at	BB530332	Fars2	phenylalanine-tRNA synthetase 2 (mitochondrial)	2,21	-0,68	-1,00	13	36 124 875
1 A	1427944_at	BE951890	C1qdc1	C1q domain containing 1	2,66	-0,47	-0,73	6	

1	A	1416131_s_at	BB188557	C920006C10Rik	RIKEN cDNA C920006C10 gene	2,05	-1,09	-1,73	15	65 616 730
1	A	1432156_a_at	AK012288	Rnf32	ring finger protein 32	2,33	-0,57	-0,90	5	29 526 864
1	A	1452654_at	BB224658	Zdhhc2	zinc finger, DHHC domain containing 2	2,16	-0,81	-0,51	8	41 922 631
1	A	1447693_s_at	BB350308	Neo1	neogenin	2,09	-1,20	-0,71	9	58 672 818
1	A	1425537_at	AF259672	Ppm1a	protein phosphatase 1A, magnesium dependent, alpha isoform	2,07	-1,00	-0,54	12	73 680 441
1	A	1452700_s_at	AK003597	Kbtbd7	kelch repeat and BTB (POZ) domain containing 7	2,14	-0,50	0,00	14	78 160 665
1	A	1432842_s_at	AK012300	Ywhaq	tyrosine 3-monooxygenase/tryptophan 5-monooxygenase activation protein, theta polypeptide	2,18	0,14	-0,99	12	21 636 820
1	A	1433540_x_at	AW823525	Ppp1cb	protein phosphatase 1, catalytic subunit, beta isoform	2,01	-0,57	-1,00	5	32 735 840
1	A	1436913_at	BB479310	Cdc14a	CDC14 cell division cycle 14 homolog A (S. cerevisiae)	2,24	-0,18	-1,03	3	116 264 540
1	A	1422492_at	BG067254	Cpox	coproporphyrinogen oxidase	2,02	0,09	0,13	16	58 612 834
1	A	1419314_at	BC010745	Tinag	tubulointerstitial nephritis antigen	2,12	-0,01	-0,48	9	76 737 606
1	A	1456273_x_at	BB744467	Tpmt	thiopurine methyltransferase	2,08	-1,01	-0,19	13	47 036 150
1	A	1429088_at	AK007400	Lbh	limb-bud and heart	2,23	-0,41	-0,54	17	72 823 325
1	A	1451257_at	BC022959	Acsl6	acyl-CoA synthetase long-chain family member 6	2,09	-0,83	1,20	11	54 147 626
1	A	1430927_at	AK008389	Ceacam18	CEA-related cell adhesion molecule 1	2,18	-0,18	-1,52	7	43 502 780
1	A	1449382_at	NM_133661	Slc6a12	solute carrier family 6 (neurotransmitter transporter, betaine/GABA), member 12	2,05	-0,19	-0,16	6	121 312 322
1	A	1443115_at	BB465968	Tgfbr2	transforming growth factor, beta receptor II	2,05	-0,13	-0,21	9	115 936 404
1	A	1450717_at	NM_007447	Ang1	angiogenin, ribonuclease A family, member 1	2,13	0,03	-0,50	14	50 018 363
1	A	1439638_at	BB336138	Erbb2ip	Erbb2 interacting protein	2,07	-1,13	-1,32	13	104 939 168
1	A	1459143_at	BB667403	Chchd3	coiled-coil-helix-coiled-coil-helix domain containing 3	2,04	-0,89	-0,64	6	32 722 384
1	A	1421052_a_at	NM_009214	Sms	spermine synthase	2,24	-0,02	0,17	X	152 788 058
1	A	1427035_at	BB399837	Slc39a14	solute carrier family 39 (zinc transporter), member 14	2,12	0,10	-0,87	14	69 038 548
1	A	1456712_at	AV231984	Lcorl	ligand dependent nuclear receptor corepressor-like	2,06	-1,27	-1,71	5	45 987 943
1	A	1439065_x_at	C77501	C230078M14Rik	RIKEN cDNA C230078M14Rik gene	2,14	-1,21	0,66	1	101 927 911
1	A	1438050_x_at	BG966742	Tspan12	tetraspanin 12	2,02	-0,35	0,67	6	21 721 395
1	A	1428643_at	AK015544	Mgat5	mannoside acetylglucosaminyltransferase 5	2,06	-0,71	0,21	1	129 134 307
1	A	1447567_at	BM118129	Odz3	odd Oz/ten-m homolog 3 (Drosophila)	2,04	-0,43	-0,53	8	49 726 507
1	A	1415937_s_at	BC026823	Pdcd6ip	programmed cell death 6 interacting protein	2,04	-1,27	-0,98	9	113 503 632
1	A	1429146_at	BF011349	6620401M08Rik	RIKEN cDNA 6620401M08 gene	2,37	-1,87	-1,13	7	51 865 094
1	A	1454870_x_at	BB251205	Gpr172b	G protein-coupled receptor 172B	2,15	-0,08	-1,07	15	76 366 197
1	B	1450380_at	AF353717	Epdr2	ependymin related protein 2 (zebrafish)	6,97	1,28	1,09	13	19 599 170
1	B	1421815_at	AF353717	Epdr2	ependymin related protein 2 (zebrafish)	6,30	1,25	0,99	13	19 599 170
1	B	1449492_a_at	NM_010702	Lect2	leukocyte cell-derived chemotaxin 2	5,65	0,75	0,15	13	56 552 088
1	B	1451139_at	BC023498	Slc39a4	solute carrier family 39 (zinc transporter), member 4	4,87	2,52	0,93	15	76 439 638
1	B	1460604_at	BE945607	Cybrd1	cytochrome b reductase 1	2,97	0,59	-0,87	2	
1	B	1460329_at	BG066773	B4galt6	UDP-Gal:betaGlcNAc beta 1,4-galactosyltransferase, polypeptide 6	3,67	0,91	-1,44	18	20 829 012
1	B	1450627_at	NM_020332	Ank	progressive ankylosis	3,66	1,08	0,23	15	27 411 401
1	B	1444266_at	AI851612	Hod	homeobox only domain	2,85	1,48	0,56	5	78 161 765
1	B	1459314_at	BB229373	Cdkal1	CDK5 regulatory subunit associated protein 1-like 1	2,98	-0,10	0,19	13	29 332 772
1	B	1457404_at	BM240058	Nfkbiz	nuclear factor of kappa light polypeptide gene enhancer in B-cells inhibitor, zeta	2,98	0,31	-0,57	16	55 736 328
1	B	1455037_at	BB002869	Plxna2	plexin A2	3,11	0,55	0,55	1	196 320 644
1	B	1425040_at	AF354666	Cybrd1	cytochrome b reductase 1	2,63	0,94	0,18	2	
1	B	1450882_s_at	AK010724	Gpr137b	G protein-coupled receptor 137B	2,79	2,52	0,04	13	12 670 181
1	B	1425002_at	BC010462	BC010462	cDNA sequence BC010462	2,98	0,96	0,31	11	120 883 492
1	B	1435708_at	BB355415	Gls	glutaminase	3,03	-0,09	0,17	1	
1	B	1449670_x_at	AW546472	Gpr137b	G protein-coupled receptor 137B	2,53	1,75	-0,38	13	12 670 181
1	B	1452203_at	AV313559	5830411E10Rik	RIKEN cDNA 5830411E10 gene	2,55	0,92	0,28	1	51 413 234
1	B	1417329_at	NM_018824	Slc23a2	solute carrier family 23 (nucleobase transporters), member 2	2,91	0,81	-0,82	2	131 743 937
1	B	1439361_at	BG797614	Myo9a	myosin IXa	2,50	0,94	0,14	9	
1	B	1434599_a_at	BB758095	Tjp2	tight junction protein 2	2,60	0,45	0,41	19	24 161 607
1	B	1427008_at	BC004781	Rnf43	ring finger protein 43	2,71	0,50	-1,15	11	87 516 091
1	B	1457756_at	BB483373	Zfp192	zinc finger protein 192	2,60	2,27	0,42	13	21 520 686
1	B	1446483_at	AV236736	Akr1e1	aldo-keto reductase family 1, member E1	2,48	0,25	0,46	13	4 591 737
1	B	1437892_at	BQ084812	Zfp306	zinc finger protein 306	2,61	0,78	-0,87	13	21 394 560
1	B	1450913_at	BG066773	B4galt6	UDP-Gal:betaGlcNAc beta 1,4-galactosyltransferase, polypeptide 6	3,17	1,27	-0,71	18	20 829 012
1	B	1436556_at	BB534983	Tmem139	transmembrane protein 139	2,85	1,94	-1,33	6	42 191 565
1	B	1421108_at	NM_053096	Cml2	camello-like 2	2,66	0,51	0,48	6	85 831 056
1	B	1434129_s_at	BG917242	Lhfpl2	lipoma HMGIC fusion partner-like 2	2,68	0,10	-0,28	13	95 158 548

1	B	1451600_s_at	BC019147	LOC13909		2,50	0,22	0,66	8	107 972 898
1	B	1445629_at	AI642520	Slc35d1	solute carrier family 35 (UDP-glucuronic acid/UDP-N-acetylgalactosamine dual transporter), member D1	2,28	1,30	-0,31	4	102 669 649
1	B	1421161_at	NM_007568	Btc	betacellulin, epidermal growth factor family member	2,49	0,40	0,02	5	92 432 460
1	B	1417593_at	NM_019742	Tusc2	tumor suppressor candidate 2	2,27	0,45	-0,02	9	107 421 355
1	B	1417649_at	NM_009876	Cdkn1c	cyclin-dependent kinase inhibitor 1C (P57)	2,26	0,79	-0,68	7	143 267 729
1	B	1453422_a_at	AK003858	1110020G09Rik	RIKEN cDNA 1110020G09 gene	2,56	0,30	0,42	15	8 998 562
1	B	1431356_at	AK019889	6430710C18Rik	RIKEN cDNA 6430710C18 gene	2,15	0,20	-0,11	2	
1	B	1439255_s_at	BB726971	Gpr137b	G protein-coupled receptor 137B	2,39	1,92	0,09	13	12 670 181
1	B	1456927_at	BB367890	Mast2	microtubule associated serine/threonine kinase 2	2,31	0,00	-0,66	4	115 804 694
1	B	1421312_a_at	NM_010630	Kifc2	kinesin family member C2	2,20	0,20	-0,53	15	76 487 910
1	B	1428527_at	AK011015	Snx7	sorting nexin 7	2,49	1,35	-0,27	3	117 773 499
1	B	1438074_at	AU024481	2210010C17Rik	RIKEN cDNA 2210010C17 gene	2,19	0,70	1,37	7	19 095 826
1	B	1437378_x_at	BB224405	Scarb1	scavenger receptor class B, member 1	2,24	0,74	-1,05	5	125 566 070
1	B	1441794_at	BB797041	4933437K13Rik	RIKEN cDNA 4933437K13 gene	2,33	0,78	-0,09	16	11 319 225
1	B	1428662_a_at	AK009007	Hod	homeobox only domain	2,28	0,84	-0,06	5	78 161 765
1	B	1447411_at	AI506883	Ugdh	UDP-glucose dehydrogenase	2,20	0,77	-1,30	5	65 692 358
1	B	1431960_at	AK019911	Wwox	WW domain-containing oxidoreductase	2,02	0,26	-0,73	8	117 325 672
1	B	1439342_at	AV353028	Clpx	caseinolytic peptidase X (E.coli)	2,06	1,04	0,36	9	65 092 314
1	B	1419734_at	NM_007393	Actb	actin, beta, cytoplasmic	2,24	1,14	0,99	5	143 168 255
1	B	1417559_at	BB478992	Sfxn1	sideroflexin 1	2,26	-0,05	0,73	13	54 081 498
1	B	1416252_at	NM_134115	Stk38	serine/threonine kinase 38	2,27	0,65	-0,60	17	28 698 475
1	B	1460684_at	BC014769	Tm7sf2	transmembrane 7 superfamily member 2	2,34	0,71	0,39	19	6 062 821
1	B	1445589_at	BM940232	Slc23a2	solute carrier family 23 (nucleobase transporters), member 2	2,22	2,86	0,77	2	131 743 937
1	B	1445062_at	BB457876	Elovl6	ELOVL family member 6, elongation of long chain fatty acids (yeast)	2,23	2,01	-0,40	3	129 524 407
1	B	1448689_at	NM_025846	Rras2	related RAS viral (r-ras) oncogene homolog 2	2,44	0,44	0,36	7	113 837 968
1	B	1457673_at	AI643819	2610005L07Rik	RIKEN cDNA 2610005L07 gene	2,08	-0,07	1,00	8	20 272 528
1	B	1434846_at	AW557010	1700065A05Rik	RIKEN cDNA 1700065A05 gene	2,58	0,33	-0,52	4	
1	B	1429847_a_at	AK014717	4833418A01Rik	RIKEN cDNA 4833418A01 gene	2,14	0,90	0,74	2	70 856 690
1	B	1417403_at	NM_130450	Elovl6	ELOVL family member 6, elongation of long chain fatty acids (yeast)	2,23	1,12	-0,34	3	129 524 407
1	B	1428821_at	AK010891	Agpat2	1-acylglycerol-3-phosphate O-acyltransferase 2 (lysophosphatidic acid acyltransferase, beta)	2,26	0,58	-0,94	2	26 415 595
1	B	1421340_at	NM_008580	Map3k5	mitogen activated protein kinase kinase kinase 5	2,00	0,05	-0,28	10	19 623 898
1	B	1427910_at	AK003744	Cst6	cystatin E/M	2,07	0,63	-0,65	19	5 344 704
1	B	1452213_at	BM239615	Tex2	testis expressed gene 2	2,03	0,09	-0,77	11	106 318 229
1	B	1432918_at	AK014868	4921511E18Rik	RIKEN cDNA 4921511E18 gene	2,04	1,62	0,27	X	
1	B	1444182_at	AV376910	2210010C17Rik	RIKEN cDNA 2210010C17 gene	2,20	0,58	-0,72	7	19 095 826
1	B	1424183_at	BG070487	Acat1	acetyl-Coenzyme A acetyltransferase 1	2,23	0,41	0,23	9	53 342 916
1	B	1424779_at	AK005026	D10Ucla1	DNA segment, Chr 10, University of California at Los Angeles 1	2,08	1,33	-0,97	10	66 406 864
1	B	1424865_at	BC010821	Pyy	peptide YY	2,08	0,73	-0,32	11	101 922 766
1	B	1424167_a_at	BC006809	Pmm1	phosphomannomutase 1	2,05	0,53	-1,55	15	81 778 366
1	B	1450787_at	NM_016691	Clcn5	chloride channel 5	2,00	0,92	-0,15	X	6 316 433
1	B	1436753_at	BB317588	Adck5	aarF domain containing kinase 5	2,25	1,51	-0,55	15	76 403 613
1	B	1452353_at	BB762731	Gpr155	G protein-coupled receptor 155	2,01	0,63	0,08	2	
1	C	1438294_at	BG067317	Atxn1	ataxin 1	5,06	0,27	-4,36	13	45 565 864
1	C	1430274_a_at	AK018331	Stard3nl	STARD3 N-terminal like	3,95	-1,24	-3,20	13	19 365 142
1	C	1455901_at	AI642069	Chpt1	choline phosphotransferase 1	3,73	-1,32	-3,47	10	87 903 332
1	C	1422493_at	BG067254	Cpox	coproporphyrinogen oxidase	3,68	0,29	-2,43	16	58 612 834
1	C	1457619_at	BB743970	Ces6	carboxylesterase 6	3,62	1,45	-2,62	8	107 623 136
1	C	1417040_a_at	NM_016778	Bok	Bcl-2-related ovarian killer protein	3,19	0,02	-3,15	1	95 516 107
1	C	1428851_at	AK005003	1300014I06Rik	RIKEN cDNA 1300014I06 gene	3,06	-0,02	-2,53	13	34 635 304
1	C	1424780_a_at	AK005026	D10Ucla1	DNA segment, Chr 10, University of California at Los Angeles 1	3,07	-0,63	-2,38	10	66 406 864
1	C	1440584_at	AV377077	9130221L21Rik	RIKEN cDNA 9130221L21 gene	3,12	-1,57	-3,56	13	
1	C	1424781_at	AK005026	D10Ucla1	DNA segment, Chr 10, University of California at Los Angeles 1	2,88	-0,11	-2,30	10	66 406 864
1	C	1437864_at	BE632137	Adipor2	adiponectin receptor 2	3,24	-0,12	-3,50	6	119 318 770
1	C	1416645_a_at	NM_007423	Afp	alpha fetoprotein	3,10	0,22	-4,68	5	91 565 936
1	C	1438937_x_at	AI385586	Rnase4	ribonuclease, RNase A family 4	3,01	-0,16	-6,12	14	50 013 146
1	C	1428921_at	AK021189	2810021B07Rik	RIKEN cDNA 2810021B07 gene	3,01	0,55	-1,78	13	17 480 554
1	C	1433976_at	BI249740	D10Ucla1	DNA segment, Chr 10, University of California at Los Angeles 1	2,65	-0,31	-2,65	10	66 406 864
1	C	1427374_at	AF027131	Muc3	mucin 3, intestinal	3,40	0,02	-4,15	5	
1	C	1451588_at	BC014724	1810022C23Rik	RIKEN cDNA 1810022C23 gene	2,60	-0,04	-1,90	13	34 954 078

1	C	1425099_a_at	BC011080	Arntl	aryl hydrocarbon receptor nuclear translocator-like	2,79	0,93	-2,62	7	112 998 645
1	C	1422914_at	NM_022435	Sp5	trans-acting transcription factor 5	2,94	0,32	-4,39	2	70 275 761
1	C	1429555_at	AA408371	Cldnd1	claudin domain containing 1	2,49	-0,63	-2,59	16	58 670 586
1	C	1421037_at	BG070037	Npas2	neuronal PAS domain protein 2	3,00	-0,89	-3,17	1	39 138 757
1	C	1460232_s_at	NM_013821	Hsd3b6	hydroxy-delta-5-steroid dehydrogenase, 3 beta- and steroid delta-isomerase 6	2,66	0,05	-3,86	3	98 934 565
1	C	1433527_at	BB080732	Ireb2	iron responsive element binding protein 2	2,26	-0,06	-2,64	9	54 661 925
1	C	1416267_at	NM_019708	Scoc	short coiled-coil protein	2,38	-0,99	-4,52	8	86 324 599
1	C	1424649_a_at	BC025461	Tspan8	tetraspanin 8	2,06	-0,28	-1,96	10	115 221 393
1	C	1447227_at	AI504711	Slc40a1	solute carrier family 40 (iron-regulated transporter), member 1	2,14	-0,33	-2,80	1	45 852 629
1	C	1456789_at	AW491540	Zfp462	zinc finger protein 462	2,10	-0,66	-2,69	4	55 041 276
1	C	1436848_x_at	AV348702	Impa1	inositol (myo)-1(or 4)-monophosphatase 1	2,20	-1,25	-3,85	3	10 296 071
1	C	1428252_at	AA881383	Chmp2b	chromatin modifying protein 2B	2,11	-0,24	-1,91	16	65 457 392
1	C	1435446_a_at	BF180212	Chpt1	choline phosphotransferase 1	2,22	-0,33	-2,58	10	87 903 332
1	C	1443078_at	AV321994	6030439D06Rik	RIKEN cDNA 6030439D06 gene	2,07	-1,71	-2,78	8	118 573 703
1	C	1460203_at	NM_010585	Itpr1	inositol 1,4,5-triphosphate receptor 1	2,07	0,28	-2,69	6	108 178 894
1	C	1448612_at	NM_018754	Sfn	stratifin	2,16	0,75	-2,33	4	132 872 631
1	C	1433741_at	BB256012	Cd38	CD38 antigen	2,02	0,55	-3,60	5	44 157 097
1	C	1434387_at	AV251853	Itfg3	integrin alpha FG-GAP repeat containing 3	2,03	0,34	-2,02	17	25 940 312
1	C	1420809_a_at	NM_019769	1500003O03Rik	RIKEN cDNA 1500003O03 gene	2,10	-1,07	-2,10	2	119 239 147
1	C	1434754_at	AU067654	Garnl4	GTPase activating RANGAP domain-like 4	2,08	0,03	-2,23	11	74 199 678
1	C	1437918_at	AV374644	4930539E08Rik	RIKEN cDNA 4930539E08 gene	2,14	-1,64	-3,85	17	28 623 985
1	C	1431293_a_at	AK012260	Cldnd1	claudin domain containing 1	2,44	-1,75	-3,76	16	58 670 586
1	C	1437595_at	BB531414	E030010A14Rik	RIKEN cDNA E030010A14 gene	2,45	1,19	-4,98	19	24 741 104
1	C	1431833_a_at	AK004902	Hmgcs2	3-hydroxy-3-methylglutaryl-Coenzyme A synthase 2	2,15	0,55	-1,80	3	98 365 839
1	D	1456573_x_at	BB205930	Nnt	nicotinamide nucleotide transhydrogenase	4,69	-0,54	5,01	13	120 453 189
1	D	1449316_at	NM_134127	Cyp4f15	cytochrome P450, family 4, subfamily f, polypeptide 15	4,46	0,92	2,71	17	32 424 968
1	D	1460194_at	NM_010726	Phyh	phytanoyl-CoA hydroxylase	3,77	1,01	2,34	2	4 836 302
1	D	1416105_at	BC008518	Nnt	nicotinamide nucleotide transhydrogenase	3,27	-0,04	6,09	13	120 453 189
1	D	1435691_at	BB209207	C630028N24Rik	RIKEN cDNA C630028N24 gene	2,64	-0,12	1,66	9	54 715 419
1	D	1447439_at	AI848394	1700023E05Rik	RIKEN cDNA 1700023E05 gene	2,80	1,74	1,97	5	78 090 797
1	D	1449076_x_at	NM_134052	Adi1	acireductone dioxygenase 1	2,21	-0,79	2,78	12	29 261 474
1	D	1428973_s_at	AK007178	0610012D17Rik	RIKEN cDNA 0610012D17 gene	2,47	-0,84	3,29	16	32 339 490
1	D	1419072_at	NM_026672	Gstm7	glutathione S-transferase, mu 7	2,17	0,41	1,79	3	108 054 386
1	D	1451461_a_at	BC008184	Aldoc	aldolase 3, C isoform	2,26	0,59	3,67	11	78 140 392
1	D	1416776_at	NM_016669	Crym	crystallin, mu	2,15	-1,31	3,06	7	119 977 532
1	D	1439143_at	BB312992	9310367 E06		2,14	-0,31	2,57	14	
1	D	1441709_at	BB771579	Slc11a2	solute carrier family 11 (proton-coupled divalent metal ion transporters), member 2	2,04	0,62	1,52	15	100 215 935
2	_	1442151_at	BM238456	D230040A04Rik	RIKEN cDNA D230040A04 gene	-5,04	-4,80	0,97	13	13 860 001
2	_	1422645_at	AJ306425	Hfe	hemochromatosis	-5,70	-7,32	0,05	13	23 711 307
2	_	1443137_at	BB534298			-3,73	-2,92	-1,36	?	
2	_	1426464_at	W13191	Nr1d1	nuclear receptor subfamily 1, group D, member 1	-2,53	-2,20	1,38	11	98 584 024
3	_	1442513_at	AU041573	BC016423	cDNA sequence BC016423	-4,30	1,03	0,24	13	
3	_	1418580_at	BC024872	Rtp4	receptor transporter protein 4	-4,22	-0,03	1,43	16	23 525 275
3	_	1435529_at	BM245961	2010002M12Rik	RIKEN cDNA 2010002M12 gene	-3,50	0,22	2,08	19	34 683 047
3	_	1457053_at	BB056354	BC016423	cDNA sequence BC016423	-3,09	-0,12	0,63	13	
3	_	1436562_at	BG063981	Ddx58	DEAD (Asp-Glu-Ala-Asp) box polypeptide 58	-3,22	0,88	1,12	4	40 394 460
3	_	1450783_at	NM_008331	Ifit1	interferon-induced protein with tetratricopeptide repeats 1	-2,92	-0,28	1,80	19	34 706 885
3	_	1421008_at	BB741897	Rsad2	radical S-adenosyl methionine domain containing 2	-2,97	0,65	2,31	12	27 029 020
3	_	1426440_at	AK009385	Dhrs7	dehydrogenase/reductase (SDR family) member 7	-2,81	-0,40	-0,52	12	73 569 193
3	_	1422903_at	NM_010745	Ly86	lymphocyte antigen 86	-2,57	-0,28	0,88	13	37 352 810
3	_	1418293_at	NM_008332	Ifit2	interferon-induced protein with tetratricopeptide repeats 2	-2,79	0,95	1,79	19	34 616 942
3	_	1451777_at	BC013672	BC013672	cDNA sequence BC013672	-2,61	-0,37	1,39	8	64 886 557
3	_	1426454_at	AK002516	Arhgdib	Rho, GDP dissociation inhibitor (GDI) beta	-2,61	-0,17	0,87	6	136 887 904
3	_	1453196_a_at	BQ033138	Oasl2	2'-5' oligoadenylate synthetase-like 2	-2,39	-0,22	0,87	5	115 157 934
3	_	1455132_at	AV312663	A430107D22Rik	RIKEN cDNA A430107D22 gene	-2,28	-0,56	1,26	17	
3	_	1437176_at	AV277444	AI451557	expressed sequence AI451557	-2,36	0,80	1,08	8	97 414 195
3	_	1438676_at	BM241485	Mpa2l	macrophage activation 2 like	-2,23	0,92	1,62	5	105 455 208
3	_	1434449_at	BB193413	Aqp4	aquaporin 4	-2,82	-1,06	1,93	18	15 535 849
3	_	1436312_at	AV317621	Zfpn1a1	zinc finger protein, subfamily 1A, 1 (Ikaros)	-2,63	-0,71	1,52	11	11 586 215

3	_	1416246_a_at	BC002136	Coro1a	coronin, actin binding protein 1A	-2,32	-0,10	1,28	7	126 490 922
3	_	1438037_at	AW208668	Ids	iduronate 2-sulfatase	-2,29	0,03	0,78	X	66 603 736
3	_	1452544_x_at	J00406	H2-D1	histocompatibility 2, D region locus 1	-2,17	0,52	6,80	17	
3	_	1450981_at	BI663014	Cnn2	calponin 2	-2,36	-1,18	1,80	10	79 391 762
3	_	1424518_at	BC020489	BC020489	cDNA sequence BC020489	-2,21	0,11	0,36	15	
3	_	1417699_at	AV325174	Gtf2f1	general transcription factor IIF, polypeptide 1	-2,14	0,94	0,90	17	56 688 526
3	_	1417190_at	AW989410	Pbef1	pre-B-cell colony-enhancing factor 1	-2,27	0,53	1,15	12	33 405 721
3	_	1417793_at	NM_019440	Iigp2	interferon inducible GTPase 2	-2,05	0,70	1,57	11	58 015 831
3	_	1450484_a_at	AK004595	Tyki	thymidylate kinase family LPS-inducible member	-2,25	-0,02	2,07	12	27 055 649
3	_	1439114_at	BB234229	BC013672	cDNA sequence BC013672	-2,11	0,37	1,78	8	
3	_	1429564_at	AK002387	Pcgf5	polycomb group ring finger 5	-2,23	1,62	1,54	19	36 444 210
3	_	1417056_at	NM_011189	Psme1	proteasome (prosome, macropain) 28 subunit, alpha	-2,18	1,23	1,89	14	54 532 564
3	_	1419697_at	NM_019494	Cxcl11	chemokine (C-X-C motif) ligand 11	-2,08	0,17	2,27	5	93 434 746
3	_	1424181_at	BC010489	38961	septin 6	-2,18	-0,03	1,26	X	33 343 689
3	_	1418191_at	NM_011909	Usp18	ubiquitin specific peptidase 18	-2,11	0,11	0,68	6	121 211 575
3	_	1449556_at	NM_010398	H2-T23	histocompatibility 2, T region locus 23	-2,09	0,07	0,02	17	35 638 031
3	_	1451306_at	BC006933	Cdca7l	cell division cycle associated 7 like	-2,03	-0,03	-0,35	12	118 285 869
3	_	1426596_a_at	BB821035	Smn1	survival motor neuron 1	-2,09	0,65	1,31	13	101 225 150
3	_	1436058_at	BB132493	Rsad2	radical S-adenosyl methionine domain containing 2	-2,02	0,05	1,27	12	27 029 020
3	_	1433963_a_at	BG066664	BC032204	cDNA sequence BC032204	-2,04	-0,41	1,47	19	7 066 021
3	_	1451787_at	AF128849	Cyp2b10	cytochrome P450, family 2, subfamily b, polypeptide 10	-7,06	-0,11	2,37	7	25 606 435
3	_	1422257_s_at	NM_009998	Cyp2b10	cytochrome P450, family 2, subfamily b, polypeptide 10	-6,39	-0,87	1,57	7	25 606 435
3	_	1425645_s_at	AF128849	Cyp2b10	cytochrome P450, family 2, subfamily b, polypeptide 10	-5,90	-0,55	1,43	7	25 606 435
3	_	1445411_at	BI076621			-3,21	0,24	2,00	?	
3	_	1454268_a_at	AK018713	Cyba	cytochrome b-245, alpha polypeptide	-3,60	-0,17	0,68	8	125 310 865
3	_	1422120_at	AY034479	Eaf2	ELL associated factor 2	-2,46	-0,29	0,69	16	36 712 124
3	_	1438024_at	AW554392	6230416A05Rik	RIKEN cDNA 6230416A05 gene	-2,75	-0,10	0,30	13	
3	_	1428393_at	AK003046	Nrn1	neuritin 1	-2,91	0,11	3,35	13	36 733 092
3	_	1422660_at	AY052560	LOC671237		-2,83	0,34	0,79	Un	
3	_	1417104_at	BC001999	Emp3	epithelial membrane protein 3	-2,70	-0,17	1,03	7	45 786 105
3	_	1449410_a_at	NM_013525	Gas5	growth arrest specific 5	-2,67	0,27	-0,50	1	
3	_	1427119_at	AV066321	Spink4	serine peptidase inhibitor, Kazal type 4	-2,88	0,43	-0,89	4	41 108 726
3	_	1437180_at	BE824567	6530403A03Rik	RIKEN cDNA 6530403A03 gene	-2,35	0,14	0,03	13	38 212 404
3	_	1451358_a_at	AF212320	Racgap1	Rac GTPase-activating protein 1	-2,50	1,74	1,25	15	99 448 532
3	_	1460121_at	BI076710	9630010G10Rik	RIKEN cDNA 9630010G10 gene	-2,72	-0,44	3,41	17	
3	_	1416235_at	AW476171	Lrrc59	leucine rich repeat containing 59	-2,64	1,01	0,89	11	94 445 913
3	_	1435737_a_at	BQ032232	Nde1	nuclear distribution gene E homolog 1 (A nidulans)	-2,50	0,14	1,01	16	14 083 052
3	_	1416035_at	BB269715	Hif1a	hypoxia inducible factor 1, alpha subunit	-2,43	0,12	2,82	12	74 826 867
3	_	1416927_at	AW495711	Trp53inp1	transformation related protein 53 inducible nuclear protein 1	-2,56	-0,24	-0,37	4	11 083 587
3	_	1425151_a_at	BC019525	Noxo1	NADPH oxidase organizer 1	-2,40	0,58	0,77	17	24 425 837
3	_	1448310_at	NM_019987	Ick	intestinal cell kinase	-2,28	0,26	1,79	9	77 899 092
3	_	1421420_at	AF215982	Ccr10	chemokine (C-C motif) receptor 10	-2,80	0,25	2,41	11	100 989 071
3	_	1416961_at	NM_009773	Bub1b	budding uninhibited by benzimidazoles 1 homolog, beta (S. cerevisiae)	-2,39	0,87	1,52	2	118 289 696
3	_	1460681_at	BC024320	Ceacam2	CEA-related cell adhesion molecule 2	-2,42	0,39	-2,04	7	25 224 802
3	_	1457350_at	BG298986	Per2	period homolog 2 (Drosophila)	-2,50	0,50	1,38	1	93 246 389
3	_	1430127_a_at	AK007904	Ccnd2	cyclin D2	-2,76	1,02	0,98	6	127 091 327
3	_	1419423_at	NM_138673	Stab2	stabilin 2	-2,19	-0,11	2,19	10	86 271 008
3	_	1423746_at	BC016252	Txndc5	thioredoxin domain containing 5	-2,76	0,34	1,26	13	38 507 740
3	_	1426004_a_at	AF114266	Tgm2	transglutaminase 2, C polypeptide	-2,26	0,17	0,59	2	157 807 847
3	_	1423090_x_at	AV216331	Slc37a2	solute carrier family 37 (glycerol-3-phosphate transporter), member 2	-2,17	-0,14	0,11	9	36 978 819
3	_	1429672_at	BB234837	Arhgap15	Rho GTPase activating protein 15	-2,48	-0,31	0,73	2	43 570 835
3	_	1460682_s_at	BC024320	Ceacam2	CEA-related cell adhesion molecule 2	-2,54	0,34	-1,73	7	25 224 802
3	_	1422029_at	AF099052	Ccl20	chemokine (C-C motif) ligand 20	-2,22	0,62	2,29	1	82 995 805
3	_	1422422_at	NM_010039	Defcr4	defensin related cryptdin 4	-2,46	-0,32	1,55	8	22 657 071
3	_	1451823_at	AY008277	Clca4	chloride channel calcium activated 4	-2,57	0,52	0,68	3	144 760 127
3	_	1425675_s_at	M77196	Ceacam1	CEA-related cell adhesion molecule 1	-2,29	0,56	-1,11	7	25 170 462
3	_	1455956_x_at	AV310588	Ccnd2	cyclin D2	-2,38	-0,02	0,10	6	127 091 327
3	_	1416122_at	NM_009829	Ccnd2	cyclin D2	-2,60	1,00	0,21	6	127 091 327
3	_	1415741_at	NM_011626	Tmem165	transmembrane protein 165	-2,12	-0,36	-0,24	5	77 258 654
3	_	1439475_at	BB667905	AI929863	expressed sequence AI929863	-2,22	0,53	-2,36	13	
3	_	1451611_at	BC024581	Hrasls3	HRAS like suppressor 3	-2,13	0,78	-0,58	19	7 624 502

3	_	1450099_a_at	NM_008094	Gba	glucosidase, beta, acid	-2,19	0,40	0,09	3	89 288 866
3	_	1418230_a_at	BC005621	Lims1	LIM and senescent cell antigen-like domains 1	-2,17	0,60	1,64	10	57 718 903
3	_	1450217_at	BG867337	Ccl28	chemokine (C-C motif) ligand 28	-2,57	0,50	5,51	13	1 330 631
3	_	1452661_at	AK011596	Tfrc	transferrin receptor	-2,07	-0,04	0,41	16	32 528 767
3	_	1416234_at	AW476171	Lrrc59	leucine rich repeat containing 59	-2,24	0,23	0,47	11	94 445 913
3	_	1438211_s_at	BB550183	Dbp	D site albumin promoter binding protein	-2,08	-0,12	2,08	7	45 573 273
3	_	1448229_s_at	NM_009829	Ccnd2	cyclin D2	-2,39	0,31	-0,94	6	127 091 327
3	_	1432466_a_at	AK019319	Apoe	apolipoprotein E	-2,15	0,13	0,34	7	18 854 794
3	_	1449591_at	NM_007609	Casp4	caspase 4, apoptosis-related cysteine peptidase	-2,25	-0,33	1,30	9	5 308 873
3	_	1428480_at	AV307110	Cdca8	cell division cycle associated 8	-2,32	0,25	0,60	4	124 420 768
3	_	1420479_a_at	BG064031	Nap1l1	nucleosome assembly protein 1-like 1	-2,12	-0,07	1,72	10	110 884 726
3	_	1423986_a_at	BC010238	Scotin	scotin gene	-2,06	0,14	0,48	9	108 896 032
3	_	1444283_at	BB223831	Gimap7	GTPase, IMAP family member 7	-2,19	-0,06	1,38	6	48 648 206
3	_	1455316_x_at	AI987693	Kcnh6	potassium voltage-gated channel, subfamily H (eag-related), member 6	-2,09	-0,99	2,73	11	105 824 292
3	_	1435431_at	AW558989	2310047M15Rik	RIKEN cDNA 2310047M15 gene	-2,17	0,76	1,65	13	
3	_	1421920_a_at	NM_009913	Ccr9	chemokine (C-C motif) receptor 9	-2,07	0,31	0,74	9	123 527 136
3	_	1416958_at	NM_011584	Nr1d2	nuclear receptor subfamily 1, group D, member 2	-2,31	-0,71	1,14	14	16 997 183
3	_	1455577_at	BE196980	Ccl28	chemokine (C-C motif) ligand 28	-2,20	0,36	-0,76	13	1 330 631
3	_	1428850_x_at	AK004342	Cd99	CD99 antigen	-2,16	-0,41	-2,73	4	
3	_	1426657_s_at	L21027	Phgdh	3-phosphoglycerate dehydrogenase	-2,02	0,64	2,40	3	98 398 575
3	_	1460259_s_at	AF108501	Clca2	chloride channel calcium activated 2	-2,07	0,36	1,10	3	144 734 469
3	_	1416384_a_at	NM_021538	Cope	coatomer protein complex, subunit epsilon	-2,03	0,38	-0,48	8	73 231 773
3	_	1439539_at	BB091955	Tram2	translocating chain-associating membrane protein 2	-2,12	0,30	-0,27	1	20 986 529
3	_	1434248_at	BM243756	Prkch	protein kinase C, eta	-2,05	-0,16	0,98	12	74 503 878
3	_	1437044_a_at	BB241507	Gba	glucosidase, beta, acid	-2,05	-0,43	-0,09	3	89 288 866
3	_	1437025_at	AV313615	Cd28	CD28 antigen	-2,12	-0,18	0,99	1	60 691 013
3	_	1456377_x_at	AV010467	LOC632329		-2,22	-0,49	1,04	1	
3	_	1455454_at	BG073853	Akr1c19	aldo-keto reductase family 1, member C19	-5,01	-0,65	1,08	13	4 232 985
3	_	1425469_a_at	BC003855	9030208C03Rik	RIKEN cDNA 9030208C03 gene	-4,89	1,98	6,27	4	
3	_	1417651_at	NM_007815	Cyp2c29	cytochrome P450, family 2, subfamily c, polypeptide 29	-3,43	-0,26	-1,34	19	39 340 413
3	_	1452565_x_at	M11024	LOC641050		-3,65	0,40	5,30	?	
3	_	1428947_at	AK008016	2010001M09Rik	RIKEN cDNA 2010001M09 gene	-3,78	-0,23	1,43	18	35 773 245
3	_	1448511_at	NM_016933	Ptprcap	protein tyrosine phosphatase, receptor type, C polypeptide-associated protein	-3,03	0,06	1,48	19	4 154 645
3	_	1444177_at	AI451538	LOC626058		-3,25	-0,10	5,04	1	
3	_	1427301_at	BE634960	Cd48	CD48 antigen	-2,94	0,26	1,17	1	173 518 729
3	_	1424927_at	BC025083	Glipr1	GLI pathogenesis-related 1 (glioma)	-3,81	0,79	2,02	10	111 389 566
3	_	1427891_at	BB667753	Gimap6	GTPase, IMAP family member 6	-2,93	-0,75	1,35	6	48 631 168
3	_	1417426_at	NM_011157	Prg1	proteoglycan 1, secretory granule	-2,93	0,06	1,38	10	61 889 784
3	_	1417597_at	NM_007642	Cd28	CD28 antigen	-3,43	-0,41	1,73	1	60 691 013
3	_	1420782_at	NM_011608	Tnfrsf17	tumor necrosis factor receptor superfamily, member 17	-2,97	-0,35	0,61	16	11 227 386
3	_	1424065_at	BG064496	Edem1	ER degradation enhancer, mannosidase alpha-like 1	-2,96	0,78	-0,21	6	108 794 428
3	_	1416957_at	NM_011136	Pou2af1	POU domain, class 2, associating factor 1	-3,62	0,22	2,55	9	50 965 919
3	_	1426174_s_at	S69212	Ighg	Immunoglobulin heavy chain (gamma polypeptide)	-3,86	1,47	2,58	12	113 755 089
3	_	1429682_at	AK015259	4930431B09Rik	RIKEN cDNA 4930431B09 gene	-3,30	0,61	3,00	3	100 600 588
3	_	1425927_a_at	AF375476	Atf5	activating transcription factor 5	-3,15	-1,17	1,73	7	44 680 299
3	_	1456064_at	AI323624	AI504432	expressed sequence AI504432	-2,76	-0,82	1,27	3	107 167 559
3	_	1420928_at	BG075800	St6gal1	beta galactoside alpha 2,6 sialyltransferase 1	-2,63	-0,44	1,01	16	23 140 095
3	_	1434068_s_at	BE688410	AI662270	expressed sequence AI662270	-2,93	0,28	2,19	11 ?	
3	_	1421653_a_at	NM_134051	Igh-VJ558	immunoglobulin heavy chain (J558 family)	-2,94	1,03	1,59	12	113 706 525
3	_	1423182_at	AK004668	Tnfrsf13b	tumor necrosis factor receptor superfamily, member 13b	-2,56	0,11	1,97	11	60 957 029
3	_	1416926_at	AW495711	Trp53inp1	transformation related protein 53 inducible nuclear protein 1	-2,95	-0,99	-0,90	4	11 083 587
3	_	1452535_at	Z95476	Igh-1a	immunoglobulin heavy chain 1a (serum IgG2a)	-2,62	0,64	0,21	12	113 876 327
3	_	1448617_at	NM_007651	Cd53	CD53 antigen	-2,56	0,53	1,92	3	106 887 989
3	_	1448021_at	AA266723	4930431B09Rik	RIKEN cDNA 4930431B09 gene	-3,80	0,79	2,14	3	100 597 118
3	_	1417500_a_at	BC016492	Tgm2	transglutaminase 2, C polypeptide	-2,67	0,65	1,21	2	157 807 847
3	_	1429381_x_at	AK007826	LOC238447		-2,87	1,16	1,48	12	113 706 562
3	_	1440196_at	BB207611	Arhgap18	Rho GTPase activating protein 18	-2,28	-0,36	1,30	10	26 461 927
3	_	1416416_x_at	NM_010358	Gstm1	glutathione S-transferase, mu 1	-2,46	1,26	1,98	3	108 140 313
3	_	1425584_x_at	BC010605			-2,34	0,25	3,49	?	
3	_	1418296_at	NM_008761	Fxyd5	FXYD domain-containing ion transport regulator 5	-2,53	-1,46	1,55	7	30 741 497

3	_	1435560_at	BI554446	Itgal	integrin alpha L	-2,53	-0,75	1,22	7	127 087 557
3	_	1436996_x_at	AV066625	Lzp-s	P lysozyme structural	-2,60	0,55	0,53	10	116 691 906
4	_	1418072_at	NM_023422	Hist1h2bc	histone 1, H2bc	3,06	-4,28	-2,60	13	23 691 663
4	_	1452540_a_at	M25487	Hist1h2bp	histone 1, H2bp	1,52	-2,82	-1,14	13	21 794 979
4	_	1435926_at	BB529627	E030003F13Rik	RIKEN cDNA E030003F13 gene	-0,01	-2,76	-0,82	1	
4	_	1456174_x_at	AV309418	Ndrg1	N-myc downstream regulated gene 1	0,75	-2,59	0,40	15	66 758 990
4	_	1436317_at	BM115569	D230012E17Rik	RIKEN cDNA D230012E17 gene	1,12	-3,06	-1,12	1	
4	_	1429694_at	AK015050	4930402H24Rik	RIKEN cDNA 4930402H24 gene	0,65	-2,84	0,01	2	130 399 302
4	_	1434277_a_at	BG069663	6430570G24		1,87	-2,40	-2,32	11	86 752 622
4	_	1427929_a_at	BG063905	Pdxk	pyridoxal (pyridoxine, vitamin B6) kinase	1,51	-2,39	-0,92	10	77 842 100
4	_	1443620_at	BB212497	Gpc4	glypican 4	0,53	-2,38	1,01	X	48 297 548
4	_	1452207_at	Y15163	Cited2	Cbp/p300-interacting transactivator, with Glu/Asp-rich carboxy-terminal domain, 2	0,27	-2,28	0,69	10	17 412 661
4	_	1427797_s_at	BF580235	Ctse	cathepsin E	-1,71	-5,49	6,93	1	133 465 859
4	_	1419506_at	NM_010282	Ggps1	geranylgeranyl diphosphate synthase 1	-1,83	-3,99	-1,01	13	13 844 574
4	_	1427798_x_at	BF580235			-0,41	-4,93	4,74	?	
4	_	1442750_at	BG067768	B3galnt2	UDP-GalNAc:betaGlcNAc beta 1,3-galactosaminyltransferase, polypeptide 2	-1,84	-3,24	-0,48	13	13 746 799
4	_	1422948_s_at	NM_013550	Hist1h3a	histone 1, H3a	-0,95	-2,83	-1,94	13	23 769 317
4	_	1453840_at	AK005009	Pabpc1	poly A binding protein, cytoplasmic 1	-1,52	-3,18	-0,85	15	36 540 251

Genes were selected as described in Materials and Methods. Values are the mean of 3 S-scores comparing D2KO versus D2WT expression, B6KO versus B6WT expression, and D2WT versus B6WT expression.
