# Supplementary material for: Gene expression profiling of Hfe-/- liver and duodenum in mouse strains with differing susceptibilities to iron loading: identification of transcriptional regulatory targets of Hfe and potential hemochromatosis modifiers
Source: Genome Biol. 2007 Oct 18;8(10):R221. doi: 10.1186/gb-2007-8-10-r221 (PMC2246295; doi:10.1186/gb-2007-8-10-r221)
Supplement: Additional File 3 — Presented is a table listing genes differentially expressed in the liver and/or the duodenum of wild-type D2 and B6 mice. [file gb-2007-8-10-r221-S3.pdf]

Additonal data file 3. Genes differentially expressed between wild-type B6 and D2 liver and/or duodenum.

Cluster	ProbesetID	Genbank	Gene symbol	Description	Liver	Duodenum	Chromosome	Position (kb)
0	1441945_s_at	AV009478	Abhd14a	abhydrolase domain containing 14A	-3,38	2,59	9	106 298 159
0	1424716_at	BB775176	Retsat	retinol saturase (all trans retinol 13,14 reductase)	-3,15	3,18	6	72 528 136
1	1457359_at	BB540672	Inpp4b	inositol polyphosphate-4-phosphatase, type II	0,18	5,41	8	84 610 946
1	1425469_a_at	BC003855	9030208C03Rik	RIKEN cDNA 9030208C03 gene	-0,79	6,27	4	
1	1460606_at	AV050442	Hsd17b13	hydroxysteroid (17-beta) dehydrogenase 13	1,82	6,30	5	104 195 745
1	1450217_at	BG867337	Ccl28	chemokine (C-C motif) ligand 28	-0,32	5,51	13	1 330 631
1	1455735_at	AW259574	Ap1s3	adaptor-related protein complex AP-1, sigma 3	0,87	5,55	1	79 485 908
1	1452565_x_at	M11024	LOC641050		0,36	5,30	?	
1	1440922_at	AI506672	9130208D14Rik	RIKEN cDNA 9130208D14 gene	0,50	4,03	7	105 697 082
1	1429079_a_at	AK009829	2310045N01Rik	RIKEN cDNA 2310045N01 gene	1,56	4,41	8	73 068 708
1	1448211_at	NM_133764	Atp6v0e2	ATPase, H+ transporting, lysosomal V0 subunit E2	1,28	5,57	6	48 467 200
1	1427726_at	X03051	H2D1	histocompatibility 2, D region locus 1	-0,38	4,31	17	
1	1415904_at	BC003305	Lpl	lipoprotein lipase	0,11	3,81	8	71 809 542
1	1448987_at	BB728073	Acadl	acyl-Coenzyme A dehydrogenase, long-chain	0,59	4,52	1	66 764 060
1	1437590_at	BB781167	4833409A17Rik	RIKEN cDNA 4833409A17 gene	0,74	3,93	19	42 800 415
1	1425856_at	BC005570	Cib1	calcium and integrin binding 1 (calmyrin)	1,28	6,95	7	80 100 672
1	1454247_a_at	AK008784	Gpa33	glycoprotein A33 (transmembrane)	-0,11	4,15	1	167 967 134
1	1453345_at	AK014427	Npal1	NIPA-like domain containing 1	1,15	6,10	5	
1	1451461_a_at	BC008184	Aldoc	aldolase 3, C isoform	0,28	3,67	11	78 140 392
1	1424097_at	BC005602	Elovl7	ELOVL family member 7, elongation of long chain fatty acids (yeast)	-0,52	3,75	13	109 335 179
1	1432556_a_at	AK013920	3100002J23Rik	RIKEN cDNA 3100002J23 gene	0,14	5,10	11	58 609 006
1	1419671_a_at	NM_134159	Il17rc	interleukin 17 receptor C	-1,24	4,05	6	113 437 350
1	1418448_at	NM_009101	Rras	Harvey rat sarcoma oncogene, subgroup R	1,25	3,85	7	44 886 054
1	1450218_at	BG867337	Ccl28	chemokine (C-C motif) ligand 28	-0,44	4,14	13	1 330 631
1	1452547_s_at	M16810	H2D1	histocompatibility 2, D region locus 1	0,37	3,48	17	
1	1447341_at	BE456208	D12Ertd551e	DNA segment, Chr 12, ERATO Doi 551, expressed	1,66	3,52	12	116 723 230
1	1428667_at	AW986246	Maoa	monoamine oxidase A	-2,29	3,77	X	15 776 656
1	1417379_at	NM_016721	Iqgap1	IQ motif containing GTPase activating protein 1	1,76	4,01	7	80 586 297
1	1417150_at	NM_010484	Slc6a4	solute carrier family 6 (neurotransmitter transporter, serotonin), member 4	0,03	4,30	11	76 814 791
1	1424748_at	BC021504	Galnt11	UDP-N-acetyl-alpha-D-galactosamine:polypeptide N-acetylgalactosaminyltransferase 11	1,40	5,08	5	24 732 967
1	1456321_at	AI503879	Npal1	NIPA-like domain containing 1	0,63	4,03	5	
1	1424953_at	BC021614	BC021614	cDNA sequence BC021614	1,59	4,35	19	4 057 486
1	1416543_at	NM_010902	Nfe2l2	nuclear factor, erythroid derived 2, like 2	1,21	3,59	2	75 476 357
1	1425584_x_at	BC010605			0,33	3,49	?	
1	1439484_at	BM245060	4930431H11Rik	RIKEN cDNA 4930431H11 gene	0,97	3,63	3	19 415 294
1	1448250_at	BG072972	9030425E11Rik	RIKEN cDNA 9030425E11 gene	-0,68	3,58	9	40 437 032
1	1417849_at	AW413620	Zfp704	zinc finger protein 704	1,73	3,70	3	9 410 092
1	1433675_at	BQ177137	Snord22	small nucleolar RNA, C/D box 22	0,99	4,57	19	
1	1446140_at	BB233975	Pcm1	pericentriolar material 1	1,45	3,85	8	42 738 591
1	1430220_at	AK017802	4833420G17Rik	RIKEN cDNA 4833420G17 gene	1,55	3,88	13	120 581 646
1	1419298_at	NM_008897	Pon3	paraoxonase 3	-0,02	3,23	6	5 170 851
1	1416530_a_at	BC003788	Pnp	purine-nucleoside phosphorylase	-0,56	3,50	14	49 866 475
1	1433465_a_at	BB234337	AI467606	expressed sequence AI467606	-0,63	2,79	7	126 882 583
1	1417220_at	NM_010176	Fah	fumarylacetoacetate hydrolase	-0,43	5,29	7	84 461 355
1	1439156_at	BG971631	Gm962	gene model 962, (NCBI)	1,25	3,43	19	5 568 073
1	1444139_at	BG797099	Ddit4l	DNA-damage-inducible transcript 4-like	-1,43	4,48	3	137 561 122
1	1452228_at	AV209678	Tbc1d23	TBC1 domain family, member 23	0,86	3,36	16	57 090 220
1	1444632_at	BF658882	BC064078	cDNA sequence BC064078	-0,13	3,57	6	128 972 573
1	1427689_a_at	AJ242777	Tnip1	TNFAIP3 interacting protein 1	1,40	2,78	11	54 754 211
1	1421103_at	NM_080708	Bmp2k	BMP2 inducible kinase	-0,10	3,74	5	97 237 990
1	1432901_at	AK018352	BC026657	cDNA sequence BC026657	0,17	3,54	2	
1	1453601_at	AK017247	Zfp710	zinc finger protein 710	1,67	2,97	7	79 899 846
1	1429682_at	AK015259	4930431B09Rik	RIKEN cDNA 4930431B09 gene	-0,20	3,00	3	100 600 588
1	1435069_at	AW493518	BC064078	cDNA sequence BC064078	0,42	3,06	6	128 958 618
1	1445724_at	BB153562	Iqgap1	IQ motif containing GTPase activating protein 1	0,06	4,44	7	80 586 297
1	1421276_a_at	NM_134448	Dst	dystonin	1,88	3,89	1	34 104 877

1	1451547_at	BC023358	0610009A07Rik	RIKEN cDNA 0610009A07 gene	0,72	2,84	10	6 791 663
1	1422230_s_at	NM_007812	Cyp2a5	cytochrome P450, family 2, subfamily a, polypeptide 5	1,03	3,58	7	26 544 100
1	1452433_at	BB662083			0,62	3,50	?	
1	1438651_a_at	BB483357	Agtrl1	angiotensin receptor-like 1	-0,55	2,60	2	84 937 224
1	1442549_at	BB211386	Mbnl3	muscleblind-like 3 (Drosophila)	-0,57	2,93	X	47 358 121
1	1453068_at	BM226301	Prdm2	PR domain containing 2, with ZNF domain	1,81	3,69	4	
1	1423291_s_at	BM231738	Hyou1	hypoxia up-regulated 1	0,83	2,89	9	44 130 579
1	1452918_at	AK011404	D19Ertd737e	DNA segment, Chr 19, ERATO Doi 737, expressed	-1,12	2,91	19	60 253 248
1	1430961_at	AK018067	Zfp292	zinc finger protein 292	0,80	2,84	4	
1	1421179_at	NM_053273	Ttyh2	tweety homolog 2 (Drosophila)	1,46	3,32	11	114 491 557
1	1449254_at	NM_009263	Spp1	secreted phosphoprotein 1	-1,92	3,28	5	104 676 506
1	1416035_at	BB269715	Hif1a	hypoxia inducible factor 1, alpha subunit	-0,08	2,82	12	74 826 867
1	1419230_at	NM_010661	Krt12	keratin 12	-0,91	3,37	11	99 231 755
1	1419022_a_at	NM_023119	Eno1	enolase 1, alpha non-neuron	0,96	2,76	4	149 081 054
1	1452548_x_at	M16810	H2D1	histocompatibility 2, D region locus 1	0,49	2,79	17	
1	1416225_at	BC013477	Adh1	alcohol dehydrogenase 1 (class I)	-0,30	3,32	3	138 215 031
1	1424045_at	BC019420	5730437N04Rik	RIKEN cDNA 5730437N04 gene	1,80	2,89	17	5 367 351
1	1433855_at	BF462185	Abat	4-aminobutyrate aminotransferase	-0,83	2,84	16	8 428 809
1	1423136_at	AI649186	Fgf1	fibroblast growth factor 1	-0,69	3,68	18	38 965 153
1	1433674_a_at	BQ177137	Snord22	small nucleolar RNA, C/D box 22	0,86	3,42	19	
1	1448813_at	NM_023383	Aadac	arylacetamide deacetylase (esterase)	-0,26	2,78	3	60 119 716
1	1420745_a_at	NM_010761	Ccndbp1	cyclin D-type binding-protein 1	0,61	2,63	2	120 699 848
1	1420410_at	NM_030676	Nr5a2	nuclear receptor subfamily 5, group A, member 2	0,19	2,90	1	138 660 686
1	1455657_at	BM219553	2610207I05Rik	RIKEN cDNA 2610207I05 gene	1,15	3,17	7	117 922 460
1	1429040_at	BE692399	2610005L07Rik	RIKEN cDNA 2610005L07 gene	1,24	3,25	8	20 272 528
1	1438528_at	AW545979	Pcm1	pericentriolar material 1	1,03	2,90	8	42 738 591
1	1438222_at	BB079962	Prpf40a	PRP40 pre-mRNA processing factor 40 homolog A (yeast)	1,95	2,95	2	52 960 486
1	1424318_at	BC019131	1110067D22Rik	RIKEN cDNA 1110067D22 gene	0,01	2,59	11	20 723 582
1	1450128_at	NM_011108	Pla2g2a	phospholipase A2, group IIA (platelets, synovial fluid)	-0,47	2,65	4	
1	1451839_a_at	AY007702	Pde7a	phosphodiesterase 7A	0,27	3,29	3	19 418 068
1	1433507_a_at	BE553881	Hmgn2	high mobility group nucleosomal binding domain 2	1,76	2,93	4	133 236 816
1	1436370_at	AI893437	Gucy2c	guanylate cyclase 2c	1,25	2,69	6	136 661 480
1	1455793_at	BB766438	BC035537	cDNA sequence BC035537	1,62	4,17	8	46 837 898
1	1448162_at	BB250384	Vcam1	vascular cell adhesion molecule 1	-1,16	3,20	3	116 102 025
1	1448315_a_at	NM_133705	Pycr2	pyrroline-5-carboxylate reductase family, member 2	0,27	3,35	1	182 740 983
1	1450724_at	NM_053090	Drctnnb1a	down-regulated by Ctnnb1, a	1,58	2,76	5	23 471 094
1	1449195_s_at	BC019961	Cxcl16	chemokine (C-X-C motif) ligand 16	-0,05	3,23	11	70 270 428
1	1434998_at	AV222277	Iqgap1	IQ motif containing GTPase activating protein 1	0,24	2,88	7	80 586 297
1	1427898_at	BI738010	Rnf6	ring finger protein (C3H2C3 type) 6	0,77	2,60	5	146 513 437
1	1415897_a_at	BI150149	Mgst1	microsomal glutathione S-transferase 1	-0,41	2,58	6	138 104 732
1	1427437_at	BC019134	2610203C20Rik	RIKEN cDNA 2610203C20 gene	1,05	3,01	9	41 331 809
1	1416503_at	NM_016753	Lxn	latexin	-0,79	3,04	3	67 545 927
1	1448939_at	NM_013918	Usp25	ubiquitin specific peptidase 25	1,93	2,63	16	76 896 660
1	1431053_at	AI536236	Mphosph9	M-phase phosphoprotein 9	1,18	3,71	5	
1	1455595_at	AW111403	Ugt2b36	UDP glucuronosyltransferase 2 family, polypeptide B36	0,34	3,45	5	88 140 487
1	1451751_at	AF335325	Ddit4l	DNA-damage-inducible transcript 4-like	-0,91	2,90	3	137 561 122
1	1443491_at	BG802688	Ptprk	protein tyrosine phosphatase, receptor type, K	1,14	2,88	10	27 764 235
1	1426174_s_at	S69212	Ighg	Immunoglobulin heavy chain (gamma polypeptide)	-0,01	2,58	12	113 755 089
1	1436591_at	BE553782	BC023744	cDNA sequence BC023744	0,76	2,69	5	117 580 347
1	1459647_at	BF148993	Pold3	polymerase (DNA-directed), delta 3, accessory subunit	0,67	4,05	7	99 956 195
1	1430555_s_at	BB524113	Lrig3	leucine-rich repeats and immunoglobulin-like domains 3	1,01	2,65	10	125 369 224
1	1451784_x_at	L36068	H2D1	histocompatibility 2, D region locus 1	1,76	2,80	17	
1	1426899_at	AV209678	Tbc1d23	TBC1 domain family, member 23	1,29	3,47	16	57 090 220
1	1431677_at	BC018486	4631424J17Rik	RIKEN cDNA 4631424J17 gene	0,01	2,61	11	43 552 883
1	1449079_s_at	NM_018784	St3gal6	ST3 beta-galactoside alpha-2,3-sialyltransferase 6	-0,17	2,81	16	58 413 086
1	1447483_s_at	AV011566	2610002F03Rik	RIKEN cDNA 2610002F03 gene	1,33	2,73	2	
1	1449076_x_at	NM_134052	Adi1	acireductone dioxygenase 1	1,92	2,78	12	29 261 474
1	1448786_at	NM_025806	1100001H23Rik	RIKEN cDNA 1100001H23 gene	0,65	3,36	6	136 576 266
1	1449078_at	NM_018784	St3gal6	ST3 beta-galactoside alpha-2,3-sialyltransferase 6	-1,08	2,58	16	58 413 086
1	1437621_x_at	AV216768	Phgdh	3-phosphoglycerate dehydrogenase	0,06	2,59	3	98 398 575
1	1457259_at	AV023631			1,43	3,88	12	55 624 711

1	1456584_x_at	BB495884	Phgdh	3-phosphoglycerate dehydrogenase	-0,49	2,83	3	98 398 575
1	1425278_at	BC021406	Ube4a	ubiquitination factor E4A, UFD2 homolog (S. cerevisiae)	1,62	2,76	9	44 684 066
1	1439521_at	AV011805			-0,55	2,91	?	
1	1421116_a_at	NM_024226	Rtn4	reticulon 4	-0,35	2,58	11	29 618 569
1	1428973_s_at	AK007178	0610012D17Rik	RIKEN cDNA 0610012D17 gene	1,20	3,29	16	32 339 490
1	1419356_at	BB524597	Klf7	Kruppel-like factor 7 (ubiquitous)	1,62	2,61	1	63 969 951
1	1418702_a_at	NM_025577	2810428I15Rik	RIKEN cDNA 2810428I15 gene	1,69	2,96	8	73 433 284
1	1436479_a_at	BB746075	Dpp7	dipeptidylpeptidase 7	0,84	3,21	2	25 174 298
1	1415899_at	NM_008416	Junb	Jun-B oncogene	-1,15	2,84	8	87 867 037
1	1434354_at	BB549292	Maob	monoamine oxidase B	0,44	2,91	X	15 866 242
1	1426074_at	BC006770	LOC434536		1,01	3,24	X	51 703 488
1	1417690_at	NM_016781	Prkag1	protein kinase, AMP-activated, gamma 1 non-catalytic subunit	1,07	2,66	15	98 640 831
1	1450276_a_at	NM_009132	Scin	scinderin	0,71	2,58	12	40 570 195
1	1455015_at	BE853276	Tbc1d9	TBC1 domain family, member 9	0,04	2,67	8	86 055 456
1	1418718_at	BC019961	Cxcl16	chemokine (C-X-C motif) ligand 16	-0,29	2,78	11	70 270 428
1	1427161_at	BE848253	Cenpf	centromere protein F	1,44	2,78	1	
1	1451479_a_at	BC019937	Tmem53	transmembrane protein 53	1,73	2,62	4	116 749 920
1	1425233_at	BC019553	2210407C18Rik	RIKEN cDNA 2210407C18 gene	-0,52	2,91	11	58 424 399
1	1449316_at	NM_134127	Cyp4f15	cytochrome P450, family 4, subfamily f, polypeptide 15	-2,02	2,71	17	32 424 968
1	1441980_at	BB355593	C030007I09Rik	RIKEN cDNA C030007I09 gene	0,47	2,70	3	132 776 760
1	1456014_s_at	BB113173	BC032204	cDNA sequence BC032204	0,61	2,60	19	7 066 021
1	1428760_at	AW537061	Snapc3	small nuclear RNA activating complex, polypeptide 3	0,17	2,61	4	82 888 979
1	1448547_at	BB703307	Rassf3	Ras association (RalGDS/AF-6) domain family 3	1,31	2,91	10	120 813 412
1	1420727_a_at	AY033513	Tmlhe	trimethyllysine hydroxylase, epsilon	0,27	3,07	X	
1	1434942_at	BM248080	2610101J03Rik	RIKEN cDNA 2610101J03 gene	1,28	2,61	2	
1	1455869_at	BG862223	Camk2b	calcium/calmodulin-dependent protein kinase II, beta	1,28	2,62	11	5 869 674
1	1432757_at	AK019313	2900011L18Rik	RIKEN cDNA 2900011L18 gene	1,67	3,17	13	
1	1445717_at	BB283604	Luc7l2	LUC7-like 2 (S. cerevisiae)	1,21	2,71	6	38 481 126
1	1430554_at	BB524113	Lrig3	leucine-rich repeats and immunoglobulin-like domains 3	0,94	3,00	10	125 369 224
1	1424713_at	AY061807	Calml4	calmodulin-like 4	1,82	2,70	9	62 656 109
1	1458282_at	BB454099	Cdc27	cell division cycle 27 homolog (S. cerevisiae)	1,26	2,68	11	?
1	1452592_at	AV066880	Mgst2	microsomal glutathione S-transferase 2	0,30	2,66	3	51 749 121
1	1434197_at	AW558010	Atrn	attractin	0,47	2,67	2	130 597 936
1	1449949_a_at	U90715	Cxadr	coxsackievirus and adenovirus receptor	-0,49	2,62	16	78 184 282
1	1447612_x_at	BB494168	Jmjd3	jumonji domain containing 3	0,89	2,65	11	69 214 714
1	1418282_x_at	NM_009244	Serpina1b	serine (or cysteine) preptidase inhibitor, clade A, member 1b	0,44	2,95	12	104 129 207
1	1425845_a_at	BC026364	Shoc2	soc-2 (suppressor of clear) homolog (C. elegans)	1,71	2,85	19	53 998 191
1	1456143_at	BM203042	Prkcbp1	protein kinase C binding protein 1	0,11	2,63	2	165 475 359
1	1420620_a_at	NM_011883	Rnf13	ring finger protein 13	-0,35	2,65	3	57 823 994
1	1444328_at	AI593816	Clta	clathrin, light polypeptide (Lca)	1,31	2,72	4	44 033 783
1	1448449_at	NM_019955	Ripk3	receptor-interacting serine-threonine kinase 3	-0,13	2,63	14	54 739 067
1	1443619_at	BB745549	Tmem30a	transmembrane protein 30A	1,90	2,73	9	79 554 632
1	1453795_at	BI872590	Fahd2a	fumarylacetoacetate hydrolase domain containing 2A	1,44	2,70	2	127 127 656
2	1436194_at	BE985366	C330008K14Rik	RIKEN cDNA C330008K14 gene	8,90	1,89	18	
2	1451731_at	AK007703	Abca3	ATP-binding cassette, sub-family A (ABC1), member 3	8,06	1,96	17	24 079 645
2	1448300_at	NM_025569	Mgst3	microsomal glutathione S-transferase 3	7,66	0,86	1	169 209 058
2	1430021_a_at	AK011772	Uble1a	ubiquitin-like 1 (sentrin) activating enzyme E1A	8,03	1,63	7	15 485 575
2	1419017_at	NM_016869	Corin	corin	7,71	0,08	5	72 579 160
2	1452716_at	AK017688	5730469M10Rik	RIKEN cDNA 5730469M10 gene	7,51	1,08	14	39 902 452
2	1425150_at	BC010829	C730036D15Rik	RIKEN cDNA C730036D15 gene	7,02	-1,02	4	49 400 944
2	1418989_at	NM_007799	Ctse	cathepsin E	6,07	-0,61	1	133 465 859
2	1449525_at	NM_008030	Fmo3	flavin containing monooxygenase 3	6,13	-0,21	1	164 790 474
2	1428004_at	BC028766	3300001G02Rik	RIKEN cDNA 3300001G02 gene	7,45	0,89	11	32 105 414
2	1448506_at	NM_007618	Serpina6	serine (or cysteine) peptidase inhibitor, clade A, member 6	6,97	-0,09	12	104 047 682
2	1418209_a_at	NM_019410	Pfn2	profilin 2	5,73	0,11	3	57 929 824
2	1447774_x_at	AV332575	5730469M10Rik	RIKEN cDNA 5730469M10 gene	6,39	1,03	14	39 902 452
2	1423867_at	BF234005	Serpina3c	serine (or cysteine) peptidase inhibitor, clade A, member 3C	6,78	-0,11	12	104 547 956
2	1418580_at	BC024872	Rtp4	receptor transporter protein 4	6,75	1,43	16	23 525 275
2	1446675_at	BB667305	Adk	adenosine kinase	6,81	1,00	14	19 841 184
2	1420928_at	BG075800	St6gal1	beta galactoside alpha 2,6 sialyltransferase 1	5,30	1,01	16	23 140 095
2	1419747_at	NM_007493	Asgr2	asialoglycoprotein receptor 2	5,63	0,38	11	69 908 838

2	1438676_at	BM241485	Mpa2l	macrophage activation 2 like	5,59	1,62	5	105 455 208
2	1451346_at	BG075139	Mtap	methylthioadenosine phosphorylase	4,76	-0,14	4	88 608 656
2	1457263_at	BI329779	Serpina3k	serine (or cysteine) peptidase inhibitor, clade A, member 3K	6,51	-0,16	12	104 739 557
2	1458302_at	BE200453			5,11	1,62	2	130 944 121
2	1456609_at	BE994488	Camk2n1	calcium/calmodulin-dependent protein kinase II inhibitor 1	5,50	0,84	4	
2	1423266_at	AI836168	2810405K02Rik	RIKEN cDNA 2810405K02 gene	5,67	0,72	4	153 740 229
2	1431566_at	AW319512	9030622O22Rik	RIKEN cDNA 9030622O22 gene	5,65	-0,23	2	
2	1417828_at	NM_007474	Aqp8	aquaporin 8	5,44	-0,20	7	123 253 459
2	1439622_at	AV291679	Rassf4	Ras association (RalGDS/AF-6) domain family 4	4,55	0,53	6	116 598 626
2	1424425_a_at	BG075139	Mtap	methylthioadenosine phosphorylase	5,69	-0,96	4	88 608 656
2	1446085_at	BB022048	Socs2	suppressor of cytokine signaling 2	4,44	0,95	10	94 841 729
2	1435887_at	BB806208	Serpina11	serine (or cysteine) peptidase inhibitor, clade A (alpha-1 antiproteinase, antitrypsin), member 11	5,53	-0,02	12	104 381 304
2	1448898_at	AF128196	Ccl9	chemokine (C-C motif) ligand 9	5,52	-1,99	11	83 389 111
2	1423555_a_at	BB329808	Ifi44	interferon-induced protein 44	4,88	0,46	3	151 668 310
2	1424929_a_at	AF230395	Trim26	tripartite motif protein 26	4,32	1,71	17	36 445 236
2	1420838_at	AK018789	Ntrk2	neurotrophic tyrosine kinase, receptor, type 2	5,45	-0,89	13	58 817 455
2	1424138_at	BC027346	Rhbdf1	rhomboid family 1 (Drosophila)	5,55	0,10	11	32 109 585
2	1416443_a_at	NM_019748	Uble1a	ubiquitin-like 1 (sentrin) activating enzyme E1A	5,22	0,99	7	15 485 575
2	1421363_at	NM_010003	Cyp2c39	cytochrome P450, family 2, subfamily c, polypeptide 39	4,05	-0,74	19	39 564 181
2	1423680_at	BC026831	Fads1	fatty acid desaturase 1	4,14	-0,09	19	10 250 017
2	1449315_at	NM_011857	Odz3	odd Oz/ten-m homolog 3 (Drosophila)	4,16	-0,05	8	49 726 507
2	1417651_at	NM_007815	Cyp2c29	cytochrome P450, family 2, subfamily c, polypeptide 29	4,84	-1,34	19	39 340 413
2	1457027_at	BB667395	Dhtkd1	dehydrogenase E1 and transketolase domain containing 1	5,15	0,16	2	
2	1420342_at	NM_010268	Gdap10	ganglioside-induced differentiation-associated-protein 10	4,27	1,44	12	33 407 119
2	1454799_at	AV300264	A230097K15Rik	RIKEN cDNA A230097K15 gene	4,60	0,27	5	101 086 665
2	1452375_at	BB282093	Aldh4a1	aldehyde dehydrogenase 4 family, member A1	5,29	0,60	4	138 895 085
2	1435417_at	BG063189	AI464131	expressed sequence AI464131	4,49	0,11	4	
2	1438649_x_at	AV207950	Pebp1	phosphatidylethanolamine binding protein 1	4,55	1,76	5	117 543 681
2	1435196_at	BB795585	Ntrk2	neurotrophic tyrosine kinase, receptor, type 2	4,53	0,58	13	58 817 455
2	1448253_at	NM_008133	Glud1	glutamate dehydrogenase 1	3,93	0,26	14	33 139 842
2	1419582_at	NM_028089	Cyp2c55	cytochrome P450, family 2, subfamily c, polypeptide 55	4,43	-0,42	19	39 072 062
2	1418191_at	NM_011909	Usp18	ubiquitin specific peptidase 18	4,74	0,68	6	121 211 575
2	1428592_s_at	BG064874	Usp38	ubiquitin specific peptidase 38	3,85	0,22	8	83 876 479
2	1443458_at	BF233964	D630033O11Rik	RIKEN cDNA D630033O11 gene	4,84	0,89	9	
2	1429410_at	AI595744	Eny2	enhancer of yellow 2 homolog (Drosophila)	3,62	1,55	15	44 258 186
2	1425194_a_at	BC024403	6330577E15Rik	RIKEN cDNA 6330577E15 gene	3,61	0,70	19	47 785 069
2	1436897_at	BE225764	Mfhas1	malignant fibrous histiocytoma amplified sequence 1	4,01	1,57	8	37 059 314
2	1444009_at	AV217868	Rassf4	Ras association (RalGDS/AF-6) domain family 4	4,20	0,63	6	116 598 626
2	1419059_at	NM_011318	Apcs	serum amyloid P-component	3,99	1,76	1	174 730 636
2	1455265_a_at	BB100249	Rgs16	regulator of G-protein signaling 16	3,94	-0,66	1	155 502 590
2	1415802_at	NM_009196	Slc16a1	solute carrier family 16 (monocarboxylic acid transporters), member 1	3,30	-1,37	3	104 767 728
2	1425109_at	BC025548	Slc44a3	solute carrier family 44, member 3	3,55	-1,34	3	121 451 550
2	1455741_a_at	AW553715	Ece1	endothelin converting enzyme 1	4,07	1,71	4	137 134 372
2	1431038_at	BF137610	Rassf4	Ras association (RalGDS/AF-6) domain family 4	4,63	0,17	6	116 598 626
2	1443620_at	BB212497	Gpc4	glypican 4	3,77	1,01	X	48 297 548
2	1451564_at	BC021340	Parp14	poly (ADP-ribose) polymerase family, member 14	4,00	0,93	16	35 752 626
2	1429239_a_at	AK014587	Stard4	StAR-related lipid transfer (START) domain containing 4	3,98	1,82	18	33 326 231
2	1425364_a_at	U25708	Slc3a2	solute carrier family 3 (activators of dibasic and neutral amino acid transport), member 2	3,46	-0,04	19	8 773 926
2	1448566_at	AF226613	Slc40a1	solute carrier family 40 (iron-regulated transporter), member 1	3,94	0,17	1	45 852 629
2	1435787_at	BB035578	Ppm1l	protein phosphatase 1 (formerly 2C)-like	4,01	0,56	3	69 404 846
2	1428484_at	AK004768	Osbpl3	oxysterol binding protein-like 3	4,65	0,57	6	50 226 392
2	1449813_at	NM_013705	Zfp30	zinc finger protein 30	4,82	1,31	7	29 493 549
2	1428547_at	AV273591	Nt5e	5' nucleotidase, ecto	3,62	1,08	9	88 125 532
2	1429514_at	AW111876	Ppap2b	phosphatidic acid phosphatase type 2B	3,15	0,68	4	104 655 278
2	1456074_at	BB143568	Sdro	orphan short chain dehydrogenase/reductase	4,55	0,41	10	127 301 483
2	1427027_a_at	AV173739	Gtf3a	general transcription factor III A	3,38	1,31	5	147 259 059
2	1455918_at	BB224790	Adrb3	adrenergic receptor, beta 3	4,70	0,28	8	28 691 711
2	1434380_at	BM241271	Gbp7	guanylate binding protein 7	4,08	1,24	3	142 467 727
2	1450696_at	NM_013585	Psmb9	proteosome (prosome, macropain) subunit, beta type 9 (large multifunctional peptidase 2)	3,24	1,66	17	33 792 386
2	1418507_s_at	NM_007706	Socs2	suppressor of cytokine signaling 2	3,48	-0,14	10	94 841 729
2	1422147_a_at	BC003487	Pla2g6	phospholipase A2, group VI	3,95	0,75	15	79 113 484

2	1418210_at	NM_019410	Pfn2	profilin 2	4,40	0,39	3	57 929 824
2	1419100_at	NM_009252	Serpina3n	serine (or cysteine) peptidase inhibitor, clade A, member 3N	3,98	-0,14	12	104 807 808
2	1427934_at	AA250510	2610208E05Rik	RIKEN cDNA 2610208E05 gene	4,11	0,84	4	33 128 877
2	1424383_at	BC003277	Tmem51	transmembrane protein 51	4,38	-0,41	4	141 303 070
2	1452339_at	AL359935	Adamts7	a disintegrin-like and metallopeptidase (reprolysin type) with thrombospondin type 1 motif, 7	3,84	0,07	9	89 961 137
2	1436643_x_at	AV051678	Hamp2	hepcidin antimicrobial peptide 2	3,36	-1,19	7	30 631 156
2	1427935_at	AA250510	2610208E05Rik	RIKEN cDNA 2610208E05 gene	4,35	1,03	4	33 128 877
2	1418763_at	BC020153	Nit2	nitrilase family, member 2	3,95	0,32	16	57 078 024
2	1417432_a_at	NM_008142	Gnb1	guanine nucleotide binding protein, beta 1	3,21	0,45	4	154 371 249
2	1417141_at	NM_018738	Igtp	interferon gamma induced GTPase	3,38	1,40	11	58 015 750
2	1424279_at	BC005467	Fga	fibrinogen, alpha polypeptide	3,02	0,10	3	83 112 080
2	1447927_at	BG092512	Mpa2l	macrophage activation 2 like	4,31	1,36	5	105 455 208
2	1427776_a_at	AF127141	Fgfr4	fibroblast growth factor receptor 4	3,08	0,25	13	55 162 439
2	1434465_x_at	AV333363	Vldlr	very low density lipoprotein receptor	3,95	-0,50	19	27 284 528
2	1420927_at	BG075800	St6gal1	beta galactoside alpha 2,6 sialyltransferase 1	3,84	1,03	16	23 140 095
2	1440327_at	AA985897	Cyp2c70	cytochrome P450, family 2, subfamily c, polypeptide 70	4,17	0,34	19	40 206 672
2	1417667_a_at	NM_008961	Pter	phosphotriesterase related	4,55	1,37	2	12 841 893
2	1431867_a_at	AK005692	1700007B13Rik	RIKEN cDNA 1700007B13 gene	2,82	-0,14	7	119 277 736
2	1460684_at	BC014769	Tm7sf2	transmembrane 7 superfamily member 2	3,53	0,39	19	6 062 821
2	1460197_a_at	NM_054098	Steap4	STEAP family member 4	3,83	0,17	5	7 966 477
2	1434482_at	BM203518	D4Ertd22e	DNA segment, Chr 4, ERATO Doi 22, expressed	3,42	0,66	4	140 385 091
2	1417793_at	NM_019440	Iigp2	interferon inducible GTPase 2	3,71	1,57	11	58 015 831
2	1416209_at	NM_008133	Glud1	glutamate dehydrogenase 1	3,26	0,47	14	33 139 842
2	1416222_at	BC019945	Nsdhl	NAD(P) dependent steroid dehydrogenase-like	3,74	1,75	X	69 171 240
2	1434211_at	BB352548	Sh3bgrl2	SH3 domain binding glutamic acid-rich protein like 2	2,86	-0,03	9	83 345 047
2	1454730_at	AV273867	4932414K18Rik	RIKEN cDNA 4932414K18 gene	3,27	0,63	5	44 463 411
2	1442005_at	BB018051	AW987390	expressed sequence AW987390	4,40	0,49	4	
2	1437085_at	AV370040	D630039A03Rik	RIKEN cDNA D630039A03 gene	3,57	-0,28	4	58 002 438
2	1428793_at	AK017918	Slc36a1	solute carrier family 36 (proton/amino acid symporter), member 1	3,79	-0,12	11	55 047 792
2	1455131_at	BB475273	Opa3	optic atrophy 3 (human)	3,01	0,91	7	18 386 910
2	1429240_at	AK014587	Stard4	StAR-related lipid transfer (START) domain containing 4	3,40	1,11	18	33 326 231
2	1417038_at	NM_017380	Sept9	septin 9	3,04	0,30	11	117 147 809
2	1451452_a_at	U72881	Rgs16	regulator of G-protein signaling 16	4,02	0,15	1	155 502 590
2	1425632_a_at	BC019216	Pqlc2	PQ loop repeat containing 2	4,28	-1,07	4	138 570 606
2	1438617_at	BB222737	Serpina7	serine (or cysteine) peptidase inhibitor, clade A (alpha-1 antiproteinase, antitrypsin), member 7	3,69	-0,24	X	134 426 521
2	1443049_at	BB199824	Tmem19	transmembrane protein 19	3,47	1,52	10	114 744 850
2	1456388_at	AV378604	Atp11a	ATPase, class VI, type 11A	3,01	-0,26	8	12 757 200
2	1418260_at	NM_015755	Hunk	hormonally upregulated Neu-associated kinase	3,94	0,20	16	90 275 255
2	1422001_at	U95962	Inhbc	inhibin beta-C	3,76	0,60	10	126 759 270
2	1427561_a_at	BC026681	Afm	afamin	2,91	0,35	5	91 594 162
2	1427442_a_at	BC005490	App	amyloid beta (A4) precursor protein	3,33	-1,23	16	84 837 873
2	1429178_at	BB472509	Odz3	odd Oz/ten-m homolog 3 (Drosophila)	2,96	0,15	8	49 726 507
2	1446412_at	AW538350	Wwox	WW domain-containing oxidoreductase	3,19	1,92	8	117 325 672
2	1451345_at	BG075139	Mtap	methylthioadenosine phosphorylase	3,93	-0,76	4	88 608 656
2	1429947_a_at	AK008179	Zbp1	Z-DNA binding protein 1	3,71	0,68	2	172 849 553
2	1435690_at	BB476775	2310008H09Rik	RIKEN cDNA 2310008H09 gene	3,20	0,92	7	118 636 911
2	1424426_at	BG075139	Mtap	methylthioadenosine phosphorylase	4,16	0,03	4	88 608 656
2	1437894_at	BE994433	Prox1	prospero-related homeobox 1	3,72	0,38	1	191 822 565
2	1448406_at	BC010712	Cri1	CREBBP/EP300 inhibitory protein 1	3,94	0,85	2	125 364 535
2	1419042_at	BM239828	Iigp1	interferon inducible GTPase 1	3,48	1,04	18	60 515 163
2	1417823_at	AK013138	Gcat	glycine C-acetyltransferase (2-amino-3-ketobutyrate-coenzyme A ligase)	3,43	1,01	15	78 858 174
2	1416368_at	NM_010357	Gsta4	glutathione S-transferase, alpha 4	4,35	-0,35	9	77 977 715
2	1450034_at	AW214029	Stat1	signal transducer and activator of transcription 1	3,86	0,84	1	52 064 001
2	1426690_a_at	AI326423	Srebf1	sterol regulatory element binding factor 1	2,86	0,50	11	60 015 283
2	1423256_a_at	BI154058	Atp6v1g1	ATPase, H+ transporting, lysosomal V1 subunit G1	2,95	0,91	4	63 031 197
2	1422557_s_at	NM_013602	Mt1	metallothionein 1	3,40	0,15	8	97 068 355
2	1424700_at	BC011072	Tmem38b	transmembrane protein 38B	3,17	-0,13	4	53 847 161
2	1419510_at	NM_133660	Es22	esterase 22	3,84	1,58	8	96 090 346
2	1423465_at	BB032852	Frrs1	ferric-chelate reductase 1	3,17	0,54	3	116 853 695
2	1438654_x_at	AV269411	Mmd2	monocyte to macrophage differentiation-associated 2	3,51	-0,23	5	142 814 838
2	1416352_s_at	NM_134118	Gpsn2	glycoprotein, synaptic 2	3,34	0,32	8	86 461 804

2	1419043_a_at	BM239828	Iigp1	interferon inducible GTPase 1	3,21	1,13	18	60 515 163
2	1455500_at	AW556558	D11Ertd759e	DNA segment, Chr 11, ERATO Doi 759, expressed	3,86	0,50	11	119 296 890
2	1450783_at	NM_008331	Ifit1	interferon-induced protein with tetratricopeptide repeats 1	3,67	1,80	19	34 706 885
2	1423624_at	BB751093	Fancl	Fanconi anemia, complementation group L	3,57	1,38	11	26 287 083
2	1435881_at	AW556977	Map3k12	mitogen activated protein kinase kinase kinase 12	2,72	1,15	15	102 325 682
2	1460258_at	NM_010701	Lect1	leukocyte cell derived chemotaxin 1	3,60	-0,09	14	78 371 848
2	1451103_at	BC011095	D14Ertd500e	DNA segment, Chr 14, ERATO Doi 500, expressed	3,60	-0,78	14	53 495 990
2	1417264_at	AK014348	Coq5	coenzyme Q5 homolog, methyltransferase (yeast)	3,96	1,64	5	115 540 700
2	1448908_at	NM_080555	Ppap2b	phosphatidic acid phosphatase type 2B	3,56	0,56	4	104 655 278
2	1423216_a_at	AV109006	2510049I19Rik	RIKEN cDNA 2510049I19 gene	3,10	1,99	8	75 148 718
2	1450226_at	NM_008932	Prlr	prolactin receptor	3,55	-0,13	15	10 122 379
2	1420919_at	BB768208	Sgk3	serum/glucocorticoid regulated kinase 3	3,58	-1,07	1	9 833 494
2	1434109_at	AV291265	Sh3bgrl2	SH3 domain binding glutamic acid-rich protein like 2	3,17	0,14	9	83 345 047
2	1422077_at	AA571017	Acot4	acyl-CoA thioesterase 4	3,05	-0,79	12	84 928 126
2	1434442_at	BB667844	D5Ertd593e	DNA segment, Chr 5, ERATO Doi 593, expressed	3,72	0,12	5	93 678 254
2	1450611_at	NM_013623	Orm3	orosomucoid 3	3,35	-0,48	4	62 842 522
2	1455162_at	BI147002	4922503N01Rik	RIKEN cDNA 4922503N01 gene	3,20	-0,47	4	108 905 054
2	1417538_at	NM_011895	Slc35a1	solute carrier family 35 (CMP-sialic acid transporter), member 1	3,27	0,14	4	34 852 144
2	1425156_at	BC010229	Gbp7	guanylate binding protein 7	3,65	1,24	3	142 467 727
2	1419595_a_at	NM_010281	Ggh	gamma-glutamyl hydrolase	3,11	-0,69	4	20 164 802
2	1434580_at	AV280361	Enpp4	ectonucleotide pyrophosphatase/phosphodiesterase 4	3,54	0,78	17	43 559 366
2	1418782_at	NM_009107	Rxrg	retinoid X receptor gamma	3,13	-0,61	1	169 435 058
2	1447845_s_at	AV360029	Vnn1	vanin 1	2,69	-0,05	10	23 584 275
2	1418736_at	BC003835	B3galnt1	UDP-GalNAc:betaGlcNAc beta 1,3-galactosaminyltransferase, polypeptide 1	3,12	-1,48	3	69 662 114
2	1426905_a_at	AV114239	Dnajc10	DnaJ (Hsp40) homolog, subfamily C, member 10	2,90	0,09	2	80 116 627
2	1424518_at	BC020489	BC020489	cDNA sequence BC020489	3,11	0,36	15	
2	1442916_at	BB202300	Psd3	pleckstrin and Sec7 domain containing 3	2,67	-0,44	8	70 625 871
2	1424617_at	BC008158	Ifi35	interferon-induced protein 35	3,41	1,35	11	101 264 501
2	1420603_s_at	NM_009016	Raet1a	retinoic acid early transcript 1, alpha	2,99	0,48	10	21 848 024
2	1451505_at	BE991735	Chchd5	coiled-coil-helix-coiled-coil-helix domain containing 5	3,06	1,46	2	128 821 262
2	1455270_at	AV222311	Adam11	a disintegrin and metallopeptidase domain 11	3,40	0,14	11	102 577 695
2	1452501_at	AF047725	Cyp2c38	cytochrome P450, family 2, subfamily c, polypeptide 38	3,07	0,29	19	39 442 866
2	1445897_s_at	AW986054	Ifi35	interferon-induced protein 35	3,51	1,20	11	101 264 501
2	1450063_at	BM228488	Fmn2	formin 2	3,69	-0,06	1	176 338 520
2	1427896_at	AK010274	2400003N08Rik	RIKEN cDNA 2400003N08 gene	3,36	-0,45	5	117 352 714
2	1424210_at	BC011220	Spfh1	SPFH domain family, member 1	3,14	0,65	19	44 088 258
2	1423035_s_at	AK004219	Txnl5	thioredoxin-like 5	2,85	-0,03	11	72 023 748
2	1449025_at	NM_010501	Ifit3	interferon-induced protein with tetratricopeptide repeats 3	3,27	1,66	19	34 649 550
2	1423034_at	AK004219	Txnl5	thioredoxin-like 5	2,91	-0,15	11	72 023 748
2	1424794_at	BC011492	Rnf186	ring finger protein 186	3,11	1,99	4	138 239 194
2	1448275_at	AK018383	Tmem19	transmembrane protein 19	2,95	0,27	10	114 744 850
2	1452309_at	BB409331	Cgnl1	cingulin-like 1	3,46	-0,40	9	71 425 068
2	1437983_at	BB739342	Sall1	sal-like 1 (Drosophila)	3,16	-0,11	8	91 918 348
2	1451229_at	BC016208	Hdac11	histone deacetylase 11	3,34	1,03	6	91 122 318
2	1422974_at	NM_011851	Nt5e	5' nucleotidase, ecto	3,50	0,85	9	88 125 532
2	1416566_at	NM_011499	Strap	serine/threonine kinase receptor associated protein	2,61	0,33	6	137 699 321
2	1450715_at	NM_009993	Cyp1a2	cytochrome P450, family 1, subfamily a, polypeptide 2	3,49	0,48	9	57 475 073
2	1424382_at	BC025602	Rcn3	reticulocalbin 3, EF-hand calcium binding domain	2,78	0,76	7	44 950 954
2	1420915_at	AW214029	Stat1	signal transducer and activator of transcription 1	3,26	1,09	1	52 064 001
2	1437654_at	BE630700	3110001K24Rik	RIKEN cDNA 3110001K24 gene	2,98	0,12	14	33 066 132
2	1422603_at	BC005569	Rnase4	ribonuclease, RNase A family 4	2,66	0,85	14	50 013 146
2	1423992_at	BC019178	Gatad2a	GATA zinc finger domain containing 2A	3,07	-0,72	8	72 837 458
2	1429089_s_at	BG063749	2900026A02Rik	RIKEN cDNA 2900026A02 gene	3,10	-0,08	5	113 329 252
2	1417869_s_at	NM_022325	Ctsz	cathepsin Z	2,68	-0,47	2	174 070 433
2	1417539_at	NM_011895	Slc35a1	solute carrier family 35 (CMP-sialic acid transporter), member 1	3,16	0,23	4	34 852 144
2	1417280_at	NM_009198	Slc17a1	solute carrier family 17 (sodium phosphate), member 1	3,10	-0,54	13	23 877 796
2	1424692_at	BC004753	2810055F11Rik	RIKEN cDNA 2810055F11 gene	2,73	0,37	12	72 992 011
2	1426043_a_at	AF127766	Capn3	calpain 3	2,78	0,12	2	120 155 313
2	1416256_a_at	NM_011655	Tubb5	tubulin, beta 5	2,70	1,13	17	35 441 972
2	1451803_a_at	U48800	Vegfb	vascular endothelial growth factor B	3,16	-0,46	19	7 049 515
2	1457434_s_at	AV102876	Ptpla	protein tyrosine phosphatase-like (proline instead of catalytic arginine), member a	3,06	0,52	2	13 944 585

2	1444032_at	AI746421	Keg1	kidney expressed gene 1	2,75	-0,06	19	12 762 811
2	1453004_at	BM234253	3110004L20Rik	RIKEN cDNA 3110004L20 gene	2,92	-1,28	13	34 186 622
2	1424790_at	BC025937	2900084M01Rik	RIKEN cDNA 2900084M01 gene	3,26	1,47	8	73 113 328
2	1424652_at	BC027150	Tmem166	transmembrane protein 166	2,62	-0,80	6	82 007 330
2	1436344_at	BB005022	5830404H04Rik	RIKEN cDNA 5830404H04 gene	3,39	0,58	16	98 013 680
2	1419703_at	NM_016919	Col5a3	procollagen, type V, alpha 3	2,66	0,33	9	20 520 451
2	1426037_a_at	U94828	Rgs16	regulator of G-protein signaling 16	2,73	-0,71	1	155 502 590
2	1424946_a_at	AK009195	Mtfr1	mitochondrial fission regulator 1	2,59	1,16	3	19 380 394
2	1448276_at	NM_053082	Tspan4	tetraspanin 4	3,39	0,34	7	141 326 559
2	1438037_at	AW208668	Ids	iduronate 2-sulfatase	3,15	0,78	X	66 603 736
2	1460688_s_at	BB390422	AA407659	expressed sequence AA407659	3,07	0,77	5	115 383 564
2	1425799_at	AF461145	Fmo4	flavin containing monooxygenase 4	3,28	-2,23	1	164 630 557
2	1450726_at	NM_018830	Asah2	N-acylsphingosine amidohydrolase 2	2,87	-0,18	19	32 050 645
2	1424176_a_at	BE628614	Anxa4	annexin A4	2,90	-0,39	6	86 702 480
2	1427440_a_at	AJ011080	Afm	afamin	2,68	0,73	5	91 594 162
2	1416424_at	BC011116	M6prbp1	mannose-6-phosphate receptor binding protein 1	2,78	-0,17	17	55 912 670
2	1435349_at	BB752129	Nrp2	neuropilin 2	2,62	0,74	1	62 637 980
2	1431806_at	AK016444	4931408D14Rik	RIKEN cDNA 4931408D14 gene	3,04	0,00	19	
2	1436389_at	AW107484			2,58	1,07	19	7 606 114
2	1452298_a_at	AW546331	Myo5b	myosin Vb	2,78	-0,80	18	74 567 983
2	1430245_at	BM209124	Fxr1h	fragile X mental retardation gene 1, autosomal homolog	3,05	0,66	3	34 211 656
2	1448130_at	NM_010191	Fdft1	farnesyl diphosphate farnesyl transferase 1	2,83	0,62	14	62 099 262
2	1431591_s_at	AK019325	LOC677168		3,14	0,62	17	
2	1425410_at	BC027413	Tprkb	Tp53rk binding protein	3,21	1,68	6	85 881 390
2	1429485_a_at	AK008801	Utp11l	UTP11-like, U3 small nucleolar ribonucleoprotein, (yeast)	2,66	0,77	4	124 181 069
2	1426833_at	BG073769	Eif4g3	eukaryotic translation initiation factor 4 gamma, 3	2,66	0,78	4	137 265 545
2	1434271_at	BM124000	Gba2	glucosidase beta 2	2,60	0,12	4	43 588 036
2	1435981_at	BM118398	E430014L09Rik	RIKEN cDNA E430014L09 gene	3,16	0,11	7	49 477 471
2	1431648_at	AK015924	4930528F23Rik	RIKEN cDNA 4930528F23 gene	3,15	0,30	17	
2	1418028_at	NM_010024	Dct	dopachrome tautomerase	3,27	-0,05	14	116 895 152
2	1431095_a_at	AI639807	Herc5	hect domain and RLD 5	3,08	0,75	6	
2	1425321_a_at	AB059644	Clmn	calmin	2,68	1,74	12	105 164 164
2	1445597_s_at	BB404920	Hrasls3	HRAS like suppressor 3	3,16	-0,35	19	7 624 502
2	1430785_at	BB150587	Sdro	orphan short chain dehydrogenase/reductase	3,30	-0,10	10	127 301 483
2	1443889_at	AI789751	9030619P08Rik	RIKEN cDNA 9030619P08 gene	2,73	-0,66	15	75 254 861
2	1432195_s_at	AK008585	Ccnl2	cyclin L2	2,80	1,81	4	154 656 288
2	1452602_a_at	AI195035	1700001C19Rik	RIKEN cDNA 1700001C19 gene	2,89	1,18	17	46 875 939
2	1455079_at	BQ176904	Dcun1d4	DCN1, defective in cullin neddylation 1, domain containing 4 (S. cerevisiae)	3,09	-0,32	5	73 770 216
2	1428714_at	BF322962	Pgrmc2	progesterone receptor membrane component 2	3,02	0,30	3	41 162 877
2	1458007_at	BG066725	C80142	expressed sequence C80142	3,16	1,51	1	
2	1451376_at	BC017138	5730596K20Rik	RIKEN cDNA 5730596K20 gene	3,10	0,46	19	7 561 620
2	1448582_at	NM_025680	Ctnnbl1	catenin, beta like 1	2,93	0,67	2	157 428 902
2	1417420_at	NM_007631	Ccnd1	cyclin D1	2,98	0,22	7	144 739 320
2	1425712_at	BC025446	BC025446	cDNA sequence BC025446	3,08	-0,43	15	75 043 850
2	1449348_at	AF199010	Mpp6	membrane protein, palmitoylated 6 (MAGUK p55 subfamily member 6)	3,18	0,11	6	50 039 962
2	1429681_a_at	AK020708	Gpsn2	glycoprotein, synaptic 2	2,95	0,46	8	86 461 804
2	1451204_at	BC016096	Scara5	scavenger receptor class A, member 5 (putative)	2,78	-0,04	14	64 620 536
2	1443858_at	BI653857	Trim34	tripartite motif protein 34	2,68	1,38	7	104 118 384
2	1442873_at	BB479296			2,73	0,52	2	
2	1448673_at	NM_021495	Pvrl3	poliovirus receptor-related 3	3,04	-1,19	16	46 367 420
2	1457054_a_at	BE991737	1110034C04Rik	RIKEN cDNA 1110034C04 gene	2,94	1,85	12	101 440 569
2	1424296_at	BC019374	Gclc	glutamate-cysteine ligase, catalytic subunit	3,24	0,55	9	77 540 493
2	1460662_at	NM_011067	Per3	period homolog 3 (Drosophila)	3,22	1,92	4	149 848 511
2	1451197_s_at	BC019178	Gatad2a	GATA zinc finger domain containing 2A	3,00	-0,59	8	72 837 458
2	1425329_a_at	AF332060	Cyb5r3	cytochrome b5 reductase 3	3,11	1,00	15	82 981 269
2	1450033_a_at	AW214029	Stat1	signal transducer and activator of transcription 1	2,84	1,03	1	52 064 001
2	1449603_at	AA711038	AI594671	expressed sequence AI594671	2,95	1,65	11	39 879 648
2	1416589_at	NM_009242	Sparc	secreted acidic cysteine rich glycoprotein	3,05	0,41	11	55 237 923
2	1422666_at	NM_023224	Cblc	Casitas B-lineage lymphoma c	2,67	1,92	7	18 938 241
2	1436339_at	BE627374	1810058I24Rik	RIKEN cDNA 1810058I24 gene	2,60	0,60	6	
2	1456735_x_at	BB458645	Acpl2	acid phosphatase-like 2	2,65	0,63	9	96 632 695

2	1448555_at	NM_028003	D15Ertd682e	DNA segment, Chr 15, ERATO Doi 682, expressed	2,99	-0,16	15	97 503 138
2	1417292_at	NM_008330	Ifi47	interferon gamma inducible protein 47	2,99	1,30	11	48 930 477
2	1427356_at	BC023460	2310031A18Rik	RIKEN cDNA 2310031A18 gene	2,61	0,97	8	127 626 333
2	1435193_at	BB085904	A230050P20Rik	RIKEN cDNA A230050P20 gene	3,16	0,64	9	20 619 097
2	1424352_at	BC025936	Cyp4a12	cytochrome P450, family 4, subfamily a, polypeptide 12	2,81	0,40	4	114 796 977
2	1453304_s_at	BM245572	Ly6e	lymphocyte antigen 6 complex, locus E	2,94	0,92	15	74 782 907
2	1454757_s_at	AW554405	D12Ertd647e	DNA segment, Chr 12, ERATO Doi 647, expressed	2,78	1,34	12	103 835 260
2	1454149_a_at	AK008585	Ccnl2	cyclin L2	2,70	1,04	4	154 656 288
2	1425387_at	BC021607	Akr1c20	aldo-keto reductase family 1, member C20	2,88	-0,60	13	4 506 404
2	1418401_a_at	NM_130447	Dusp16	dual specificity phosphatase 16	2,79	0,36	6	134 681 162
2	1425514_at	M60651	Pik3r1	phosphatidylinositol 3-kinase, regulatory subunit, polypeptide 1 (p85 alpha)	2,87	-0,22	13	102 781 019
2	1424758_s_at	BC018416	Serpina10	serine (or cysteine) peptidase inhibitor, clade A (alpha-1 antiproteinase, antitrypsin), member 10	3,11	0,32	12	104 017 726
2	1459238_at	AV319481	Pln	phospholamban	2,67	-0,31	10	53 026 121
2	1421056_at	BC012671	Dnase1l3	deoxyribonuclease 1-like 3	3,09	1,07	14	6 757 069
2	1419559_at	BC011228	Cyp4f14	cytochrome P450, family 4, subfamily f, polypeptide 14	2,65	0,57	17	32 643 488
2	1453692_at	AK019824	4930581F22Rik	RIKEN cDNA 4930581F22 gene	2,99	-0,57	9	
2	1417244_a_at	NM_016850	Irf7	interferon regulatory factor 7	3,19	0,53	7	141 114 508
2	1418825_at	NM_008326	Irgm	immunity-related GTPase family, M	2,86	1,03	11	48 708 671
2	1460733_at	BB390422	AA407659	expressed sequence AA407659	2,76	0,26	5	115 383 564
2	1431302_a_at	AK011172	Nudt7	nudix (nucleoside diphosphate linked moiety X)-type motif 7	2,97	0,20	8	117 019 591
2	1457601_at	BB653614			2,67	0,05	16	
2	1425752_at	AJ132857	BC014805	cDNA sequence BC014805	2,77	-0,05	19	7 849 028
2	1449409_at	NM_026935	Sult1c2	sulfotransferase family, cytosolic, 1C, member 2	2,89	0,38	17	53 294 567
2	1442182_at	AV256803	Dnajc19	DnaJ (Hsp40) homolog, subfamily C, member 19	2,90	1,18	3	34 249 306
2	1449677_s_at	C77858	Tmem38b	transmembrane protein 38B	2,99	-0,78	4	53 847 161
2	1451490_at	BC027340	Lyplal1	lysophospholipase-like 1	2,71	1,28	1	187 788 673
2	1445144_at	BG072125	Sntb1	syntrophin, basic 1	2,68	-0,38	15	55 469 236
2	1443163_at	BB049001	Slc39a2	solute carrier family 39 (zinc transporter), member 2	2,62	0,10	14	50 815 590
2	1420621_a_at	NM_007471	App	amyloid beta (A4) precursor protein	2,83	-1,14	16	84 837 873
2	1418293_at	NM_008332	Ifit2	interferon-induced protein with tetratricopeptide repeats 2	2,75	1,79	19	34 616 942
2	1426670_at	BM208224	Agrin	agrin	2,80	1,35	4	155 009 093
2	1453300_at	AK017526	Slc35d2	solute carrier family 35, member D2	2,66	0,94	13	64 105 531
2	1456012_x_at	AV101824	Rnaset2	ribonuclease T2	2,59	0,17	17	6 828 517
2	1439109_at	AV378320	Ccdc68	coiled-coil domain containing 68	2,80	0,46	18	70 050 927
2	1436993_x_at	BB560492	Pfn2	profilin 2	2,77	0,95	3	57 929 824
2	1417900_a_at	NM_013703	Vldlr	very low density lipoprotein receptor	2,67	-0,63	19	27 284 528
2	1426419_at	AK005802	Rbm26	RNA binding motif protein 26	2,77	1,32	14	104 001 821
2	1425222_x_at	AB056443	AB056442	cDNA sequence AB056442	2,69	0,45	19	
2	1454086_a_at	AK013416	Lmo2	LIM domain only 2	2,88	1,03	2	103 771 061
2	1438724_at	AI875442	Osbpl3	oxysterol binding protein-like 3	3,02	0,30	6	50 226 392
2	1419134_at	NM_021375	Rhbg	Rhesus blood group-associated B glycoprotein	2,59	1,76	3	88 328 800
2	1460428_at	BC003286	Ankrd13a	ankyrin repeat domain 13a	2,86	0,97	5	115 036 138
2	1427878_at	AK002512	0610010O12Rik	RIKEN cDNA 0610010O12 gene	2,69	-1,10	18	
2	1448358_s_at	NM_026506	Snrpg	small nuclear ribonucleoprotein polypeptide G	2,61	1,17	6	86 337 173
2	1454033_at	AK005692	1700007B13Rik	RIKEN cDNA 1700007B13 gene	2,69	-0,24	7	119 277 736
2	1421704_a_at	NM_011084	Pik3c2g	phosphatidylinositol 3-kinase, C2 domain containing, gamma polypeptide	2,63	0,57	6	139 803 850
2	1423305_at	BB826950	Extl1	exostoses (multiple)-like 1	2,83	-0,03	4	133 628 449
2	1451668_at	BG060641	C530043G21Rik	RIKEN cDNA C530043G21 gene	2,63	-0,53	1	158 515 247
2	1445621_at	AI593401			2,58	0,51	2	
2	1428022_at	BC027556	Lcn13	lipocalin 13	2,65	-0,01	2	25 522 082
2	1417636_at	NM_008135	Slc6a9	solute carrier family 6 (neurotransmitter transporter, glycine), member 9	2,68	0,02	4	117 333 189
2	1458245_at	BM198146	A530080P10		2,59	0,20	4	
2	1416833_at	NM_029550	Keg1	kidney expressed gene 1	2,63	-0,20	19	12 762 811
2	1455673_at	BG070741	Alkbh2	alkB, alkylation repair homolog 2 (E. coli)	2,67	1,41	5	114 384 933
2	1427035_at	BB399837	Slc39a14	solute carrier family 39 (zinc transporter), member 14	2,59	-0,87	14	69 038 548
2	1437205_at	BE988778	Tcf25	transcription factor 25 (basic helix-loop-helix)	2,61	0,62	8	126 259 924
3	1452544_x_at	J00406	H2D1	histocompatibility 2, D region locus 1	10,54	6,80	17	
3	1425614_x_at	M83244	H2K1	histocompatibility 2, K1, K region	9,98	5,53	17	
3	1419327_at	NM_053181	2210010A19Rik	RIKEN cDNA 2210010A19 gene	9,96	9,18	16	13 747 507
3	1418701_at	NM_007744	Comt	catechol-O-methyltransferase	8,86	4,37	16	18 321 105
3	1427651_x_at	X00246	H2D1	histocompatibility 2, D region locus 1	9,42	6,01	17	

3	1443621_at	BG092359	1700010H23Rik	RIKEN cDNA 1700010H23 gene	8,29	3,78	11	?
3	1422892_s_at	U13648	H2Ea	histocompatibility 2, class II antigen E alpha	7,48	3,63	17	33 949 793
3	1426787_at	AW744519	Eef2	eukaryotic translation elongation factor 2	8,19	4,61	10	80 579 787
3	1429831_at	BI684288	Pik3ap1	phosphoinositide-3-kinase adaptor protein 1	6,74	3,33	19	41 325 683
3	1451593_at	BC018402	H2K1	histocompatibility 2, K1, K region	7,72	2,68	17	
3	1425521_at	BC019726	Paip1	polyadenylate binding protein-interacting protein 1	6,72	6,23	13	120 547 808
3	1416105_at	BC008518	Nnt	nicotinamide nucleotide transhydrogenase	6,56	6,09	13	120 453 189
3	1415950_a_at	NM_018858	Pebp1	phosphatidylethanolamine binding protein 1	6,35	2,15	5	117 543 681
3	1426959_at	BF322712	Bdh1	3-hydroxybutyrate dehydrogenase, type 1	6,88	2,63	16	31 342 040
3	1426753_at	BG065238	Phf17	PHD finger protein 17	6,59	4,84	3	41 677 743
3	1425496_at	AK007703	Abca3	ATP-binding cassette, sub-family A (ABC1), member 3	7,27	3,14	17	24 079 645
3	1421163_a_at	AF326553	Nfia	nuclear factor I/A	7,42	4,79	4	97 269 916
3	1458719_at	BM233846			6,73	7,08	17	
3	1440771_at	BB093204	Zkscan1	zinc finger with KRAB and SCAN domains 1	6,37	5,68	5	138 314 871
3	1441955_s_at	BB381990	Paip1	polyadenylate binding protein-interacting protein 1	6,35	5,80	13	120 547 808
3	1417462_at	NM_007598	Cap1	CAP, adenylate cyclase-associated protein 1 (yeast)	6,98	3,14	4	122 361 357
3	1435129_at	AW495875	Ptp4a2	protein tyrosine phosphatase 4a2	5,71	4,71	4	129 341 776
3	1416776_at	NM_016669	Crym	crystallin, mu	6,64	3,06	7	119 977 532
3	1453172_at	BE533039	Stch	stress 70 protein chaperone, microsome-associated, human homolog	6,18	6,57	16	75 637 874
3	1424108_at	BC024663	Glo1	glyoxalase 1	6,40	5,67	17	30 319 872
3	1457666_s_at	AV229143	Ifi202b	interferon activated gene 202B	5,30	5,71	1	175 799 250
3	1438663_at	BF730196	Bat2d	BAT2 domain containing 1	5,31	5,32	1	
3	1426995_a_at	BI901126	Gfer	growth factor, erv1 (S. cerevisiae)-like (augmenter of liver regeneration)	6,04	4,08	17	24 420 790
3	1430889_a_at	AK002335	Tpmt	thiopurine methyltransferase	5,31	6,42	13	47 036 150
3	1434500_at	BF585303	Ttyh2	tweety homolog 2 (Drosophila)	5,69	2,06	11	114 491 557
3	1426876_at	BC025830	4732466D17Rik	RIKEN cDNA 4732466D17 gene	5,06	2,35	1	
3	1455726_at	BB756522	Gm71	gene model 71, (NCBI)	5,99	4,32	12	70 496 234
3	1450484_a_at	AK004595	Tyki	thymidylate kinase family LPS-inducible member	5,32	2,07	12	27 055 649
3	1421551_s_at	NM_011940	Ifi202b	interferon activated gene 202B	5,55	4,47	1	175 799 250
3	1438980_x_at	BB027848	4732466D17Rik	RIKEN cDNA 4732466D17 gene	5,82	2,29	1	
3	1444073_at	AV352375	Maf	avian musculoaponeurotic fibrosarcoma (v-maf) AS42 oncogene homolog	4,69	2,36	8	118 589 229
3	1452302_at	AV345051	Arhgef10	Rho guanine nucleotide exchange factor (GEF) 10	4,30	3,01	8	14 911 693
3	1424877_a_at	BC018236	Alad	aminolevulinate, delta-, dehydratase	5,54	4,40	4	61 995 530
3	1424109_a_at	BC024663	Glo1	glyoxalase 1	4,38	4,98	17	30 319 872
3	1428692_at	AW259452	Hddc3	HD domain containing 3	6,47	7,52	7	80 216 649
3	1449018_at	NM_011072	Pfn1	profilin 1	4,43	4,30	11	70 468 043
3	1425764_a_at	AF031467	Bcat2	branched chain aminotransferase 2, mitochondrial	5,47	3,35	7	45 438 404
3	1417461_at	NM_007598	Cap1	CAP, adenylate cyclase-associated protein 1 (yeast)	5,95	5,10	4	122 361 357
3	1429509_at	BB771548	2600001B17Rik	RIKEN cDNA 2600001B17 gene	4,96	5,98	11	101 979 578
3	1444530_at	BE686339			4,21	3,61	2	
3	1417298_at	BC027422	Ebpl	emopamil binding protein-like	3,82	3,56	14	60 294 870
3	1425343_at	BC003491	Hdhd3	haloacid dehalogenase-like hydrolase domain containing 3	4,44	4,35	4	61 985 415
3	1423626_at	BB150886	Dst	dystonin	4,12	4,38	1	34 104 877
3	1443128_at	BB522781	Ints6	integrator complex subunit 6	5,03	2,05	14	61 630 437
3	1452439_s_at	AF250135	Sfrs2	splicing factor, arginine/serine-rich 2 (SC-35)	5,00	6,03	11	116 665 996
3	1416709_a_at	NM_031375	Ngrn	neugrin, neurite outgrowth associated	4,28	3,31	7	80 134 732
3	1430519_a_at	AK007767	Cnot7	CCR4-NOT transcription complex, subunit 7	4,82	5,89	8	41 991 852
3	1451240_a_at	BC024663	Glo1	glyoxalase 1	4,88	4,64	17	30 319 872
3	1455908_a_at	AV102733	Scpep1	serine carboxypeptidase 1	3,93	5,83	11	88 740 111
3	1451809_s_at	AF439556	Rwdd3	RWD domain containing 3	3,98	2,23	3	121 147 887
3	1449183_at	NM_007744	Comt	catechol-O-methyltransferase	4,77	3,89	16	18 321 105
3	1440651_at	BM238701	Dusp16	dual specificity phosphatase 16	4,06	6,38	6	134 681 162
3	1438831_at	BB208315	Crkrs	Cdc2-related kinase, arginine/serine-rich	4,56	5,04	11	98 019 403
3	1427153_at	AW047304	Bckdhb	branched chain ketoacid dehydrogenase E1, beta polypeptide	4,54	2,73	9	83 745 498
3	1460674_at	BC022922	Paqr7	progestin and adipoQ receptor family member VII	4,44	4,06	4	133 769 053
3	1428944_at	BB417360	Ube1l2	ubiquitin-activating enzyme E1-like 2	3,98	3,98	5	87 185 296
3	1452195_s_at	AW744519	Eef2	eukaryotic translation elongation factor 2	4,68	8,24	10	80 579 787
3	1430026_at	AK021006	Stch	stress 70 protein chaperone, microsome-associated, human homolog	4,16	4,11	16	75 637 874
3	1439272_at	BB183240	Lcorl	ligand dependent nuclear receptor corepressor-like	4,00	4,52	5	46 018 585
3	1447901_x_at	BB169770	4933439C20Rik	RIKEN cDNA 4933439C20 gene	4,55	5,02	11	3 025 788
3	1417265_s_at	AK014348	Coq5	coenzyme Q5 homolog, methyltransferase (yeast)	4,04	2,74	5	115 540 700

3	1456573_x_at	BB205930	Nnt	nicotinamide nucleotide transhydrogenase	4,25	5,01	13	120 453 189
3	1451683_x_at	M34962	H2K1	histocompatibility 2, K1, K region	3,59	3,36	17	
3	1439197_at	BB371241	Pik4cb	phosphatidylinositol 4-kinase, catalytic, beta polypeptide	4,14	4,16	3	95 060 139
3	1435267_at	BB041868	2610005L07Rik	RIKEN cDNA 2610005L07 gene	3,70	3,34	8	20 272 528
3	1448693_at	NM_024472	BC002216	cDNA sequence BC002216	3,50	2,24	4	154 708 522
3	1415896_x_at	NM_013670	Snrpn	small nuclear ribonucleoprotein N	3,63	3,28	7	59 862 065
3	1424454_at	BC027354	Tmem87a	transmembrane protein 87A	3,66	6,97	2	120 046 756
3	1458128_at	BB363084	Rpe	ribulose-5-phosphate-3-epimerase	4,40	4,69	1	66 634 100
3	1442886_at	BB667153			4,55	3,75	6	49 189 828
3	1418172_at	AF117613	Hebp1	heme binding protein 1	3,22	2,10	6	135 103 220
3	1445773_at	BG070088	Meis1	myeloid ecotropic viral integration site 1	3,21	2,99	11	18 780 432
3	1456518_at	BG068263	4930422I07Rik	RIKEN cDNA 4930422I07 gene	4,36	3,35	9	
3	1424469_a_at	BC025045	Cpsf4	cleavage and polyadenylation specific factor 4	4,03	2,88	5	145 420 750
3	1439241_x_at	BB825787	Srd5a2l	steroid 5 alpha-reductase 2-like	3,11	2,27	5	77 215 051
3	1439300_at	BG065782	Chic1	cysteine-rich hydrophobic domain 1	4,12	2,43	X	99 559 274
3	1457262_at	BB024162	2610207I05Rik	RIKEN cDNA 2610207I05 gene	3,97	3,67	7	117 922 460
3	1457088_at	BB780781	Pldn	pallidin	3,19	5,39	2	122 429 945
3	1450685_at	BE648432	Arpp19	cAMP-regulated phosphoprotein 19	3,56	2,41	9	74 824 336
3	1440604_at	BB070941	Eif4g3	eukaryotic translation initiation factor 4 gamma, 3	3,30	2,39	4	137 265 545
3	1419371_s_at	NM_019650	Gosr2	golgi SNAP receptor complex member 2	3,26	2,23	11	103 492 950
3	1415895_at	NM_013670	Snrpn	small nuclear ribonucleoprotein N	3,63	2,70	7	59 862 065
3	1415710_at	BM123013	Cox18	COX18 cytochrome c oxidase assembly homolog (S. cerevisiae)	2,87	3,58	5	91 289 926
3	1428393_at	AK003046	Nrn1	neuritin 1	3,89	3,35	13	36 733 092
3	1436740_at	AI585679	2610005L07Rik	RIKEN cDNA 2610005L07 gene	3,21	2,78	8	20 272 528
3	1455936_a_at	BG069460	Rbpms	RNA binding protein gene with multiple splicing	2,70	3,50	8	35 248 578
3	1441158_at	BB707145	Chn2	chimerin (chimaerin) 2	3,74	2,67	6	54 202 398
3	1425191_at	BC019407	9430098E02Rik	RIKEN cDNA 9430098E02 gene	3,72	5,60	8	74 300 341
3	1435764_a_at	BB832885	Gemin7	gem (nuclear organelle) associated protein 7	2,83	2,08	7	18 723 470
3	1438049_at	AW541326	2610005L07Rik	RIKEN cDNA 2610005L07 gene	3,95	2,38	8	20 272 528
3	1439055_at	AU022548	LOC217066		3,67	4,07	11	88 780 758
3	1447517_at	BM208991	Skiv2l2	superkiller viralicidic activity 2-like 2 (S. cerevisiae)	3,45	5,50	13	113 988 659
3	1421473_at	BC003727	Il1a	interleukin 1 alpha	3,07	2,68	2	128 991 050
3	1460121_at	BI076710	9630010G10Rik	RIKEN cDNA 9630010G10 gene	2,96	3,41	17	
3	1423255_at	BI154058	Atp6v1g1	ATPase, H+ transporting, lysosomal V1 subunit G1	3,58	2,70	4	63 031 197
3	1450900_at	AV305633	AW011752	expressed sequence AW011752	2,70	3,86	11	29 072 995
3	1422028_a_at	BC010588	Ets1	E26 avian leukemia oncogene 1, 5' domain	3,45	3,81	9	32 445 606
3	1421063_s_at	NM_033174	Snurf	SNRPN upstream reading frame	3,47	2,18	7	59 867 551
3	1423129_at	BQ032685	Shoc2	soc-2 (suppressor of clear) homolog (C. elegans)	3,25	4,66	19	53 998 191
3	1431241_at	AA692147	Chchd3	coiled-coil-helix-coiled-coil-helix domain containing 3	3,47	5,15	6	32 722 384
3	1426603_at	BF714880	Rnasel	ribonuclease L (2', 5'-oligoisoadenylate synthetase-dependent)	2,73	4,69	1	155 515 147
3	1417920_at	NM_033603	Amn	amnionless	2,84	2,49	12	111 718 918
3	1427998_at	AV015526	2600001B17Rik	RIKEN cDNA 2600001B17 gene	3,77	4,50	11	101 979 578
3	1436758_at	AI661423	Hdac4	histone deacetylase 4	2,92	3,45	1	93 763 153
3	1422891_at	U13648	H2Ea	histocompatibility 2, class II antigen E alpha	3,47	2,82	17	33 949 793
3	1455316_x_at	AI987693	Kcnh6	potassium voltage-gated channel, subfamily H (eag-related), member 6	2,62	2,73	11	105 824 292
3	1451251_at	BC018442	Appbp2	amyloid beta precursor protein (cytoplasmic tail) binding protein 2	2,84	3,78	11	85 007 505
3	1457306_at	C86690			3,11	4,67	4	
3	1458491_at	BI650220	4930422I07Rik	RIKEN cDNA 4930422I07 gene	3,46	3,46	9	
3	1420612_s_at	BE134116	Ptp4a2	protein tyrosine phosphatase 4a2	2,97	2,10	4	129 341 776
3	1456857_at	BB782615	1500011B03Rik	RIKEN cDNA 1500011B03 gene	2,88	5,84	5	
3	1452997_at	BE692399	2610005L07Rik	RIKEN cDNA 2610005L07 gene	2,96	3,11	8	20 272 528
3	1419741_at	AW536705	Supt16h	suppressor of Ty 16 homolog (S. cerevisiae)	2,96	4,56	14	51 082 369
3	1434328_at	BI081163	Rpl15	ribosomal protein L15	3,18	2,27	9	17 062 078
3	1431056_a_at	AK017272	Lpl	lipoprotein lipase	2,94	3,80	8	71 809 542
3	1457483_at	BM214036	Arid5b	AT rich interactive domain 5B (Mrf1 like)	2,94	3,04	10	67 490 949
3	1436070_at	BM933153	Glo1	glyoxalase 1	3,09	2,58	17	30 319 872
3	1428778_at	AK019095	Sfi1	Sfi1 homolog, spindle assembly associated (yeast)	3,25	3,85	11	3 031 887
3	1417571_at	BC014754	Mpg	N-methylpurine-DNA glycosylase	3,11	2,52	11	32 126 510
3	1452303_at	AV345051	Arhgef10	Rho guanine nucleotide exchange factor (GEF) 10	3,09	2,65	8	14 911 693
3	1427797_s_at	BF580235	Ctse	cathepsin E	2,92	6,93	1	133 465 859
3	1430384_at	AK017548	Tle4	transducin-like enhancer of split 4, homolog of Drosophila E(spl)	2,94	2,45	19	14 515 090

3	1431007_at	AA153045	9030025P20Rik	RIKEN cDNA 9030025P20 gene	2,90	5,52	17	14 726 722
3	1429882_at	AK012880	2610005L07Rik	RIKEN cDNA 2610005L07 gene	3,35	2,12	8	20 272 528
3	1426359_at	AV328883	Zc3h11a	zinc finger CCCH type containing 11A	2,98	5,40	1	135 449 061
3	1444722_at	BG075584	Psme4	proteasome (prosome, macropain) activator subunit 4	3,00	5,28	11	30 672 063
3	1454696_at	BG071068	Gnb1	guanine nucleotide binding protein, beta 1	2,77	2,92	4	154 371 249
3	1457007_at	BI793615	Ncor1	nuclear receptor co-repressor 1	2,89	3,40	11	62 132 884
3	1452776_a_at	BG066220	Nub1	negative regulator of ubiquitin-like proteins 1	2,77	3,18	5	24 196 039
3	1452020_a_at	AF033112	Siva	Cd27 binding protein (Hindu God of destruction)	2,74	2,40	12	113 092 719
3	1438082_at	BB279158	2310028N02Rik	RIKEN cDNA 2310028N02 gene	2,81	2,60	1	193 026 579
3	1422554_at	NM_023239	Ndnl2	necdin-like 2	2,94	2,09	7	64 750 599
3	1422128_at	NM_025974	Rpl14	ribosomal protein L14	2,59	3,40	9	120 420 213
3	1451567_a_at	BC008167	Ifi203	interferon activated gene 203	2,86	3,48	1	175 757 079
3	1437358_at	BM233251	Wdfy1	WD repeat and FYVE domain containing 1	2,74	5,43	1	79 605 439
3	1435588_at	BQ031098	Wdfy1	WD repeat and FYVE domain containing 1	2,62	3,25	1	79 605 439
3	1416411_at	NM_008183	Gstm2	glutathione S-transferase, mu 2	2,62	2,78	3	108 109 758
3	1456262_at	BE446879	Rbm5	RNA binding motif protein 5	2,79	4,71	9	107 598 601
3	1459253_at	AW556597	Arrdc3	arrestin domain containing 3	2,25	6,73	13	81 347 116
3	1424749_at	BC025226	Wdfy1	WD repeat and FYVE domain containing 1	2,31	3,87	1	79 605 439
3	1428617_at	AK018461	Hcfc2	host cell factor C2	2,31	2,83	10	
3	1441529_at	BB325889	B3gnt2	UDP-GlcNAc:betaGal beta-1,3-N-acetylglucosaminyltransferase 2	2,02	6,22	11	22 734 747
3	1427798_x_at	BF580235			2,50	4,74	?	
3	1426093_at	AF220141	Trim34	tripartite motif protein 34	2,49	2,82	7	104 118 384
3	1460691_at	BF385956	Zfp598	zinc finger protein 598	2,50	3,43	17	24 397 351
3	1419469_at	BI713933	Gnb4	guanine nucleotide binding protein, beta 4	2,25	4,45	3	32 775 076
3	1449578_at	AW536705	Supt16h	suppressor of Ty 16 homolog (S. cerevisiae)	2,40	3,19	14	51 082 369
3	1426361_at	AV328883	Zc3h11a	zinc finger CCCH type containing 11A	2,38	3,79	1	135 449 061
3	1444177_at	AI451538	LOC626058		2,17	5,04	1	
3	1426555_at	AK014680	Scpep1	serine carboxypeptidase 1	2,42	7,69	11	88 740 111
3	1442944_at	BG065699	Luzp1	leucine zipper protein 1	2,44	4,23	4	135 793 467
3	1446904_at	AU040633	Arhgef11	Rho guanine nucleotide exchange factor (GEF) 11	2,52	4,23	3	87 704 677
3	1451931_x_at	M69068	H2L	histocompatibility 2, D region	2,27	3,55	17	
3	1432393_a_at	AK007233	5730409G07Rik	RIKEN cDNA 5730409G07 gene	2,10	2,96	11	45 794 601
3	1448568_a_at	NM_015747	Slc20a1	solute carrier family 20, member 1	2,43	3,49	2	128 890 240
3	1453589_a_at	BI737178	2610005L07Rik	RIKEN cDNA 2610005L07 gene	2,34	4,50	8	20 272 528
3	1445226_at	BI664409	BC023969	cDNA sequence BC023969	2,16	3,73	5	
3	1441414_at	BG075856	Eef2	eukaryotic translation elongation factor 2	2,48	4,60	10	80 579 787
3	1434313_at	BB762434	6330407D12Rik	RIKEN cDNA 6330407D12 gene	2,03	2,76	6	49 248 938
3	1443557_at	BG071446			2,15	2,79	18	
3	1449051_at	BC016892	Ppara	peroxisome proliferator activated receptor alpha	2,48	3,56	15	85 563 541
3	1435459_at	BM936480	Fmo2	flavin containing monooxygenase 2	2,21	3,82	1	164 711 723
3	1425113_x_at	BC020068			2,52	5,47	?	
3	1453435_a_at	AK009753	Fmo2	flavin containing monooxygenase 2	2,13	4,14	1	164 711 723
3	1418658_at	BC025158	2410005O16Rik	RIKEN cDNA 2410005O16 gene	2,28	3,22	4	19 502 353
3	1437904_at	BB821609	Drbp1	developmentally regulated RNA binding protein 1	2,01	4,37	2	76 170 822
3	1441584_at	BB555654	Fli1	Friend leukemia integration 1	2,19	3,87	9	32 171 774
3	1426360_at	AV328883	Zc3h11a	zinc finger CCCH type containing 11A	2,13	3,71	1	135 449 061
3	1425545_x_at	M86502	H2L	histocompatibility 2, D region	2,14	2,99	17	
3	1440144_x_at	BB821700	C330046E03		2,12	2,69	14	
3	1442108_at	BB559647	Rnf25	ring finger protein 25	2,31	3,12	1	74 526 958
3	1428013_at	BC028769	6030458C11Rik	RIKEN cDNA 6030458C11 gene	2,20	4,40	15	12 753 139
3	1437089_at	AV223337	3830409H07Rik	RIKEN cDNA 3830409H07 gene	2,21	2,75	19	
3	1432181_s_at	AK013765	Ecgf1	endothelial cell growth factor 1 (platelet-derived)	2,15	5,00	15	89 199 697
3	1438220_at	BM240405	Foxj3	forkhead box J3	2,09	3,16	4	119 037 619
3	1417703_at	NM_008990	Pvrl2	poliovirus receptor-related 2	2,03	2,81	7	18 875 185
4	1421144_at	NM_023879	Rpgrip1	retinitis pigmentosa GTPase regulator interacting protein 1	-9,03	-8,49	14	51 032 872
4	1436240_at	BM211445	Sost	sclerostin	-9,39	-10,19	11	101 778 625
4	1438758_at	AU046270			-9,57	-3,51	?	
4	1435872_at	BE631223	Pim1	proviral integration site 1	-8,45	-9,49	17	29 217 823
4	1452231_x_at	M74124	Ifi205	interferon activated gene 205	-7,78	-4,99	1	175 848 673
4	1419523_at	NM_007819	Cyp3a13	cytochrome P450, family 3, subfamily a, polypeptide 13	-8,17	-2,13	5	138 122 720
4	1426906_at	M74124	Ifi205	interferon activated gene 205	-7,59	-5,52	1	175 848 673

4	1456655_at	BM231698	Ext1	exostoses (multiple) 1	-7,89	-6,17	15	52 898 621
4	1438691_at	BB379602	Zzef1	zinc finger, ZZ-type with EF hand domain 1	-8,57	-7,63	11	72 729 457
4	1456257_at	BG070093	C130065N10Rik	RIKEN cDNA C130065N10 gene	-7,70	-6,54	1	
4	1457991_at	BB052061	Abcd3	ATP-binding cassette, sub-family D (ALD), member 3	-6,70	-6,71	3	121 750 932
4	1452705_at	AK004611	2210010A19Rik	RIKEN cDNA 2210010A19 gene	-7,45	-9,68	16	13 747 507
4	1438936_s_at	AI385586	Rnase4	ribonuclease, RNase A family 4	-7,23	-4,22	14	50 013 146
4	1443100_at	BB523900	Thrb	thyroid hormone receptor beta	-7,42	-4,54	14	16 774 841
4	1451457_at	AB016248	Sc5d	sterol-C5-desaturase (fungal ERG3, delta-5-desaturase) homolog (S. cerevisae)	-7,23	-6,60	9	42 005 171
4	1419636_at	BG067119	4833420G17Rik	RIKEN cDNA 4833420G17 gene	-6,23	-5,10	13	120 581 646
4	1424226_at	BC021385	9030617O03Rik	RIKEN cDNA 9030617O03 gene	-5,82	-2,55	12	101 180 172
4	1438294_at	BG067317	Atxn1	ataxin 1	-7,11	-4,36	13	45 565 864
4	1426584_a_at	BI143942	Sord	sorbitol dehydrogenase	-6,78	-4,51	2	121 926 279
4	1459917_at	BG078867	AI451896	expressed sequence AI451896	-7,12	-8,08	11	84 685 063
4	1453571_at	BB324973	Depdc6	DEP domain containing 6	-6,28	-2,16	15	54 963 518
4	1430979_a_at	AK011963	Prdx2	peroxiredoxin 2	-5,65	-6,95	8	87 859 726
4	1437478_s_at	AA409309	Efhd2	EF hand domain containing 2	-6,22	-5,16	4	141 130 217
4	1445543_at	AI428811	9830124H08Rik	RIKEN cDNA 9830124H08 gene	-5,51	-6,94	14	55 747 430
4	1419839_x_at	AA409562	Prpf19	PRP19/PSO4 pre-mRNA processing factor 19 homolog (S. cerevisiae)	-5,44	-2,45	19	10 962 336
4	1437128_a_at	BB391874	A630033E08Rik	RIKEN cDNA A630033E08 gene	-6,00	-6,21	17	22 552 193
4	1451602_at	BC025911	Snx6	sorting nexin 6	-7,00	-9,07	12	55 667 982
4	1422591_at	BI965936	Tceb3	transcription elongation factor B (SIII), polypeptide 3	-5,22	-3,09	4	135 275 445
4	1424893_at	BC021434	Ndel1	nuclear distribution gene E-like homolog 1 (A. nidulans)	-7,19	-8,06	11	68 637 643
4	1441315_s_at	BB449198	Slc19a2	solute carrier family 19 (thiamine transporter), member 2	-6,69	-7,52	1	166 085 729
4	1458947_at	AV341977	2010111I01Rik	RIKEN cDNA 2010111I01 gene	-5,81	-4,82	13	63 024 556
4	1443027_at	BB667435	4930523C07Rik	RIKEN cDNA 4930523C07 gene	-6,79	-4,12	1	161 881 063
4	1423696_a_at	BC006869	Psmd6	proteasome (prosome, macropain) 26S subunit, non-ATPase, 6	-5,26	-5,01	14	12 905 451
4	1433953_at	BQ176535	Zfp277	zinc finger protein 277	-5,47	-7,05	12	40 825 468
4	1459747_at	AI836671			-6,55	-7,71	4	
4	1457458_at	BG076273	BC057627	cDNA sequence BC057627	-6,11	-5,67	7	15 559 772
4	1436630_at	AI550443	Aqp11	aquaporin 11	-5,26	-4,40	7	97 601 570
4	1441423_at	BB344763	Eif4g3	eukaryotic translation initiation factor 4 gamma, 3	-6,03	-2,35	4	137 265 545
4	1444037_at	BE981934	Lman1	lectin, mannose-binding, 1	-6,13	-6,64	18	66 106 125
4	1443908_at	BB205833			-6,11	-3,59	17	
4	1426951_at	AK018666	Crim1	cysteine rich transmembrane BMP regulator 1 (chordin like)	-6,85	-3,91	17	78 105 543
4	1433758_at	BB025231	Nisch	nischarin	-6,14	-4,64	14	29 999 938
4	1425054_a_at	BC024696	2510006D16Rik	RIKEN cDNA 2510006D16 gene	-5,56	-5,39	4	129 103 010
4	1448775_at	NM_008328	Ifi203	interferon activated gene 203	-6,44	-4,24	1	175 757 079
4	1459187_at	BG075096	St3gal4	ST3 beta-galactoside alpha-2,3-sialyltransferase 4	-4,83	-3,94	9	34 796 249
4	1416142_at	NM_009096	Rps6	ribosomal protein S6	-5,26	-4,24	4	86 325 329
4	1446130_at	AW557530	Pctk2	PCTAIRE-motif protein kinase 2	-5,86	-5,22	10	92 638 098
4	1425134_a_at	BC002202	Pigx	phosphatidylinositol glycan anchor biosynthesis, class X	-4,57	-3,77	16	32 004 170
4	1421907_at	BB760479	Pparbp	peroxisome proliferator activated receptor binding protein	-6,73	-6,08	11	97 970 338
4	1419258_at	BC006022	Tcea1	transcription elongation factor A (SII) 1	-4,64	-5,18	1	4 847 894
4	1436332_at	BB755506	Hspb6	heat shock protein, alpha-crystallin-related, B6	-5,27	-5,17	7	30 262 061
4	1455208_at	AV229364	Pex19	peroxisome biogenesis factor 19	-5,11	-4,35	1	173 963 446
4	1434375_at	BB699417	B930006L02Rik	RIKEN cDNA B930006L02 gene	-5,51	-4,57	7	100 580 788
4	1448822_at	BC013897	Psmb6	proteasome (prosome, macropain) subunit, beta type 6	-5,25	-4,79	11	70 341 602
4	1418662_at	NM_025617	2210012G02Rik	RIKEN cDNA 2210012G02 gene	-5,03	-4,03	4	106 636 754
4	1457847_at	BM238972	Rfxdc2	regulatory factor X domain containing 2 homolog (human)	-4,53	-6,21	9	72 330 806
4	1429331_at	AK019505	4632427E13Rik	RIKEN cDNA 4632427E13 gene	-5,81	-6,19	7	92 614 084
4	1417124_at	NM_019771	Dstn	destrin	-5,12	-3,66	2	143 607 051
4	1456663_x_at	BB718785	Tm2d2	TM2 domain containing 2	-4,53	-3,52	8	26 482 824
4	1453749_at	BB749723	2610507I01Rik	RIKEN cDNA 2610507I01 gene	-5,85	-3,47	11	59 014 454
4	1425336_x_at	BC011306	H2K1	histocompatibility 2, K1, K region	-5,10	-2,13	17	
4	1438238_at	BB744779	2010315B03Rik	RIKEN cDNA 2010315B03 gene	-6,24	-6,19	9	
4	1429691_at	AK017277	5430405N12Rik	RIKEN cDNA 5430405N12 gene	-4,37	-5,61	14	
4	1449001_at	AV221988	Ivd	isovaleryl coenzyme A dehydrogenase	-4,22	-2,62	2	118 553 440
4	1419637_s_at	BG067119	4833420G17Rik	RIKEN cDNA 4833420G17 gene	-4,37	-5,33	13	120 581 646
4	1441573_at	BB297140	Scmh1	sex comb on midleg homolog 1	-4,39	-3,09	4	119 903 043
4	1439059_at	BB709811	BC031748	cDNA sequence BC031748	-4,68	-5,93	X	132 395 992
4	1424039_at	BC022616	Tmem66	transmembrane protein 66	-4,92	-4,68	8	35 623 104

4	1434906_at	AI182092	0610005C13Rik	RIKEN cDNA 0610005C13 gene	-5,00	-3,56	7	45 435 823
4	1436358_at	AV333459	Atpaf1	ATP synthase mitochondrial F1 complex assembly factor 1	-4,06	-2,56	4	115 282 915
4	1424857_a_at	AF220142	Trim34	tripartite motif protein 34	-5,49	-7,66	7	104 118 384
4	1419635_at	BG067119	4833420G17Rik	RIKEN cDNA 4833420G17 gene	-5,59	-3,97	13	120 581 646
4	1438937_x_at	AI385586	Rnase4	ribonuclease, RNase A family 4	-4,55	-6,12	14	50 013 146
4	1437900_at	AV032877	4930523C07Rik	RIKEN cDNA 4930523C07 gene	-5,58	-3,59	1	161 881 063
4	1439363_at	BB736518	1200014J11Rik	RIKEN cDNA 1200014J11 gene	-4,06	-5,65	11	72 864 074
4	1426607_at	BG068672	Grik5	glutamate receptor, ionotropic, kainate 5 (gamma 2)	-4,69	-5,51	7	24 718 635
4	1434171_at	BB398891	C330011K17Rik	RIKEN cDNA C330011K17 gene	-4,80	-3,85	13	67 939 530
4	1428851_at	AK005003	1300014I06Rik	RIKEN cDNA 1300014I06 gene	-5,50	-2,53	13	34 635 304
4	1441238_at	BG074304	9030416H16Rik	RIKEN cDNA 9030416H16 gene	-3,92	-2,57	5	
4	1417648_s_at	NM_024225	Snx5	sorting nexin 5	-4,06	-4,03	2	143 941 568
4	1454973_at	BF456242	Atf7ip	activating transcription factor 7 interacting protein	-4,32	-3,84	6	136 483 046
4	1438130_at	BB427389	Taf15	TAF15 RNA polymerase II, TATA box binding protein (TBP)-associated factor	-4,44	-5,86	11	83 289 302
4	1416770_at	NM_021537	Stk25	serine/threonine kinase 25 (yeast)	-3,83	-6,17	1	95 451 156
4	1445263_at	BF148780	Cst7	cystatin F (leukocystatin)	-4,75	-6,80	2	150 261 874
4	1453485_s_at	AK016897	1110005A03Rik	RIKEN cDNA 1110005A03 gene	-3,64	-3,19	11	116 659 689
4	1441604_at	BM248597	Esd	esterase D/formylglutathione hydrolase	-4,57	-5,06	14	73 466 499
4	1444128_at	AI447325	Pip5k2b	phosphatidylinositol-4-phosphate 5-kinase, type II, beta	-4,85	-4,07	11	97 531 247
4	1438092_x_at	AV003424	H2afz	H2A histone family, member Z	-4,09	-2,24	3	137 801 985
4	1417034_at	NM_025960	Trappc6a	trafficking protein particle complex 6A	-4,82	-3,32	7	18 667 246
4	1452730_at	AK004068	1110033J19Rik	RIKEN cDNA 1110033J19 gene	-4,18	-4,57	6	148 311 853
4	1448214_at	AK011810	Pdhb	pyruvate dehydrogenase (lipoamide) beta	-5,09	-3,54	14	6 957 525
4	1437840_s_at	BB222646	LOC673662		-4,64	-5,38	Un	
4	1426581_at	BI247018	Ptpmt1	protein tyrosine phosphatase, mitochondrial 1	-4,70	-2,90	2	90 711 573
4	1434585_at	BB667130	Tulp4	tubby like protein 4	-3,90	-4,11	17	6 095 225
4	1444366_at	BE981006	Taok3	TAO kinase 3	-3,81	-3,60	5	
4	1452660_s_at	AK012326	Klhl7	kelch-like 7 (Drosophila)	-4,07	-2,59	5	23 610 676
4	1437432_a_at	BM244351	Trim12	tripartite motif protein 12	-3,71	-7,82	7	104 177 417
4	1441937_s_at	AV371921	Pink1	PTEN induced putative kinase 1	-4,47	-3,14	4	137 585 487
4	1428588_a_at	BF134369	Wdr85	WD40 repeat domain 85	-3,59	-2,33	2	24 784 453
4	1441437_at	BM119933	Rbms1	RNA binding motif, single stranded interacting protein 1	-4,57	-2,44	2	60 553 578
4	1419162_s_at	BE624323	Dnajc3	DnaJ (Hsp40) homolog, subfamily C, member 3	-3,62	-2,33	14	117 820 544
4	1455806_x_at	AV124207	Ndufa12	NADH dehydrogenase (ubiquinone) 1 alpha subcomplex, 12	-4,00	-2,84	10	93 628 807
4	1428137_at	AV233429	Arl8b	ADP-ribosylation factor-like 8B	-4,30	-4,94	6	108 748 886
4	1430274_a_at	AK018331	Stard3nl	STARD3 N-terminal like	-4,17	-3,20	13	19 365 142
4	1427425_at	BC026435	9130208E07Rik	RIKEN cDNA 9130208E07 gene	-4,16	-2,26	4	
4	1435704_at	BB552195	C920006O11Rik	RIKEN cDNA C920006O11 gene	-4,62	-3,08	9	
4	1421023_at	NM_011083	Pik3c2a	phosphatidylinositol 3-kinase, C2 domain containing, alpha polypeptide	-4,68	-2,02	7	116 130 709
4	1426921_at	AV309591	Abcf1	ATP-binding cassette, sub-family F (GCN20), member 1	-3,55	-2,29	17	35 564 872
4	1437660_at	AW987751	Nktr	natural killer tumor recognition sequence	-4,03	-5,94	9	121 567 878
4	1430292_a_at	BG792484	1810030N24Rik	RIKEN cDNA 1810030N24 gene	-4,43	-2,23	4	34 957 558
4	1433495_at	AV122321	Glt25d1	glycosyltransferase 25 domain containing 1	-3,68	-2,92	8	74 539 986
4	1441274_at	BB283974	Rabgef1	RAB guanine nucleotide exchange factor (GEF) 1	-4,46	-3,32	5	130 471 882
4	1428288_at	AW488885	2310051E17Rik	RIKEN cDNA 2310051E17 gene	-3,51	-3,82	19	
4	1434441_at	BB525584	1110018J18Rik	RIKEN cDNA 1110018J18 gene	-4,87	-5,71	13	64 301 605
4	1460352_s_at	BC017537	Pik3r4	phosphatidylinositol 3 kinase, regulatory subunit, polypeptide 4, p150	-3,65	-3,62	9	
4	1457721_at	AI118064	AI118064	expressed sequence AI118064	-4,13	-2,30	15	
4	1422675_at	AK018493	Smarce1	SWI/SNF related, matrix associated, actin dependent regulator of chromatin, subfamily e, member 1	-3,49	-2,79	11	99 025 139
4	1417619_at	BE368753	Gadd45gip1	growth arrest and DNA-damage-inducible, gamma interacting protein 1	-3,48	-2,10	8	87 722 386
4	1418585_at	NM_023243	Ccnh	cyclin H	-3,45	-2,17	13	85 662 586
4	1430653_at	AI481778	Vamp5	vesicle-associated membrane protein 5	-3,53	-2,85	6	72 298 541
4	1437708_x_at	BB552111	Vamp3	vesicle-associated membrane protein 3	-4,20	-3,94	4	149 891 104
4	1424345_s_at	BC021792	Ube2m	ubiquitin-conjugating enzyme E2M (UBC12 homolog, yeast)	-4,47	-4,01	7	11 935 417
4	1416247_at	NM_016890	Dctn3	dynactin 3	-4,49	-3,46	4	41 903 467
4	1419115_at	AA198774	Alg14	asparagine-linked glycosylation 14 homolog (yeast)	-3,73	-3,11	3	121 283 839
4	1437615_s_at	BB270615	Vps37c	vacuolar protein sorting 37C (yeast)	-4,34	-4,18	19	10 755 859
4	1450740_a_at	BM228525	Mapre1	microtubule-associated protein, RP/EB family, member 1	-3,85	-4,04	2	153 432 816
4	1419971_s_at	C86506	Slc35a5	solute carrier family 35, member A5	-3,97	-5,50	16	45 058 904
4	1438862_at	BB227434	Arntl	aryl hydrocarbon receptor nuclear translocator-like	-3,96	-7,23	7	112 998 645
4	1436494_x_at	BF319098	Trmt1	TRM1 tRNA methyltransferase 1 homolog (S. cerevisiae)	-3,51	-2,40	8	87 579 382

4	1429399_at	BB667823	Rnf125	ring finger protein 125	-3,64	-3,24	18	21 087 634
4	1443153_at	BB306866	Trip11	thyroid hormone receptor interactor 11	-4,06	-6,74	12	102 259 701
4	1436173_at	BB768194	Dlc1	deleted in liver cancer 1	-4,24	-3,28	8	38 036 380
4	1420810_at	NM_019769	1500003O03Rik	RIKEN cDNA 1500003O03 gene	-3,21	-2,42	2	119 239 147
4	1426975_at	BG067859	4632413K17Rik	RIKEN cDNA 4632413K17 gene	-3,36	-2,38	10	126 498 600
4	1429478_at	AK020138	6720463M24Rik	RIKEN cDNA 6720463M24 gene	-2,66	-4,08	14	97 929 510
4	1455914_at	AW554430	AI987944	expressed sequence AI987944	-3,43	-2,98	7	41 146 941
4	1438318_x_at	BB559925	Ngdn	neuroguidin, EIF4E binding protein	-2,79	-3,50	14	53 969 595
4	1434664_at	BI153133	2410129H14Rik	RIKEN cDNA 2410129H14 gene	-3,84	-4,33	14	97 917 683
4	1434624_x_at	AA762498	Rps9	ribosomal protein S9	-3,51	-7,58	7	3 307 127
4	1437142_a_at	BB546713	Pigo	phosphatidylinositol glycan anchor biosynthesis, class O	-3,60	-4,23	4	43 038 737
4	1416153_at	NM_011899	Srp54	signal recognition particle 54	-3,67	-2,55	12	55 998 584
4	1447526_at	C79489	D5Ertd255e	DNA segment, Chr 5, ERATO Doi 255, expressed	-3,34	-3,65	5	
4	1460709_a_at	BG071718	Bat5	HLA-B associated transcript 5	-4,21	-4,39	17	34 697 346
4	1441375_at	AU043080	Lrig1	leucine-rich repeats and immunoglobulin-like domains 1	-3,03	-5,90	6	94 569 992
4	1436229_at	BB256501	BC049806	cDNA sequence BC049806	-3,79	-2,58	1	58 471 801
4	1451448_a_at	BC026936	1110005A03Rik	RIKEN cDNA 1110005A03 gene	-3,44	-4,15	11	116 659 689
4	1436372_a_at	BB026304	2210010A19Rik	RIKEN cDNA 2210010A19 gene	-4,47	-2,06	16	13 747 507
4	1417012_at	AU021035	Sdc2	syndecan 2	-3,55	-2,47	15	32 865 311
4	1434493_at	BG092222	1810022K09Rik	RIKEN cDNA 1810022K09 gene	-3,38	-5,56	3	14 583 033
4	1448545_at	AU021035	Sdc2	syndecan 2	-3,32	-2,32	15	32 865 311
4	1433757_a_at	BB025231	Nisch	nischarin	-4,04	-3,50	14	29 999 938
4	1428748_at	BM202530	5830428H23Rik	RIKEN cDNA 5830428H23 gene	-3,13	-3,17	19	61 180 754
4	1441683_at	BM220862	1110033M05Rik	RIKEN cDNA 1110033M05 gene	-3,50	-3,59	13	
4	1431214_at	BG297038	1300007C21Rik	RIKEN cDNA 1300007C21 gene	-3,39	-4,66	18	
4	1446155_at	BB046613	2700089E24Rik	RIKEN cDNA 2700089E24 gene	-3,70	-2,60	6	
4	1430798_x_at	AV306676	Mrpl15	mitochondrial ribosomal protein L15	-3,69	-5,19	1	4 763 291
4	1458504_at	BB508669	Zc3h12d	zinc finger CCCH type containing 12D	-2,78	-2,13	10	7 522 659
4	1442568_at	BM213120	C330002I19Rik	RIKEN cDNA C330002I19 gene	-3,79	-5,91	12	25 616 563
4	1459698_at	BM123577	LOC622320		-3,67	-2,98	7	97 207 518
4	1422485_at	AK004804	Smad4	MAD homolog 4 (Drosophila)	-3,59	-5,05	18	73 764 382
4	1448533_at	NM_025548	Ckap1	cytoskeleton-associated protein 1	-3,58	-3,27	7	29 932 899
4	1438408_at	BB131927	Ankrd56	ankyrin repeat domain 56	-3,36	-4,24	5	94 116 374
4	1455600_at	BG069767	Rps3	ribosomal protein S3	-2,93	-2,43	7	99 353 009
4	1424746_at	BC016221	Kif1c	kinesin family member 1C	-3,25	-3,87	11	70 518 994
4	1420630_at	NM_028982	8430419L09Rik	RIKEN cDNA 8430419L09 gene	-3,41	-2,03	6	135 163 744
4	1440624_at	BB477150	Ptprd	protein tyrosine phosphatase, receptor type, D	-3,61	-2,86	4	75 412 469
4	1418386_at	NM_026526	2510005D08Rik	RIKEN cDNA 2510005D08 gene	-3,15	-3,80	14	56 503 707
4	1437774_at	BB778614	1700020I14Rik	RIKEN cDNA 1700020I14 gene	-2,62	-2,51	2	
4	1441001_at	BB045561	AI225934	expressed sequence AI225934	-3,44	-5,07	2	
4	1417040_a_at	NM_016778	Bok	Bcl-2-related ovarian killer protein	-2,67	-3,15	1	95 516 107
4	1454772_at	AI327392	Ascc3l1	activating signal cointegrator 1 complex subunit 3-like 1	-3,29	-4,09	2	126 899 844
4	1424615_at	BG063931	Frag1	FGF receptor activating protein 1	-2,67	-2,66	7	102 096 894
4	1435084_at	BB200607	C730049O14Rik	RIKEN cDNA C730049O14 gene	-3,14	-3,12	18	
4	1438719_at	AV381143	Map3k2	mitogen activated protein kinase kinase kinase 2	-3,24	-3,01	18	32 306 098
4	1418537_at	NM_026158	0610042E07Rik	RIKEN cDNA 0610042E07 gene	-3,23	-3,72	7	4 448 046
4	1415685_at	NM_133767	Mtif2	mitochondrial translational initiation factor 2	-2,84	-3,46	11	29 426 456
4	1436689_a_at	AV028069	Aldh9a1	aldehyde dehydrogenase 9, subfamily A1	-3,00	-4,79	1	169 186 887
4	1434933_at	AV307999	5730557L09Rik	RIKEN cDNA 5730557L09 gene	-2,60	-2,02	1	
4	1430485_at	AW324327	Trpc2	transient receptor potential cation channel, subfamily C, member 2	-3,36	-4,86	7	101 949 700
4	1425918_at	BC022961	Egln3	EGL nine homolog 3 (C. elegans)	-2,93	-4,00	12	55 100 607
4	1438646_x_at	BB326325	2510039O18Rik	RIKEN cDNA 2510039O18 gene	-3,36	-4,34	4	146 784 694
4	1441413_at	BM247599	Cat	catalase	-2,86	-2,79	2	103 254 842
4	1421385_a_at	NM_008663	Myo7a	myosin VIIa	-3,62	-4,24	7	97 926 520
4	1448753_at	BI661964	Srp9	signal recognition particle 9	-3,26	-3,59	1	183 961 424
4	1454703_x_at	BF163381	Snord22	small nucleolar RNA, C/D box 22	-2,86	-2,36	19	
4	1434934_at	BB771055	Atpaf1	ATP synthase mitochondrial F1 complex assembly factor 1	-3,63	-3,50	4	115 282 915
4	1441440_at	BB530484	Atg4c	autophagy-related 4C (yeast)	-2,92	-3,27	4	98 685 951
4	1438230_at	BM240147	Pggt1b	protein geranylgeranyltransferase type I, beta subunit	-3,01	-4,10	18	46 365 279
4	1447206_at	AW552490	Arhgap21	Rho GTPase activating protein 21	-3,62	-5,72	2	
4	1441779_at	BB750043	9530006C21Rik	RIKEN cDNA 9530006C21 gene	-3,09	-3,50	8	35 394 097

4	1448131_at	NM_133201	Mfn2	mitofusin 2	-2,86	-3,30	4	146 717 398
4	1423487_at	BB775592	Cript	cysteine-rich PDZ-binding protein	-2,58	-3,44	17	86 933 921
4	1436684_a_at	AV066689	Riok2	RIO kinase 2 (yeast)	-3,62	-3,31	17	17 079 193
4	1446972_at	BG063165	D15Wsu126e	DNA segment, Chr 15, Wayne State University 126, expressed	-3,30	-4,41	15	
4	1459973_x_at	BB228059	Dpp4	dipeptidylpeptidase 4	-3,63	-2,43	2	62 132 578
4	1447458_at	BE954474	St3gal4	ST3 beta-galactoside alpha-2,3-sialyltransferase 4	-2,94	-3,96	9	34 796 249
4	1447894_x_at	BB429200	Vps52	vacuolar protein sorting 52 (yeast)	-2,77	-4,61	17	33 566 265
4	1441404_at	BG075643	Pafah1b1	platelet-activating factor acetylhydrolase, isoform 1b, beta1 subunit	-3,71	-6,52	11	74 490 149
4	1438504_x_at	BB120486	Tm7sf3	transmembrane 7 superfamily member 3	-3,31	-4,38	6	146 560 772
4	1440830_at	AW547876	Gpr116	G protein-coupled receptor 116	-3,25	-2,76	17	
4	1425665_a_at	BC005543	Srp54	signal recognition particle 54	-2,78	-2,47	12	55 998 584
4	1456991_at	BM237837	Cobll1	Cobl-like 1	-3,42	-2,95	2	64 890 150
4	1447164_at	BM198308	Rlf	rearranged L-myc fusion sequence	-2,82	-2,55	4	
4	1429423_at	BB484739	4930518I15Rik	RIKEN cDNA 4930518I15 gene	-2,73	-3,33	2	
4	1441115_at	BB205273	Rnf125	ring finger protein 125	-2,58	-2,07	18	21 087 634
4	1459984_at	AI552688	Mia3	melanoma inhibitory activity 3	-3,12	-2,64	1, Un	1 161 236
4	1437295_at	AI507382	Pkn2	protein kinase N2	-2,69	-2,69	3	142 730 844
4	1417961_a_at	BM240719	Trim30	tripartite motif protein 30	-2,97	-2,38	7	104 282 848
4	1428507_at	AK014208	Hdhd2	haloacid dehalogenase-like hydrolase domain containing 2	-2,96	-2,84	18	77 148 867
4	1428820_at	BB464192	Mapre1	microtubule-associated protein, RP/EB family, member 1	-2,72	-4,57	2	153 432 816
4	1451749_at	BM220651	Irak4	interleukin-1 receptor-associated kinase 4	-2,71	-5,02	15	94 371 899
4	1456005_a_at	BB667581	Bcl2l11	BCL2-like 11 (apoptosis facilitator)	-3,49	-5,03	2	127 817 478
4	1418462_at	NM_019393	Exosc9	exosome component 9	-3,42	-2,28	3	36 744 191
4	1451218_at	BG064496	Edem1	ER degradation enhancer, mannosidase alpha-like 1	-2,76	-3,18	6	108 794 428
4	1438824_at	BB465699	Slc20a1	solute carrier family 20, member 1	-2,81	-3,33	2	128 890 240
4	1428228_at	AK013402	Pgm3	phosphoglucomutase 3	-3,06	-3,17	9	86 349 193
4	1452189_at	BG865652	Wdr82	WD repeat domain containing 82	-2,85	-3,83	9	
4	1436506_a_at	AW988981	1110008H02Rik	RIKEN cDNA 1110008H02 gene	-2,70	-4,06	1	
4	1435521_at	BQ174527	Msi2	Musashi homolog 2 (Drosophila)	-3,64	-5,46	11	88 155 654
4	1433593_at	BB316456	Ypel5	yippee-like 5 (Drosophila)	-3,27	-2,94	17	72 741 703
4	1454402_at	AK014195	3110048L19Rik	RIKEN cDNA 3110048L19 gene	-2,81	-5,29	17	
4	1441768_at	BB122864	D4Bwg0951e	DNA segment, Chr 4, Brigham & Women's Genetics 0951 expressed	-2,94	-4,42	4	80 381 926
4	1435491_at	BI076615	AI875089	expressed sequence AI875089	-2,81	-3,35	8	72 671 306
4	1421204_a_at	NM_029385	Nudt16	nudix (nucleoside diphosphate linked moiety X)-type motif 16	-2,69	-4,34	9	104 987 772
4	1416215_at	NM_016810	Gosr1	golgi SNAP receptor complex member 1	-3,08	-2,47	11	76 542 796
4	1442956_at	BG075869	Ppp1r13b	protein phosphatase 1, regulatory (inhibitor) subunit 13B	-2,63	-4,93	12	112 276 265
4	1455441_at	AW547374	Map3k7	mitogen activated protein kinase kinase kinase 7	-2,71	-2,55	4	32 292 734
4	1456746_a_at	BB334959	Cd99l2	Cd99 antigen-like 2	-2,97	-2,91	X	67 680 627
4	1452091_a_at	BM228459	Rbm28	RNA binding motif protein 28	-2,97	-2,77	6	29 075 167
4	1453475_at	BM123601	4930445K14Rik	RIKEN cDNA 4930445K14 gene	-3,10	-2,37	2	
4	1427407_s_at	AV013785	Trip11	thyroid hormone receptor interactor 11	-3,06	-3,40	12	102 235 058
4	1440977_at	BB201490	Akap13	A kinase (PRKA) anchor protein 13	-3,03	-3,22	7	?
4	1436569_at	BB360125	C030045D06Rik	RIKEN cDNA C030045D06 gene	-2,72	-2,28	1	11 249 176
4	1434778_at	AU022230			-2,76	-3,01	14	
4	1428196_a_at	AK004786	1200015F23Rik	RIKEN cDNA 1200015F23 gene	-2,90	-2,17	2	118 828 441
4	1428258_at	BG795140	2010107E04Rik	RIKEN cDNA 2010107E04 gene	-2,78	-2,72	12	112 409 183
4	1447703_x_at	AV214133	Zfp593	zinc finger protein 593	-2,76	-3,37	4	133 515 382
4	1449072_a_at	NM_026526	2510005D08Rik	RIKEN cDNA 2510005D08 gene	-2,68	-2,83	14	56 503 707
4	1423649_at	BC016240	Tmem68	transmembrane protein 68	-3,23	-3,51	4	3 476 187
4	1436848_x_at	AV348702	Impa1	inositol (myo)-1(or 4)-monophosphatase 1	-3,04	-3,85	3	10 296 071
4	1459894_at	BE631962	Iqgap2	IQ motif containing GTPase activating protein 2	-2,81	-2,17	13	
4	1431314_a_at	AK017936	5830417I10Rik	RIKEN cDNA 5830417I10 gene	-2,85	-2,66	3	88 906 895
4	1434968_a_at	BB758476	Actr3	ARP3 actin-related protein 3 homolog (yeast)	-2,60	-2,68	1	127 220 451
4	1438062_at	BM933984	4832420A03Rik	RIKEN cDNA 4832420A03 gene	-2,85	-2,27	7	97 565 107
4	1451458_at	BC019745	Tmem2	transmembrane protein 2	-2,77	-2,32	19	21 845 438
4	1444057_at	BB253461	Ubxd2	UBX domain containing 2	-2,65	-4,31	1	130 071 726
4	1459224_at	BB005980	Ppp1r13b	protein phosphatase 1, regulatory (inhibitor) subunit 13B	-2,75	-4,50	12	112 276 265
4	1457233_at	BB324466	Dnaja2	DnaJ (Hsp40) homolog, subfamily A, member 2	-2,69	-2,19	8	88 427 744
4	1423205_at	BB367487	Tm9sf4	transmembrane 9 superfamily protein member 4	-2,59	-3,41	2	152 852 743
4	1457028_at	BM226884	A430033K04Rik	RIKEN cDNA A430033K04 gene	-2,72	-2,61	5	138 852 681
4	1416409_at	AB034914	Acox1	acyl-Coenzyme A oxidase 1, palmitoyl	-2,67	-2,39	11	115 987 978

4	1439560_x_at	AV066667	LOC432995		-2,65	-3,01	16	
4	1456277_at	BB813490	7530414M10Rik	RIKEN cDNA 7530414M10 gene	-2,64	-2,84	15	
4	1434277_a_at	BG069663	6430570G24		-2,71	-2,32	11	86 752 622
4	1416205_at	NM_009752	Glb1	galactosidase, beta 1	-2,83	-2,48	9	114 249 816
4	1431266_at	BI102599	5830433G22Rik	RIKEN cDNA 5830433G22 gene	-2,83	-3,80	1	
4	1456099_at	BB312474	Hnrpc	heterogeneous nuclear ribonucleoprotein C	-2,84	-2,51	14	50 996 749
4	1439650_at	BG072267	Rtn4	reticulon 4	-2,57	-3,95	11	29 618 569
4	1434118_at	BI737397	0610009K11Rik	RIKEN cDNA 0610009K11 gene	-2,42	-2,95	4	137 706 747
4	1440455_at	AI848599	AI848599	expressed sequence AI848599	-2,54	-3,24	4	
4	1425266_a_at	BC011279	Rap1gds1	RAP1, GTP-GDP dissociation stimulator 1	-2,25	-2,88	3	138 863 292
4	1454795_at	AV080881	Cobll1	Cobl-like 1	-2,07	-2,65	2	64 890 150
4	1436951_x_at	BE623245	Txndc9	thioredoxin domain containing 9	-2,57	-2,72	1	37 928 412
4	1439185_x_at	BB433489	D430028G21Rik	RIKEN cDNA D430028G21 gene	-2,55	-3,10	2	130 925 578
4	1459376_at	BM120619	Hivep2	human immunodeficiency virus type I enhancer binding protein 2	-2,07	-2,62	10	13 656 576
4	1424721_at	BI661422	Mfap3	microfibrillar-associated protein 3	-2,34	-3,99	11	57 334 859
4	1452704_at	AK004786	1200015F23Rik	RIKEN cDNA 1200015F23 gene	-2,33	-3,13	2	118 828 441
4	1457812_at	BB119893	Trp53bp1	transformation related protein 53 binding protein 1	-2,10	-3,34	2	120 889 692
4	1442338_at	BB740904			-2,13	-3,17	4	
4	1415694_at	AK004541	Wars	tryptophanyl-tRNA synthetase	-2,01	-5,96	12	109 307 840
4	1444326_at	BB414484	P4hb	prolyl 4-hydroxylase, beta polypeptide	-2,13	-4,14	11	120 376 393
4	1434541_x_at	BF142112	Khdrbs1	KH domain containing, RNA binding, signal transduction associated 1	-2,01	-2,88	4	129 217 162
4	1454898_s_at	AU016407	4833421E05Rik	RIKEN cDNA 4833421E05 gene	-2,32	-4,77	12	21 562 881
4	1430000_at	AK020978	B230117O15Rik	RIKEN cDNA B230117O15 gene	-2,14	-2,78	4	
4	1455773_at	BG797225	Picalm	phosphatidylinositol binding clathrin assembly protein	-2,39	-3,86	7	90 005 423
4	1450109_s_at	NM_013806	Abcc2	ATP-binding cassette, sub-family C (CFTR/MRP), member 2	-2,01	-3,50	19	43 835 714
4	1418469_at	NM_008735	Nrip1	nuclear receptor interacting protein 1	-2,48	-2,85	16	76 173 541
4	1452095_a_at	AK005204	H47	histocompatibility 47	-2,51	-2,96	7	65 958 598
4	1416499_a_at	NM_011722	Dctn6	dynactin 6	-2,48	-3,20	8	35 558 940
4	1419979_s_at	AU044960	Creb3	cAMP responsive element binding protein 3	-2,39	-3,29	4	43 583 733
4	1458426_at	BM941075	Kif1b	kinesin family member 1B	-2,03	-2,86	4	148 077 534
4	1427305_at	AV340508	Piga	phosphatidylinositol glycan anchor biosynthesis, class A	-2,10	-2,60	X	159 763 920
4	1428200_a_at	AK004611	2210010A19Rik	RIKEN cDNA 2210010A19 gene	-2,33	-3,23	16	13 747 507
4	1440305_at	BB546013			-2,25	-3,63	17	
4	1439917_at	AW124545	Pdzd8	PDZ domain containing 8	-2,27	-4,13	19	59 351 677
4	1430079_at	AK020272	5033406O09Rik	RIKEN cDNA 5033406O09 gene	-2,03	-3,19	12	112 390 155
4	1434216_a_at	BG070689	Nudt19	nudix (nucleoside diphosphate linked moiety X)-type motif 19	-2,56	-3,41	7	35 255 946
4	1445697_at	AI594688	Ddef2	development and differentiation enhancing factor 2	-2,19	-3,17	12	21 358 245
4	1416267_at	NM_019708	Scoc	short coiled-coil protein	-2,14	-4,52	8	86 324 599
4	1452272_a_at	BI901126	Gfer	growth factor, erv1 (S. cerevisiae)-like (augmenter of liver regeneration)	-2,46	-3,26	17	24 420 790
4	1458868_at	AV352179	Atp1a1	ATPase, Na+/K+ transporting, alpha 1 polypeptide	-2,45	-3,20	3	101 705 285
4	1457839_at	BB535108	Dhx40	DEAH (Asp-Glu-Ala-His) box polypeptide 40	-2,37	-2,86	11	86 585 948
4	1436061_at	BG092263	Trim34	tripartite motif protein 34	-2,22	-5,43	7	104 118 384
4	1437481_at	AV032349	1110039F03Rik	RIKEN cDNA 1110039F03 gene	-2,43	-3,14	17	
4	1437864_at	BE632137	Adipor2	adiponectin receptor 2	-2,46	-3,50	6	119 318 770
4	1428083_at	AK018202	2310043N10Rik	RIKEN cDNA 2310043N10 gene	-2,15	-5,64	19	
4	1436322_a_at	BE952576	2810001A02Rik	RIKEN cDNA 2810001A02 gene	-2,21	-3,28	15	
4	1441615_at	BB027163	Cbfa2t2h	core-binding factor, runt domain, alpha subunit 2, translocated to, 2 homolog (human)	-2,35	-3,18	2	154 127 958
4	1444195_at	BB386165	Rmnd5a	required for meiotic nuclear division 5 homolog A (S. cerevisiae)	-2,08	-3,73	6	71 320 760
4	1456157_at	AI452290	Mett11d1	methyltransferase 11 domain containing 1	-2,04	-4,41	14	50 806 792
4	1429197_s_at	BB431654	Rabgap1l	RAB GTPase activating protein 1-like	-2,49	-3,34	1	162 055 850
4	1424904_at	BC019143	1300010F03Rik	RIKEN cDNA 1300010F03 gene	-2,35	-3,69	14	
4	1417902_at	NM_054087	Slc19a2	solute carrier family 19 (thiamine transporter), member 2	-2,21	-6,20	1	166 085 729
4	1422748_at	NM_015753	Zfhx1b	zinc finger homeobox 1b	-2,04	-2,60	2	44 805 740
4	1440511_at	BG067940	Psme4	proteasome (prosome, macropain) activator subunit 4	-2,07	-2,66	11	30 672 063
4	1437911_at	BM245142	6330416L07Rik	RIKEN cDNA 6330416L07 gene	-2,07	-2,74	17	22 042 690
4	1451456_at	BC004768	6430706D22Rik	RIKEN cDNA 6430706D22 gene	-2,12	-3,28	1	90 094 279
4	1436907_at	BE980206	Nav1	neuron navigator 1	-2,05	-2,59	1	137 255 283
4	1442643_at	AW912463	Jmjd3	jumonji domain containing 3	-2,06	-3,67	11	69 214 714
4	1456358_at	BM932547	Etv3	ets variant gene 3	-2,10	-3,62	3	87 611 524
4	1457817_at	AI661358	Bcas3	breast carcinoma amplified sequence 3	-2,01	-3,74	11	85 169 408
4	1448005_at	BI658899	Sash1	SAM and SH3 domain containing 1	-2,16	-3,01	10	8 412 408

4	1429502_at	BE533039	Stch	stress 70 protein chaperone, microsome-associated, human homolog	-2,20	-2,64	16	75 637 874
4	1423810_at	BC014867	Ppme1	protein phosphatase methylesterase 1	-2,17	-5,48	7	100 200 820
4	1445968_at	BG074728	Tmtc2	transmembrane and tetratricopeptide repeat containing 2	-2,03	-3,55	10	104 591 773
0	1417936_at	AF128196	Ccl9	chemokine (C-C motif) ligand 9	4,87	-3,09	11	83 389 111
5	1418734_at	U96752	H2Q1	histocompatibility 2, Q region locus 1	-0,66	-10,15	17	34 928 614
5	1428909_at	C85657	A130040M12Rik	RIKEN cDNA A130040M12 gene	-0,49	-6,44	4	
5	1427013_at	AJ245857	Car9	carbonic anhydrase 9	-0,15	-5,63	4	43 528 106
5	1442123_at	AW909226	Tnfsf12	tumor necrosis factor (ligand) superfamily, member 12	-0,41	-8,68	11	69 502 542
5	1429308_at	BF471654	Prdm16	PR domain containing 16	-0,82	-6,50	4	153 164 091
5	1447831_s_at	BB431693	Mtmr7	myotubularin related protein 7	-1,33	-8,13	8	42 048 976
5	1425359_at	BC012256	Mall	mal, T-cell differentiation protein-like	-0,01	-5,45	2	127 395 830
5	1425528_at	L06502	Prrx1	paired related homeobox 1	0,31	-6,91	1	165 091 951
5	1416528_at	NM_080559	Sh3bgrl3	SH3 domain binding glutamic acid-rich protein-like 3	-1,89	-5,43	4	133 399 482
5	1439422_a_at	AV048291	C1qdc2	C1q domain containing 2	-0,70	-6,45	4	154 806 117
5	1436964_at	BB314814	D7Ertd715e	DNA segment, Chr 7, ERATO Doi 715, expressed	-1,99	-5,40	7	59 854 166
5	1460558_at	AV295080	Ccdc32	coiled-coil domain containing 32	-1,96	-5,08	2	118 709 602
5	1434362_at	BM249454	AW550831	expressed sequence AW550831	-0,40	-5,08	1	
5	1455851_at	AV032115	Bmp5	bone morphogenetic protein 5	-0,51	-4,66	9	75 561 273
5	1425668_a_at	AB061305	St3gal4	ST3 beta-galactoside alpha-2,3-sialyltransferase 4	0,53	-4,95	9	34 796 249
5	1422914_at	NM_022435	Sp5	trans-acting transcription factor 5	-0,07	-4,39	2	70 275 761
5	1436890_at	BG963358	Uap1l1	UDP-N-acteylglucosamine pyrophosphorylase 1-like 1	-0,94	-4,34	2	25 183 500
5	1438511_a_at	BB408123	1190002H23Rik	RIKEN cDNA 1190002H23 gene	-0,09	-5,50	14	78 022 905
5	1420641_a_at	AF174535	Sqrdl	sulfide quinone reductase-like (yeast)	1,98	-4,17	2	122 473 439
5	1436857_at	AV066538	Defcr21	defensin related cryptdin 21	-0,65	-4,16	8	22 520 295
5	1459897_a_at	AI507307	Sbsn	suprabasin	-0,74	-3,96	7	30 460 738
5	1416012_at	NM_010119	Ehd1	EH-domain containing 1	-1,78	-5,71	19	6 276 905
5	1433879_a_at	BG073396	C130032J12Rik	RIKEN cDNA C130032J12 gene	-1,69	-4,44	14	46 220 190
5	1434360_s_at	AI596632	Ptprg	protein tyrosine phosphatase, receptor type, G	-1,38	-4,93	14	10 347 278
5	1437871_at	BG967597	Pgm5	phosphoglucomutase 5	0,23	-4,48	19	24 745 357
5	1460232_s_at	NM_013821	Hsd3b6	hydroxy-delta-5-steroid dehydrogenase, 3 beta- and steroid delta-isomerase 6	-1,06	-3,86	3	98 934 565
5	1416046_a_at	BM054266	Fuca2	fucosidase, alpha-L- 2, plasma	1,96	-4,78	10	13 191 225
5	1417294_at	NM_025337	Akr7a5	aldo-keto reductase family 7, member A5 (aflatoxin aldehyde reductase)	0,71	-4,31	4	138 582 819
5	1436334_at	BM232846	Synj1	synaptojanin 1	-1,86	-4,24	16	
5	1453003_at	AK013519	Sorl1	sortilin-related receptor, LDLR class A repeats-containing	-0,10	-3,77	9	41 719 483
5	1416645_a_at	NM_007423	Afp	alpha fetoprotein	-1,71	-4,68	5	91 565 936
5	1422540_at	NM_010180	Fbln1	fibulin 1	-0,09	-4,14	15	85 033 776
5	1442365_at	BI076620	Rtn3	reticulon 3	-1,82	-4,39	19	7 492 944
5	1420566_at	NM_025367	Sphk1	sphingosine kinase 1	0,06	-4,77	11	116 350 829
5	1431293_a_at	AK012260	Cldnd1	claudin domain containing 1	-1,83	-3,76	16	58 670 586
5	1427374_at	AF027131	Muc3	mucin 3, intestinal	0,65	-4,15	5	
5	1445757_at	BB045423	Tbx3	T-box 3	-0,06	-4,10	5	119 931 285
5	1433615_at	BB521146	Tmem117	transmembrane protein 117	0,50	-4,01	15	94 457 364
5	1457477_at	BM119376	Mbnl2	muscleblind-like 2	-0,20	-4,28	14	119 410 850
5	1419658_at	NM_010398	H2T23	histocompatibility 2, T region locus 23	0,24	-5,82	17	35 638 031
5	1449874_at	NM_016923	Ly96	lymphocyte antigen 96	-1,53	-4,72	1	16 673 669
5	1448227_at	NM_010346	Grb7	growth factor receptor bound protein 7	-1,95	-3,27	11	98 262 980
5	1434895_s_at	BG064715	Ppp1r13b	protein phosphatase 1, regulatory (inhibitor) subunit 13B	-1,89	-4,16	12	112 276 265
5	1424077_at	AK016023	Gdpd1	glycerophosphodiester phosphodiesterase domain containing 1	-0,42	-5,47	11	86 850 089
5	1451119_a_at	BC007140	Fbln1	fibulin 1	-0,65	-4,66	15	85 033 776
5	1437918_at	AV374644	4930539E08Rik	RIKEN cDNA 4930539E08 gene	-0,36	-3,85	17	28 623 985
5	1429951_at	AK005150	Ssbp2	single-stranded DNA binding protein 2	-1,10	-4,16	13	91 935 335
5	1444806_at	BB551256	6720456B07Rik	RIKEN cDNA 6720456B07 gene	-0,88	-3,47	6	113 570 548
5	1449113_at	NM_030247	Gpbp1l1	GC-rich promoter binding protein 1-like 1	-1,39	-4,38	4	116 055 658
5	1417066_at	AK014605	Cabc1	chaperone, ABC1 activity of bc1 complex like (S. pombe)	-1,48	-3,93	1	182 001 814
5	1442862_at	BG069698	0610010D24Rik	RIKEN cDNA 0610010D24 gene	0,03	-3,27	12	101 313 751
5	1433586_at	BB612707	Rgmb	RGM domain family, member B	-0,18	-3,51	17	15 510 942
5	1433741_at	BB256012	Cd38	CD38 antigen	-1,49	-3,60	5	44 157 097
5	1460187_at	BI658627	Sfrp1	secreted frizzled-related sequence protein 1	-0,16	-4,53	8	24 877 062
5	1423309_at	AI314055	Tgoln1	trans-golgi network protein	-1,16	-3,77	6	72 537 931
5	1436908_at	BG076129	Pcm1	pericentriolar material 1	-1,00	-3,17	8	42 738 591
5	1427042_at	BB127697	Mal2	mal, T-cell differentiation protein 2	-0,29	-3,73	15	54 401 567

5	1440781_at	BB333095	B830007D08Rik	RIKEN cDNA B830007D08 gene	-1,65	-4,02	9	
5	1433592_at	AW543361	Calm1	calmodulin 1	-1,15	-3,88	12	100 600 590
5	1437595_at	BB531414	E030010A14Rik	RIKEN cDNA E030010A14 gene	0,77	-4,98	19	24 741 104
5	1435562_at	BB795209	Rps12	ribosomal protein S12	-1,60	-3,18	10	23 474 600
5	1435176_a_at	BF019883	Id2	inhibitor of DNA binding 2	0,35	-4,14	12	25 682 880
5	1457901_at	BB244812	D4Ertd76e	DNA segment, Chr 4, ERATO Doi 76, expressed	-1,59	-5,09	4	
5	1442024_at	BB328892	Ppp1r3e	protein phosphatase 1, regulatory (inhibitor) subunit 3E	-1,15	-3,37	14	
5	1421223_a_at	NM_013471	Anxa4	annexin A4	-1,97	-3,43	6	86 702 480
5	1427377_x_at	M77015	Hsd3b3	hydroxy-delta-5-steroid dehydrogenase, 3 beta- and steroid delta-isomerase 3	-1,50	-3,14	3	98 870 584
5	1442920_at	BB128849	Klf3	Kruppel-like factor 3 (basic)	-0,91	-3,05	5	65 082 657
5	1442402_at	BB110728	Sh3rf1	SH3 domain containing ring finger 1	-1,46	-5,02	8	64 116 413
5	1438315_x_at	BB206121	Akr7a5	aldo-keto reductase family 7, member A5 (aflatoxin aldehyde reductase)	0,58	-3,05	4	138 582 819
5	1447173_at	BB704012	E230002P03Rik	RIKEN cDNA E230002P03 gene	-0,02	-4,79	3	?
5	1430317_at	BB279437	Ube2j2	ubiquitin-conjugating enzyme E2, J2 homolog (yeast)	-1,38	-3,54	4	154 787 630
5	1451148_at	AF316872	Pink1	PTEN induced putative kinase 1	-1,48	-3,66	4	137 585 487
5	1438802_at	BB021390	Foxp1	forkhead box P1	-0,87	-3,11	6	98 895 553
5	1431916_at	AK018558	9030618K22Rik	RIKEN cDNA 9030618K22 gene	-0,83	-3,18	3	98 889 683
5	1451243_at	BC019200	Rnpep	arginyl aminopeptidase (aminopeptidase B)	-0,74	-3,54	1	137 079 123
5	1451310_a_at	J02583	Ctsl	cathepsin L	-0,92	-2,95	13	64 377 944
5	1451750_at	BM220651	Irak4	interleukin-1 receptor-associated kinase 4	-0,97	-3,40	15	94 371 899
5	1456849_at	BB427041			-1,70	-3,50	4	
5	1430904_at	AK018680	Arfgap3	ADP-ribosylation factor GTPase activating protein 3	-0,35	-3,58	15	83 127 505
5	1453056_at	AK005699	Slc16a13	solute carrier family 16 (monocarboxylic acid transporters), member 13	-0,44	-3,23	11	70 032 986
5	1417840_at	NM_025892	1500031L02Rik	RIKEN cDNA 1500031L02 gene	-1,34	-4,39	16	32 019 548
5	1446608_at	BB458874	Cbl	Casitas B-lineage lymphoma	-1,19	-4,06	9	43 900 256
5	1445341_at	BG074147	Ywhaq	tyrosine 3-monooxygenase/tryptophan 5-monooxygenase activation protein, theta polypeptide	-1,21	-4,10	12	21 636 820
5	1449614_s_at	AW558560	AI314976	expressed sequence AI314976	-1,35	-3,01	17	47 875 646
5	1424357_at	BC026654	Tmem45b	transmembrane protein 45b	-0,13	-3,97	9	31 175 761
5	1437767_s_at	AA138720	Rbl2	retinoblastoma-like 2	-1,50	-3,33	8	93 960 213
5	1444589_at	AI504688	6330416L07Rik	RIKEN cDNA 6330416L07 gene	-1,97	-3,95	17	22 042 690
5	1426974_at	BG067859	4632413K17Rik	RIKEN cDNA 4632413K17 gene	-1,55	-3,25	10	126 498 600
5	1417394_at	BG069413	Klf4	Kruppel-like factor 4 (gut)	0,34	-3,28	4	55 548 368
5	1447754_x_at	BB130418	Thap4	THAP domain containing 4	-1,30	-3,19	1	95 535 796
5	1426894_s_at	BC023470	C230093N12Rik	RIKEN cDNA C230093N12 gene	-0,34	-3,21	2	32 357 367
5	1439774_at	BB051738	Prrx1	paired related homeobox 1	0,21	-2,77	1	165 091 951
5	1450122_at	NM_008981	Ptprg	protein tyrosine phosphatase, receptor type, G	-1,07	-2,93	14	10 347 278
5	1456947_at	BG072758	Pafah1b1	platelet-activating factor acetylhydrolase, isoform 1b, beta1 subunit	-1,11	-2,97	11	74 490 149
5	1457199_at	BB477101	Sdhc	succinate dehydrogenase complex, subunit C, integral membrane protein	-0,95	-3,38	1	172 965 839
5	1448850_a_at	AK012029	Dnajc5	DnaJ (Hsp40) homolog, subfamily C, member 5	-0,75	-3,54	2	181 449 911
5	1444430_at	BB481523	Armc8	armadillo repeat containing 8	-1,50	-2,70	9	99 289 446
5	1443698_at	BB645745	Fbxo39	F-box protein 39	-1,54	-4,21	11	72 117 859
5	1428369_s_at	BM213829	Arhgap21	Rho GTPase activating protein 21	-1,75	-3,10	2	
5	1443823_s_at	AV325919	Atp1a2	ATPase, Na+/K+ transporting, alpha 2 polypeptide	-0,24	-3,25	1	174 108 384
5	1431805_a_at	AK004849	Rhpn2	rhophilin, Rho GTPase binding protein 2	-1,52	-3,64	7	35 043 083
5	1440320_at	BB149299	Nrf1	nuclear respiratory factor 1	-1,22	-3,13	6	29 997 997
5	1420709_s_at	BC018377	Dao1	D-amino acid oxidase 1	-0,06	-3,04	5	114 264 824
5	1434625_at	BM118654	4930432O21Rik	RIKEN cDNA 4930432O21 gene	-1,43	-3,56	17	21 667 457
5	1425201_a_at	BC024619	Hyi	hydroxypyruvate isomerase homolog (E. coli)	-1,08	-3,29	4	117 857 942
5	1423457_at	BB778934	Slc35a5	solute carrier family 35, member A5	-1,81	-3,19	16	45 058 904
5	1460009_at	BB667115			-0,52	-3,08	1	
5	1436333_a_at	BM232846	Synj1	synaptojanin 1	-1,15	-4,01	16	
5	1422024_at	NM_008026	Fli1	Friend leukemia integration 1	-1,61	-2,69	9	32 171 774
5	1435141_at	AV238378	Sft2d2	SFT2 domain containing 2	-0,43	-2,85	1	167 011 171
5	1455371_at	BB477076	Rfxank	regulatory factor X-associated ankyrin-containing protein	-1,40	-3,09	8	73 059 794
5	1427398_at	AF218265	Muc4	mucin 4	0,09	-3,04	16	32 656 021
5	1434296_at	BI692075	BC049349	cDNA sequence BC049349	-1,89	-3,29	8	74 897 990
5	1450700_at	BB012489	Cdc42ep3	CDC42 effector protein (Rho GTPase binding) 3	-0,66	-3,00	17	79 239 082
5	1431003_a_at	AK003268	2610002J02Rik	RIKEN cDNA 2610002J02 gene	-0,30	-2,60	4	154 093 795
5	1439018_at	AV329790	6330505N24Rik	RIKEN cDNA 6330505N24 gene	-0,29	-3,06	3	84 529 964
5	1457417_at	BB307346	Ctdspl2	CTD (carboxy-terminal domain, RNA polymerase II, polypeptide A) small phosphatase like 2	-1,62	-3,09	2	121 647 893
5	1440584_at	AV377077	9130221L21Rik	RIKEN cDNA 9130221L21 gene	-0,49	-3,56	13	

5	1435399_at	AV092324	2310068J10Rik	RIKEN cDNA 2310068J10 gene	0,49	-2,91	3	123 068 541
5	1431708_a_at	AK009502	Tia1	cytotoxic granule-associated RNA binding protein 1	-1,23	-4,44	6	86 369 947
5	1421670_a_at	NM_029926	Irak4	interleukin-1 receptor-associated kinase 4	-1,81	-4,54	15	94 371 899
5	1442466_a_at	BG071019	Hisppd2a	histidine acid phosphatase domain containing 2A	-1,09	-3,47	2	121 002 003
5	1448613_at	NM_007899	Ecm1	extracellular matrix protein 1	-0,38	-2,78	3	95 819 553
5	1418176_at	AV290079	Vdr	vitamin D receptor	-0,48	-3,16	15	97 682 460
5	1460466_at	BB824055	Srp54	signal recognition particle 54	-0,11	-3,82	12	55 998 584
5	1439535_at	BB204161	Dstn	destrin	-0,32	-3,78	2	143 607 051
5	1443657_at	BB736539	6330505N24Rik	RIKEN cDNA 6330505N24 gene	0,37	-2,72	3	84 529 964
5	1438561_x_at	BB400326	4930538D17Rik	RIKEN cDNA 4930538D17 gene	-0,29	-3,11	19	46 410 245
5	1460629_at	BB033733	Trim16	tripartite motif protein 16	-0,28	-2,81	11	62 636 447
5	1419147_at	NM_020002	Rec8L1	REC8-like 1 (yeast)	0,02	-4,11	14	54 572 240
5	1443078_at	AV321994	6030439D06Rik	RIKEN cDNA 6030439D06 gene	-0,67	-2,78	8	118 573 703
5	1433527_at	BB080732	Ireb2	iron responsive element binding protein 2	0,89	-2,64	9	54 661 925
5	1416646_at	NM_007423	Afp	alpha fetoprotein	-0,36	-3,36	5	91 565 936
5	1439441_x_at	BB134767	Lats2	large tumor suppressor 2	-1,69	-2,72	14	56 643 772
5	1455099_at	BB414982	Mogat2	monoacylglycerol O-acyltransferase 2	0,43	-3,35	7	99 094 277
5	1429277_at	AV063773	LOC632126		0,42	-2,82	17	
5	1429145_at	AK002650	Nhlrc2	NHL repeat containing 2	-0,16	-2,83	19	56 601 510
5	1422854_at	BB753533	Shc1	src homology 2 domain-containing transforming protein C1	-1,91	-3,14	3	89 504 540
5	1425298_a_at	AF135491	Birc1a	baculoviral IAP repeat-containing 1a	0,70	-3,15	13	101 508 022
5	1456320_at	BB701297	BC049806	cDNA sequence BC049806	-1,95	-3,86	1	58 471 801
5	1420936_s_at	BM239527	Cpsf2	cleavage and polyadenylation specific factor 2	-1,73	-3,20	12	102 377 048
5	1437026_at	AV026910	BC057893	cDNA sequence BC057893	-1,69	-2,77	4	46 135 848
5	1436726_s_at	BB470898	Sptlc1	serine palmitoyltransferase, long chain base subunit 1	-1,98	-2,86	13	53 340 285
5	1439727_at	AV375098	Clca6	chloride channel calcium activated 6	0,43	-2,99	3	144 889 871
5	1420673_a_at	NM_053115	Acox2	acyl-Coenzyme A oxidase 2, branched chain	-0,12	-3,77	14	7 019 267
5	1455682_at	BG076081	AA536808	expressed sequence AA536808	-1,94	-3,45	1	
5	1438852_x_at	BB099487	Mcm6	minichromosome maintenance deficient 6 (MIS5 homolog, S. pombe) (S. cerevisiae)	-0,41	-2,99	1	130 159 136
5	1455292_x_at	BE989616	Rsl1	regulator of sex limited protein 1	-1,84	-2,60	13	67 672 278
5	1427851_x_at	AF065324	IghVJ558	immunoglobulin heavy chain (J558 family)	-0,31	-4,21	12	116 205 792
5	1416298_at	NM_013599	Mmp9	matrix metallopeptidase 9	0,21	-2,68	2	164 639 455
5	1436836_x_at	BB724741	Cnn3	calponin 3, acidic	1,04	-3,16	3	121 418 563
5	1439088_at	BB102308	Pdzd8	PDZ domain containing 8	-1,82	-3,57	19	59 351 677
5	1455390_at	BF021286	Alkbh6	alkB, alkylation repair homolog 6 (E. coli)	-1,55	-3,02	7	30 017 512
5	1442442_at	AI507322	Thoc7	THO complex 7 homolog (Drosophila)	-1,08	-3,27	14	12 744 207
5	1429215_at	BQ175646	2310058N22Rik	RIKEN cDNA 2310058N22 gene	-1,79	-2,61	12	116 821 230
5	1437359_at	BI793607	Rnps1	ribonucleic acid binding protein S1	-1,46	-3,03	17	24 142 692
5	1425099_a_at	BC011080	Arntl	aryl hydrocarbon receptor nuclear translocator-like	-1,58	-2,62	7	112 998 645
5	1427183_at	BC023060	Efemp1	epidermal growth factor-containing fibulin-like extracellular matrix protein 1	-0,13	-2,77	11	28 767 772
5	1439884_at	AI851783	Nudt16	nudix (nucleoside diphosphate linked moiety X)-type motif 16	-1,40	-3,04	9	104 987 772
5	1423204_at	BB367487	Tm9sf4	transmembrane 9 superfamily protein member 4	-1,58	-3,06	2	152 852 743
5	1424745_at	BC024461	2900006B13Rik	RIKEN cDNA 2900006B13 gene	-1,56	-3,26	11	51 428 225
5	1448789_at	AF253409	Aldh1a3	aldehyde dehydrogenase family 1, subfamily A3	0,15	-2,86	7	66 269 846
5	1434663_at	BI153133	2410129H14Rik	RIKEN cDNA 2410129H14 gene	-0,49	-2,77	14	97 917 683
5	1451536_at	AK009195	Mtfr1	mitochondrial fission regulator 1	-1,68	-2,99	3	19 380 394
5	1455901_at	AI642069	Chpt1	choline phosphotransferase 1	-0,09	-3,47	10	87 903 332
5	1417841_at	AF309644	Pxmp2	peroxisomal membrane protein 2	-1,68	-3,15	5	110 514 589
5	1438435_at	BB329313	Phca	phytoceramidase, alkaline	-0,78	-3,05	7	98 088 853
5	1433685_a_at	BM248225	6430706D22Rik	RIKEN cDNA 6430706D22 gene	-1,92	-2,70	1	90 094 279
5	1442928_at	AI481757	Kpna4	karyopherin (importin) alpha 4	-1,48	-3,73	3	69 160 149
5	1453330_at	AK002458	0610010D24Rik	RIKEN cDNA 0610010D24 gene	-0,06	-3,09	12	101 313 751
5	1427406_at	AV013785	Trip11	thyroid hormone receptor interactor 11	-1,23	-3,60	12	102 235 058
5	1418635_at	BI456953	Etv3	ets variant gene 3	-1,19	-2,96	3	87 611 524
5	1451253_at	BC016131	Pxk	PX domain containing serine/threonine kinase	-0,72	-2,72	14	6 890 121
5	1429555_at	AA408371	Cldnd1	claudin domain containing 1	-1,13	-2,59	16	58 670 586
5	1440252_at	AI849211	H2afj	H2A histone family, member J	-1,89	-3,44	6	136 772 519
5	1460652_at	NM_007953	Esrra	estrogen related receptor, alpha	-0,43	-2,68	19	6 978 021
5	1423819_s_at	AF133669	Arl6ip1	ADP-ribosylation factor-like 6 interacting protein 1	-1,23	-3,40	7	117 910 048
5	1440974_at	BB242458	9030203C11Rik	RIKEN cDNA 9030203C11 gene	-0,52	-2,72	10	
5	1439988_at	BG066975	Mrpl15	mitochondrial ribosomal protein L15	-0,70	-2,74	1	4 763 291

5	1457619_at	BB743970	Ces6	carboxylesterase 6	-0,88	-2,62	8	107 623 136
5	1437126_at	BB115513	Immt	inner membrane protein, mitochondrial	-1,26	-3,13	6	71 760 839
5	1456310_a_at	AV218922	2610002J02Rik	RIKEN cDNA 2610002J02 gene	-1,53	-3,07	4	154 093 795
5	1440493_at	BB399121	Galnt10	UDP-N-acetyl-alpha-D-galactosamine:polypeptide N-acetylgalactosaminyltransferase 10	-0,43	-3,33	11	57 461 636
5	1437274_at	BM204808	Copa	coatomer protein complex subunit alpha	-0,81	-3,76	1	173 919 477
5	1458460_at	BB030901	Stim1	stromal interaction molecule 1	-0,66	-2,68	7	102 141 653
5	1432198_at	AK018172	A230083H22Rik	RIKEN cDNA A230083H22 gene	-1,08	-4,13	19	
5	1426724_at	AI314104	Cnn3	calponin 3, acidic	1,40	-3,12	3	121 418 563
5	1457147_at	BB557242	BC026657	cDNA sequence BC026657	-1,28	-3,60	2	
5	1460203_at	NM_010585	Itpr1	inositol 1,4,5-triphosphate receptor 1	0,70	-2,69	6	108 178 894
5	1423223_a_at	BB796358	Prdx6	peroxiredoxin 6	1,23	-3,06	1	163 076 788
5	1418175_at	AV290079	Vdr	vitamin D receptor	-0,06	-2,95	15	97 682 460
5	1439079_a_at	BM240030	Erbb2ip	Erbb2 interacting protein	-0,74	-3,45	13	104 939 168
5	1424861_at	BM200153	Zbtb24	zinc finger and BTB domain containing 24	-1,48	-2,74	10	41 138 810
5	1422153_a_at	NM_026853	Asb11	ankyrin repeat and SOCS box-containing protein 11	0,24	-2,72	X	159 782 205
5	1417884_at	NM_134038	Slc16a6	solute carrier family 16 (monocarboxylic acid transporters), member 6	-0,21	-2,76	11	109 266 947
5	1415949_at	BC010197	Cpe	carboxypeptidase E	0,60	-3,18	8	67 484 747
5	1417644_at	BC021484	Sspn	sarcospan	-1,08	-3,51	6	145 891 305
5	1442952_at	BG066106	Shroom3	shroom family member 3	-0,38	-3,07	5	93 758 638
5	1433976_at	BI249740	D10Ucla1	DNA segment, Chr 10, University of California at Los Angeles 1	0,83	-2,65	10	66 406 864
5	1448244_at	BC013536	Lypla1	lysophospholipase 1	-0,06	-3,38	1	4 797 973
5	1418072_at	NM_023422	Hist1h2bc	histone 1, H2bc	-1,53	-2,60	13	23 691 663
5	1433271_at	AK020543	9530004M16Rik	RIKEN cDNA 9530004M16 gene	-1,55	-2,66	4	
5	1419309_at	NM_010329	Pdpn	podoplanin	-0,40	-3,16	4	142 534 132
5	1443517_at	BG061911	6030443O07Rik	RIKEN cDNA 6030443O07 gene	-1,58	-2,76	19	
5	1439278_at	BB175650	Zbtb20	zinc finger and BTB domain containing 20	-1,37	-2,86	16	43 429 639
5	1425127_at	BC026757	Hsd3b2	hydroxy-delta-5-steroid dehydrogenase, 3 beta- and steroid delta-isomerase 2	0,45	-3,36	3	98 838 322
5	1435693_at	AV378589	Mall	mal, T-cell differentiation protein-like	-0,23	-2,63	2	127 395 830
5	1424784_at	AV047635	6330416L07Rik	RIKEN cDNA 6330416L07 gene	-1,53	-2,99	17	22 042 690
5	1441339_at	BG230324	Chd9	chromodomain helicase DNA binding protein 9	-1,84	-2,72	8	93 718 941
5	1417393_a_at	NM_026125	C1qdc2	C1q domain containing 2	-0,59	-3,18	4	154 806 117
5	1445963_at	BG076280	Pde5a	phosphodiesterase 5A, cGMP-specific	0,41	-2,64	3	122 721 208
5	1418113_at	BC010989	Cyp2d10	cytochrome P450, family 2, subfamily d, polypeptide 10	-0,81	-2,60	15	82 230 609
5	1457751_at	BB371102	4832420A03Rik	RIKEN cDNA 4832420A03 gene	-1,73	-2,63	7	97 564 445
5	1428850_x_at	AK004342	Cd99	CD99 antigen	-1,03	-2,73	4	
5	1438726_at	BB533836	Mical2	microtubule associated monoxygenase, calponin and LIM domain containing 2	-0,38	-2,67	7	112 017 070
5	1435446_a_at	BF180212	Chpt1	choline phosphotransferase 1	-0,45	-2,58	10	87 903 332
5	1459740_s_at	AW108044	Ucp2	uncoupling protein 2 (mitochondrial, proton carrier)	1,83	-2,75	7	100 367 445
5	1442693_at	BB271468	LOC552905		-0,50	-3,34	7	104 137 208
5	1456070_at	AI507538	5430405N12Rik	RIKEN cDNA 5430405N12 gene	-1,98	-3,14	14	
5	1422716_a_at	AW554436	Acp1	acid phosphatase 1, soluble	-1,74	-3,65	12	31 479 377
5	1450165_at	NM_011408	Slfn2	schlafen 2	-1,51	-2,85	11	82 881 306
5	1431339_a_at	AK007560	Efhd2	EF hand domain containing 2	-1,74	-2,83	4	141 130 217
5	1448416_at	NM_008597	Mgp	matrix Gla protein	-0,56	-2,58	6	136 836 631
5	1449106_at	NM_008161	Gpx3	glutathione peroxidase 3	-1,18	-2,59	11	54 746 372
5	1441362_at	AW493584	Ptprg	protein tyrosine phosphatase, receptor type, G	-1,87	-3,05	14	10 347 278
5	1426436_at	BG066131	Tmem159	transmembrane protein 159	-0,81	-2,58	7	119 893 574
5	1421037_at	BG070037	Npas2	neuronal PAS domain protein 2	0,20	-3,17	1	39 138 757
5	1456050_at	BG066927	Rock2	Rho-associated coiled-coil containing protein kinase 2	-0,27	-2,73	12	16 920 669
5	1441200_at	BB327909	Klf3	Kruppel-like factor 3 (basic)	-1,59	-2,64	5	65 082 657
5	1453556_x_at	AK002762	Cd99	CD99 antigen	-1,59	-3,10	4	
5	1457275_at	AI594683	Dmn	desmuslin	-0,28	-2,81	7	67 603 680
5	1417797_a_at	NM_133707	1810019J16Rik	RIKEN cDNA 1810019J16 gene	-1,50	-2,68	4	132 791 110
5	1448687_at	NM_026125	C1qdc2	C1q domain containing 2	-0,41	-3,18	4	154 806 117
5	1425526_a_at	L06502	Prrx1	paired related homeobox 1	0,15	-2,73	1	165 091 951
5	1444260_at	BF661746			-0,29	-2,69	8	72 245 685
5	1455503_at	AW107826	Zfp273	zinc finger protein 273	-1,67	-2,58	13	68 312 918
5	1438221_at	AI875682	C130065N10Rik	RIKEN cDNA C130065N10 gene	0,34	-2,86	1	
5	1436879_x_at	AV124668	Afp	alpha fetoprotein	-0,94	-2,60	5	91 565 936
5	1447227_at	AI504711	Slc40a1	solute carrier family 40 (iron-regulated transporter), member 1	-0,02	-2,80	1	45 852 629
5	1440416_at	BB150699	Usp46	ubiquitin specific peptidase 46	-1,60	-2,68	5	74 281 735

5	1426221_at	BC004727	Loh11cr2a	loss of heterozygosity, 11, chromosomal region 2, gene A homolog (human)	-1,32	-2,65	9	38 468 868
5	1441281_s_at	AU024536	Ninj1	ninjurin 1	0,72	-2,58	13	49 199 483
5	1431218_at	BB655693	Zdhhc20	zinc finger, DHHC domain containing 20	-1,05	-2,82	14	56 786 812
5	1430414_at	BB524769	1300010F03Rik	RIKEN cDNA 1300010F03 gene	-1,14	-2,58	14	
5	1431688_at	AK014661	LOC73899		0,25	-3,33	19	
5	1456478_at	BB183423	Pgm2l1	phosphoglucomutase 2-like 1	-1,38	-2,73	7	100 101 706
5	1418739_at	NM_013731	Sgk2	serum/glucocorticoid regulated kinase 2	1,66	-2,62	2	162 678 986
5	1420287_at	R75193			1,28	-2,69	8	87 305 126
5	1435330_at	BM241008	AI447904	expressed sequence AI447904	-1,62	-2,96	1	175 467 542
5	1438763_at	BB016751	Dnahc2	dynein, axonemal, heavy chain 2	0,27	-2,71	11	69 237 006
5	1456471_x_at	BB204486	Phgdh	3-phosphoglycerate dehydrogenase	0,43	-2,62	3	98 398 575
5	1445387_at	BM118663	Senp6	SUMO/sentrin specific peptidase 6	-1,68	-2,90	9	79 852 586
5	1422211_a_at	AY037785	B3gnt3	UDP-GlcNAc:betaGal beta-1,3-N-acetylglucosaminyltransferase 3	-0,55	-2,59	8	74 620 710
5	1434424_at	BB276950	9630055N22Rik	RIKEN cDNA 9630055N22 gene	0,95	-2,90	1	
5	1431380_at	BB662587	Ica1	islet cell autoantigen 1	-0,18	-2,78	6	8 580 527
5	1435449_at	BM120925	Bcl2l11	BCL2-like 11 (apoptosis facilitator)	-1,89	-2,91	2	127 817 478
5	1439630_x_at	AI844734	Sbsn	suprabasin	-0,21	-2,80	7	30 460 738
5	1428418_s_at	BE198251	3110050N22Rik	RIKEN cDNA 3110050N22 gene	-1,55	-2,58	3	7 486 534
5	1456789_at	AW491540	Zfp462	zinc finger protein 462	-0,20	-2,69	4	55 041 276
5	1442183_at	AW555202	LOC236069		-1,41	-2,65	4	
5	1430999_a_at	BM932452	Scoc	short coiled-coil protein	-0,88	-2,66	8	86 324 599
5	1436054_at	BM220028	9130227C08Rik	RIKEN cDNA 9130227C08Rik gene	-1,66	-2,67	14	54 839 039
5	1421283_at	NM_007555	Bmp5	bone morphogenetic protein 5	-0,51	-2,88	9	75 561 273
5	1434725_at	AV255657	Gramd1c	GRAM domain containing 1C	-1,79	-2,78	16	43 899 688
5	1444205_at	BB663238	Smad4	MAD homolog 4 (Drosophila)	-1,30	-2,68	18	73 764 382
5	1445104_at	BB366803	E230029C05Rik	RIKEN cDNA E230029C05 gene	-0,96	-2,64	7	89 914 687
6	1440921_at	AI527293	Nalp12	NACHT, LRR and PYD containing protein 12	-10,93	0,64	7	?
6	1424105_a_at	AF069051	Pttg1	pituitary tumor-transforming 1	-9,30	0,97	11	43 263 687
6	1425869_a_at	BC010403	Psen2	presenilin 2	-8,82	-0,43	1	182 063 680
6	1438390_s_at	AV105428	Pttg1	pituitary tumor-transforming 1	-8,95	-1,81	11	43 263 687
6	1441430_at	BQ174667			-8,21	0,06	3	130 626 163
6	1418858_at	NM_023617	Aox3	aldehyde oxidase 3	-7,09	-0,68	1	58 057 681
6	1419407_at	NM_010406	Hc	hemolytic complement	-7,59	-0,54	2	34 805 339
6	1437040_at	AV280878	Etnk2	ethanolamine kinase 2	-7,21	0,47	1	135 191 334
6	1459141_at	BB667838	1810008I18Rik	RIKEN cDNA 1810008I18 gene	-6,70	-0,09	7	65 684 687
6	1416178_a_at	NM_013746	Plekhb1	pleckstrin homology domain containing, family B (evectins) member 1	-6,51	-0,75	7	100 517 984
6	1424576_s_at	BC025819	Cyp2c44	cytochrome P450, family 2, subfamily c, polypeptide 44	-5,69	-0,33	19	44 058 336
6	1451239_a_at	BC022130	Slc26a1	solute carrier family 26 (sulfate transporter), member 1	-6,14	-0,22	5	108 910 185
6	1449836_x_at	NM_007546	Bik	Bcl2-interacting killer	-5,76	-0,59	15	83 354 637
6	1418288_at	NM_015763	Lpin1	lipin 1	-6,28	-0,69	12	16 562 115
6	1436221_at	BG067625	D1Ertd471e	DNA segment, Chr 1, ERATO Doi 471, expressed	-4,76	-0,19	1	
6	1435217_at	BG066901	LOC666185		-6,41	-0,45	13	
6	1423397_at	AI118428	Ugt2b36	UDP glucuronosyltransferase 2 family, polypeptide B36	-4,89	1,43	5	88 140 487
6	1418250_at	NM_025404	Arfl4	ADP-ribosylation factor 4-like	-5,72	-0,76	11	101 481 650
6	1421092_at	AK014346	Serpina12	serine (or cysteine) peptidase inhibitor, clade A (alpha-1 antiproteinase, antitrypsin), member 12	-5,35	-0,51	12	104 429 818
6	1457324_at	BM115786	Oprs1	opioid receptor, sigma 1	-5,95	-0,65	4	41 927 006
6	1426516_a_at	AK014526	Lpin1	lipin 1	-5,46	-1,87	12	16 562 115
6	1452349_x_at	AI481797	Ifi205	interferon activated gene 205	-5,62	-0,08	1	175 848 673
6	1438759_x_at	AU046270			-5,53	-1,19	?	
6	1420983_at	AF114437	Pctp	phosphatidylcholine transfer protein	-5,10	-1,28	11	89 800 681
6	1455240_x_at	BG066901	LOC666185		-4,89	0,03	13	
6	1457724_at	AV023994	Ctsl	cathepsin L	-5,94	-1,08	13	64 377 944
6	1435579_at	BM220110	4933409K07Rik	RIKEN cDNA 4933409K07 gene	-4,59	-0,72	4	42 199 016
6	1442124_at	BG797231	AI450326	expressed sequence AI450326	-4,28	-1,33	4	
6	1422513_at	NM_007634	Ccnf	cyclin F	-5,69	0,53	17	23 950 148
6	1434348_at	BM206792	Fez2	fasciculation and elongation protein zeta 2 (zygin II)	-4,39	-0,12	17	78 282 943
6	1454777_at	BB553107	Slco2b1	solute carrier organic anion transporter family, member 2b1	-4,87	-0,89	7	99 531 886
6	1428832_at	AK005395	1600002H07Rik	RIKEN cDNA 1600002H07 gene	-4,30	0,07	17	23 942 655
6	1449635_at	AA409562	Prpf19	PRP19/PSO4 pre-mRNA processing factor 19 homolog (S. cerevisiae)	-4,92	-1,79	19	10 962 336
6	1442406_at	BE852666	9230104K21Rik	RIKEN cDNA 9230104K21 gene	-5,70	-1,01	4	
6	1437308_s_at	AV024285	F2r	coagulation factor II (thrombin) receptor	-4,59	1,02	13	96 702 487

6	1454995_at	AW556888	Ddah1	dimethylarginine dimethylaminohydrolase 1	-5,65	-0,77	3	145 696 112
6	1449740_s_at	C79957	Dsg2	desmoglein 2	-4,81	-0,73	18	20 701 125
6	1457141_at	BB229969	Aqp11	aquaporin 11	-4,66	-1,70	7	97 601 570
6	1438391_x_at	AV078914	Hadh2	hydroxyacyl-Coenzyme A dehydrogenase type II	-4,61	-0,34	X	147 342 611
6	1433933_s_at	BB553107	Slco2b1	solute carrier organic anion transporter family, member 2b1	-5,11	-1,78	7	99 531 886
6	1449419_at	NM_028785	Dock8	dedicator of cytokinesis 8	-4,69	0,19	19	25 066 625
6	1439476_at	BG092030	Dsg2	desmoglein 2	-4,17	-0,18	18	20 701 125
6	1416250_at	NM_007570	Btg2	B-cell translocation gene 2, anti-proliferative	-4,52	1,40	1	135 891 275
6	1420688_a_at	NM_011360	Sgce	sarcoglycan, epsilon	-4,03	-0,57	6	4 624 351
6	1436931_at	AV255458	Rfx4	regulatory factor X, 4 (influences HLA class II expression)	-4,05	-0,46	10	84 185 846
6	1424811_at	BC024605	Cml5	camello-like 5	-4,40	-0,86	6	85 782 877
6	1420362_a_at	NM_007546	Bik	Bcl2-interacting killer	-5,82	-0,96	15	83 354 637
6	1448286_at	NM_016763	Hadh2	hydroxyacyl-Coenzyme A dehydrogenase type II	-4,04	0,75	X	147 342 611
6	1457904_at	BB125515	Car8	carbonic anhydrase 8	-3,77	-1,32	4	8 068 640
6	1423554_at	AV370848	Ggcx	gamma-glutamyl carboxylase	-5,15	-1,80	6	72 343 841
6	1437892_at	BQ084812	Zfp306	zinc finger protein 306	-4,70	-0,87	13	21 394 560
6	1439015_at	AV221299	Gfra1	glial cell line derived neurotrophic factor family receptor alpha 1	-4,39	-0,94	19	58 289 941
6	1427213_at	X98848	Pfkfb1	6-phosphofructo-2-kinase/fructose-2,6-biphosphatase 1	-3,97	0,45	X	145 930 636
6	1429254_at	BF472491	Aqp11	aquaporin 11	-4,72	0,31	7	97 601 570
6	1435458_at	AI323550	Pim1	proviral integration site 1	-4,36	-0,72	17	29 217 823
6	1425619_s_at	AB072269	Dsg2	desmoglein 2	-4,34	-0,91	18	20 701 125
6	1427370_at	AK005066	Amdhd1	amidohydrolase domain containing 1	-4,81	-0,14	10	
6	1416665_at	AF098949	Coq7	demethyl-Q 7	-5,26	-0,13	7	118 316 211
6	1451006_at	AV286265	Xdh	xanthine dehydrogenase	-3,43	-0,73	17	73 788 789
6	1418536_at	M29881	H2Q8	histocompatibility 2, Q region locus 8	-3,67	-1,49	17	35 002 151
6	1440346_at	BG228765	Jmjd3	jumonji domain containing 3	-4,60	-1,53	11	69 214 714
6	1441946_at	AV239969	Itih5	inter-alpha (globulin) inhibitor H5	-3,49	0,12	2	10 071 468
6	1423666_s_at	BM114165	Rpl5	ribosomal protein L5	-4,55	-1,93	5	108 140 891
6	1449523_at	NM_009746	Bcl7c	B-cell CLL/lymphoma 7C	-4,80	-0,63	7	127 496 127
6	1420624_a_at	NM_016794	Vamp8	vesicle-associated membrane protein 8	-4,96	-1,73	6	72 314 732
6	1428267_at	AK010512	Dhx40	DEAH (Asp-Glu-Ala-His) box polypeptide 40	-4,53	-1,76	11	86 585 948
6	1437629_at	AV321315	Arhgef19	Rho guanine nucleotide exchange factor (GEF) 19	-4,15	-1,10	4	140 514 959
6	1417421_at	BC005590	S100a1	S100 calcium binding protein A1	-3,81	-0,79	3	90 596 964
6	1439631_at	BG060248	Zcchc11	zinc finger, CCHC domain containing 11	-4,13	-1,97	4	107 957 357
6	1449410_a_at	NM_013525	Gas5	growth arrest specific 5	-4,59	-0,50	1	
6	1426008_a_at	M62838	Slc7a2	solute carrier family 7 (cationic amino acid transporter, y+ system), member 2	-4,12	-0,60	8	42 361 228
6	1427590_at	BC011182	Zfp39	zinc finger protein 39	-3,16	0,26	11	58 704 347
6	1416408_at	AB034914	Acox1	acyl-Coenzyme A oxidase 1, palmitoyl	-4,27	-1,86	11	115 987 978
6	1453070_at	BB305930	Pcdh17	protocadherin 17	-3,61	-1,46	14	83 253 697
6	1436503_at	BF302511	BC048546	cDNA sequence BC048546	-4,13	-1,19	6	128 505 900
6	1443056_at	BB374993	Ptprd	protein tyrosine phosphatase, receptor type, D	-4,48	-1,48	4	75 412 469
6	1434510_at	BF780807	Papss2	3'-phosphoadenosine 5'-phosphosulfate synthase 2	-3,89	-0,89	19	32 686 001
6	1423686_a_at	BC016234	Prr13	proline rich 13	-3,03	-1,65	15	102 287 265
6	1449844_at	AB031813	Slco1a1	solute carrier organic anion transporter family, member 1a1	-3,94	0,93	6	141 870 258
6	1429159_at	AK018605	4631408O11Rik	RIKEN cDNA 4631408O11 gene	-4,19	-0,17	2	
6	1448564_at	BC003714	Cib1	calcium and integrin binding 1 (calmyrin)	-3,61	0,15	7	80 100 672
6	1426502_s_at	AK008086	Gpt1	glutamic pyruvic transaminase 1, soluble	-2,99	-0,17	15	76 524 018
6	1418249_at	NM_007761	Crcp	calcitonin gene-related peptide-receptor component protein	-3,31	0,21	5	130 314 008
6	1428468_at	AK014175	3110043O21Rik	RIKEN cDNA 3110043O21 gene	-3,66	-1,30	4	
6	1448034_at	AA215276	AI842396	expressed sequence AI842396	-4,28	0,09	11	70 504 555
6	1456036_x_at	AV003026	Gsto1	glutathione S-transferase omega 1	-3,40	-1,50	19	47 908 299
6	1449685_s_at	C80494	Oxsm	3-oxoacyl-ACP synthase, mitochondrial	-3,46	-1,69	14	15 031 925
6	1427282_a_at	AV007132	Fxn	frataxin	-3,09	0,19	19	24 328 549
6	1435665_at	BM241342	AI451617	expressed sequence AI451617	-3,67	-1,75	7	104 345 671
6	1422975_at	NM_008604	Mme	membrane metallo endopeptidase	-4,29	0,00	3	63 383 800
6	1431240_at	AK017207	Clec2h	C-type lectin domain family 2, member h	-4,47	0,75	6	128 628 004
6	1423181_s_at	AK011789	Clns1a	chloride channel, nucleotide-sensitive, 1A	-3,81	-1,75	7	97 571 882
6	1448350_at	NM_133768	Asl	argininosuccinate lyase	-4,40	0,40	5	130 295 966
6	1422648_at	BF533509	Slc7a2	solute carrier family 7 (cationic amino acid transporter, y+ system), member 2	-3,39	-1,01	8	42 361 228
6	1418254_at	NM_019735	Apip	APAF1 interacting protein	-3,83	1,58	2	102 874 513
6	1446461_at	BB462504	Sox5	SRY-box containing gene 5	-4,07	-0,08	6	143 790 050

6	1459635_at	BG144294	Dlgh1	discs, large homolog 1 (Drosophila)	-3,01	-0,95	16	31 583 785
6	1460380_at	AB072269	Dsg2	desmoglein 2	-4,09	-0,74	18	20 701 125
6	1426389_at	BG071931	Camk1d	calcium/calmodulin-dependent protein kinase ID	-3,35	2,06	2	5 210 729
6	1436935_x_at	BB168483	Clns1a	chloride channel, nucleotide-sensitive, 1A	-3,99	-1,47	7	97 571 882
6	1419257_at	BC006022	Tcea1	transcription elongation factor A (SII) 1	-4,48	-1,89	1	4 847 894
6	1439517_at	BB660695	Mysm1	myb-like, SWIRM and MPN domains 1	-4,50	-1,78	4	94 434 058
6	1428229_at	BF160591	Prkcn	protein kinase C, nu	-3,14	-1,02	17	78 855 612
6	1454880_s_at	BB212341	Bmf	Bcl2 modifying factor	-2,91	-0,85	2	118 220 198
6	1429189_at	BI440651	1110007C02Rik	RIKEN cDNA 1110007C02 gene	-3,84	-1,96	13	95 042 273
6	1449342_at	NM_023587	Ptplb	protein tyrosine phosphatase-like (proline instead of catalytic arginine), member b	-3,92	-0,98	16	34 942 167
6	1435559_at	BB200233	Myo6	myosin VI	-3,19	-1,78	9	79 950 723
6	1457486_at	BM120811	D9Ucla1	DNA segment, Chr 9, University of California at Los Angeles 1	-3,51	0,81	9	42 972 274
6	1431817_at	AK004863	Adh6ps1	alcohol dehydrogenase 6 (class V), pseudogene 1	-3,50	0,11	3	138 311 508
6	1450852_s_at	BQ173958	F2r	coagulation factor II (thrombin) receptor	-3,72	0,91	13	96 702 487
6	1432517_a_at	AK006371	Nnmt	nicotinamide N-methyltransferase	-2,90	-0,73	9	48 343 843
6	1427011_a_at	AJ294535	Lancl1	LanC (bacterial lantibiotic synthetase component C)-like 1	-4,17	-0,49	1	66 933 724
6	1424373_at	AK004598	Armcx3	armadillo repeat containing, X-linked 3	-2,93	-0,06	X	130 102 962
6	1448622_at	NM_015816	Lsm4	LSM4 homolog, U6 small nuclear RNA associated (S. cerevisiae)	-3,96	-0,68	8	73 602 380
6	1424676_s_at	BC026948	Sec14l4	SEC14-like 4 (S. cerevisiae)	-3,80	0,59	11	3 931 784
6	1438385_s_at	BB068040	Gpt2	glutamic pyruvate transaminase (alanine aminotransferase) 2	-4,13	-1,76	8	88 382 721
6	1416104_at	NM_011900	Mpdu1	mannose-P-dolichol utilization defect 1	-4,17	-1,68	11	69 472 898
6	1455961_at	AV174022			-3,44	0,50	3	63 470 872
6	1438055_at	BB035017	Rarres1	retinoic acid receptor responder (tazarotene induced) 1	-3,79	0,14	3	67 566 815
6	1435742_at	AV028026	1110034C04Rik	RIKEN cDNA 1110034C04 gene	-3,81	-1,84	12	101 440 569
6	1448239_at	NM_010442	Hmox1	heme oxygenase (decycling) 1	-3,66	-0,48	8	77 989 693
6	1429720_at	AK021042	Mak10	MAK10 homolog, amino-acid N-acetyltransferase subunit, (S. cerevisiae)	-2,95	1,58	13	59 595 033
6	1416381_a_at	BC008174	Prdx5	peroxiredoxin 5	-3,11	-1,82	19	6 973 862
6	1437874_s_at	AV225808	Hexb	hexosaminidase B	-2,88	1,03	13	98 277 106
6	1425137_a_at	BC011215	H2Q10	histocompatibility 2, Q region locus 10	-3,23	-1,02	17	35 078 159
6	1425823_at	BC026782	BC026782	cDNA sequence BC026782	-4,20	-0,43	1	141 626 703
6	1429723_at	AK018153	6330409N04Rik	RIKEN cDNA 6330409N04 gene	-4,02	-0,84	14	60 488 414
6	1450702_at	AJ306425	Hfe	hemochromatosis	-3,16	-0,82	13	23 711 307
6	1455508_at	BF318246	A530082C11Rik	RIKEN cDNA A530082C11 gene	-3,29	-0,45	4	154 445 215
6	1420363_at	NM_007546	Bik	Bcl2-interacting killer	-4,26	-0,12	15	83 354 637
6	1424673_at	AF350410	Clec2h	C-type lectin domain family 2, member h	-4,28	0,29	6	128 628 004
6	1441926_x_at	BB326566	Tmie	transmembrane inner ear	-3,78	-1,48	9	110 710 862
6	1419196_at	NM_032541	Hamp1	hepcidin antimicrobial peptide 1	-3,57	-0,08	7	30 651 130
6	1436293_x_at	AI852300	D1Ertd471e	DNA segment, Chr 1, ERATO Doi 471, expressed	-3,76	-0,69	1	
6	1416726_s_at	NM_133777	Ube2s	ubiquitin-conjugating enzyme E2S	-3,32	0,13	7	4 411 102
6	1439399_a_at	BB493265	Snord22	small nucleolar RNA, C/D box 22	-3,21	-1,93	19	
6	1450738_at	NM_016705	Kif21a	kinesin family member 21A	-3,78	-1,65	15	90 762 149
6	1434043_a_at	AV286809	Repin1	replication initiator 1	-3,26	-0,75	6	48 523 525
6	1438081_at	BB794635	Mcc	mutated in colorectal cancers	-4,07	-0,67	18	44 550 391
6	1417146_at	NM_026423	2410018C20Rik	RIKEN cDNA 2410018C20 gene	-4,06	-1,74	8	87 140 010
6	1460241_a_at	BB829192	St3gal5	ST3 beta-galactoside alpha-2,3-sialyltransferase 5	-3,16	-0,17	6	72 027 121
6	1424245_at	BC015290	Ces2	carboxylesterase 2	-2,94	0,15	8	107 736 214
6	1442408_at	BE981170	2010305B15Rik	RIKEN cDNA 2010305B15 gene	-3,69	-1,21	2	
6	1436539_at	BQ266254	Clmn	calmin	-3,88	-1,04	12	105 164 164
6	1416149_at	AB038696	Olig1	oligodendrocyte transcription factor 1	-3,67	-0,18	16	91 158 627
6	1458092_at	BM232899	Ap3m1	adaptor-related protein complex 3, mu 1 subunit	-3,54	-1,58	14	19 823 361
6	1450958_at	BQ177170	Tm4sf1	transmembrane 4 superfamily member 1	-3,95	0,01	3	57 374 992
6	1453023_at	AK003441	Ankhd1	ankyrin repeat and KH domain containing 1	-3,32	-0,31	18	
6	1444377_at	BM201103	Psmb2	proteasome (prosome, macropain) subunit, beta type 2	-2,79	-0,90	4	126 179 969
6	1429144_at	AV291259	Prei4	preimplantation protein 4	-3,74	0,51	2	132 220 524
6	1428381_a_at	BG868450	2700038C09Rik	RIKEN cDNA 2700038C09 gene	-3,52	-0,14	2	181 116 749
6	1437953_at	BM246706	Prei4	preimplantation protein 4	-3,32	0,75	2	132 220 524
6	1434082_at	BM243464	Pctk2	PCTAIRE-motif protein kinase 2	-3,34	-0,88	10	92 638 098
6	1418327_at	NM_026503	1110058L19Rik	RIKEN cDNA 1110058L19 gene	-3,04	-0,73	1	23 950 220
6	1438404_at	BB125272	Rnf144	ring finger protein 144	-2,86	-1,02	12	26 893 339
6	1421921_at	BC011158	Serpina3m	serine (or cysteine) peptidase inhibitor, clade A, member 3M	-3,26	0,60	12	104 788 241
6	1458327_x_at	BB275387	Slc26a1	solute carrier family 26 (sulfate transporter), member 1	-2,95	0,21	5	108 910 185

6	1421500_at	NM_009293	Sts	steroid sulfatase	-3,95	0,03	X, Y	
6	1420524_a_at	NM_010767	Masp2	mannan-binding lectin serine peptidase 2	-2,60	0,26	4	147 446 343
6	1450440_at	BE534815	Gfra1	glial cell line derived neurotrophic factor family receptor alpha 1	-3,61	-0,63	19	58 289 941
6	1450899_at	BB829652	Nedd1	neural precursor cell expressed, developmentally down-regulated gene 1	-3,29	-0,88	10	92 114 544
6	1416395_at	BC024625	Guk1	guanylate kinase 1	-3,64	1,24	11	59 000 071
6	1439966_x_at	AV213621	Sfxn2	sideroflexin 2	-2,96	-0,54	19	46 626 675
6	1416923_a_at	AK018668	Bnip3l	BCL2/adenovirus E1B interacting protein 3-like	-2,92	-1,28	14	65 939 349
6	1451259_at	BC003445	Rexo2	REX2, RNA exonuclease 2 homolog (S. cerevisiae)	-3,08	1,30	9	48 220 480
6	1425633_at	M29007	BC026782	cDNA sequence BC026782	-3,50	-0,60	1	141 626 703
6	1436039_at	BM245957			-2,96	0,68	13	24 484 196
6	1424886_at	BC025145	Ptprd	protein tyrosine phosphatase, receptor type, D	-3,90	-1,82	4	75 412 469
6	1419874_x_at	AA419994	AI467657	expressed sequence AI467657	-2,93	-1,23	9	
6	1452348_s_at	AI481797	Ifi205	interferon activated gene 205	-4,13	0,40	1	175 848 673
6	1433857_at	AV088463	Fath	fat tumor suppressor homolog (Drosophila)	-2,71	-0,27	8	46 535 639
6	1451648_a_at	BC022108	Folr2	folate receptor 2 (fetal)	-3,40	-0,46	7	101 713 810
6	1429709_at	AI157548	Pmpcb	peptidase (mitochondrial processing) beta	-2,87	-0,31	5	21 248 983
6	1452737_at	AK012692	2810008M24Rik	RIKEN cDNA 2810008M24 gene	-2,62	-1,35	13	
6	1455350_at	BB667368	Tmem62	transmembrane protein 62	-3,64	0,28	2	120 668 502
6	1416489_at	NM_025951	Pi4k2b	phosphatidylinositol 4-kinase type 2 beta	-2,98	-0,70	5	53 029 858
6	1448261_at	NM_009864	Cdh1	cadherin 1	-3,95	-0,91	8	109 492 496
6	1419491_at	BC024380	Defb1	defensin beta 1	-3,13	-0,34	8	23 242 143
6	1419291_x_at	NM_013525	Gas5	growth arrest specific 5	-2,91	-0,38	1	
6	1436233_at	BM123923	AI117581	expressed sequence AI117581	-3,67	0,81	11	49 009 005
6	1435254_at	BM119522	Plxnb1	plexin B1	-3,13	-0,35	9	108 952 997
6	1425028_a_at	BC024358	Tpm2	tropomyosin 2, beta	-2,62	-0,17	4	43 535 811
6	1441331_at	BB153043	A230061C15Rik	RIKEN cDNA A230061C15 gene	-3,12	0,03	10	
6	1443860_at	BE979672	Ptprd	protein tyrosine phosphatase, receptor type, D	-3,32	-1,38	4	75 412 469
6	1430318_at	BB745042	Sat2	spermidine/spermine N1-acetyl transferase 2	-3,49	-1,85	11	69 438 257
6	1434410_at	AV276428	BC043118	cDNA sequence BC043118	-3,28	-1,54	16	59 434 352
6	1453540_at	BM218780	Phldb2	pleckstrin homology-like domain, family B, member 2	-3,34	-1,72	16	45 665 575
6	1456072_at	AU067663	Ppp1r9a	protein phosphatase 1, regulatory (inhibitor) subunit 9A	-3,39	1,65	6	4 853 319
6	1417120_at	NM_133873	D4Wsu114e	DNA segment, Chr 4, Wayne State University 114, expressed	-3,06	-0,43	4	146 704 591
6	1448426_at	BI217574	Sardh	sarcosine dehydrogenase	-3,08	-0,21	2	27 011 391
6	1424715_at	BB775176	Retsat	retinol saturase (all trans retinol 13,14 reductase)	-3,12	2,07	6	72 528 136
6	1451040_at	AI451865	Hars2	histidyl tRNA synthetase 2	-2,74	0,64	2	144 291 425
6	1431980_a_at	AK009814	As3mt	arsenic (+3 oxidation state) methyltransferase	-3,50	0,59	19	46 760 783
6	1417785_at	NM_134102	Pla1a	phospholipase A1 member A	-2,75	-0,31	16	38 315 357
6	1454649_at	AV003635	Srd5a1	steroid 5 alpha-reductase 1	-3,46	0,40	13	70 040 821
6	1423731_at	BC013548	Aldh16a1	aldehyde dehydrogenase 16 family, member A1	-3,12	0,84	7	45 009 883
6	1447543_at	BB225041	Wdfy1	WD repeat and FYVE domain containing 1	-3,36	0,86	1	79 605 439
6	1417042_at	NM_008063	Slc37a4	solute carrier family 37 (glycerol-6-phosphate transporter), member 4	-3,03	-1,23	9	44 149 170
6	1419096_at	NM_018816	Apom	apolipoprotein M	-2,96	-0,65	17	34 737 053
6	1448615_at	AF173379	Ccs	copper chaperone for superoxide dismutase	-3,49	1,97	19	4 825 373
6	1441198_at	BB311524	Zfp39	zinc finger protein 39	-3,36	-0,30	11	58 704 347
6	1417211_a_at	NM_023483	1110032A03Rik	RIKEN cDNA 1110032A03 gene	-2,75	-1,46	9	50 515 057
6	1416389_a_at	NM_134083	Rcbtb2	regulator of chromosome condensation (RCC1) and BTB (POZ) domain containing protein 2	-3,10	-1,83	14	71 876 342
6	1426089_a_at	BC003331	BC003331	cDNA sequence BC003331	-2,58	-1,25	1	152 124 753
6	1450970_at	AA792094	Got1	glutamate oxaloacetate transaminase 1, soluble	-3,26	-1,00	19	43 553 065
6	1435568_at	AW545855	AK129128	cDNA sequence AK129128	-2,88	-1,65	13	
6	1416101_a_at	NM_015786	Hist1h1c	histone 1, H1c	-2,78	-0,71	13	23 746 271
6	1453181_x_at	BF319989	Plscr1	phospholipid scramblase 1	-2,85	-1,24	9	92 066 797
6	1426246_at	Z25469	Pros1	protein S (alpha)	-3,32	0,85	16	62 796 589
6	1447520_at	AW208574			-2,89	-0,70	2	
6	1419441_at	NM_007475	Arbp	acidic ribosomal phosphoprotein P0	-3,08	-0,49	5	115 820 513
6	1426622_a_at	BB150720	Qpct	glutaminyl-peptide cyclotransferase (glutaminyl cyclase)	-2,91	-0,79	17	78 956 963
6	1436555_at	AV244175	Slc7a2	solute carrier family 7 (cationic amino acid transporter, y+ system), member 2	-2,62	-1,48	8	42 361 228
6	1451326_at	BC019410	Abhd14b	abhydrolase domain containing 14b	-2,73	0,22	9	106 306 761
6	1436185_at	BG076313	AI314180	expressed sequence AI314180	-2,64	-1,22	4	58 894 086
6	1455169_at	BG076094	Rab11fip2	RAB11 family interacting protein 2 (class I)	-2,58	-0,96	19	59 958 358
6	1424002_at	BC005601	Pdcl3	phosducin-like 3	-2,70	-1,44	1	38 932 359
6	1459853_x_at	AV312901			-2,92	-0,97	?	

6	1429181_at	AK005804	1700009P17Rik	RIKEN cDNA 1700009P17 gene	-2,59	-0,90	1	
6	1424962_at	BC010814	Tm4sf4	transmembrane 4 superfamily member 4	-3,25	-1,76	3	57 513 387
6	1452975_at	AK005060	Agxt2l1	alanine-glyoxylate aminotransferase 2-like 1	-2,99	-0,85	3	130 606 729
6	1417930_at	NM_008668	Nab2	Ngfi-A binding protein 2	-2,63	-0,10	10	127 063 867
6	1423119_at	AK016473	Rshl2a	radial spokehead-like 2A	-3,03	-1,91	17	7 784 156
6	1429699_at	AK016905	Oxsm	3-oxoacyl-ACP synthase, mitochondrial	-2,61	-1,25	14	15 031 925
6	1433659_at	BB503221	Trp53bp1	transformation related protein 53 binding protein 1	-2,74	-1,25	2	120 889 692
6	1427321_s_at	AK004908	Cxadr	coxsackievirus and adenovirus receptor	-3,11	-0,40	16	78 184 282
6	1459962_at	BM123926	4930523C07Rik	RIKEN cDNA 4930523C07 gene	-3,53	-1,82	1	161 881 063
6	1437354_at	AI154956	Ube3a	ubiquitin protein ligase E3A	-2,64	-0,42	7	59 096 620
6	1419095_a_at	NM_018816	Apom	apolipoprotein M	-3,30	0,07	17	34 737 053
6	1421906_at	BB760479	Pparbp	peroxisome proliferator activated receptor binding protein	-3,06	-1,69	11	97 970 338
6	1430802_at	AK013097	H2Q8	histocompatibility 2, Q region locus 8	-2,71	0,40	17	35 002 151
6	1436910_at	BB529484	Rasal2	RAS protein activator like 2	-2,94	0,14	1	
6	1423667_at	BC003451	Mat2a	methionine adenosyltransferase II, alpha	-2,65	-1,23	6	72 362 307
6	1430570_at	AV227891	Kynu	kynureninase (L-kynurenine hydrolase)	-2,61	0,37	2	43 377 337
6	1459679_s_at	AA406997	Myo1b	myosin IB	-2,77	-0,49	1	51 694 312
6	1448364_at	U95826	Ccng2	cyclin G2	-2,71	0,75	5	94 342 460
6	1439170_at	AU067772			-2,72	-1,79	8	34 869 859
6	1416531_at	NM_010362	Gsto1	glutathione S-transferase omega 1	-2,78	-1,23	19	47 908 299
6	1416480_a_at	NM_019814	Higd1a	HIG1 domain family, member 1A	-2,95	-1,64	9	121 697 260
6	1451154_a_at	BB644164	Cugbp2	CUG triplet repeat, RNA binding protein 2	-3,21	0,07	2	6 459 141
6	1421989_s_at	BF786072	Papss2	3'-phosphoadenosine 5'-phosphosulfate synthase 2	-2,71	-1,92	19	32 686 001
6	1453238_s_at	AU018141	A130040M12Rik	RIKEN cDNA A130040M12 gene	-3,10	0,56	4	
6	1434550_at	AW146284	3830406C13Rik	RIKEN cDNA 3830406C13 gene	-2,91	-0,94	14	11 077 509
6	1418780_at	NM_018887	Cyp39a1	cytochrome P450, family 39, subfamily a, polypeptide 1	-2,68	-1,93	17	43 130 479
6	1448989_a_at	AI255256	Myo1b	myosin IB	-2,90	0,08	1	51 694 312
6	1441975_at	BB008092	Acpp	acid phosphatase, prostate	-3,11	-0,04	9	104 157 344
6	1424988_at	BC010206	Mylip	myosin regulatory light chain interacting protein	-3,05	-0,43	13	45 400 713
6	1455007_s_at	BI648645	Gpt2	glutamic pyruvate transaminase (alanine aminotransferase) 2	-2,99	-0,44	8	88 382 721
6	1418012_at	BB221842	Sh3glb1	SH3-domain GRB2-like B1 (endophilin)	-2,72	0,14	3	144 626 229
6	1425193_at	BC026578	2010106G01Rik	RIKEN cDNA 2010106G01 gene	-2,77	-0,02	2	126 585 573
6	1460359_at	AK004598	Armcx3	armadillo repeat containing, X-linked 3	-2,83	-0,37	X	130 102 962
6	1426223_at	BC020021	2810439F02Rik	RIKEN cDNA 2810439F02 gene	-2,87	-0,40	18	12 786 968
6	1437798_at	BB051012	Lphn2	latrophilin 2	-2,97	-1,80	3	148 868 221
6	1418117_at	NM_010887	Ndufs4	NADH dehydrogenase (ubiquinone) Fe-S protein 4	-2,68	-0,49	13	115 409 528
6	1424828_a_at	BC006048	Fh1	fumarate hydratase 1	-2,91	0,09	1	177 438 058
6	1453022_at	AK003305	Gpihbp1	GPI-anchored HDL-binding protein 1	-2,68	0,04	15	75 423 912
6	1424683_at	BC019494	1810015C04Rik	RIKEN cDNA 1810015C04 gene	-2,67	-1,17	15	25 787 988
6	1456494_a_at	BG068242	AI451617	expressed sequence AI451617	-2,80	-1,69	7	104 345 671
6	1443670_at	AI451392	2010001J22Rik	RIKEN cDNA 2010001J22 gene	-2,80	0,82	15	89 205 215
6	1449007_at	NM_009770	Btg3	B-cell translocation gene 3	-2,61	-0,14	16	78 242 453
6	1435101_at	BB389641	Derl2	Der1-like domain family, member 2	-3,00	-0,79	11	70 823 639
6	1454609_x_at	BB770958	6430527G18Rik	RIKEN cDNA 6430527G18 gene	-3,03	-1,52	12	87 769 805
6	1425862_a_at	U52193	Pik3c2a	phosphatidylinositol 3-kinase, C2 domain containing, alpha polypeptide	-2,84	-0,13	7	116 130 709
6	1417431_a_at	AF245448	Sphk2	sphingosine kinase 2	-2,78	-1,07	7	45 578 326
6	1451381_at	BC026557	1810020D17Rik	RIKEN cDNA 1810020D17 gene	-2,68	0,44	7	97 425 534
6	1431086_s_at	AA389937	Pcmt1	protein-L-isoaspartate (D-aspartate) O-methyltransferase 1	-2,59	-1,44	10	7 320 426
6	1443252_at	BB179497	Ibrdc2	IBR domain containing 2	-2,72	0,41	13	47 133 691
6	1424717_at	BC026790	Mis12	MIS12 homolog (yeast)	-2,78	-1,91	11	70 835 805
6	1439845_at	BQ174438			-2,93	-1,56	4	
6	1455411_at	AI648923	Aqp11	aquaporin 11	-2,82	-0,26	7	97 601 570
6	1416939_at	NM_026438	Ppa1	pyrophosphatase (inorganic) 1	-2,78	0,15	10	61 043 977
6	1418449_at	NM_133664	Lad1	ladinin	-2,60	0,54	1	137 635 008
6	1426454_at	AK002516	Arhgdib	Rho, GDP dissociation inhibitor (GDI) beta	-2,80	0,87	6	136 887 904
6	1454712_at	AW212577	Mcart1	mitochondrial carrier triple repeat 1	-2,97	-0,76	4	45 417 165
6	1419829_a_at	AW049055	Gab2	growth factor receptor bound protein 2-associated protein 2	-2,73	-1,70	7	96 956 942
6	1418892_at	AF309564	Rhoj	ras homolog gene family, member J	-2,86	-1,26	12	76 227 152
6	1440786_x_at	BB377927	Sfxn2	sideroflexin 2	-2,65	-0,72	19	46 626 675
6	1421212_at	NM_018795	Abcc6	ATP-binding cassette, sub-family C (CFTR/MRP), member 6	-2,59	-0,78	7	45 844 421
6	1460696_at	BC026585	BC026585	cDNA sequence BC026585	-2,70	-0,36	1	159 295 258

6	1443872_at	AW493461	March2	membrane-associated ring finger (C3HC4) 2	-2,66	-1,49	17	33 292 398
6	1419398_a_at	NM_007874	Reep5	receptor accessory protein 5	-2,76	-0,42	18	34 469 688
6	1451421_a_at	BC006914	Rogdi	rogdi homolog (Drosophila)	-2,77	-1,44	16	4 924 070
6	1423895_a_at	BB644164	Cugbp2	CUG triplet repeat, RNA binding protein 2	-2,58	-0,05	2	6 459 141
6	1421900_at	BB077436	Eif2ak1	eukaryotic translation initiation factor 2 alpha kinase 1	-2,67	-1,06	5	144 073 457
6	1459731_at	BE996194			-2,86	-0,78	11	88 518 011
6	1419067_a_at	BB280137	Rabgef1	RAB guanine nucleotide exchange factor (GEF) 1	-2,60	-0,72	5	130 471 882
6	1422804_at	NM_011454	Serpinb6b	serine (or cysteine) peptidase inhibitor, clade B, member 6b	-2,71	-0,63	13	32 972 977
6	1434021_at	BG064982	C230096C10Rik	RIKEN cDNA C230096C10 gene	-2,61	-1,72	4	138 624 668
6	1418704_at	NM_009113	S100a13	S100 calcium binding protein A13	-2,70	-0,18	3	90 600 361
6	1415853_at	NM_054046	Def8	differentially expressed in FDCP 8	-2,71	-1,16	8	126 329 075
6	1427016_at	BC027125	4932438A13Rik	RIKEN cDNA 4932438A13 gene	-2,81	-0,85	3	37 228 204
6	1441539_at	BB532135			-2,73	-0,36	6	
6	1427548_a_at	U53455	Clns1a	chloride channel, nucleotide-sensitive, 1A	-2,60	0,13	7	97 571 882
6	1436842_at	AV229336	B230380D07Rik	RIKEN cDNA B230380D07 gene	-2,62	-0,20	9	70 403 759
6	1422811_at	NM_011977	Slc27a1	solute carrier family 27 (fatty acid transporter), member 1	-2,65	-1,80	8	74 497 915
6	1445980_at	BG076366	Aldh1a1	aldehyde dehydrogenase family 1, subfamily A1	-2,69	-1,45	19	20 669 078
6	1429326_at	BB538449	Cenpl	centromere protein L	-2,65	-1,10	1	162 907 617

Genes were selected as described in Materials and Methods. Values are the mean of 3 S-scores comparing D2 versus B6 expression.
